# Combined ctDNA and serum PSA for dynamic monitoring of metastatic prostate cancer starting first-line treatment: a prospective national cohort study

**DOI:** 10.1038/s43018-026-01172-9

**Published:** 2026-05-15

**Authors:** Anuradha Jayaram, Memuna Rashid, Alison H. M. Reid, Francesco Orlando, Suparna Thakali, Leila Zakka, Miriam Goncalves, Jacqueline O’Dwyer, Constantine Alifrangis, Rob Jones, Elias Pintus, Sarah Needleman, Diletta Bianchini, Anna Wingate, Kenrick Ng, Mark Linch, Ursula McGovern, John Staffurth, Simon J. Crabb, Susannah Brock, Alison Birtle, Osvaldas Vainauskas, Gianmarco Leone, Millenn Chiwewe, Allan Hackshaw, Andre Lopes, Daniel Wetterskog, Francesca Demichelis, Gerhardt Attard

**Affiliations:** 1 University College London Cancer Institute, London, UK; 2 Department of Oncology, University College London Hospitals NHS Trust, London, UK; 3 Cancer Research UK and University College London Cancer Trials Centre, University College London, London, UK; 4 The Royal Marsden NHS Foundation Trust, London, UK; 5 Kingston Hospital NHS Foundation Trust, London, UK; 6 Laboratory of Computational and Functional Oncology, Department of Cellular, Computational, and Integrative Biology, University of Trento, Trento, Italy; 7 The Christie NHS Foundation Trust, Manchester, UK; 8 University of Glasgow, Beatson West of Scotland Cancer Centre, Glasgow, UK; 9 Guy’s and St Thomas’ NHS Foundation Trust, London, UK; 10 Royal Free NHS Foundation Trust, London, UK; 11 Medway Maritime Hospital NHS Foundation Trust, Gillingham, UK; 12 Bart’s Health NHS Trust, London, UK; 13 Velindre Cancer Centre, Velindre University NHS Trust, Cardiff, UK; 14 University Hospital Southampton NHS Foundation Trust, Southampton, UK; 15 Royal Bournemouth Hospital, Bournemouth, UK; 16 Lancashire Teaching Hospitals NHS Foundation Trust, Preston, UK; 17 University of Central Lancashire, Preston, UK; 18 University of Manchester, Manchester, UK

**Keywords:** Tumour biomarkers, Prostate cancer, Cancer

## Abstract

The prognosis of newly diagnosed metastatic prostate cancer is highly variable. The primary objective of the PARADIGM prospective cohort study was to evaluate predictors of survival in blood collected at the start of each of the first six treatment cycles from 114 patients with high-volume metastatic prostate cancer (biologically male) who were starting androgen deprivation therapy in combination with docetaxel or an androgen receptor pathway inhibitor. Here circulating tumor DNA (ctDNA) was detected in 29% of patients after 6–12 weeks of combination therapy (compared to 70% before any treatment) and associated with 12 month overall survival of 73% versus 99% for patients who were ctDNA-negative and 24 month survival of 50% versus 85%. The secondary objective was to test ctDNA with serum prostate-specific antigen (PSA). In multivariable models, both were independent risk factors on combination treatment with a hazard ratio of death of 20.34 for the poorest prognosis group, but only ctDNA was associated with shorter survival on androgen deprivation before the start of combination therapy. Using ctDNA with serum PSA and clinical characteristics can improve the accuracy of survival prediction and should be evaluated for ctDNA-informed treatment modification. ClinicalTrials.gov: NCT04067713.

## Abstract (web summary)

Jayaram et al. assessed the performance of the combination of circulating tumor DNA and serum prostate-specific antigen for dynamic monitoring of metastatic prostate cancer at first-line treatment in this prospective observational clinical trial.

## Main

Prostate cancer was the fifth leading cause of cancer deaths in 2022, with 400,000 deaths globally^1,2^. Approximately half of patients who die from prostate cancer present with metastatic disease^3^. Metastatic prostate cancer often shows an initial response to androgen deprivation therapy (ADT) and more profound responses with an improvement in survival when combined with docetaxel chemotherapy or an androgen receptor pathway inhibitor (ARPI) in a doublet regimen^4–9^. Better treatments have also resulted in highly variable survival outcomes. Patients classified as having high-volume metastases (that is, visceral metastases and/or four or more bone metastases) at diagnosis or relapse^5^ have a poorer prognosis; however, although approximately 30% died within 24 months, a similar proportion were in remission after 8 years^8,9^. Tests that predict survival will enable targeted treatment modifications to improve outcomes while minimizing over-treatment.

Given the highly variable natural history and the ubiquitous availability of testing for the circulating tumor marker PSA, prostate cancer is well-placed for treatment modifications based on blood-based monitoring. However, PSA has several limitations. In patients with metastatic cancer starting their first systemic treatment, nadir PSA levels (≤0.2, 0.2–4 and >4 ng ml^−1^) are prognostic, but the clinical utility for informing treatment decisions is limited by the long time it takes to reach these nadir levels^10–13^. In addition, PSA is regulated by the androgen receptor and therefore reflects tumor dynamics in the context of androgen receptor signaling^14^. Consequently, as several studies have reported, PSA-based changes do not adequately capture treatment effects^15^.

ctDNA can be used to detect minimal residual disease and resistant clones many months before radiological progression^16,17^. In colon, lung or bladder cancer, ctDNA detection just a few weeks after curative-intent treatments identifies patients with residual disease that could benefit from adjuvant treatment^18–20^. In advanced estrogen-receptor-positive breast cancer, treatment modification based on the emergence of resistant clones in ctDNA delays progression and deterioration in quality of life^21^. ctDNA has been combined with blood protein tumor markers for improved screening and detection of minimal residual disease for example in colorectal and ovarian cancer^22^ but has not yet been used to dynamically track response when patients with metastatic cancer start treatment. By contrast, in metastatic castration-resistant prostate cancer (mCRPC) relapsing after sequential treatments, ctDNA has been extensively studied and shown to associate with patient outcomes^23–27^. However, the opportunity to improve patient care through treatment change based on ctDNA detection in mCRPC is probably limited, given the high disease burden and intractable drug resistance. Therefore, ctDNA is currently only used clinically in prostate cancer for molecular detection of patients eligible for inhibition of poly(ADP-ribose) polymerase^28^.

**Fig. 1:**
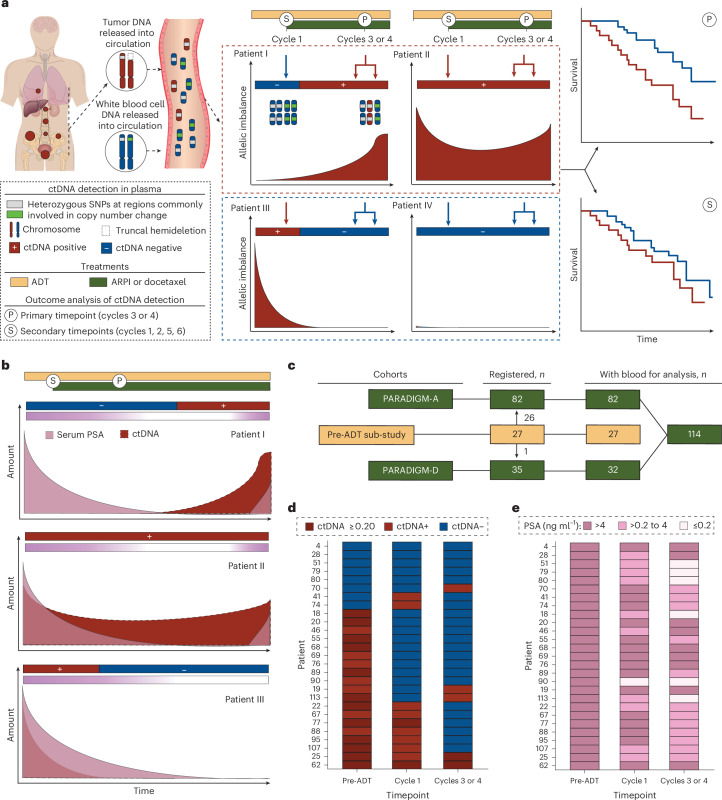
The PARADIGM study. **a**, Strategy for ctDNA detection in plasma using allelic imbalance at heterozygous single-nucleotide polymorphisms (SNPs). The middle panel demonstrates four scenarios of ctDNA dynamics (red shaded area) after the start of ADT. Horizontal bars demonstrate treatment type and ctDNA status across time. The study posited that ctDNA detection after the start of treatment is associated with poorer outcomes. ctDNA at cycles 3 or 4 was pre-defined as the timepoint of primary (P) interest for testing this hypothesis. Secondary timepoints (S) were then evaluated, including before the start of doublet therapy. **b**, The hypothesized relationship between ctDNA and serum PSA in three scenarios from **a**, highlighting potential differences in kinetics. In **a** and **b**, the *x* axis represents time. **c**, Patient flow in PARADIGM-A, PARADIGM-D and the pre-ADT sub-study (also see Extended Data Fig. 1). **d**,**e**, The 27 patients in the pre-ADT sub-study showing ctDNA status in sequential samples (d) and PSA categories for matched patients (**e**). ctDNA at cycle 3 or 4 was classified as positive if ctDNA was detected at either timepoint; for PSA, the cycle 4 value was used or else the cycle 3 value when cycle 4 PSA was not available.

Metastatic castration-sensitive prostate cancer (mCSPC) is a disease state of recent intense therapeutic development, with several positive phase 3 trials including a range of classes of agents^29^. We reasoned that combining ctDNA with PSA testing for metastatic prostate cancer starting systemic therapy could improve patient stratification and identify patients at high risk of relapse who could therefore benefit from treatment intensification. First-line hormone therapy often results in a decline of both PSA and ctDNA^30^, and residual detection may identify resistant clones many months before clinical or radiological manifestation. ctDNA is not directly regulated by androgen receptor signaling, unlike serum PSA, so it captures different elements of tumor activity^25^. Most targeted ctDNA assays track single-nucleotide variations that are less common and rarely recurrent in prostate cancer, which is characterized by copy number changes, including losses of 8p, 16q, *PTEN* and *RB1* (ref. ^31^). We established a structured biomarker roadmap to implement ctDNA analysis in the clinical management of mCSPC and used a computational approach designed to detect ctDNA using tumor-specific copy number changes or allelic imbalance, even at low circulating fractions^32^. Here, we report the first analysis of the Plasma Analysis for Response Assessment and to Direct the Management of Metastatic prostate cancer (PARADIGM) study, which prospectively evaluated associations with clinical outcomes of ctDNA from targeted next-generation sequencing classified using a pre-specified test (PCF_SELECT^32^), and serum PSA, first individually and then in combination, for patients initiating first-line treatment for mCSPC (Fig. 1a,b).

## Results

### Patient cohort

**Extended Data Fig. 1:**
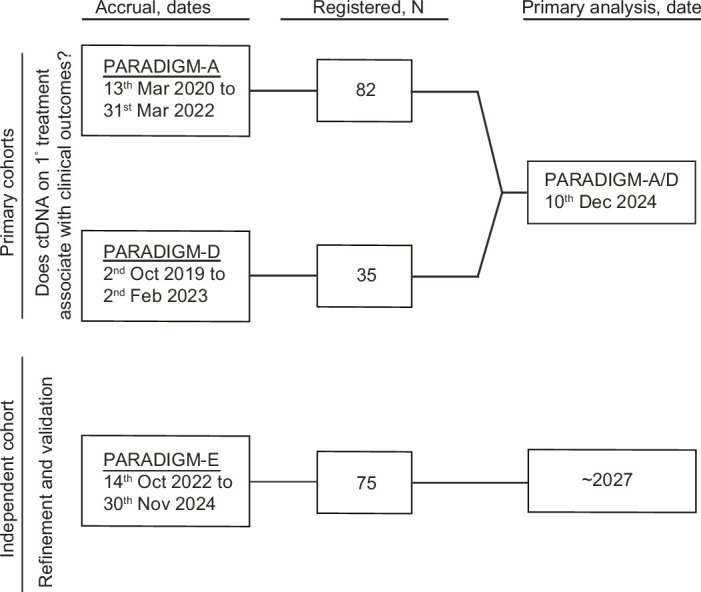
Patient recruitment to the PARADIGM study protocol. Patient flow in the three cohorts active in the PARADIGM protocol.

Between September 2019 and February 2023, 117 patients from 14 centres around the United Kingdom (Extended Data Fig. 1 and Supplementary Table 1) were recruited to the PARADIGM study. Of these, 114 commenced doublet therapy (32 with docetaxel and 82 with an ARPI) and gave a blood sample (Fig. 1c). To address the risks of multiple testing when evaluating associations of ctDNA and outcomes at multiple timepoints, we a priori defined ctDNA detection at cycles 3 or 4 as the primary timepoint of interest, as it would be early enough to institute a change in treatment. In total, 104 patients provided blood samples at this primary timepoint: either at both cycles (*n* = 59), or exclusively at cycle 3 (*n* = 38) or cycle 4 (*n* = 7). Of these, 31 patients received docetaxel in addition to ADT (PARADIGM-D) and 73 patients received an ARPI (PARADIGM-A). Baseline characteristics by treatment cohort and the types of ARPIs used in PARADIGM-A are outlined in Supplementary Tables 2 and 3. For the study population included in the primary timepoint analysis, the median age was 68 years, the median PSA before starting ADT was 171 ng ml^−1^, 72% (75 out of 104) of patients had a Gleason score of ≥8 and 92% (96 out of 104) had metastatic disease at diagnosis (synchronous). Docetaxel or ARPI were started a median of 57 days after initiation of ADT (range, 12–108 days). At cycle 3 or 4, 27% (28 out of 104) of patients had a serum PSA > 4 ng ml^−1^, 52% (54 out of 104) had a PSA of 0.2–4 ng ml^−1^ and the remainder (21%; 22 out of 104) had a PSA of ≤0.2 ng ml^−1^ (Supplementary Table 4).

Consistent with our pre-specified estimate, 29% (30 out of 104) of patients at cycles 3 or 4 had detectable ctDNA (32%; 10 out of 31) in PARADIGM-D and 27% (20 out of 73) in PARADIGM-A; the median ctDNA fraction among patients who were ctDNA-positive was 0.03 with an interquartile range (IQR) of 0.02–0.14. Patient baseline characteristics according to ctDNA detection at cycles 3 or 4 are listed in Table 1. Among patients who were ctDNA-positive, none had metachronous metastases, and only 7% of patients had visceral metastases (versus 24% of those who were ctDNA-negative).

**Table 1:** Patient characteristics according to ctDNA status at cycles 3 or 4

Baseline characteristics, *n* (%) ^*^	Overall cohort	ctDNA-negative	ctDNA-positive	*P* value
	104	74 (71%)	30 (29%)	
Age, in years				
Median (range)	68 (48–90)	69 (49–90)	64 (48–84)	0.14
Ethnicity				
Asian	4 (4%)	4 (5%)	0 (0%)	0.66
Black	8 (8%)	6 (8%)	2 (7%)	
White	87 (84%)	62 (84%)	25 (84%)	
Not recorded	5 (5%)	2 (3%)	3 (10%)	
Eastern Cooperative Oncology Group performance status				
0	54 (52%)	38 (51%)	16 (53%)	1.00
1–2	50 (48%)	36 (49%)	14 (57%)	
PSA before starting ADT^¥^ (ng ml^−1^)				
Median (range)	171 (2.6–5,000)	170 (2.5–5,000)	278 (16.8–3,295)	0.35
Gleason score				
≤7	14 (14%)	11 (15%)	3 (10%)	0.54
≥8	75 (72%)	51 (69%)	24 (80%)	
Not evaluable^†^	15 (14%)	12 (16%)	3 (10%)	
Presentation of metastases relative to diagnosis				
Metachronous	8 (8%)	8 (10%)	0 (0%)	0.10
Synchronous	96 (92%)	66 (90%)	30 (100%)	
Time on ADT^¥^ before day 1 cycle 1 (days)				
Median (range)	57 (12–108)	59 (18–106)	53 (12–108)	0.73
Visceral metastases				
No	84 (81%)	56 (76%)	28 (93%)	0.05
Yes^‡^	20 (19%)	18 (24%)	2 (7%)	

^*^ At study registration

*P* values stated are for the comparison between ctDNA, negative and positive. A two-sided Fisher’s exact test was used for categorical variables, and the Wilcoxon rank-sum test was used for continuous variables with no adjustment for multiple comparisons.

^†^Patients with a biopsy from any metastatic site (n = 6) or radiological or biochemical diagnosis (*n* = 9).

^‡^With or without bone metastases.

^¥^ADT in the form of luteinizing-releasing hormone antagonist or agonist.

### Circulating tumor DNA detection in sequential sampling from initiation of ADT

For patients with suspected metastatic disease, ADT is often started immediately at diagnosis. Obtaining a research blood sample before starting ADT may therefore not be feasible for every patient. To confirm the extent of ctDNA change with treatment in individual patient sequential samples, we conducted a sub-study that included 27 (out of 104) patients who, in addition to having blood samples taken at cycle 1 and cycles 3 or 4, also had blood samples taken before ADT. Comparing sequential timepoints in individual patients, ctDNA detection declined significantly after the start of ADT: 19 out of 27 samples (70%) collected before ADT were ctDNA-positive (median ctDNA fraction, 0.22; IQR, 0.04–0.31) compared to ten samples (37%; median ctDNA fraction, 0.04; IQR, 0.02–0.11) at cycle 1 (*P* = 0.02) and four samples (15%; median ctDNA fraction, 0.07; IQR, 0.01–0.15) at cycles 3 or 4 (*P* < 0.001) (Fig. 1d). One patient (4%) had ctDNA at cycle 3 or 4 but no ctDNA before ADT or at cycle 1. There was no notable difference in the ctDNA detection rate between samples collected at cycle 1 and cycles 3 or 4 (*P* = 0.23). Similarly, comparing high ctDNA levels (defined in our ctDNA calling algorithm as ≥0.20 tumor fraction; see Methods) in sequential ctDNA from individual patients, 12 out of 27 patients (44%) had a high ctDNA fraction before ADT compared to two out of of 27 patients (7%) at cycle 1 (*P* = 0.002) and two out of 27 patients (7%) at cycles 3 or 4 (*P* = 0.002) (Fig. 1d,e).

### Major outcomes according to detection of circulating tumor DNA at cycle 3 or 4

#### Progression-free survival

**Fig. 2:**
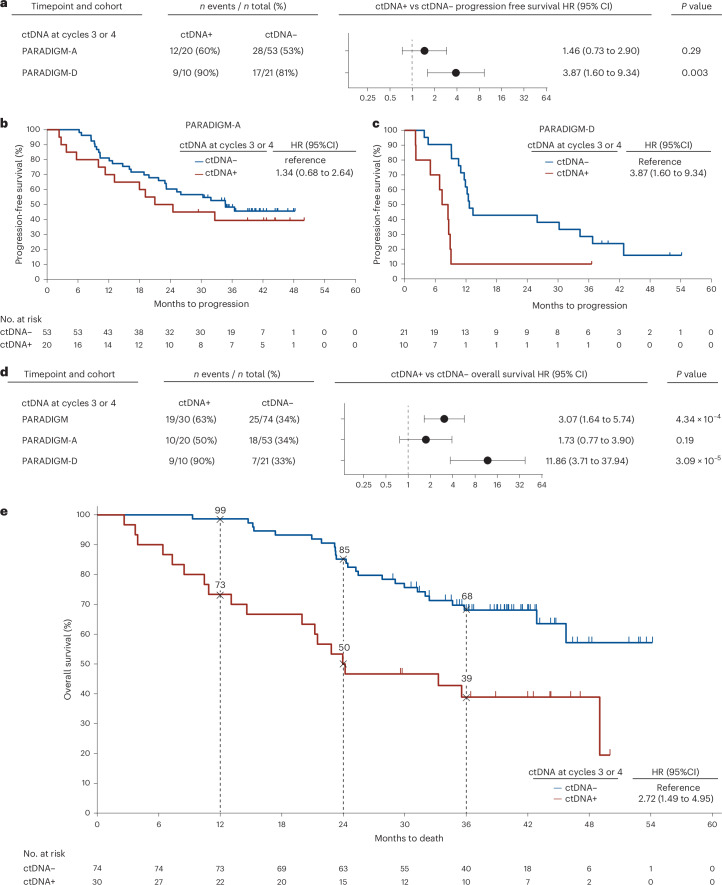
Primary endpoint: circulating tumor DNA at cycles 3 or 4. **a**, Forest plot of adjusted HR estimates for PFS in PARADIGM-A (*n* = 73 patients) and PARADIGM-D (*n* = 31 patients). **b**, PFS Kaplan–Meier curve for PARADIGM-A (*n* = 73 patients). **c**, PFS Kaplan–Meier curve for PARADIGM-D (*n* = 31 patients). **d**, Forest plot of adjusted HR estimates for OS for the total cohort included in primary analysis (*n* = 104 patients), PARADIGM-A and PARADIGM-D. **e**, Kaplan–Meier curve for OS for the total cohort included in primary analysis (*n* = 104 patients). For the forest plots, HRs were estimated by multivariable Cox proportional hazard models. The points marked by a circle represent the adjusted HR estimates, and the horizontal whiskers denote the corresponding 95% confidence intervals. The area to the right of the dotted line represents increasing risk of death or shorter time to progression in patients who were ctDNA-positive. Exact *P* values were reported from the Wald *z*-test without adjustments for multiple comparisons, and all statistical tests were two-sided. Event rates at pre-specified timepoints are indicated by the dotted lines. Tick marks indicate censored data. An unadjusted HR is stated on the Kaplan–Meier curve. Survival distributions were compared using the two-sided log-rank test. Source data

After a median follow-up of 41 months, 44 patients had died and 66 had progressed or died. Progression-free survival (PFS) for PARADIGM-D (median, 11.66 months; 95% CI, 8.90–13.37 months) and PARADIGM-A (median, 32.62 months; 95% CI, 21.68 months to not reached) (Supplementary Table 5) are reported separately owing to different definitions of disease progression for patients treated with docetaxel versus ARPI. In PARADIGM-D, the 12 month PFS rates were 10% (95% CI, 1–36%) for patients who were ctDNA-positive and 62% (95% CI, 38–79%) for those who were ctDNA-negative. In PARADIGM-A, patients who were ctDNA-positive had a 12 month PFS rate of 70% (95% CI, 45–85%), and those who were ctDNA-negative had a rate of 81% (95% CI, 68–89%) (Supplementary Table 6). In the PARADIGM-D cohort, the hazard ratio (HR) was 3.87 (95% CI, 1.60–9.34; *P* = 0.003), while the HR for PFS in the PARADIGM-A cohort was 1.46 (95% CI, 0.73–2.90; *P* = 0.29) (Fig. 2a–c). Consistent with this finding, in the PARADIGM-A cohort, age was the only variable associated with PFS (Supplementary Table 7; multivariable adjustments were not performed for PARADIGM-D owing to the smaller number of events). The landmark analyses showed a similarly strong association for ctDNA with PFS in PARADIGM-D (HR, 3.57; 95% CI, 1.43–8.90) but no association in PARADIGM-A (HR, 1.20; 95% CI, 0.57–2.51; Supplementary Table 8).

#### Overall survival

Unlike PFS, median overall survival (OS) was similar in PARADIGM-D (42.87 months, (95% CI, 23.95 months to not reached) and PARADIGM-A (49.02 months; 95% CI, 35.55 months to not reached; Supplementary Table 5)). The OS rate in both cohorts combined at 12 months was 73% (95% CI, 54–86%) for patients who were ctDNA-positive compared to 99% (95% CI, 91–100%) in those who were ctDNA-negative. The corresponding 24 month OS rates were 50% (95% CI, 31–66%) and 85% (95% CI, 75–91%), respectively. At 36 months, only 39% (95% CI, 22–56%) of the patients who were ctDNA-positive were alive compared to 68% (95% CI, 56–78%) of those who were ctDNA-negative (Supplementary Table 6). The median OS was shorter in patients who had detectable ctDNA at cycles 3 or 4 compared to those who were ctDNA-negative (median, 24 months versus not reached; HR, 3.07; 95% CI, 1.64–5.74; *P* < 0.001; Fig. 2d,e). The association was strongest in the PARADIGM-D cohort (HR, 11.86; 95% CI, 3.71–37.94; *P* < 0.001; Fig. 2d). Landmark analysis at treatment cycle 4 was consistent with these estimates, with median OS of 21.39 months (95% CI, 11.56 months to not reached) for patients who were ctDNA-positive, and not reached (95% CI, 40.80 months to not reached) for patients who were ctDNA-negative (HR, 3.01; 95% CI, 1.61–5.64; Supplementary Table 9). In multivariable models including ctDNA and all clinical variables used for adjustment, ctDNA at the primary timepoint was the only factor that had a significant association with OS (Supplementary Table 10).

### OS according to circulating tumor DNA detection at secondary timepoints

**Fig. 3:**
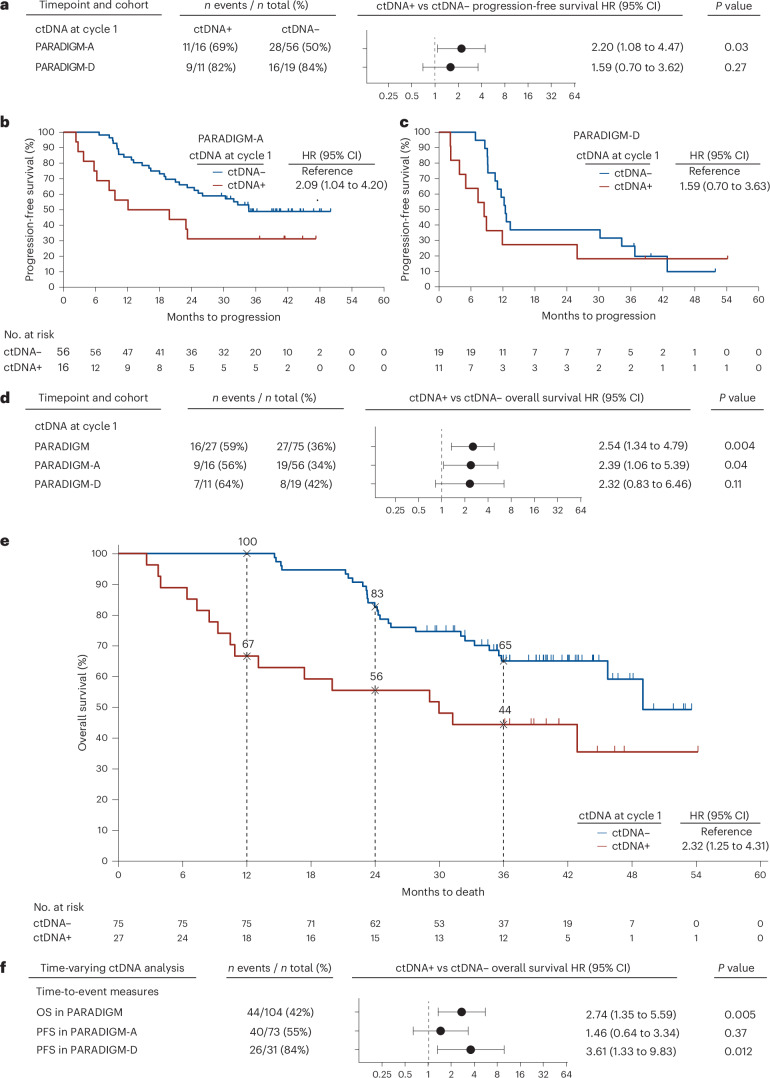
ctDNA at exploratory timepoints. Analyses by ctDNA status at cycle 1. **a**, Forest plot of adjusted HR estimates for PFS for PARADIGM-A (*n* = 72 patients) and PARADIGM-D (*n* = 30 patients). **b**, Kaplan–Meier curves of PFS for PARADIGM-A (*n* = 72 patients). **c**, Kaplan–Meier curves of PFS for PARADIGM-D (*n* = 30 patients). **d**, Forest plot of adjusted HR for OS for all patients with a cycle 1 sample (*n* = 102 patients), PARADIGM-A and PARADIGM-D. HRs in forest plots were estimated from a multivariable Cox proportional hazard model. The points marked by a circle represent the adjusted HR estimates, and the horizontal whiskers denote the corresponding 95% confidence intervals. Exact *P* values were reported from two-sided Wald *z*-tests without adjustments for multiple comparisons. **e**, Kaplan–Meier curves of OS for all patients with a cycle 1 sample (*n* = 102 patients). Survival distributions were compared using the two-sided log-rank test. Event rates at pre-specified timepoints are indicated by dotted lines. Tick marks indicate censored data. Unadjusted HR is stated on the Kaplan–Meier curve. **f**, Forest plot for time-varying analysis using information from every timepoint from all patients with a cycle 3 or 4 sample (*n* = 104 patients) for OS and for PFS split by PARADIGM-A (*n* = 73 patients) or PARADIGM-D (*n* = 31 patients). HRs were estimated from a time-dependent Cox proportional hazard model. The points marked by a circle represent HRs, and the horizontal whiskers denote the 95% confidence intervals. Exact *P* values were reported from two-sided Wald *z*-tests without adjustments for multiple comparisons. The area to the right of the dotted line represents increasing risk of death or shorter time to progression in patients who were ctDNA-positive. Source data

Having met the primary objective, we proceeded to evaluate the association of ctDNA at cycles 1, 2, 5 and 6. OS was prioritized as a more reproducible and clinically relevant endpoint. Of the 114 patients who started doublet therapy, 112 gave a blood sample at cycle 1. Amongst these patients, 102 also had ctDNA testing at cycles 3 or 4 (a blood sample was not collected from the other two patients at cycle 1). At cycle 1, 27 out of 102 patients (27%) were ctDNA-positive (median ctDNA fraction, 0.03; IQR, 0.02–0.30). CtDNA detection at cycle 1 was associated with PFS in PARADIGM-A (HR, 2.20; 95% CI, 1.08–4.47) but not in PARADIGM-D (HR, 1.59; 95% CI, 0.70–3.62) (Fig. 3a–c). There was an association between ctDNA detection at cycle 1 and worse OS (HR, 2.54; 95% CI, 1.34–4.79) (Fig. 3d,e). This result was similar in PARADIGM-A (HR, 2.39; 95% CI, 1.06–5.39) and PARADIGM-D (HR, 2.32; 95% CI, 0.83–6.46; Fig. 3d). Time-dependent analysis (adjusted for baseline variables) demonstrated that at any given time during follow-up, among all evaluable patients (*n* = 104), those who were ctDNA-positive had a higher hazard of death compared to patients who were ctDNA-negative (HR, 2.74; 95% CI, 1.35–5.59; *P* = 0.005) (Fig. 3f). Patients who were ctDNA-positive both at cycle 1 and cycles 3 or 4 had significantly worse OS (median, 8.5 months, 95% CI, 3.7–13.1 months; HR, 17.00, 95% CI, 6.64–43.47, *P* < 0.0001) compared to those who were ctDNA-negative at both timepoints (Supplementary Table 11).

### Secondary analyses of combined circulating tumor DNA and PSA for predicting survival

#### ctDNA and PSA at cycles 3 or 4

**Fig. 4:**
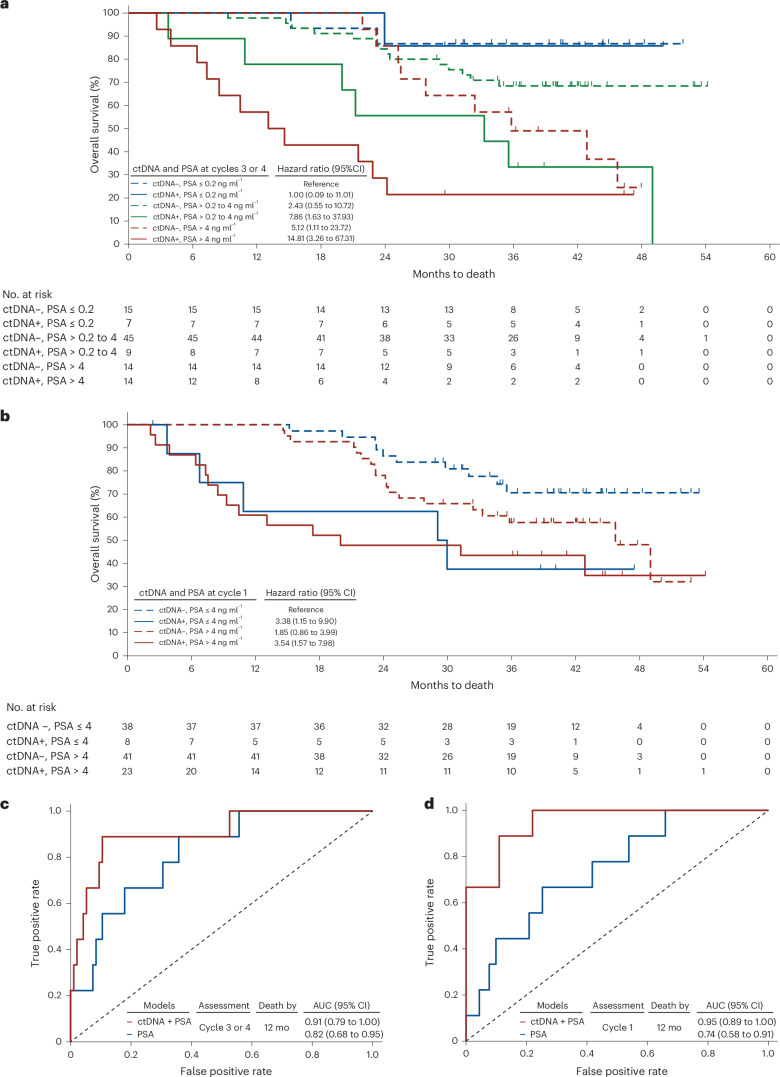
Combined circulating tumor DNA and PSA. **a**, Kaplan–Meier curves for OS by ctDNA status at cycles 3 or 4 split by serum PSA categories ≤0.2 ng ml^−1^ (*n* = 22 patients), >0.2 to 4 ng ml^−1^ (*n* = 54 patients) and >4 ng ml^−1^ (*n* = 28 patients) measured at cycle 4 or, if not available at cycle 4, at cycle 3 (*n* = 26 of 104 patients). **b**, Kaplan–Meier curves for OS by ctDNA status at cycle 1 (*n* = 110 patients) split by serum PSA ≤ 4 ng ml^−1^ (*n* = 46 patients) or >4 ng ml^−1^ (*n* = 64 patients). Tick marks indicate censored data. **c**,**d**, Receiver operating characteristic (ROC) curve for ctDNA prediction of deaths at 12 months for cycles 3 or 4 (*n* = 104 patients) (**c**) and for cycle 1 (*n* = 110 patients) (**d**). AUC, Area under the ROC Curve. Source data

**Extended Data Fig. 2:**
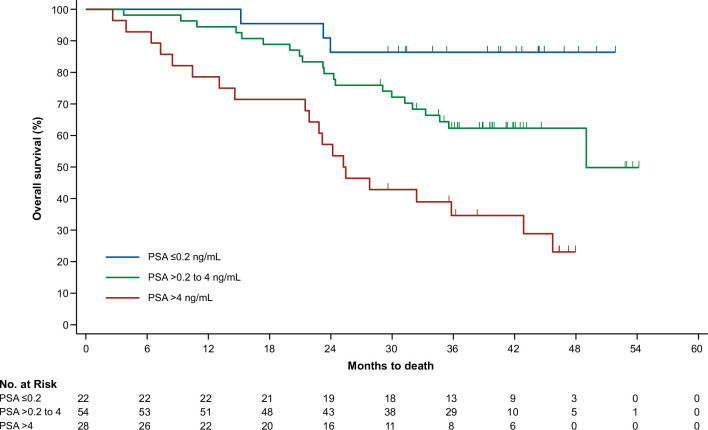
Overall survival by PSA at primary time-points. Kaplan-Meier curves for overall survival by serum PSA categories ≤0.2 ng/mL (*n* = 22 patients), >0.2 to 4 ng/mL (*n* = 54 patients) and >4 ng/mL (*n* = 28 patients). Serum PSA at cycle 4 was used or, if not available, a cycle 3 serum PSA was used. The ticks indicate censored data. Source data

The secondary objective of the study was to evaluate combinations of serum PSA categories, previously shown to be prognostic after 6–12 months of treatment^10,11^, and ctDNA. First we compared patients with PSA ≤ 0.2 ng ml^−1^ at cycles 3 or 4 to higher PSA values at this timepoint and identified that OS was worse for patients with PSA > 0.2 to 4 ng ml^−1^ (HR, 3.22; 95% CI, 0.94–11.05) or PSA > 4 ng ml^−1^ (HR, 9.07; 95% CI, 2.51–32.83) (Extended Data Fig. 2). In multivariable analyses, we identified that the associations between ctDNA and OS were at least as strong as between PSA and OS, and, importantly, that both ctDNA and PSA were independent risk factors for shorter survival (HR, 3.63; 95% CI, 1.94–6.81 for ctDNA and HR, 5.52; 95% CI, 1.65–18.39 for PSA > 0.2 ng ml^−1^) (Table 2). Including patients who were ctDNA-negative with PSA ≤ 0.2 ng ml^−1^ as the reference group, survival was significantly shorter for those who were ctDNA-positive with PSA > 0.2 to 4 ng ml^−1^ (HR, 9.24; 95% CI, 1.84–46.38) and PSA >4 ng ml^−1^ (HR, 20.34; 95% CI, 4.06–101.90) (Fig. 4a). To illustrate the value of a combination liquid biopsy, we also compared OS for ctDNA detection in patients within PSA categories. Although for patients with PSA ≤ 0.2 ng ml^−1^, ctDNA detection did not associate with shorter OS, OS was significantly shorter for ctDNA-positive patients with PSA > 0.2 to 4 ng ml^−1^ (HR, 2.93; 95% CI, 1.16–7.40) or >4 ng ml^−1^ (HR, 8.08; 95% CI, 2.06–31.70) (Supplementary Table 12).

**Table 2:** Multivariable analysis of ctDNA and PSA with OS

Analysis	Timepoint	Cox model contains	HR	95% CI	*P* value
I	Cycle 3 or 4 |	ctDNA+ vs ctDNA−	2.86	1.50, 5.43	0.001
		PSA > 4 vs ≤4	3.10	1.60, 5.99	0.001
II	Cycle 3 or 4 |	ctDNA+ vs ctDNA−	3.63	1.94, 6.81	5.76 × 10^−5^
		PSA > 0.2 vs ≤0.2	5.52	1.65, 18.39	0.005
III	Cycle 1 |	ctDNA+ vs ctDNA−	2.46	1.25, 4.82	0.009
		PSA > 4 vs ≤4	1.44	0.69, 3.02	0.330
IV	Cycle 3 or 4 |	ctDNA+ vs ctDNA−	4.28	2.13, 8.58	4.26 × 10^−5^
		PSA > 4 vs ≤4	3.59	1.66, 7.78	0.001
	Cycle 1 |	ctDNA+ vs ctDNA−	3.67	1.79, 7.55	3.94 × 10^−4^
		PSA > 4 vs ≤4	0.76	0.33, 1.78	0.532
V	Cycle 3 or 4 |	ctDNA+ vs ctDNA−	6.18	3.03, 12.57	5.14 × 10^−7^
		PSA > 0.2 vs ≤0.2	6.05	1.63, 22.42	0.001
	Cycle 1 |	ctDNA+ vs ctDNA−	2.49	1.24, 4.97	0.010
		PSA > 4 vs ≤4	0.83	0.40, 1.73	0.620

Five multivariable analyses were performed: ctDNA and serum PSA at cycle 3 or 4 (serum PSA ≥ 4 ng ml^−1^ and ≥0.2 ng ml^−1^ tested separately; analyses I and II, respectively) and cycle 1 (serum PSA ≥ 4 ng ml^−1^; analysis III) followed by ctDNA at both cycle 1 and cycle 3 or 4 with PSA at both cycle 1 (serum PSA ≥ 4 ng ml^−1^) and cycle 3 or 4 (serum PSA ≥ 4 ng ml^−1^ and ≥0.2 ng ml^−1^; analyses IV and V, respectively). A two-sided Wald test was conducted, and exact *P* values are provided. No adjustments for multiple comparisons were made.

#### ctDNA and PSA at cycle 1

Next, we examined the combination of ctDNA and PSA at cycle 1 (110 patients). In keeping with a slower dynamic for PSA, only five out of 110 patients (5%) had PSA < 0.2 ng ml^−1^ at cycle 1. We therefore categorized patients by PSA > 4 ng ml^−1^ or PSA ≤4 ng ml^−1^. On multivariable analysis, PSA at cycle 1 was not associated with OS, while ctDNA was (HR 2.46; 95% CI, 1.25–4.82) (Table 2). This was independent of ctDNA at cycles 3 or 4. ctDNA detection was associated with OS both when PSA was ≤4 ng ml^−1^ (HR, 3.88; 95% CI, 1.29–11.62) or >4 ng ml^−1^ (HR, 3.54; 95% CI, 1.54–8.10) (Fig. 4b).

### Dynamic improvement in survival prediction when circulating tumor DNA is combined with PSA

OS models including baseline clinical variables had a better fit when including ctDNA in addition to serum PSA (likelihood *P* < 0.001 for cycle 3 or 4 measurements and likelihood *P* = 0.012 for cycle 1 testing). We then tested the accuracy of survival prediction at 12 or 24 months after the start of treatment, chosen because all evaluable patients had follow-up of at least 24 months. Firstly, adding ctDNA to serum PSA collected at cycle 3 or 4 led to an increase in area under the curve for predicting OS at 12 months from 0.82 (95% CI, 0.68–0.95) to 0.91 (95% CI, 0.79–1.00) (Fig. 4c) and 24 months from 0.69 (95% CI, 0.57–0.82) to 0.73 (95% CI, 0.60–0.86). At cycle 1, the area under the curve for predicting OS at 12 months increased from 0.74 (95% CI, 0.58–0.91) to 0.95 (95% CI, 0.89–1.00) (Fig. 4d) and at 24 months, from 0.70 (95% CI, 0.58–0.82) to 0.74 (95% CI, 0.62–0.87).

## Discussion

The PARADIGM study prospectively shows that ctDNA detection after the start of doublet therapy as first-line treatment for metastatic prostate cancer identifies patients who are at high-risk of shorter survival. Cycles 3 or 4 were pre-defined as the primary timepoint because these were considered early enough to modify treatment with potentially the greatest effect. We also report that ctDNA becomes a risk factor before PSA and as early as after starting ADT and before doublet therapy. This result supports our hypothesis that ctDNA is more responsive than serum PSA and introduces the opportunity to use blood biomarkers to predict outcome at an earlier timepoint rather than waiting for serum PSA nadir. Our primary analysis reported from the start of treatment to allow comparisons of median times to event for samples collected at different timepoints. The time from the start of treatment to the primary endpoint blood draw was only 4% of the total follow-up time, and in landmark analysis, there was a consistent effect. Although ctDNA has been shown to be prognostic in mCRPC, the PARADIGM study results have the potential, if implemented in the next generation of clinical trials, to lead to ctDNA implementation for treatment intensification at the start of ADT. By defining changes in ctDNA sequentially from before ADT, we show that although ctDNA is sufficiently abundant for molecular analyses in some patients before treatment, a notable decrease in fraction will restrict the type of molecular studies possible after the start of ADT and an ARPI. We also show in the whole cohort that persistence of ctDNA on treatment is associated with a very poor prognosis and identifies a group of urgent unmet need.

Key strengths of our study included being multi-centre, having OS as a major endpoint, using serial plasma samples, being centralized, with blinded ctDNA measurements and a pre-specified classification for ctDNA detection to enable reproducibility. The study design was pragmatic and allowed for over-recruitment to the ARPI cohort if docetaxel use decreased. Several ctDNA tests use recurrent somatic point mutations for detecting ctDNA at low fractions^16,20^. We used a ctDNA panel that can be implemented for prostate cancer, as it also detects somatic allele-specific imbalance, characteristic of advanced prostate cancer, at sufficient sensitivity for on-treatment ctDNA detection in patients with poor prognosis. Circulating tumor cells associate with OS and PFS in metastatic prostate cancer, but because current technologies capture a relatively small number of circulating tumor cells at the start of ADT or at progression after several treatment lines, the decrease in tumor load we report with sequential sampling suggests possibly lower utility at the timepoints we report in this PARADIGM study^33,34^.

Although PFS was used to estimate study size initially, both PFS and OS were pre-specified to be joint major endpoints in later versions of the protocol, and the study was powered for both PFS and OS with sufficient follow-up. Data emerging over the course of the PARADIGM study identified a relatively moderate association between PFS and OS in mCSPC^15,35^, so we therefore reported associations with both PFS and OS in our primary analyses but prioritized testing of OS in secondary and exploratory analyses. Current use of PSA for initiating treatment change or new imaging can also influence time to recording of progression, potentially confounding the testing of PSA and ctDNA for predicting outcomes. In addition, increasing clinical use of diffusion weighted magnetic resonance imaging and positron emission tomography for patients with a rising PSA on ADT and ARPI is further affecting the PFS endpoint. Notably, we observe numerically stronger associations between ctDNA and the endpoint of OS rather than PFS, which may be partly explained by the difficulty of recording radiographic progression for this group of patients.

There were some limitations. As is commonly observed in clinical practice, there was some heterogeneity within the PARADIGM study in the time from diagnosis of metastatic disease to the start of treatment. Secondly, the PARADIGM-D cohort did not recruit the target number of patients owing to changes in national prescribing practices. Nonetheless, because of the large magnitude of the main associations with OS and PFS, these were statistically significant and therefore by definition, these analyses were not underpowered. At this primary analysis of OS, we combined patients receiving doublet therapy in either cohort, based on prior data showing that although time to progression differed between ARPI and docetaxel, there was no difference in median survival^36,37^. Consistent with these prior data, there was a numerical but not significant difference in OS between PARADIGM-D and PARADIGM-A. Although the difference in progression rates between patients who were ctDNA-positive and ctDNA-negative in PARADIGM-D was larger than we had estimated before study initiation, the progression rate among patients who were ctDNA-positive in PARADIGM-A was lower than estimated. Further work can assess ctDNA prognostic performance according to treatment type. Given that at the time of study design, there was no information on the evaluation of ctDNA on treatment in this disease setting, this first study was not large enough to have sufficient power to formally test every timepoint from cycle 1 to cycle 6. The protocol, therefore, pre-specified ctDNA testing after two or three cycles of combination treatment to prove the association with worse outcome, chosen as the primary endpoint because this is most pragmatically straightforward to implement in future clinical trials that use ctDNA to change management. Additional timepoints were included in exploratory analyses using time-varying analyses that support the association with shorter survival is consistent across timepoints (evaluated up to cycle 6). This observation and the strong association reported for cycle 1 ctDNA suggest that ctDNA testing after shorter durations of treatment could also be considered. The size of this study also limits the interpretation of tests of interaction between biomarkers (for example, ctDNA and PSA at different timepoints) and prevents us from assessing missing not-at-random mechanisms. Another limitation of the analysis is that it does not strictly follow the commonly cited guidelines of ten events per variable for multivariable Cox models^38,39^. This may introduce risk of over-fitting; however, the similarity between adjusted and unadjusted HR estimates provides reassurance regarding the stability of the model despite the limited number of events. In addition, covariates were selected a priori based on established clinical relevance rather than statistical significance within this dataset. A higher proportion of patients with visceral disease were ctDNA-negative at the primary timepoint, which, although interesting, was not expected and pre-stated as a hypothesis during the design of the trial. These considerations can be addressed in future work with larger cohorts.

Recently, combinations of ADT with both ARPI and docetaxel have been shown to be superior to doublet therapy with ADT and docetaxel alone^40,41^. These ‘triplet’ therapies were introduced into clinical practice after completion of accrual to the ARPI cohort. Given the potential detrimental impact on quality of life associated with docetaxel treatment^42^ and the potentially good outcomes on ADT and ARPI alone, there is now some uncertainty regarding the selection of patients for the addition of docetaxel to ARPI and ADT. This is more pertinent after recent trials reported positive results in mCSPC for combinations with PSMA-directed radioligand targeting^43^ or inhibition of poly(ADP-ribose) polymerase^44^ or the phosphoinositide 3-kinase (PI3K)–Akt signaling pathway in molecularly selected populations^45^.

In conclusion, this prospective evaluation reports strong associations with survival for residual ctDNA. This finding is important, as it could enable the initiation of life-prolonging treatment for patients with poor prognosis. Although more expensive than PSA testing, ctDNA is an independent risk factor after the start of doublet therapy for metastatic prostate cancer and improves the prediction of survival when combined with PSA to dynamically predict survival. The use of discrete categorical classifiers for ctDNA and PSA allows a path to clinical implementation, allowing assignment of patients to pre-defined prognostic subgroups. These data support the conduct of clinical trials that formally assess whether modifying treatment with ADT and an ARPI based on ctDNA detection improves patient outcomes.

## Methods

### Study design and patient population

PARADIGM was a national, multi-centre, prospective, observational cohort study, registered at ClinicalTrials.gov (NCT04067713), sponsored by University College London (UCL). The study received national ethics approval from the Health Research Authority ethics committee at Brighton and East Sussex on 15 April 2019. All participants provided written informed consent. This primary report adheres to the Strengthening the Reporting of Observational Studies in Epidemiology guidelines for cohort studies. Patients were eligible for registration in the study if they had high-volume, metastatic prostate cancer defined as ≥5 bone metastases (on whole body technetium-99m bone scan) or ≥1 unequivocal visceral metastasis (on computed tomography scans (or equivalent) of the chest, abdomen and pelvis). Patients who had relapsed (metachronous) and had received radical treatment with either prostatectomy or radiotherapy were eligible. Patients were excluded if they had a concurrent malignancy or were planned for surgery or radiotherapy before cycle 4. All patients were biologically male. Registered patients were included in the analysis if they gave a blood sample and started doublet therapy within 16 weeks of the first dose of luteinizing hormone-releasing hormone agonist or 14 weeks of starting a luteinizing hormone-releasing hormone antagonist. Detailed inclusion and exclusion criteria are defined within the protocol, available online. Patients treated with docetaxel (administered in three weekly cycles up to a maximum of six cycles) were included in the PARADIGM-D cohort. Patients treated with an ARPI (abiraterone acetate with prednisolone or enzalutamide or apalutamide, administered continuously in four weekly cycles until progression) were included in the PARADIGM-A cohort. There was no randomization, and the treatment cohort was based on local practice. The clinical data cut for analysis was 10 December 2024.

### Laboratory and clinical outcomes data

Patients were followed up with tumor assessments, including blood for PSA every 1–3 months, and imaging, done at 6–12 month intervals or when clinically indicated. Research blood samples for ctDNA analysis were collected at the same time as PSA on day 1 of each treatment cycle (or up to 5 days prior) from cycles 1 to 6, including for a subset of patients, before the start of ADT (pre-ADT sub-study). Research blood was processed centrally at the UCL Cancer Institute by staff who were blinded to patient characteristics and outcomes. Samples were analyzed for ctDNA at completion of accrual (investigators and patients therefore had no knowledge of ctDNA results) and before extraction of any clinical data.

PFS was defined as the interval from the start of docetaxel or ARPI to disease progression, determined by the first occurrence of any of the following: new or unequivocal progression of distant metastases confirmed by imaging, symptomatic progression of cancer in the prostate confirmed by imaging or death from any cause. To reflect international practice, a rise in PSA was included in the definition of progression for patients who had received docetaxel. Therefore, PFS was reported separately for patients who had been given docetaxel (PARADIGM-D) or ARPI (PARADIGM-A). OS was defined as the interval from the start of docetaxel or ARPI to death from any cause and was reported for the overall cohort, with subgroup analyses by treatment cohort. Additional landmark analyses were performed for both OS and PFS at cycle 4.

### Experimental procedures

A total of 40 ml of blood was collected for ctDNA analysis in EDTA or PAXGene circulating cell-free DNA (ccfDNA) tubes. All samples were handled as per the laboratory manual provided to each site and transferred at room temperature (20 °C to 24 °C) to the Cancer Biomarker Centre at UCL Cancer Institute.

For all samples, ccfDNA was extracted from 4–10 ml of plasma using the QIAamp Circulating Nucleic Acid kit (Qiagen) as per the manufacturer’s instructions, with the exception of the incubation period at 60 °C, which was extended to 1 h instead of 30 min. Germline DNA was extracted from 0.2 ml of buffy coat using QIAamp DNA Blood Mini Kit (Qiagen) as per the manufacturer’s instructions. The extracted plasma and germline DNA were then quantified using the Qubit dsDNA high-sensitivity or broad range kit, respectively. Germline DNA samples were first sonicated to 150–200 bp size using Covaris before library preparation. Indexed whole-genome libraries (WGLs) were generated from 20 ng of ccfDNA and 100 ng of germline DNA using the KAPA Hyperprep kit (Roche). WGLs generated from ccfDNA and germline DNA samples were subjected to target enrichment using the Roche targeted capture protocol, KAPA HyperCap Workflow and a custom targeted next-generation sequencing panel (PCF-SELECT probe panel v.3, 2.7 Mbp)^32^. In brief, eight plasma WGLs (250 ng each) or eight germline WGLs (250 ng each) were pooled and hybridized with PCF-SELECT probes overnight before isolation of target fragments and post-capture amplification was performed according to the manufacturer’s protocol. The PCF_SELECT panel captures genes frequently altered in prostate cancer and includes >20,000 high minor allele frequency single-nucleotide polymorphisms (SNPs) in 94 target and control gene regions. Target enrichment sequencing was performed on an Illumina NovaSeq 6000 platform using 100 bp paired-end reads. A minimum average coverage of 500× for plasma DNA and 200× for germline DNA in targeted regions was required for post-sequencing analyses.

### Data processing

Sequencing data were pre-processed, and paired-end reads were trimmed to remove adaptors using trimadap (https://github.com/lh3/trimadap). The data were then aligned to the human G1Kv37 reference genome using BWA-MEM^46^. Duplicate reads were marked and removed using Picard MarkDuplicates, and realignment and recalibration were performed using GATK^47^. Overlapping read pairs were clipped using bamUtil (https://github.com/statgen/bamUtil). To confirm that there was correct matching between plasma and germline DNA from the same individual, a sample identity check was applied^48^. If the assigned patient-matched samples had a correlation value of >95%, they were designated as correctly matched and were taken forward for ctDNA estimation.

**Extended Data Fig. 3:**
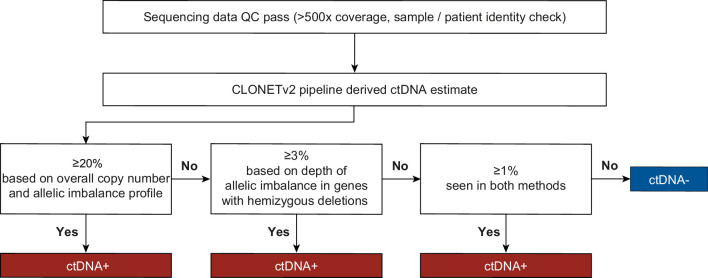
Decision tree for assigning circulating tumor DNA status. Copy number and allelic imbalance calls are outputs from the CLONET version 2 pipeline as implemented in the PCF-SELECT framework.

ctDNA fractions and allele-specific copy numbers were computed by integrating read-depth estimations and allelic imbalance calls using the CLONETv2 computational pipeline. The read-depth estimations were GC-content corrected and normalized using the mean depth coverage. A pre-computed reference model was used for allelic imbalance computation based on heterozygous SNPs assessed from a panel of 40 normal samples. Allelic imbalance and read-depth were then calculated per gene region, including the exonic regions and flanking non-coding regions extending 200 kb on either side^49^. ctDNA fraction was estimated using the abundance of allelic imbalance calculated as 1 − (β / (2 − β)), where β is the proportion of neutral reads (that is, the number of reads with matching heterozygous SNPs). Patients were classified as ctDNA-positive when allelic imbalance was detected (≥0.01) using a pre-specified decision tree (Extended Data Fig. 3). A ctDNA fraction of ≥0.2 also required confirmation from log_2_ copy number ratios.

### Statistics and reproducibility

There was no randomization and no blinding to treatment. All patients with a blood sample at cycle 3 or 4 were included in primary and exploratory analyses; additionally, patients with a blood sample at cycle 1 were included in the analysis of this timepoint of combinations of ctDNA and serum PSA. To detect a 20-percentage point lower 12 month progression event rate among patients who were ctDNA-negative compared to those who were ctDNA-positive, we estimated that we needed 65 evaluable patients based on time to event assumption and log-rank based methods. We assumed 20% of patients would be ctDNA-positive at cycles 3 or 4, and among those who were ctDNA-positive, we estimated a 50% progression rate with docetaxel and a 60% progression rate in patients treated with an ARPI. At the outset (protocol version 1.0), PFS was the primary endpoint with a plan to report OS after sufficient deaths at a later analysis. Owing to additional funding for longer follow-up, the protocol was amended in May 2022 (changes listed on page 49, protocol v.6.0) to include both OS and PFS as major endpoints, while retaining the same sample size, and report both OS and PFS concurrently when ~30% deaths had occurred.

Comparisons of OS and PFS between patients who were ctDNA-positive and ctDNA-negative were performed using multivariable Cox regression. The proportional hazards assumption was evaluated before conducting the analysis for the following analyses: PFS in PARADIGM-A and PFS in PARADIGM-D. This assumption was not assessed for the remaining analyses. HRs were reported after adjustment for age at registration, time on ADT before cycle 1, PSA before start of ADT (all continuous variables), Eastern Cooperative Oncology Group performance status (0 vs 1 or 2) and, if applicable, study cohort (docetaxel or ARPI). Landmark analyses included all patients who were event-free and not censored at cycle 4 and had ctDNA measured at cycle 3 or cycle 4. Survival time was measured from day 1 cycle 4, and models were adjusted for the time from start of treatment to day 1 cycle 4 to reduce potential confounding. HRs for PARADIGM-D alone were reported unadjusted, owing to the smaller size of this cohort. On-treatment PSA (alone and in combination with ctDNA) was tested both as a continuous variable and by categories previously reported to be prognostic at 7 months after ADT initiation^10^. Fisher’s exact test was used for comparisons of categorical baseline factors. The Wilcoxon rank-sum test was used to compare baseline continuous variables between ctDNA groups. As this method does not assume normality, no formal test of normality was undertaken. McNemar’s paired test was applied to compare ctDNA detection across samples from individual patients in the pre-ADT sub-study. We fitted a Cox proportional hazards model with ctDNA modeled as a time-dependent covariate to account for changes in ctDNA status over time. The follow-up of each patient was divided into intervals between consecutive assessments. For each interval, ctDNA status was assumed to remain constant until the next assessment, although ctDNA status may have changed over time from assessment to assessment.

Exploratory testing of ctDNA and PSA characteristics was conducted using Cox proportional hazards models fitted using ctDNA, serum PSA and baseline clinical covariates. For each model, the linear predictor was derived and used to evaluate discrimination for 12 and 24 month survival using ROC analysis. Timepoints were chosen to have no censoring. Two model specifications were compared: ctDNA plus serum PSA plus baseline clinical covariates, and serum PSA and the same covariates (without ctDNA). Analyses were performed using biomarker values from cycle 3 or 4 and were separately repeated for cycle 1. The likelihood ratio test was applied to assess whether inclusion of ctDNA significantly improved the model fit.

### Data handling and management

All data within the PARADIGM study were collected prospectively and managed centrally by the UCL Cancer Clinical Trials Centre using Case Report Tracker (v.4.0.0.0), Macro EDC (v.4.9.1.8852) and Macro Paradigm Database (version PARADIGM-20240520-0170).

### Reporting summary

Further information on research design is available in the Nature Portfolio Reporting Summary linked to this article.

## Supplementary information

Supplementary Information Supplementary Tables 1–12, Original PARADIGM Protocol and Statistical Analysis Plan.

Reporting Summary

Peer Review File

Supplementary Code Readme file for the computational pipeline for allele-informed copy number analysis of plasma DNA samples.

## Source data

Source Data Fig. 2 Statistical source data.

Source Data Fig. 3 Statistical source data.

Source Data Fig. 4 Statistical source data.

Source Data Extended Data Fig. 2 Statistical source data.

## Data Availability

The original files and raw next-generation sequencing data generated in this study have been deposited in the European Genome-Phenome Archive (EGA) and can be downloaded, under controlled access, from the EGA web portal (https://ega-archive.org) with study number EGAS50000001357. Additional clinical information can be made available upon institutional approval. Requests should be sent to g.attard@ucl.ac.uk. The estimated timeframe for access to be granted is 2 months, and the duration will be determined according to the requested needs. All relevant clinical trial data used in this study are accessible in the Source Data. All patient data are deidentified. Source data are provided with this paper.

## Code availability

The code for CLONETv2 has been published previously and is available for download as a singularity package at the PCF_SELECT GitHub repository (https://github.com/demichelislab/PCF_SELECT).
