# Supplementary material for: Combined ctDNA and serum PSA for dynamic monitoring of metastatic prostate cancer starting first-line treatment: a prospective national cohort study
Source: Nat Cancer. 2026 May 15;7(6):915–27. doi: 10.1038/s43018-026-01172-9 (PMC13309274; doi:10.1038/s43018-026-01172-9)
Supplement: Supplementary file 1 — Supplementary Tables 1–12, Original PARADIGM Protocol and Statistical Analysis Plan. [file 43018_2026_1172_MOESM1_ESM.pdf]

nature cancer

Supplementary information https://doi.org/10.1038/s43018-026-01172-9

Combined ctDNA and serum PSA for dynamic monitoring of metastatic prostate cancer starting first-line treatment: a prospective national cohort study

In the format provided by the authors and unedited

Table of Contents

Table S1. PARADIGM study recruitment by clinical site........ **3**

Table S2. Baseline characteristics of patients included in the primary analysis, by treatment........ 4

Table S3. Androgen receptor pathway inhibitors used in PARADIGM-A. **5**

Table S4. PSA subgroups by treatment cohort........ 5

Table S5. Median progression free and overall survival by cohort........ **5**

Table S6. Overall and progression-free survival rates based on ctDNA. **6**

Table S7. Univariable and multivariable analysis for progression free survival in PARADIGM-A........ 6

Table S8. Progression free survival analysis from start of treatment and landmark analysis. **7**

Table S9. Univariable and multivariable analysis for overall survival. **8**

Table S10. Overall survival analysis from start of treatment and landmark analysis........ **9**

Table S11. Changes in circulating tumor DNA and overall survival. **10**

Table S12. Overall survival by ctDNA within PSA prognostic groups........ **11**

REMARK Checklist **12**

PARADIGM Protocol and Statistical Analysis Plan

Table S1. PARADIGM study recruitment by clinical site.

Clinical site	Number of patients recruited
University College London Hospital	24
Kingston Hospital	22
Royal Marsden Hospital	21
The Christie Hospital	9
St Bartholomew's Hospital	8
Guy’s and St Thomas Hospitals	7
Doncaster Royal Infirmary	7
Beatson Cancer Centre	7
Royal Free Hospital	4
Medway Maritime Hospital	4
Bournemouth Hospital	1
Southampton Hospital	1
Velindre Cancer Centre	1
Aberdeen Royal Infirmary	1

Table S2. Baseline characteristics of patients included in the primary analysis, by treatment.

Baseline characteristic, N (%) *	Overall cohort	PARADIGM-D	PARADIGM-A	p-value
	104	31 (30%)	73 (70%)	
**Age, in years**				
Median (range)	68 (48 to 90)	63 (48 to 75)	71 (49 to 90)	0.001
**Ethnicity**				
Asian	4 (4%)	2 (7%)	2 (3%)	0.03
Black	8 (8%)	1 (3%)	7 (10%)	
White	87 (84%)	24 (77%)	63 (86%)	
Not recorded	5 (5%)	4 (13%)	1 (1%)	
**Eastern Cooperative Oncology Group performance status**				
0	54 (52%)	21 (68%)	33 (45%)	0.03
1- 2	50 (48%)	10 (32%)	40 (55%)	
**PSA prior to start of ADT[¥] (ng/mL)**				
Median (range)	171(2.5 to 5000.0)	159 (2.5 to 2500.0)	216 (6.4 to 5000.0)	0.34
**Gleason score**				
≤7	14 (14%)	2 (7%)	12 (16%)	0.10
≥8	75 (72 %)	27 (87%)	48 (66%)	
Not evaluable[†]	15 (14%)	2 (7%)	13 (18%)	
**Presentation of metastases relative to diagnosis**				
Metachronous	8 (8%)	2 (7%)	6 (8%)	0.56
Synchronous	96 (92%)	29 (94%)	67 (92%)	
**Type of ADT**				
LHRH agonist	64 (62%)	19 (61%)	45 (62%)	0.99
LHRH antagonist	40 (38%)	12 (39%)	28 (38%)	
**Time on ADT[¥] before day 1 cycle 1 (days)**				
Median (range)	57 (12 to 108)	68 (22 to 108)	51 (12 to 106)	0.004
**Visceral metastases**				
No	84 (81%)	21 (68%)	63 (86%)	0.03
Yes[‡]	20 (19%)	10 (32%)	10 (14%)	

[*] At study registration.

p-values stated are for the comparison between treatment cohorts.

† Patients with a biopsy from any metastatic site (N, 6) or radiological or biochemical diagnosis (N, 9).

‡ With or without bone metastases.

¥Androgen deprivation therapy (ADT) in the form of luteinizing hormone releasing hormone (LHRH) antagonist or agonist.

N, number; PSA, prostate specific antigen.

Table S3. Androgen receptor pathway inhibitors used in PARADIGM-A.

Androgen receptor pathway inhibitor	Number of patients (%)
Abiraterone acetate and prednisolone	18 (25%)
Apalutamide	2 (3%)
Enzalutamide	53 (72%)
**Total**	**73**

Table S4. PSA subgroups by treatment cohort.

PSA at cycles 3 or 4 (ng/mL)	PARADIGM-D	PARADIGM-A
≤0.2	3 (10%)	19 (26%)
0.2 to 4	17 (55%)	37 (51%)
>4	11/ (35%)	17 (23%)

Table S5. Median progression free and overall survival by cohort.

Time to event	PARADIGM-D	PARADIGM-A
Patients (N)	31	73
Median PFS in months (95% CI)	11.66 (8.90 to 13.37)	32.62 (21.68 to NR)
Median OS in months (95% CI)	42.87 (23.95 to NR)	49.02 (35.55 to NR)

CI, confidence interval; NR, not yet reached.

Table S6. Overall and progression-free survival rates based on ctDNA.

ctDNA status*	Patients alive (%, 95% CI)			
	12 months	18 months	24 months	36 months
**Negative, N, 74**	99 (91 to 100)	93 (85 to 97)	85 (75 to 91)	68 (56 to 78)
**Positive, N, 30**	73 (54 to 86)	67 (47 to 80)	50 (31 to 66)	39 (22 to 56)
	**Patients alive and without progression (%, 95% CI), PARADIGM-A**			
**Negative, N, 53**	81 (68 to 89)	72 (58 to 82)	60 (46 to 72)	48 (34 to 61)
**Positive, N, 20**	70 (45 to 85)	60 (36 to 78)	50 (27 to 69)	39 (19 to 60)
	**Patients alive and without progression (%, 95% CI), PARADIGM-D**			
**Negative, N, 21**	62 (38 to 79)	43 (22 to 62)	43 (22 to 62)	29 (12 to 48)
**Positive, N, 10**	10 (1 to 36)	10 (1 to 36)	10 (1 to 36)	10 (1 to 36)

** at cycles 3 or 4; ctDNA, circulating tumor DNA; N, number; CI, confidence interval.*

Table S7. Univariable and multivariable analysis for progression free survival in PARADIGM-A.

Variables	Univariable Cox Model			Multivariable Cox Model		
	HR	95% CI	p value	HR	95% CI	p-value
ctDNA pos vs neg*	1.34	0.68, 2.64	0.394	1.46	0.73, 2.90	0.286
ECOG 1-2 vs 0	1.26	0.67, 2.36	0.471	1.88	0.94, 3.74	0.074
Age at baseline	0.97	0.93, 1.00	0.084	0.95	0.92, 0.99	0.021
Time on ADT before day 1 cycle 1	1.00	0.99, 1.01	0.842	1.00	0.98, 1.01	0.526
PSA prior to start of ADT	1.00	1.00, 1.00	0.608	1.00	1.00,1.00	0.533

** at cycles 3 or 4; ctDNA, circulating tumor DNA; ECOG, Eastern Co-operative Oncology Group performance status; ADT, androgen deprivation therapy.*

Table S8. Progression free survival analysis from start of treatment and landmark analysis.

ctDNA status	Time-point	Median PFS (95% CI)	Unadjusted			Adjusted*		
			HR	95% CI	p value	HR	95% CI	p-value
**PARADIGM-A**								
ctDNA pos vs neg at cycles 3 or 4	From start of treatment	20.96 (9.99, NR) vs 34.96 (22.93, NR)	1.34	0.68, 2.64	0.394	1.46	0.73, 2.90	0.286
	From cycle 4 day 1**	21.72 (10.32 to NR) vs 31.90 (20.21 to NR)	1.15	0.56, 2.37	0.705	1.20	0.57, 2.51	0.634
**PARADIGM-D**								
ctDNA pos vs neg at cycles 3 or 4	From start of treatment	7.33 (2.14, 8.90) vs 12.65 (10.97, 34.30)	3.87	1.60, 9.34	0.003	5.01	1.65, 15.19	0.004
	From cycle 4 day 1**	6.05 (0.13, 6.83) vs 10.58 (8.67, 32.00)	3.57	1.43, 8.90	0.006	4.31	1.39, 13.33	0.011

* Adjusted for age at registration, time on ADT prior to cycle 1, PSA prior to ADT, Eastern Cooperative Oncology Group performance status and study cohort (ARPI or docetaxel).

** Adjusted for time from start of treatment to cycle 4 day 1 date. For three patients without a recorded Cycle 4 date, the Cycle 4 date was estimated by adding the protocol-specified interval to their Cycle 3 date. Three patients had a PFS event between cycle 3 and cycle 4 and were excluded from the overall PFS analysis.

ctDNA, circulating tumor DNA; pos, positive; neg, negative; vs, versus; HR, hazard ratio CI, confidence interval.

Table S9. Univariable and multivariable analysis for overall survival.

Variables	Univariable Cox Model			Multivariable Cox Model		
	HR	95% CI	p-value	HR	95% CI	p-value
ctDNA pos vs neg*	2.72	1.49, 4.95	0.001	3.07	1.64, 5.74	<0.001
PARADIGM-D vs PARADIGM-A	1.41	0.76, 2.62	0.277	1.60	0.78, 3.25	0.197
ECOG 1-2 vs 0	1.29	0.71, 2.33	0.402	1.68	0.89, 3.15	0.108
Age at baseline	0.98	0.94, 1.01	0.190	0.98	0.94, 1.02	0.289
Time on ADT before day 1 cycle 1	1.00	0.99, 1.01	0.946	1.00	0.99, 1.02	0.907
PSA prior to start of ADT	1.00	1.00, 1.00	0.891	1.00	1.00,1.00	0.789

**at cycle 3 or 4; ctDNA, circulating tumor DNA; ECOG, Eastern Co-operative Oncology Group performance status; ADT, androgen deprivation therapy.*

Table S10. Overall survival analysis from start of treatment and landmark analysis.

ctDNA status	Time-point	Median OS (95% CI)	Unadjusted			Adjusted*		
			HR	95% CI	p value	HR	95% CI	p-value
ctDNA pos vs neg at cycles 3 or 4	From start of treatment	23.95 (14.59, NR) vs NR (42.9, NR)	2.72	1.49, 4.95	0.001	3.07	1.64, 5.74	<0.001
	From cycle 4 day 1**	21.39 (11.56 to NR) vs NR (40.80 to NR)	2.89	1.58, 5.31	0.001	3.01	1.61, 5.64	0.001

* Adjusted for age at registration, time on ADT prior to cycle 1, PSA prior to ADT, Eastern Cooperative Oncology Group performance status and study cohort (ARPI or docetaxel).

** Adjusted for time from start of treatment to cycle 4 day 1 date. All 104 patients were included in the analysis as no patients had an OS event between cycle 3 and 4. For the three patients without a recorded Cycle 4 date, the Cycle 4 date was estimated by adding the protocol-specified interval to their Cycle 3 date.

ctDNA, circulating tumor DNA; pos, positive; neg, negative; vs, versus; HR, hazard ratio CI, confidence interval.

Table S11. Changes in circulating tumor DNA and overall survival.

ctDNA at cycle 1, cycles 3/4	Patients N	Events N (%)	Median overall survival, months (95% CI)	Unadjusted Cox regression	Adjusted HR†	
				HR (95% CI)	HR (95% CI)	p-value
negative, negative	56	18 (32%)	NR (45.7 to NR)	1 (reference)	1 (reference)	-
negative, positive	19	9 (47%)	49.0 (24.0 to NR)	1.57 (0.70 to 3.50)	1.85 (0.81 to 4.20)	0.15
positive, negative	16	6 (38%)	NR (30.0 to NR)	1.15 (0.46 to 2.90)	1.17 (0.45 to 3.01)	0.75
positive, positive	11	10 (91%)	8.5 (3.7 to 13.1)	13.20 (5.85 to 29.82)	17.00 (6.64 to 43.47)	<0.001

†Adjusted for age at registration, time on ADT prior to cycle 1, PSA prior to ADT, Eastern Cooperative Oncology Group performance status and study cohort (ARPI or docetaxel).N, number; CI, confidence interval; NR, not yet reached; HR, hazard ratio.

Table S12. Overall survival by ctDNA within PSA prognostic groups.

ctDNA status**	PSA (ng/mL)**	Patients, N	Events (%)	Median OS (95% CI)	Adjusted*		
					HR	95% CI	p-value
Positive vs negative	≤0.2	7 vs 15	1 (14%) vs 2 (13%)	NR (24.0, NR) vs NR (NR, NR)	0.44	NR, NR	NR
	>0.2 to 4	9 vs 45	7 (78%) vs 14 (31%)	33.3 (3.7, NR) vs NR (NR, NR)	2.93	1.16, 7.40	0.023
	>4	14 vs 14	11 (79%) vs 9 (64%)	13.1 (6.4, 24.2) vs 35.8 (25.2 vs NR)	8.08	2.06, 31.70	0.003

**Adjusted for age at registration, time on ADT prior to cycle 1, PSA prior to ADT, Eastern Co-operative Oncology Group performance status and study cohort (ARPI or docetaxel); **at cycle 3 or 4.*

PSA, prostate specific antigen; ctDNA, circulating tumor DNA; N, number; NR, not yet reached; HR, hazard ratio; CI, confidence interval.

REMARK Checklist

Item to be reported		Page no.
**INTRODUCTION**		
1	State the marker examined, the study objectives, and any pre-specified hypotheses.	4,5
**MATERIALS AND METHODS**		
Patients		
2	Describe the characteristics (e.g., disease stage or co-morbidities) of the study patients, including their source and inclusion and exclusion criteria.	15,16
3	Describe treatments received and how chosen (e.g., randomized or rule-based).	16
Specimen characteristics		
4	Describe type of biological material used (including control samples) and methods of preservation and storage.	16,17
Assay methods		
5	Specify the assay method used and provide (or reference) a detailed protocol, including specific reagents or kits used, quality control procedures, reproducibility assessments, quantitation methods, and scoring and reporting protocols. Specify whether and how assays were performed blinded to the study endpoint.	17,18
Study design		
6	State the method of case selection, including whether prospective or retrospective and whether stratification or matching (e.g., by stage of disease or age) was used. Specify the time period from which cases were taken, the end of the follow-up period, and the median follow-up time.	
7	Precisely define all clinical endpoints examined.	
8	List all candidate variables initially examined or considered for inclusion in models.	19

9	Give rationale for sample size; if the study was designed to detect a specified effect size, give the target power and effect size.	19
Statistical analysis methods		
10	Specify all statistical methods, including details of any variable selection procedures and other model-building issues, how model assumptions were verified, and how missing data were handled.	19
11	Clarify how marker values were handled in the analyses; if relevant, describe methods used for cutpoint determination.	
**RESULTS**		
Data		
12	Describe the flow of patients through the study, including the number of patients included in each stage of the analysis (a diagram may be helpful) and reasons for dropout. Specifically, both overall and for each subgroup extensively examined report the numbers of patients and the number of events.	6
13	Report distributions of basic demographic characteristics (at least age and sex), standard (disease-specific) prognostic variables, and tumor marker, including numbers of missing values.	6
Analysis and presentation		
14	Show the relation of the marker to standard prognostic variables.	7,8,9, 10,11
15	Present univariable analyses showing the relation between the marker and outcome, with the estimated effect (e.g., hazard ratio and survival probability). Preferably provide similar analyses for all other variables being analyzed. For the effect of a tumor marker on a time-to-event outcome, a Kaplan-Meier plot is recommended.	7,8,9, 10,11

16	For key multivariable analyses, report estimated effects (e.g., hazard ratio) with confidence intervals for the marker and, at least for the final model, all other variables in the model.	7,8,9, 10,11
17	Among reported results, provide estimated effects with confidence intervals from an analysis in which the marker and standard prognostic variables are included, regardless of their statistical significance.	7,8,9, 10,11
18	If done, report results of further investigations, such as checking assumptions, sensitivity analyses, and internal validation.	7,8,9, 10,11
**DISCUSSION**		
19	Interpret the results in the context of the pre-specified hypotheses and other relevant studies; include a discussion of limitations of the study.	11, 12,13 ,14
20	Discuss implications for future research and clinical value.	

PARADIGM

Plasma Analysis for Response Assessment and to DIrect the manaGement of Metastatic prostate cancer

Study Sponsor: University College London (UCL)
Study Sponsor reference: UCL/18/0513
Study funder: Prostate Cancer UK
Funder reference: MA-TR15-007
Clinicaltrials.gov no:

Protocol version no: 1.0
Protocol version date: 14/01/2019

COORDINATING CENTRE:

For general queries, supply of study documentation and central data management please contact:

PARADIGM Trial Coordinator
Cancer Research UK & UCL Cancer Trials Centre
90 Tottenham Court Road
London
W1T 4TJ
United Kingdom

Tel: +44 (0) 20 7679 9351
Fax: +44 (0) 20 7679 9871
09:00 to 17:00 Monday to Friday, excluding Bank Holidays

Email: ctc.paradigm@ucl.ac.uk

Other Study contacts:

Chief Investigator:	Professor Gerhardt Attard
Address:	UCL Cancer Institute<br>Paul O'Gorman Building<br>University College London<br>72 Huntley Street<br>London<br>WC1E 6BT
Trial statistician:	Graham Wheeler
Address:	Cancer Research UK & UCL Cancer Trials Centre<br>90 Tottenham Court Road<br>London<br>W1T 4TJ<br>United Kingdom

Protocol 1.0, 14th January 2019, Authorisation signatures:

Name & Role:	Signature:	Date authorised:
Chief Investigator:<br>Professor Gerhardt Attard<br>Consultant Medical Oncologist, UCL		5th Jun 2019
For the Sponsor:<br>Professor Jonathan Ledermann<br>Director, UCL CTC		20 May 2019
Laura White<br>Trials Group Lead, UCL CTC		23 May 2019

Please note: This study protocol must not be applied to patients treated outside the PARADIGM study. Cancer Research UK & UCL Cancer Trials Centre (UCL CTC) can only ensure that approved trial investigators are provided with amendments to the protocol.

Patrick Magill and Joseph Hanlon are the patient representatives on the Trial Management Group (TMG) and have contributed to the development of this study and have reviewed the patient information documentation.

Trial Management Group (TMG):

Name	Position	Institution
Gerhardt Attard	Consultant Medical Oncologist	UCL Cancer Institute
Anuradha Jayaram	Clinical Research Fellow	UCL Cancer Institute
Blanca Trujillo Alba	Clinical Research Fellow	UCL Cancer Institute
Laura White	Trials Group Lead	UCL CTC
Marian Duggan	Senior Trial Co-ordinator	UCL CTC
Meena Reddi	Trial Co-ordinator	UCL CTC
Graham Wheeler	Trial Statistician	UCL CTC
Simon Crabb	Consultant Medical Oncologist	Southampton General Hospital
Rob Jones	Consultant Medical Oncologist	Beatson Cancer Centre
Alison Birtle	Consultant Medical Oncologist	Royal Lancashire
Alison Reid	Consultant Medical Oncologist	Royal Marsden and Kingston Hospital
Elias Pintus	Consultant Medical Oncologist	Guy's Hospital
Ursula McGovern	Consultant Medical Oncologist	High Barnet Hospital
Costi Alifrangis	Consultant Medical Oncologist	Bart's and the London Hospital
Silke Gillessen	Consultant Medical Oncologist	Christie Hospital
Prasanna Sooriakumaran	Consultant Urological Surgeon	UCL Hospital
Patrick Magill	Patient representative	
Joe Hanlon	Patient representative	

Translational research sub-committee

Name	Institution	Responsibilities
Gerhardt Attard	UCL Cancer Institute	Chair
Anuradha Jayaram	UCL Cancer Institute	Immunoprofiling and plasma DNA analysis
Daniel Wetterskog	UCL Cancer Institute	NGS assays
Blanca Trujillo Alba	UCL Cancer Institute	Plasma DNA analysis
Anna Wingate	UCL Cancer Institute	Logistics
Ryan Dittamore	Epic Sciences	CTC analysis
Mark Linch	UCLH	Immune therapies
Francesca Demichelis	University of Trento, Italy	Computational bioinformatics
Shonit Punwani	UCLH	Imaging
Harbir Sidhu	UCLH	Imaging
Prabhakar Rajan	QMUL	Exosomes

TABLE OF CONTENTS

1 PROTOCOL SUMARY 7

1.1 SUMMARY OF STUDY DESIGN 7
1.2 GRAPHICAL SUMMARY 12
1.3 FUNDING 12

2 INTRODUCTION 13

2.1 MANAGEMENT OF DE NOVO METASTATIC PROSTATE CANCER 13
2.2 PLASMA DNA ANALYSIS 14
2.3 NOMENCLATURE AND CATEGORISATION RELATED TO DISEASE METASTATIC STATUS 16
2.4 JUSTIFICATION FOR CONDUCTING PARADIGM 17
2.5 IMMUNE PROFILING IN METASTATIC PROSTATE CANCER 17
2.6 CIRCULATING TUMOUR CELLS IN METASTATIC PROSTATE CANCER 18
2.7 WHOLE BODY DIFFUSION-WEIGHTED MRI FOR HORMONE SENSITIVE METASTATIC PROSTATE CANCER 19
2.8 FUTURE IMPLICATIONS OF PARADIGM 20

3 STUDY DESIGN 21

3.1 STUDY OBJECTIVES 21
3.1.1 Primary Objective: 21
3.1.2 Secondary Objectives: 21
3.1.3 Exploratory objective 21
3.1.4 Translational objectives 21
3.2 STUDY ENDPOINTS 22
3.2.1 Primary end point 22
3.2.2 Secondary End Point 22
3.3 STUDY ACTIVATION 22

4 SELECTION OF SITES/SITE INVESTIGATORS 24

4.1 SITE SELECTION 24
4.2 SELECTION OF PRINCIPAL INVESTIGATOR AND OTHER INVESTIGATORS AT SITES 24
4.3 TRAINING REQUIREMENTS FOR SITE STAFF 24
4.4 SITE INITIATION AND ACTIVATION 24
4.4.1 Site initiation 24
4.4.2 Required documentation 24
4.4.3 Site activation letter 25

5 SELECTION OF PATIENTS 26

5.1 SCREENING LOG 26
5.2 PATIENT ELIGIBILITY 26
5.2.1 Inclusion criteria 26
5.2.2 Exclusion criteria 26

6 INFORMED CONSENT 28

6.1 CONSENT TO PRE-STUDY (ADT SAMPLE) 28
6.2 CONSENT TO MAIN STUDY 29

6.2.1 *Consent to patient directed sample collection* 29
6.2.2 *Consent for feedback of clinically-relevant genetic information* 29
6.3 CONSENT TO WHOLE BODY MAGNETIC RESONANCE IMAGING (WBMRI) 29
(AT SELECTED CENTRES ONLY) 29

7 REGISTRATION PROCEDURES 30

7.1 REGISTRATION TO PRE-STUDY (ADT SAMPLE) 30
7.2 REGISTRATION TO MAIN STUDY 30

8 STUDY INTERVENTION 31

8.1 TREATMENT SUMMARY 31
8.2 STANDARD OF CARE ADT 31
8.2.1 *Standard of care docetaxel* 31
8.2.2 *Standard of care abiraterone* 31
8.3 PREDNISOLONE SWITCH TO DEXAMETHASONE 31
8.4 RESEARCH BLOOD SAMPLE COLLECTION 31
8.4.1 *Patient-directed sample collection* 32
8.4.2 *Site directed sample collection* 32
8.5 ARCHIVAL DIAGNOSTIC BLOCK COLLECTION 32
8.6 RESEARCH BLOOD SAMPLE PROCESSING 32
8.6.1 *Collection of ptDNA sample* 33
8.6.2 *Collection of CTC samples* 33
8.6.3 *Collection of Immunoprofiling samples* 33

9 WITHDRAWAL OF PATIENTS 34

9.1 FUTURE DATA COLLECTION 34
9.2 LOSSES TO FOLLOW-UP 34
9.3 LOSS OF CAPACITY 34

10 ASSESSMENTS & DATA COLLECTION 35

10.1 PRE-REGISTRATION 35
10.2 ASSESSMENTS PRIOR TO STARTING ABIRATERONE/DOCETAXEL 35
10.3 ASSESSMENTS DURING FIRST SIX CYCLES OF TREATMENT 36
10.4 ASSESSMENTS AFTER FIRST SIX CYCLES OF TREATMENT AND FOLLOW-UP 37
10.5 RECORDING DISEASE PROGRESSION 37
10.6 ASSESSMENTS AFTER DISEASE PROGRESSION 38

11 TRANSLATIONAL RESEARCH 39

11.1 PREDICTORS OF RESPONSE TO SYSTEMIC TREATMENT 39
11.2 TRACKING OF PLASMA DNA DYNAMICS 39
11.3 CTC DYNAMICS AT INITIATION OF ADT 40
11.4 INTERROGATION OF PERIPHERAL IMMUNE CHANGES SECONDARY TO INITIATION OF ADT 40
11.5 WHOLE BODY MRI DERIVED IMAGING BIOMARKERS AS A SURROGATE OF RESPONSE (SELECTED SITES ONLY) 41

12 STATISTICS 42

12.1 CALCULATION OF TARGET NUMBER OF PATIENTS 42
12.2 DEFINITION OF CRITERIA REQUIRED FOR A PATIENT TO BE INCLUDED IN THE PRIMARY ENDPOINT ANALYSIS 42
12.3 PRIMARY ENDPOINT: ANALYSIS AND DETAILED DEFINITION 43
12.3.1 *Symptomatic or asymptomatic new or unequivocal progression of prior distant metastases confirmed by imaging* 43
12.3.2 *Symptomatic progression of cancer in the prostate confirmed by imaging* 43

12.3.3 Serum PSA progression 43
12.3.4 Prostate cancer specific death 44
12.4 SECONDARY ENDPOINTS: DETAILED DEFINITION AND ANALYSES 44
12.5 EXPLORATORY ENDPOINT 45
12.6 STATISTICAL PLAN FOR TRANSLATIONAL RESEARCH 45
12.6.1 Predictors of response to systemic treatment 45
12.6.2 Tracking of plasma DNA dynamics 45
12.6.3 CTC dynamics at initiation of ADT 46
12.6.4 Interrogation of peripheral immune changes secondary to initiation of ADT 46
12.6.5 Novel imaging as a surrogate of response 46

13 DATA MANAGEMENT AND DATA HANDLING GUIDELINES 47

13.1 ENTERING DATA INTO THE ECRF 47
13.2 CORRECTIONS TO ECRF FORMS 47
13.3 MISSING DATA 47
13.4 TIMELINES FOR DATA ENTRY 47
13.5 DATA QUERIES 47

14 SAFETY REPORTING 48

14.1 DEFINITIONS 48
14.1.1 Adverse Reactions (AR) 48
14.1.2 Related & Unexpected SARs 48
14.1.3 Serious Adverse Reactions (SAR) 48
14.2 REPORTING OF SERIOUS ADVERSE REACTIONS (SARS) 48
RELATED AND UNEXPECTED SERIOUS ADVERSE REACTION 49

15 INCIDENT REPORTING AND SERIOUS BREACHES 50

15.1 INCIDENT REPORTING 50
15.2 SERIOUS BREACHES 50

16 STUDY MONITORING AND OVERSIGHT 51

16.1 CENTRAL MONITORING 51
16.2 'FOR CAUSE' ON-SITE MONITORING 51
16.3 OVERSIGHT COMMITTEES 51
16.3.1 Trial Management Group (TMG) 51
16.3.2 Translational Research Committee 52
16.3.3 Trial Steering Committee (TSC) 52
16.3.4 Role of UCL CTC 52

17 STUDY CLOSURE 53

17.1 END OF TRIAL 53
17.2 ARCHIVING OF TRIAL DOCUMENTATION 53
17.3 EARLY DISCONTINUATION OF TRIAL 53
17.4 WITHDRAWAL FROM TRIAL PARTICIPATION BY A SITE 53

18 ETHICAL CONSIDERATIONS 54

18.1 ETHICAL APPROVAL 54
18.2 SITE APPROVALS 54
18.3 PROTOCOL AMENDMENTS 54
18.4 PATIENT CONFIDENTIALITY & DATA PROTECTION 54

19 SPONSORSHIP AND INDEMNITY 55

19.1 SPONSOR DETAILS 55
19.2 INDEMNITY 55

20 PUBLICATION POLICY 56

21 REFERENCES 57

APPENDIX 1: ABBREVIATIONS 61

APPENDIX 2: SCHEDULE OF ASSESSMENTS 64

APPENDIX 3: PROTOCOL VERSION HISTORY 65

1 PROTOCOL SUMMARY

1.1 Summary of Study Design

Title:	**P**lasma **A**nalysis for **R**esponse **A**ssessment and to **DI**rect the mana**G**ement of **M**etastatic prostate cancer
Short Title/acronym:	PARADIGM
Sponsor name & reference:	University College London, UCL/18/0513
Funders & reference:	Prostate Cancer UK is the main funder of the study; MA-TR15-007<br><br>Additional funding:<br>Cancer Research UK, C35118/A22744; C65130/A26321,<br>Medical Research Council, MR/P002072/1<br>Epic Sciences
Clinicaltrials.gov no:	Pending
Design:	A prospective, observational, biomarker-focused, translational platform, cohort study in newly diagnosed polymetastatic prostate cancer patients starting long-term systemic therapy.
Target accrual:	~170 men to have 130 men evaluable for the primary endpoint.
Inclusion criteria:	1. Able and willing to provide written informed consent<br>2. Prostate adenocarcinoma confirmed on biopsy obtained in previous 6 months<br>3. Polymetastatic disease defined as two of the following:<br>i. Gleason score of ≥ 8,<br>ii. Presence of ≥3 lesions on bone scan,<br>iii. Presence of measurable visceral lesion<br>4. Eastern Cooperative Oncology Group (ECOG) Performance status 0 to 2<br>5. No medical contra-indications to abiraterone or docetaxel<br>6. Patients should be either of the following:<br>i. Planned to start long-term Luteinizing hormone (LH) suppression, or<br>ii. within 10 weeks of starting long-term LHRH antagonist, or<br>iii. within 12 weeks of starting LHRH agonist or an anti-androgen when the latter is used in combination with or prior to LHRH agonist for flare protection.<br>7. Patients should be planned for addition of docetaxel (PARADIGM-D) or abiraterone (PARADIGM-A) 5 to 10 weeks after start of LHRHa (or 7 to 12 weeks if LHRH agonist is started without anti-androgen) with a target of 6 cycles or continuation until progression respectively.<br>8. No concomitant medical conditions likely to reduce life expectancy.<br>9. Patient agrees to be followed up in the recruiting centre and to having sequential plasma samples collected as per the study protocol.

Exclusion criteria:	1. Medically unsuitable for either abiraterone, prednisolone or docetaxel.<br>2. Concurrent or planned for (within the first 5 cycles of docetaxel or abiraterone) treatment with any experimental drugs, oestrogen patches, radiotherapy or surgery to the primary tumour. Patients randomised to the standard of care (SOC) arm in open-label clinical trials are eligible. Patients who are still to be randomised to STAMPEDE may be included where the randomisation will be limited to SOC or arm K. Patients can participate in other observational studies.<br>3. Prior systemic therapy for prostate cancer other than for LHRHa +/- anti-androgen (started within the time limits defined in inclusion criterion 6).<br>4. Metastatic brain disease or leptomeningeal disease.<br>5. Any surgery planned prior to Cycle 3 Day 1 (C3 D1)<br>6. Other current malignancy or malignancy diagnosed or relapsed within the past 5 years (other than non-melanomatous skin cancer, stage 0 melanoma in situ and non-muscle invasive bladder cancer).<br>7. Patients who consent to the whole-body magnetic resonance imaging (WBMRI) translational sub-study should have no contraindications to MRI as per local guidelines.
Primary objective:	To determine whether the detection of plasma tumour DNA (ptDNA) after two cycles of abiraterone (with prednisone) or docetaxel (with or without prednisone) added after start of ADT is associated with a worse clinical outcome in newly diagnosed metastatic prostate cancer.
Secondary objectives:	1. To compare ptDNA classification at C2D1 and C5D1 with C3D1.<br>2. To determine whether the detection of ptDNA after four to twelve weeks of starting ADT and prior to starting abiraterone or docetaxel associates with a worse clinical outcome.<br>3. To determine the association between clinical outcome and prostate specific antigen (PSA) level (<0.2, 0.2-4, >4ng/dl) after four to twelve weeks of starting ADT and prior to starting abiraterone or docetaxel and at C2D1, C3D1, C5D1 (for both abiraterone and docetaxel) and at 7 months after start of ADT.<br>4. To assess whether ptDNA detection is a better predictor of clinical outcome than PSA level (as assessed in objective 3) after four to twelve weeks of starting ADT and prior to starting abiraterone or docetaxel and at C2D1, C3D1 and C5D1.<br>5. To compare associations with clinical outcome for the change in ptDNA detection and PSA level (as assessed in objective 3) prior to start of abiraterone or docetaxel and at C3D1.<br>6. To evaluate whether ptDNA fraction prior to LHRHa (stratified by no anti-androgen versus 2-3 weeks anti-androgen) associates with Progression Free Survival (PFS) and Overall Survival (OS).

Exploratory objectives	1. To develop and test a composite model incorporating different biomarkers for assessing response.<br>2. To describe ptDNA dynamics and compare to PSA kinetics.
Translational Research	Translational research will be performed on sub-sets of patients from whom the required evaluations are made. It is expected that not all patients will be able to participate in all the translational studies but as a fundamental aspect of PARADIGM, as many patients as possible should be included in these assessments.<br><br>1. Predictors of response to systemic treatment<br>• To identify molecular signatures in plasma and tumour that associate with PFS or OS with abiraterone or docetaxel.<br>• To identify a molecular signature in pre-ADT plasma or tumour that associates with plasma androgen receptor (AR) aberrant status at progression to castration-resistant disease.<br><br>2. Tracking of plasma DNA dynamics<br>• To determine whether detection of ptDNA precedes clinical, biochemical or radiological progression.<br>• To characterise resistant clones<br>• To evaluate whether patients who progress with AR gain at the development of metastatic castration resistant prostate cancer (mCRPC) have a shorter time to PFS (on ADT) and OS.<br><br>3. Circulating tumour cell (CTC) dynamics at initiation of ADT<br>• To evaluate whether CTC count pre-ADT and after starting ADT associates with shorter PFS and OS.<br>• To identify CTC molecular features prior to and after starting ADT that associate with PFS or OS with abiraterone or docetaxel.<br><br>4. Interrogation of peripheral immune changes secondary to initiation of ADT and following addition of docetaxel or abiraterone<br>• To determine changes in Polymorphonuclear myeloid-derived suppressor cells (PMN-MDSC) (CD11b+CD33+CD15+ cells) and monocytic myeloid-derived suppressor cells (M-MDSCs) and immune cells ((Natural killer (NK) cells, T-lymphocytes, CD4+ T-lymphocytes, CD8+ T-lymphocytes and B-lymphocytes)) from peripheral blood leukocyte samples after exposure to ADT and subsequently ADT with abiraterone or docetaxel.<br>• To determine dynamic changes in IL-23 and other cytokines in plasma after exposure to ADT and subsequently ADT with abiraterone or docetaxel.

	• To evaluate whether patients with rising PMN-MDSCs, M-MDSCs or cytokines post ADT and during ADT with abiraterone or docetaxel have a shorter progression free survival (PFS) and radiological progression free survival (rPFS) and OS.<br>• To define the peripheral blood immune profile and correlate with archival tumour tissue and PFS and OS.<br>• To correlate peripheral immune changes pre and post ADT with genomic changes in circulation.<br>To determine T cell receptor (TCR) repertoire changes pre and post ADT and correlate this to PFS and OS.<br><br>5. WBMRI derived imaging biomarkers as a surrogate of response (only at select sites and for patients with no contra-indication to MRI)<br>• To determine utility of WBMRI derived quantitative imaging biomarkers in predicting and assessing early response to docetaxel and abiraterone as determined by PFS and OS and association with plasma tumour markers both at baseline and changes on treatment.<br>• To identify WB imaging biomarkers (at baseline and changes during treatment) which may associate with increased risk of developing mCRPC and may be incorporated into biomarker composite models of response.
Primary Endpoint	PFS for PARADIGM-D and PARADIGM-A will be reported separately and will be defined as the interval from start of docetaxel or abiraterone to disease failure as determined by at least one or more of these factors:<br>1. Symptomatic or asymptomatic new or unequivocal progression of prior distant metastases confirmed by imaging,<br>2. Symptomatic progression of cancer in the prostate confirmed by imaging,<br>3. Serum PSA progression in PARADIGM-D<br>4. Prostate cancer specific death, defined as time from start of abiraterone or docetaxel with ADT to death from prostate cancer
Secondary Endpoint	1. Prostate Cancer Specific Survival (PCSS) defined as time from start of abiraterone or docetaxel with ADT to death from prostate cancer<br><br>2. OS defined as time from start of abiraterone or docetaxel with ADT to death from any cause.
Number of sites:	10
Country:	UK
Trial Intervention	**Treatment summary:**<br>All patients will receive SOC treatment for metastatic prostate cancer that must include ADT with an LHRHa and addition of

	docetaxel or abiraterone. Prednisolone can be used as per local guidelines.<br>**There will be no randomisation and treatment selection will be based on patient and physician choice, funding availability and local guidelines.**<br><br>Men will be recruited to two cohorts concurrently:<br>• ADT + docetaxel, PARADIGM-D<br>• ADT + abiraterone, PARADIGM-A<br><br>Men must have plasma collected at C3D1 of their allocated treatment and additional plasma samples at C1D1, C2D1 and C5D1. Additional samples will be collected pre-ADT* and every three months, including at treatment failure/progression, until initiation of the next line of treatment. (**Sub set of ~50 patients)*.<br><br>Patients will be followed up for at least 36 months for PFS and OS and subsequent treatments will be recorded.<br>All patients will be asked to consent to the collection of their archival diagnostic blocks immediately after consent.<br><br>**Clinical data collection**<br>Clinical data will be collected on a regular basis, including baseline characteristics, treatment details, results of key investigations (including blood tests and scans), patient status (including performance status) and outcome.<br><br>**Pre-ADT sample collection**<br>Research teams will try their best to collect a plasma sample as soon as possible after diagnosis of metastatic prostate cancer and when feasible, prior to start of ADT. If their long-term management plan has not been decided and discussed with them, patients could be invited to sign a pre-study consent form, have a blood sample collected and the date of start of ADT will be recorded. Samples collected after start of anti-androgen but prior to start of LHRHa will be included and will be grouped for analysis based on exposure to anti-androgen . Patients who sign the pre-study consent form will be invited to consent to the main patient information sheet (PIS) at a later date. If patients decline to consent to the PARADIGM study, they will be given the option for their samples to be either retained for translational research or destroyed. We aim to collect plasma from approximately 50 men prior to start of ADT.
Duration of recruitment:	Approximately 18 months
Duration of follow up:	For 60 months from start of accrual or 42 months after last patient registered, whichever occurs first.
Definition of end of study:	For regulatory purposes the end of study will be 60 months after the first patient has been registered, or once all patients have died, whichever is sooner.

1.2 Graphical summary

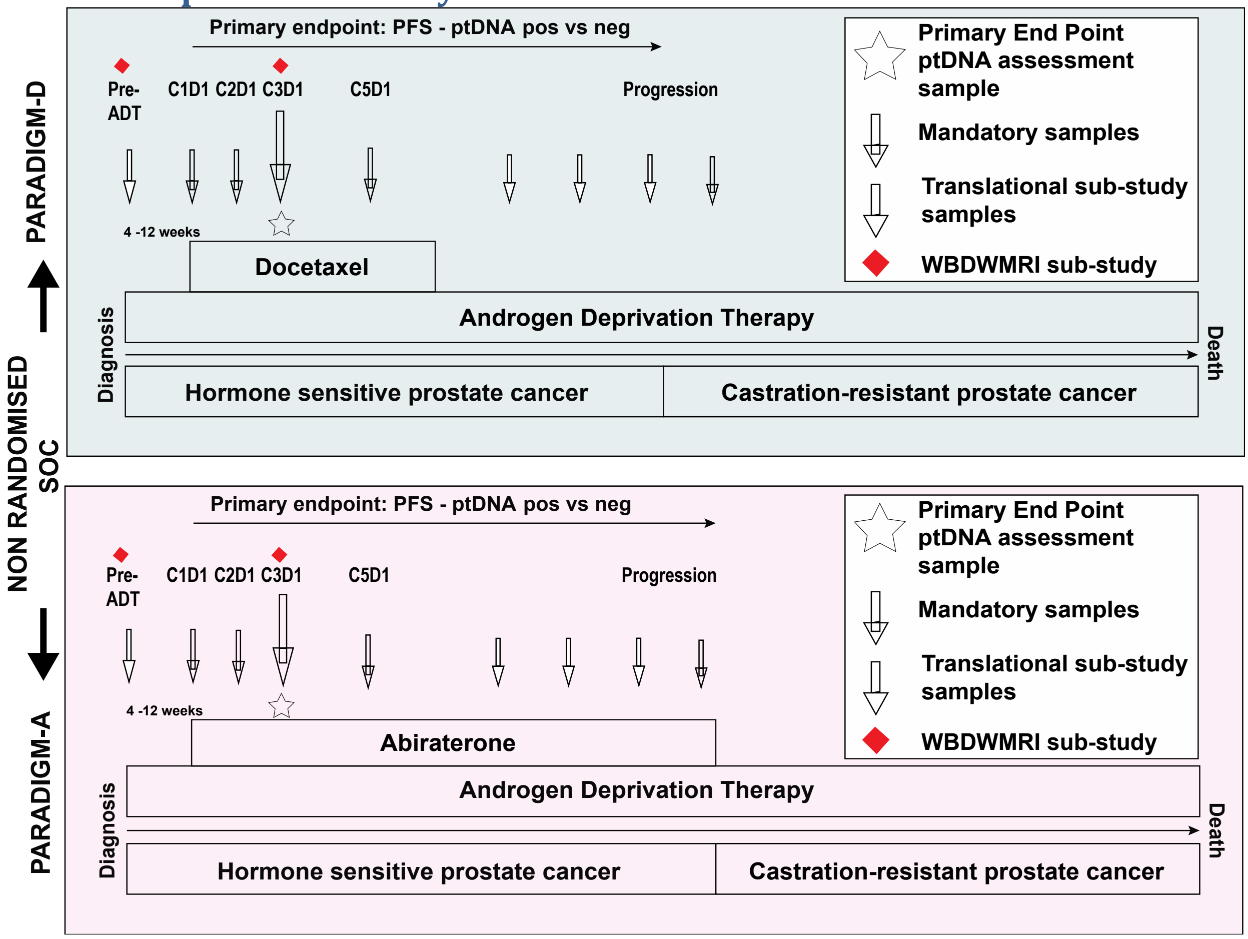

1.3 Funding

Prostate Cancer UK is the main funder of the study and is supporting the central coordination of the study through the UCL CTC (MA-TR15-007). Research A costs will be reimbursed to sites as per the finance section of the Model Agreement for Non-commercial research (mNCA).

Cancer Research UK (grant numbers: C35118/A22744; C65130/A26321) supports some of the translational research.

An MRC Clinical Research Fellowship (MR/P002072/1) supported A Jayaram during the study design and set-up.

Epic Sciences, Inc. is providing support for the collection, shipment and analysis of the Circulating Tumour Cells (CTCs).

2 INTRODUCTION

2.1 Management of de novo metastatic prostate cancer

In the UK, prostate cancer is the most common cancer in men, with about 1 in 8 men diagnosed with prostate cancer at some point of their lives, equivalent to approximately 47,000 men diagnosed every year. Between 2014-2016, there were approximately 11,500 prostate cancer deaths annually in the UK[1]. Up to a third of prostate cancer deaths in the UK arise in men with metastatic disease at diagnosis: de novo metastatic (M1) prostate cancer is a lethal disease and major health care burden.

Until 2015, long term ADT alone was the SOC for patients with newly-diagnosed metastatic prostate cancer, with a median time to castration resistance of approximately 13 months[2]. Recently, randomised controlled trials have demonstrated a survival advantage and prolonged PFS for addition to ADT of systemic treatment with either docetaxel with or without prednisolone/prednisone (Doc)[3-7], or more recently, abiraterone acetate with prednisolone/prednisone (AAP)[8,9]. However, despite significant tumour responses in many patients, the majority progress to CRPC that is lethal and leads to significant suffering. There is an urgent need to improve the management of men with de novo metastatic disease. This will require accurate treatment selection, early detection of relapse and deep interrogation of treatment resistance.

The benefit of the addition of Doc to long-term ADT in patients with metastatic prostate cancer was demonstrated in two studies: CHAARTED and STAMPEDE. In the CHAARTED study, the median PFS was 20.2 months in the Doc plus ADT arm versus 11.7 months with ADT alone ((Hazard ratio (HR) 0.61; 95%CI: 0.51-0.72, p<0.001))[7]. Long term survival analysis after a median follow-up of 53.7 months, demonstrated the median OS was 57.6 months for the chemo-hormonal therapy arm versus 47.2 months for ADT alone ([HR], 0.72; 95% CI, 0.59 to 0.89; p=0.0018). For patients with high-volume disease defined as the presence of visceral metastases or ≥4 bone lesions with ≥1 beyond the vertebral bodies and pelvis, the median OS was 51.2 months with chemo-hormonal therapy versus 34.4 months with ADT alone (HR, 0.63; 95%CI, 0.50 to 0.79; p<0.001)[10]. The time to CRPC was 19.4 months in the combination arm versus 11.7 months in the ADT alone arm (HR, 0.61; 95% CI, 0.52 to 0.73; *P* < .001). For high-volume disease, the median time to CRPC was 14.9 months for the combination arm versus 8.6 months for the ADT alone arm (HR, 0.58; 95% CI, 0.47 to 0.71; *P* < .001).

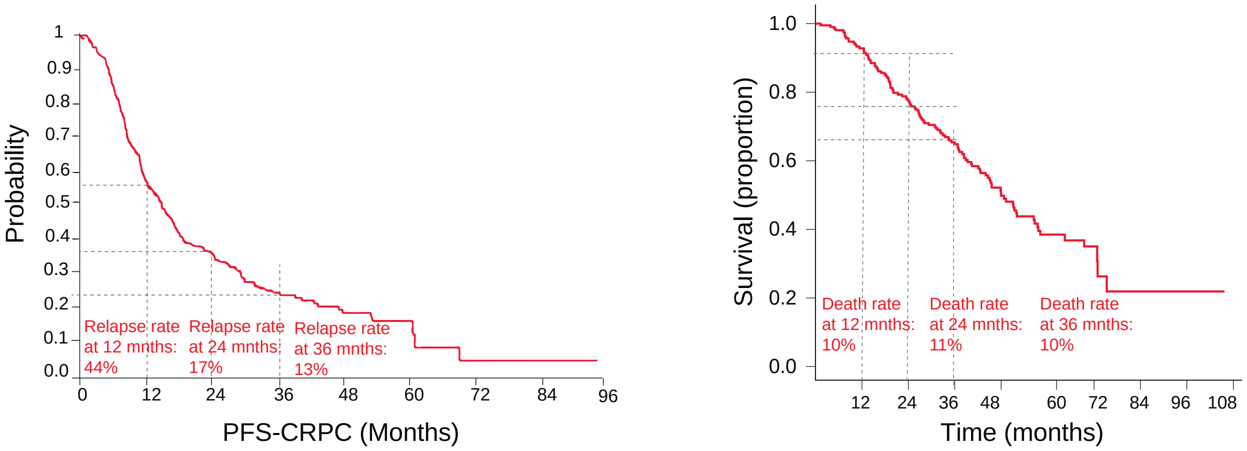

	TOTAL	EVENT	CENSOR	MEDIAN
ADT + D	240	198	55	14.9

No. at risk:

ADT plus docetaxel	214	194	159	118	64	27	11	3	2	0

Figure 1. PFS and OS with ADT and docetaxel in de novo high-volume M1 prostate cancer in the CHAARTED trial with relapse rate estimates at 12 months, 12-24 months and 24-36 months

For M1 patients randomised in the STAMPEDE trial, after a median follow-up of 43 months ((Interquartile range (IQR) 30–60)), the median survival with ADT alone was 45 months ([IQR 23–91], 5-year survival 39%) and 60 months ([IQR 27–103], 5-year survival of 50%) for patients who received ADT with docetaxel (HR) 0·76, 95% CI 0·62–0·92; p=0·005). The proportion of patients who developed CRPC was 81% for ADT alone versus 70% for ADT with docetaxel (HR 0·61, 95% CI 0·53–0·70; p=0·413 × 10^{-13}). A post-hoc analysis of the OS and PFS in high-volume M1 is planned but has not been presented yet.

The benefit of the addition of AAP to ADT is supported by both the LATITUDE[8] and STAMPEDE studies. The LATITUDE study recruited only high-risk metastatic hormone-naive prostate cancer patient defined as meeting at least 2 of 3 high-risk criteria: Gleason score ≥8, presence of ≥ 3 lesions on bone scan or presence of measurable visceral disease. After a median follow-up of 30.4 months, patients receiving AAP and ADT had a longer rPFS of 33 months versus 14.8 months for patients receiving ADT alone (HR=0.47; 95% CI, 0.39-0.55; p<0.001) and a longer OS (not reached vs. 34.7 months; HR=0.61; 95% CI, 0.51-0.76; p<0.001[8].

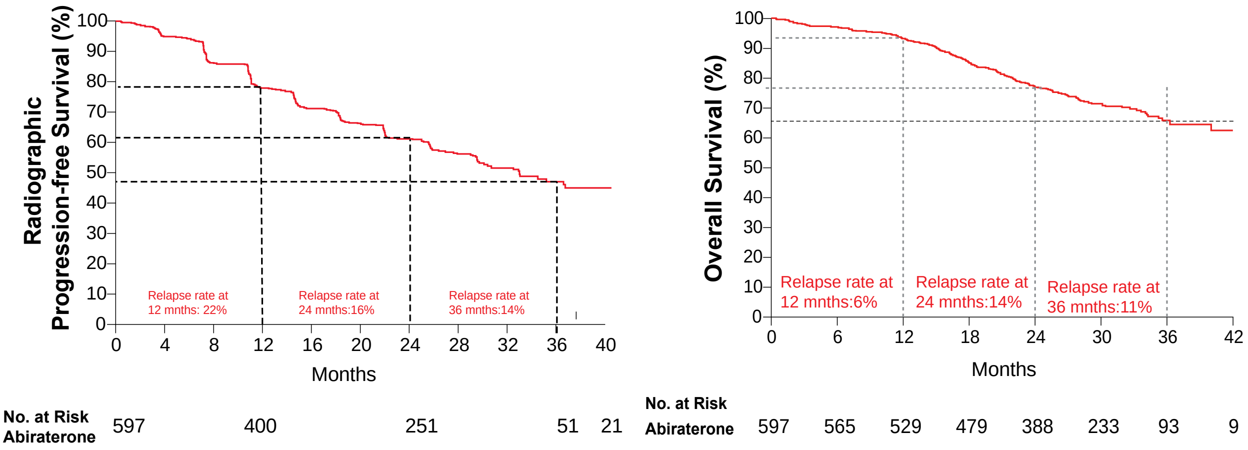

Figure 2. rPFS and OS for high-risk M1 patients in the LATITUDE trial with relapse rate estimates at 12 months, 12-24 months and 24-36 months

In the STAMPEDE trial, after a median follow-up of 40 months, the OS benefit in the high-risk M1 patient population was similarly significant (not reached versus 34 months, HR 0.54 (0.41-0.70) p<0.001)[9]. The STAMPEDE trial did not measure rPFS but reported failure-free survival (as for time to CRPC above). This was 30 months versus 7.9 months in the high-risk M1 group, (HR 0.31 (0.25-0.39) p<0.001)[11]

2.2 Plasma DNA analysis

Plasma DNA can be extracted from the non-cellular blood compartment from all individuals. It is fragmented to an average length of 140 to 170 base pairs (bp) and is very amenable to next-generation sequencing (NGS). In cancer patients, a fraction of plasma DNA is tumour in origin ranging from <1% to >90% of total plasma DNA, with an association observed with increasing tumour or metastatic volume. PtDNA is usually present as only a few thousand amplifiable copies per millilitre of blood [12-14 15-17 18]. Studies have demonstrated that the half-life of ptDNA is around 2 hours, and its release could therefore be a very sensitive indicator of tumour behaviour [19]. This approach has been primarily tested for detection of minimal residual disease(MRD) after primary treatment or for tracking disease response in metastatic disease[12].

The utility of ptDNA to quantify MRD and predict post-operative relapse has been studied in lung cancer. One of the main goals of a study published by Abbosh et al was to examine the capability of detecting MRD and the tumour subclones that drive relapse using patient-personalised ptDNA. The authors collected pre- and post-operative ptDNA

for a sub-group of 24 patients, and patients were followed-up for every 3 to 6 months and up to 31 months for relapse. Of the 14 patients that were confirmed with relapse, 13were ptDNA positive defined as at least 2 single nucleotide variants (SNVs) detected, and the median time between ptDNA detection and relapse confirmation was 70 days. Conversely, 9 out 10 patients who are ptDNA negative lived disease free within the follow-up period. The remaining one patient that had ptDNA detected prior to adjuvant chemotherapy, but remained ptDNA negative after the treatment, was free of relapse 688 days post-surgery. Despite the relatively small sample size, the results of the study demonstrate the utility of circulating tumour DNA (ctDNA) for predicting post-operative relapse of non-small cell lung cancer with both a sensitivity and specificity above 90%[20].

Similarly in colorectal cancer, the detection of ptDNA after resection of stage II colon cancer has demonstrated utility in detecting recurrence. In a study of 230 patients with resected stage II colon cancer, in patients not treated with adjuvant chemotherapy, ptDNA was detected postoperatively in 14 of 178 (7.9%) patients, 11 (79%) of whom had recurred at a median follow-up of 27 months; recurrence occurred in only 16 (9.8 %) of 164 patients with negative ctDNA [(HR), 18; 95% confidence interval (CI), 7.9 to 40; P < 0.001]. In patients treated with chemotherapy, the presence of ptDNA after completion of chemotherapy was also associated with an inferior recurrence-free survival (HR, 11; 95% CI, 1.8 to 68; P = 0.001). This study also evaluated the sensitivity of serial ptDNA analysis during the follow up period to predict subsequent radiologic recurrence. ptDNA was more frequently positive in 23 out of 27 patients than carcinoembryonic antigen (CEA) elevation at the time of radiologic recurrence (85% versus 41%); p=0.002) The time between ctDNA detection and radiologic recurrence (median, 167 days; IQR, 81 to 279 days) was significantly longer than the time between CEA elevation and radiologic recurrence (median, 61 days; IQR, 0 to 207 days; P = 0.04)[21] .

In a prospective cohort of 55 early breast cancer patients receiving neoadjuvant chemotherapy, detection of ptDNA in plasma after completion of apparently curative treatment—either at a single postsurgical time point or with serial follow-up plasma samples—predicted metastatic relapse with high accuracy. Patients with detectable ptDNA in a single post treatment sample had a median disease free survival (DFS) of 6.5 months [HR:25.1 (CI, 4.08 to 130.5; log-rank P < 0.0001)]. Detection of ctDNA in serial samples was predictive of early relapse [disease-free survival: median of 13.6 months (ptDNA detected) versus median not reached (ptDNA not detected); HR, 12.0 (95% CI, 3.36 to 43.07)], with a C-index of 0.75. Detection of ptDNA by mutation tracking was a significant predictor of early relapse in a multivariable model. Mutation tracking in serial samples increased sensitivity for the prediction of relapse, with a median lead time of 7.9 months over clinical relapse[22].

PtDNA analyses in mCRPC have shown a strong association with clinical outcome and clinico-pathological variables [23-27]. In mCRPC, a phase II study of cabazitaxel versus abiraterone or enzalutamide in poor prognosis mCRPC presented at European Society of Medical Oncology (ESMO) 2018, demonstrated that notably no patients with undetectable ptDNA had died in the study. Patients with ptDNA percent of 30-100 had a HR for progression of 4.2 (95%CI 2.04-8.68, p<0.001). ptDNA change on therapy was also highly prognostic - ptDNA increase while on therapy had a HR of 6.24 (95%CI 2.09-16.63, p=0.001) for OS. Additionally on treatment change of ptDNA was prognostic. Patients with an increase in ptDNA fraction between baseline and end of cycle 4 had a shorter PFS (HR 4.26; 95% CI 1.76-10.32, p<0.001) as well as shorter OS [28]. Preliminary data from analysis of plasma DNA collected from M1 patients prior to and after starting ADT shows a rapid decline in ptDNA levels with ~20% of patients remaining with detectable ptDNA on treatment[29].

Studies of sequential plasma samples in mCRPC have identified i) emergence of genomic aberrations harboured by resistant clones several months prior to clinical or radiological progression and ii) drivers of resistance, for example *AR* mutations in patients treated with abiraterone and prednisolone or *BRCA2* reversion mutations in *BRCA2* mutant patients treated with PARP inhibitors[26,30,31] [32]. This introduces the opportunity to expand this approach to analysis of patients treated with ADT and docetaxel or abiraterone. Given tumour fraction may be lower than later stage mCRPC, higher sensitivity approaches will be required. Plasma *AR* copy number (CN) gain is detected in 15% of patients at

development of CRPC and associates with worse clinical outcome in mCRPC patients treated with AR targeting agents [33]. Prostate cancer patients who have plasma *AR* gain at development of CRPC have a significantly shorter response to ADT[34](Figure 3), suggesting pre-existence of the *AR* aberrant clone and introducing the opportunity to detect it prior to development of mCRPC, allowing treatment intensification. These reports are retrospective and potential biases resulting from presenting metastatic burden have not been controlled for.

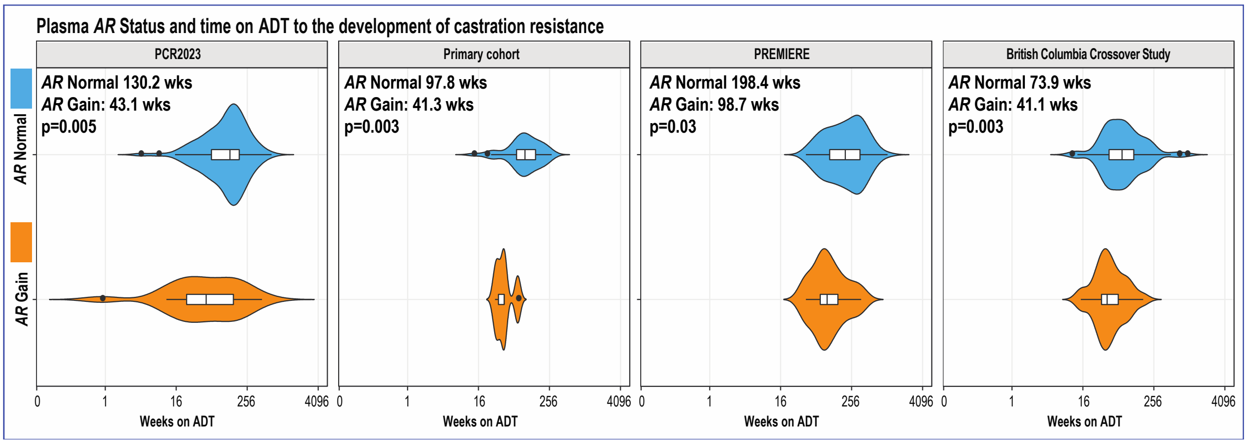

Figure 3. Plasma *AR* copy number status and time on ADT to the development of castration resistance. X axis (weeks) scaled as log 2

PtDNA detection in early disease requires approaches with high sensitivity and specificity that are best achieved by bespoke optimisation for the tumour type and disease setting. For example, amplicon-based or customized target enrichment using improved biochemistry of random molecular barcoding and optimized, error-correcting analysis on ultra-deep sequencing (i.e. >10,000X) can potentially improve the sensitivity of rare mutation and indel detection [35-38]. Epigenetic information, such as DNA methylation status, can be extracted from plasma DNA using modified NGS protocols to obtain information additional to the genomic status [39]. DNA methylation, the addition of methyl group to cytosine, is a modification that occurs at thousands of sites across the genome and is tissue-of-origin and cancer specific. This could be used to improve the sensitivity of assays.

2.3 Nomenclature and categorisation related to disease metastatic status

The CHAARTED and LATITUDE trials pre-defined an M1 patient population with a more homogeneous worse outcome using poor prognosis variables based on radiologically defined metastatic volume or a composite of radiological and pathological criteria (used for sub-group analysis or patient selection) respectively. The CHAARTED trial used the term high-volume and the LATITUDE trial used high risk. The LATITUDE trial composite had one of its requirements as Gleason score >8, which was present in ~85% of patients. The radiological criteria differed slightly between the two trials: both recognised visceral disease as a criterion for inclusion but CHAARTED specified a requirement for bone metastases outside the spine or pelvis whilst the LATITUDE trial required ≥3 lesions on bone scan. Other trials have used higher numbers of bone metastases detected on bone scan for categorising patients, for e.g. the HORRAD trial sub-group analyses used alternative cut-offs of <5 lesions, 5–15 lesions, >15 lesions on bone scan[40]. The sub-group analysis of Arm H in the STAMPEDE trial, namely the randomisation of patients to radiotherapy to the primary tumour in combination with ADT, used the CHAARTED definition for its pre-planned primary analysis but plans sub-group analyses using the LATITUDE and HORRAD definitions[41]. The primary publication of this trial used the term "high burden". Although approximately 18% of patients show a discordance in classification by LATITUDE or CHAARTED criteria, the OS of the ADT alone arms in the two trials was very similar: 34.4 months in CHAARTED and 34.7 in LATITUDE. Post-hoc analysis of patients randomised to abiraterone in the STAMPEDE trial have shown an equivalent benefit for all patients regardless of volume status, using both the CHAARTED and LATITUDE definitions, suggesting

an equivalent treatment effect across all M1 patients [11]. PARADIGM is selecting patients meeting the LATITUDE definition of high-risk disease for the following reasons:

- To maximise detection of ctDNA given the technical challenges for its detection in lower volume patients
- Approval of abiraterone and funding access in the UK will be restricted to this population
- The high relapse rate and relatively short survival make improved outcomes in this population an urgent unmet need.

Patient support groups have expressed concern about the use of terms such as “high-risk” or “high-volume”, as they increase the anguish of a man recently conveyed a diagnosis of prostate cancer. Following discussions with a number of patient representatives, we have decided to use the term polymetastatic. This term will also distinguish from “oligo-metastatic” disease that will be increasingly managed differently and more radically than the polymetastatic population.

2.4 Justification for conducting PARADIGM

As both therapeutic combinations are effective, there are now two distinct standards-of-care for patients with prostate cancer starting ADT. There is currently very little data to guide clinicians as to which is the more effective treatment; direct, randomised, comparative analysis of the ADT and Doc with the ADT and AAP arm showed no evidence of a difference in overall or prostate cancer-specific survival, nor in other important outcomes such as symptomatic skeletal events[42]. Moreover, multiple new therapeutic approaches are being considered for evaluation in this setting. Physicians use a maximum of six cycles of docetaxel and continue abiraterone until radiographic disease progression. Earlier detection of treatment futility or predictive biomarkers of sensitivity could minimise unnecessary toxicity and maximise efficacy with improved treatment sequencing. A PSA level (<0.2, 0.2-4, >4ng/dl) after 7 months of starting ADT with or without docetaxel is associated with shorter survival (85% vs 73% vs 55%) but is insufficiently sensitive to be used alone to guide treatment [43] and the 7-month time-point for analysis limits utility for early discontinuation of ineffective treatment. The challenges with using serum PSA have been highlighted in several studies in mCRPC and include the relatively long half-life of serum PSA and due to its exquisite androgen-regulated sensitivity, serum PSA may not be reflective of less androgen-regulated clones[44]

The PARADIGM study primary endpoint is PFS represented as progression to CRPC and initiation of the next line of treatment. The criteria used by physicians for initiating first-line treatment for mCRPC differ by whether docetaxel or abiraterone are used and this is reflected in the definition of the primary endpoint in PARADIGM-D and PARADIGM-A. OS is not the primary endpoint due to the impact subsequent effective treatments could have an OS that would not be captured by ptDNA after two cycles of docetaxel or abiraterone.

2.5 Immune Profiling in Metastatic Prostate Cancer

The well-established dependency of cancer cells on the tumour microenvironment indicates that the microenvironment might control the emergence of CRPC. Changes in peripheral blood cell fractions of the innate and adaptive immune system have been found in mCRPC when compared to sex-matched healthy volunteers. In mCRPC, it was noted that there was a trend for decreased levels of NK cell (p=0.09), lower T-lymphocytes (p<0.0001), CD4+ T-lymphocytes (p<0.0001), CD8+ T-lymphocytes, as well as a trend for a lower CD4 over CD8 ratio (p=0.11), and decreased B-lymphocytes (p<0.003). Monocytic myeloid derived suppressor cells (M-MDSC) levels are increased in the peripheral blood of mCRPC patients, with an increase in M-MDSC levels following 1 or 2 lines of hormonal treatment (abiraterone or enzalutamide), mirrored by a decreased Human leukocyte antigen – DR isotype (HLA-DR) expression on mature monocytes. M-MDSCs were found to be associated with a shorter PSA-PFS (HR 1.072 with

95%CI 1.001-1.149, p=0.047). PMN-MDSCs is also associated with a lower likelihood of PSA response (OR 0.310 with 95%CI 0.101-0.954, p=0.001). M-MDSC and CD14+CD15+ PMN-MDSC were associated with shorter OS (HR 1.064 with 95%CI 1.021-1.109, p=0.003 and HR 1.13 with 95%CI 1.018-1.247, p=0.021, respectively). Conversely, NK and CD4+ T-cells were associated with a longer OS (HR 0.580 with 95%CI 0.366-0.917, p=0.02 and HR 0.962 with 95%CI of 0.932-0.993, p=0.015, respectively)[45].

Analysis of biopsies from patients with CRPC compared with castration-sensitive prostate cancer (CSPC) revealed that CRPCs had an enrichment of PMN-MDSC ($CD11b^+CD33^+CD15^+$ cells) but not $CD11b^+CD15^-$ which were localized in close proximity to Epithelial Cell Adhesion Molecule (EpCAM) + epithelial tumour cells. In mouse models of CRPC, PMN-MDSC infiltration is linked to AR activation, conferring castration resistance. With castration, PMN-MDSCs number increased over time, paralleling the emergence of CRPC. While PMN-MDSCs increased in castrated tumours, the frequency of tumour associated macrophages (TAMs) were decreased. It has been noted that PMN-MDSCs represented the major subset of immune cells that increased in Pten-null tumours upon castration. Additionally, mouse models of PTEN null prostate tumours are heavily infiltrated by a population of infiltrating $CD11b^+Gr\text{-}1^+$ myeloid cells. These cells protect a fraction of proliferating tumour cells from senescence and therefore maintaining tumour growth. It has been suggested that MDSCs may drive chemo-resistance in human prostate cancer. In the adjuvant setting, it was observed that prostate cancer patients having tumours infiltrated by CD33fl myeloid cells, relapsed after docetaxel treatment[46].

Tumor-infiltrating MDSCs secrete IL23, which induce transcription of *AR* target genes, confer resistance to androgen deprivation and promote prostate cancer cell proliferation and survival[47]. In CRPC patients IL23 is expressed in PMN-MDSCs from biopsies and plasma levels of IL-23 was substantially higher than in patients with CSPC. Plasma IL-23 levels statistically correlated with tumour-infiltrating PMN-MDSC counts (EpCAM−CD11b+CD33+CD15+ cells) but not with other myeloid cell population counts (CD11b+CD15− cells).

MDSC blockage has recently been shown to revert docetaxel chemo-resistance in a mouse model of prostate cancer, thus suggesting that combinatorial approaches aimed to affect MDSC trafficking or functionality should be taken in consideration. Indeed, docetaxel-induced senescence and efficacy was increased in Pten-null prostate tumours when the percentage of tumour was reduced by treating the mice with an antagonist of CXC chemokine receptor 2 (CXCR2). Therapeutically, docetaxel-induced senescence and efficacy were higher in PTEN null tumours when the percentage of tumour-infiltrating $CD11b^+Gr\text{-}\ 1^+$ myeloid cells was reduced using a CXCR2 antagonist [46].

Some studies have suggested that ADT with a Gonadotropin-releasing hormone (GnRH) analogue 1) can induce an expansion of the naïve T-cell compartment with continued thymic output; 2) may decrease the T-cell activation threshold; and 3) may elicit changes and adaptive responses by 1 month after beginning therapy with changes persisting over the course of therapy. This may augment the desired effects prostate cancer-directed immunotherapies by increasing the pool of naïve T-cells which could respond to the immunotherapy, by enhancing T-cell responsiveness, and these effects may persist over the course of androgen deprivation. Patients treated with ADT develop persistent changes in adaptive immune responses. In particular some patients developed a continued expansion of naïve T-cells through thymic output demonstrated by an increase in the CD4+ naïve T-cell (CCR7+, CD45RO-) and Recent Thymic Emigrants (RTE) (CD31+, CD45RO−) population. This suggests the pool of T-cells with greater TCR diversity may be present after ADT. In addition, T-cells in the periphery of treated patients proliferated more robustly to TCR and co-receptor stimulation, and IgG responses developed to proteins of the prostate which could be detected in the sera by one month after beginning ADT[48].A number of studies have suggested that whole-blood gene profiling could identify gene- expression signatures that stratify patients with castration-resistant prostate cancer into distinct prognostic groups[49,50].

2.6 Circulating tumour cells in Metastatic Prostate Cancer

Several studies in mCRPC have shown CTC count to be strongly prognostic [44]. Molecular analyses have suggested additional utility. The detection of AR-V7 in CTCs is associated with worse outcome with AR targeting therapies. AR-V7 is a splice variant that encodes a truncated AR protein that lacks the C-terminal ligand-binding domain but retains the transactivating N-terminal domain[51]. Although the resulting truncated proteins are unable to bind ligand, they are constitutively active as transcription factors and capable of promoting activation of target genes. The association between the detection of AR-V7 messenger RNA (mRNA) in an enriched (selected) fraction of CTCs, poor PSA responses, and shorter radiographic progression-free survival times after treatment with AR targeting therapy was first reported in 2014[52,53]. Follow-up studies with the same assay showed not only a negative association with OS for patients positive for AR-V7 who were treated with AR targeting therapy but also that PSA response and survival with taxane-based therapy were not affected by AR-V7 status[54].

Use of the mRNA determinant as a blood-based biomarker has limitations such as stability of the blood sample, which varies as a function of the collection tube used and the time to sample processing, and in the case of a transcription factor such as AR-V7, an inability to discern if the coded protein is actually localized in the nucleus of cells where it functions to drive tumour growth. The Epic Sciences platform has developed a protein-based assay that can discern the presence and cellular localization of AR-V7 protein in CTCs. This approach deposits all nucleated cells from a patient's blood sample onto proprietary, positively charged slides and uses fluorescent scanners to image each cell and identify CTCs. The approach enables a high sensitivity of CTC detection with minimization of cell loss or damage[55-58],as well as protein biomarker assessment on individual CTCs. Higher PSA response rates, longer radiographic progression-free survival times, and better OS were observed among patients with detectable nuclear-localised AR-V7– positive CTCs who received taxanes, relative to those who received AR targeting therapy[55,59]. Additional value appears to arise in mCRPC from including molecular features in prognostic algorithms, including CTC nuclear AR-V7 expression, features of intra-patient inter-cellular heterogeneity and indices of genomic instability[55,57]. Utilizing digital pathology features on individual CTCs enables defining phenotypically distinct cell types. This enables heterogeneity to be quantified on the basis of the diversity of cell types in individual patient samples using the Shannon index. Low CTC phenotypic heterogeneity was associated with better OS in patients treated with AR targeting therapy, whereas high heterogeneity was associated with better OS in patients treated with taxane chemotherapy[57]. Limited data for CTC analysis exists for de novo polymetastatic prostate cancer but capture and analysis of single CTC may provide additional information to tissue biopsy and plasma DNA analysis.

Micro-fluidic based and very sensitive CTC capture platforms combined with PSA/ Prostate specific membrane antigen (PSMA) dual immunophenotyping detected CTCs in patients with newly-diagnosed metastatic prostate cancer. AR activity was predominantly positive among the patients with detectable CTCs, with the vast majority of CTCs showing an "AR-on" phenotype (PSA^+/ $(PSMA)^-$). The initiation of ADT resulted in a change from an "AR-on" to an "AR-off" phenotype in the majority of CTCs within a month of treatment and complete disappearance of CTCs by 3 months of treatment[60]. However overall, the data on CTC at start of ADT is limited and their analyses in PARADIGM could constitute a novel contribution.

2.7 Whole body diffusion-weighted MRI for hormone sensitive metastatic prostate cancer

Isotope bone scans and computed tomography (CT) remain the SOC imaging modalities for the assessment of metastatic prostate cancer though suffer from widely known limitations in evaluating baseline disease burden as well as monitoring treatment response[61,62]. Sensitivity for metastatic disease detection on CT is low; sub-centimetre metastatic lymph nodes are incorrectly ascribed as normal and early bone lesions are not visible[63]. Whole-body technetium-labelled bone scan is insensitive for early disease detection as it images the reaction within bone to disease presence rather than tumour itself[64], as such bone scan are inferior to emerging imaging methods in bone lesion detection[65]. Early response assessment is also problematic, for example, on CT in the absence of extra-osseous soft tissue disease but may be seen late when normal trabecular bone is restored[61].

Adding to the complexity of imaging assessment is that metastatic lesions demonstrate considerable inter-patient as well as intra-patient/inter-metastasis heterogeneity.Whole-genome and deep sequencing analyses on metastatic biopsies from prostate cancer, has demonstrated that after sharing a common clonal origin, individual metastases may differentiate independently of each other and/or the primary tumour[66]. There is therefore a clearly recognised requirement for the development of imaging biomarkers to better detect disease, monitor treatment and potentially predict response to guide therapy in concert with ongoing molecular blood-based testing. A potential added benefit of imaging is its ability to assess each tumour site individually (rather than a global assessment afforded by plasma biomarkers for example) to further tailor treatment.

Although nuclear medicine scans such as choline and PSMA PET (Positron emission tomography) -CT have shown promising results for disease detection, their values for response monitoring remains largely unclear and limited to small cohort studies. Furthermore, PET-CT scans impart substantial radiation dose, are largely limited to tertiary centres and are expensive to run[67].

Whole body diffusion weighted magnetic resonance imaging (WB-DW-MRI) is a non-ionising imaging technique that can be used to assess a variety of cancers, including Prostate cancer at a lower cost compared with PET-CT. It has been shown that WBMRI is superior to conventional imaging techniques (including bone scan, CT, PET-CT) for bony disease detection in variety of cancers[68,69], including metastatic Prostate cancer[70]. Additionally, using WBMRI derived quantitative imaging biomarkers (QIBs), one can interrogate multiple aspects of tumoural microenvironments including cellularity, vascularity and fat content in one scanning session such that early post-treatment QIB changes have been shown to address current shortcomings of global measurements of response in multiple myeloma[71]. Ongoing work done at UCL Centre for Medical Imaging has looked at WBMRI derived signal fat fraction in lymph nodes as a marker of nodal disease status and treatment response in patients with radio-recurrent prostate cancer capable of providing a QIB to classify pre-treatment nodal disease status whilst change in signal fat fraction may help identify responding lymph nodes (ROC-AUC 0.86). A translational sub-study of WBMRI within the PARADIGM study represents an opportunity to assess the utility of QIB derived from WBMRI in assessing response in concert with other state of the art biomarkers to individualise therapy and their potential to feed into composite biomarker modelling.

2.8 Future implications of PARADIGM

PARADIGM will provide Stage 1 clinical qualification[72] for the association of ptDNA and worse outcome after 1-2 cycles of abiraterone or docetaxel at start of ADT. If positive, our aim will be to then conduct a second study (PARADIGM-2) that will evaluate improved outcomes by changing treatment after one or two cycles in ptDNA positive patients. This second study would be required to provide the Level 1 evidence to change clinical practice. Use of ptDNA as studied in the PARADIGM studies will have a number of implications for patients:

- Reducing exposure to ineffective treatments: less toxicity and greater cost efficiency
- Maximising treatment efficacy by earlier switch to a potentially more effective therapy
- Implementation of biomarkers for improved treatment selection

3 STUDY DESIGN

A prospective, observational, biomarker-focused, translational-platform cohort study in newly diagnosed high-risk metastatic prostate cancer patients starting long-term systemic therapy.

3.1 Study Objectives

3.1.1 Primary Objective:

To determine whether the detection of ptDNA after two cycles of abiraterone (with prednisolone) or docetaxel (with or without prednisolone) added after start of ADT is associated with a worse clinical outcome in newly diagnosed polymetastatic prostate cancer.

3.1.2 Secondary Objectives:

1. To compare ptDNA classification at C2D1 and C5D1 with C3D1.
2. To determine whether the detection of ptDNA after four to twelve weeks of starting ADT and prior to starting abiraterone or docetaxel associates with a worse clinical outcome.
3. To determine the association between clinical outcome and prostate specific antigen (PSA) level (<0.2, 0.2-4, >4ng/dl) after four to twelve weeks of starting ADT and prior to starting abiraterone or docetaxel and at C2D1, C3D1, C5D1 (for both abiraterone and docetaxel) and at 7 months after start of ADT.
4. To assess whether ptDNA detection is a better predictor of clinical outcome than PSA level (as assessed in objective 3) after four to twelve weeks of starting ADT and prior to starting abiraterone or docetaxel and at C2D1, C3D1 and C5D1.
5. To compare associations with clinical outcome for the change in ptDNA detection and PSA level (as assessed in objective 3) prior to start of abiraterone or docetaxel and at C3D1.
6. To evaluate whether ptDNA fraction prior to LHRHa (stratified by no anti-androgen versus 2-3 weeks anti-androgen) associates with Progression Free Survival (PFS) and Overall Survival (OS).

3.1.3 Exploratory objective

1. To develop and test a composite model incorporating different biomarkers for assessing response.
2. To describe ptDNA dynamics and compare to PSA kinetics.

3.1.4 Translational objectives

Translational research will be performed on sub-sets of patients from whom the required evaluations are made. It is expected that not all patients will be able to participate in all the translational studies but as a fundamental aspect of PARADIGM, as many patients as possible should be included in these assessments.

1. Predictors or response to systemic treatment
   i. To identify molecular signatures in plasma and tumour that associate with PFS or OS with abiraterone and docetaxel.
   ii. To identity a molecular signature in pre-ADT plasma or tumour that associates with plasma *AR* aberrant status at progression to castration-resistant disease.

2. Tracking of plasma DNA dynamics
   i. To determine whether detection of ptDNA precedes PFS as defined for the primary endpoint.
   ii. To molecularly characterise resistant clones.
   iii. To evaluate whether patients who progress with AR gain at the development of mCRPC have a shorter time to PFS (on ADT) and OS.

3. CTC dynamics at initiation of ADT
   i. To evaluate whether CTC count pre-ADT and after starting ADT associates with shorter PFS and OS.
   ii. To identify CTC molecular features prior to and after starting ADT that associate with PFS or OS with abiraterone or docetaxel.

4. Interrogation of peripheral immune changes secondary to initiation of ADT
   i. To determine changes in PMN-MDSC (CD11b+CD33+CD15+ cells) and M-MDSCs and immune cells NK cells, T-lymphocytes, CD4+ T-lymphocytes, CD8+ T-lymphocytes and B-lymphocytes)) from peripheral blood leukocyte samples after exposure to ADT and subsequently ADT with abiraterone or docetaxel.
   ii. To determine dynamic changes in IL-23 and other cytokines in plasma after exposure to ADT and subsequently ADT with abiraterone or docetaxel.
   iii. To evaluate whether patients with rising PMN-MDSCs, M-MDSCs or cytokines post ADT and during ADT with abiraterone or docetaxel have a shorter PFS and rPFS and OS.
   iv. To define the peripheral blood immune profile and correlate with archival tumour tissue and PFS and OS.
   v. To correlate peripheral immune changes pre and post ADT with genomic changes in circulation.
   vi. To determine TCR repertoire changes pre and post ADT and correlate this to PFS and OS.

5. WBMRI derived imaging biomarkers as a surrogate of response
   i. To determine utility of WBMRI derived quantitative imaging biomarkers in predicting and assessing early response to docetaxel and abiraterone as determined by PFS and OS and association with plasma tumour markers both at baseline and changes on treatment.
   ii. To identify WBMRI imaging biomarkers (at baseline and changes during treatment), which may associate with increased risk of developing mCRPC and may be incorporated into biomarker composite models of response.

3.2 Study Endpoints

3.2.1 Primary end point

The primary endpoint is PFS for PARADIGM-D and PARADIGM-A. This will be reported separately and will be defined as the interval from start of docetaxel or abiraterone to progression to CRPC, usually necessitating a treatment change, as determined by at least one or more of the following factors:

1. Symptomatic or asymptomatic progression of or new distant metastases confirmed by imaging,
2. Symptomatic progression of cancer in the prostate confirmed by imaging,
3. Serum PSA progression in PARADIGM-D,
4. Prostate cancer specific death, defined as death from prostate cancer. Death from any other cause including toxic death will be excluded.
   The calculation is detailed further in section 12.3

3.2.2 Secondary End Point

1. PCSS, defined as time from start of abiraterone or docetaxel with ADT to death from prostate cancer.
2. OS defined as time from start of abiraterone or docetaxel with ADT to death from any cause.

3.3 Study Activation

UCL CTC will ensure that all study documentation has been reviewed and approved by all relevant bodies and that the following have been obtained prior to activating the study:

- Health Research Authority (HRA) approval, including Research Ethics Committee approval
- ‘Adoption’ into NIHR portfolio
- Adequate funding for central coordination

- Confirmation of sponsorship
- Adequate insurance provision

4 SELECTION OF SITES/SITE INVESTIGATORS

4.1 Site Selection

In this protocol study 'site' refers to a hospital where study-related activities are conducted.

Sites must be able to comply with:

- SOC treatment(s), imaging, clinical care, follow up schedules and all requirements of the study protocol
- Requirements of the Research Governance Framework and all amendments
- Data collection requirements, including adherence to Case Report Form (CRF) submission timelines as per section 13.
- Biological sample collection, processing and storage requirements
- Monitoring requirements, as outlined in protocol section 16 (Study Monitoring and Oversight) and the trial monitoring plan

4.2 Selection of Principal Investigator and other investigators at sites

Each sites must appoint an appropriate Principal Investigator (PI), i.e. a health care professional authorised by the site to lead and coordinate the work of the study on behalf of a site. Co-investigators must be trained and approved by the PI. The PI is responsible for the conduct of the study at their site and for ensuring that any amendments are implemented in a timely fashion. If a PI plans to take a leave of absence, UCL CTC must be informed promptly. For absences greater than three months or where the PI is no longer able to perform his/her role, a new suitable replacement PI must be identified by the site and UCL CTC notified. UCL CTC may suspend recruitment at a site where a suitable replacement PI has not been identified within three months

4.3 Training requirements for site staff

All site staff must be appropriately qualified by education, training and experience to perform the study related duties allocated to them, which must be recorded on the site delegation log.
CVs for all staff must be kept up-to-date, signed and dated and copies held in the Investigator Site File (ISF). A current, signed copy of the CV with evidence of Good clinical practice (GCP) training (or copy of GCP certificate) for the PI must be forwarded to UCL CTC upon request.
GCP training is required for all staff responsible for study activities. The frequency of repeat training may be dictated by the requirements of their employing institution, or two yearly where the institution has no policy, and more frequently when there have been updates to the legal or regulatory requirements for the conduct of clinical trials.

4.4 Site Initiation and Activation

4.4.1 Site initiation

Before a site is activated, the UCL CTC trial team will arrange a site initiation with the site which the PI and site research team must attend. The site will be trained in the day-to-day management of the study and essential documentation required for the study will be checked.
Site initiation will be performed for each site initially by site visit. This training will include the management of the blood sample collection. Re-initiating sites may be required when there has been a significant delay between initiation and enrolling the first patient, in accordance with the monitoring plan.

4.4.2 Required documentation

The following documentation must be submitted by the site to UCL CTC prior to a site being activated by the UCL CTC trial team:

- Trial specific UK Site Registration Form (identifying relevant local staff).

- Relevant institutional approvals.
- A completed site delegation log that is initialled and dated by the PI (with all tasks and responsibilities delegated appropriately).
- Completed site contacts form (with contact information for all members of local staff).
- A signed and dated copy of the PI's current CV (with documented up-to-date GCP training, or copy of GCP training certificate).

In addition, the following agreements must be in place:

- A signed model clinical trial agreement (mNCA) between the Sponsor and the relevant institution (usually an NHS Trust/Health Board).

4.4.3 Site activation letter

Once the UCL CTC trial team has received all required documentation and the site has been initiated, a site activation letter will be issued to the PI, at which point the site may start to approach patients.

Following site activation, the PI is responsible for ensuring:

- Adherence to the most recent version of the protocol.
- All relevant site staff are trained in the protocol requirements.
- Appropriate recruitment and medical care of patients in the trial.
- Timely completion and return of Case report forms (CRFs)
- Prompt notification and assessment of all serious adverse reactions (SARs).

5 SELECTION OF PATIENTS

5.1 Screening Log

A screening log will not be mandated for the study. Research teams are encouraged to raise screening concerns that arise at TMG meetings.

5.2 Patient Eligibility

Queries in relation to the eligibility criteria must be addressed prior to registration. Patients are eligible for the study if all the inclusion criteria are met and none of the exclusion criteria applies.

Patients' eligibility must be confirmed by an investigator who is suitably qualified and who has been allocated this duty, as documented on the site staff delegation log, prior to registering the patient. Confirmation of eligibility must be documented in the patients' notes and on the registration CRF.

Patients must give written informed consent before any study-specific screening investigations are carried out. Refer to section 10.1 (Pre-registration) for the list of assessments and procedures required to evaluate the suitability of patients prior to entry.

5.2.1 Inclusion criteria

1. Able and willing to provide written informed consent
2. Prostate adenocarcinoma confirmed on biopsy obtained in previous 6 months
3. Polymetastatic disease defined as two of the following:
   i. Gleason score of ≥ 8,
   ii. Presence of ≥3 lesions on bone scan,
   iii. Presence of measurable visceral lesion
4. Eastern Cooperative Oncology Group (ECOG) Performance status 0 to 2
5. No medical contra-indications to abiraterone or docetaxel
6. Patients should be either of the following:
   i. Planned to start long-term Luteinizing hormone (LH) suppression, or
   ii. within 10 weeks of starting long-term LHRH antagonist, or
   iii. within 12 weeks of starting LHRH agonist or an anti-androgen when the latter is used in combination with or prior to LHRH agonist for flare protection.
7. Patients should be planned for addition of docetaxel (PARADIGM-D) or abiraterone (PARADIGM-A) 5 to 10 weeks after start of LHRHa (or 7 to 12 weeks if LHRH agonist is started without anti-androgen) with a target of 6 cycles or continuation until progression respectively.
8. No concomitant medical conditions likely to reduce life expectancy.
9. Patient agrees to be followed up in the recruiting centre and to having sequential plasma samples collected as per the study protocol.

5.2.2 Exclusion criteria

1. Medically unsuitable for either abiraterone, prednisolone or docetaxel.
2. Concurrent or planned for (within the first 5 cycles of docetaxel or abiraterone) treatment with any experimental drugs, oestrogen patches, radiotherapy or surgery to the primary tumour. Patients randomised to the standard of care (SOC) arm in open-label clinical trials are eligible. Patients who are still to be randomised to STAMPEDE may be included where the randomisation will be limited to SOC or aK. Patients can participate in other observational studies.

3. Prior systemic therapy for prostate cancer other than for LHRHa +/- anti-androgen (started within the time limits defined in inclusion criterion 6).
4. Metastatic brain disease or leptomeningeal disease.
5. Any surgery planned prior to Cycle 3 Day 1 (C3 D1)
6. Other current malignancy or malignancy diagnosed or relapsed within the past 5 years (other than non-melanomatous skin cancer, stage 0 melanoma in situ and non-muscle invasive bladder cancer).
7. Patients who consent to the whole-body magnetic resonance imaging (WBMRI) translational sub-study should have no contraindications to MRI as per local guidelines.

6 INFORMED CONSENT

Sites are responsible for assessing a patient's capacity to give informed consent. There are three separate PIS and consent forms for this study which are further explained below. There is no minimum time that must pass from first approaching the patient before consent can be taken. If the patient wishes, he can sign the consent form on the same day he is approached. Sites must assess a patient's ability to understand verbal and written information in English and whether or not an interpreter would be required to ensure fully informed consent. If a patient requires an interpreter and none is available, the patient should not be considered for the study.

The PI, or, where delegated by the PI, other appropriately trained site staff, are required to provide a full explanation of the study and all relevant treatment options to each patient prior to study entry.

Written informed consent on the current approved version of the consent form for the study must be obtained before any study-specific procedures are conducted. The discussion and consent process must be documented in the patient notes.

Site staff are responsible for:

- Checking that the current approved version of the PIS and consent form are used.
- Checking that information on the consent form is complete and legible.
- Checking that the patient has initialled <u>all</u> relevant sections and signed and dated the form.
- Checking that an appropriate member of staff has countersigned and dated the consent form to confirm that they provided information to the patient.
- Checking that an appropriate member of staff has made dated entries in the patient's medical notes relating to the informed consent process (i.e. information given, consent signed etc.).
- Following registration adding the patients' study number to all copies of the consent form, which should be filed in the patient's medical notes and investigator site file.
- Following registration, giving the patient a copy of their signed consent form, and PIS.

The right of the patient to refuse to participate in the study without giving reasons must be respected. All patients are free to withdraw at any time. Also refer to section 9.

6.1 Consent to Pre-study (ADT sample)

Collection of blood prior to ADT may be logistically challenging due to the indication to start ADT as soon as possible in men with polymetastatic disease. Explaining the PARADIGM study in detail on the day of diagnosis could over-burden patients with information and may not be feasible. Additionally, the treatment plan for the patient may not have been decided and it would therefore not be feasible to consent them to the Main Study. However, scientifically the pre-ADT sample is very valuable as it includes tumour DNA from sensitive clones that will rapidly regress after initiation of ADT.

To facilitate the collection of blood prior to starting anti-androgen or LHRHa, patients who have metastatic prostate cancer who are deemed to be potentially eligible for the study will be provided with a pre-study PIS and asked to consent to collect blood for research purposes. Once they have consented, the pre-ADT sample can be collected. The PIS will state that up to 70mls of blood will be collected, processed and stored for future research but not analysed until patients have consented to the main study.

At a later date, the patient will be provided with the main study PIS and they can then decide whether they agree to proceed onto the PARADIGM study. If they do not consent to the main study, they can choose whether to allow their pre-ADT sample to be used for translational research or request for it to be destroyed.

If patients wish to consent to the Main study at this point or the investigator believes that it is appropriate to do so then they will be provided with the PIS for the Main Study (see below).

6.2 Consent to Main Study

Patients who have previously consented on the Pre-study consent form can be approached for the main study at a later date, along with any other potential patients. The PI, or, where delegated by the PI, other appropriately trained site staff, will decide whether the patient will start docetaxel or abiraterone. There are two separate PIS depending on which treatment the patient will receive and the relevant current approved PIS should be discussed with the patient:

- PIS PARADIGM-A- for patients receiving abiraterone
- PIS PARADIGM-D- for patients receiving docetaxel

6.2.1 Consent to patient directed sample collection

Patients will be provided the opportunity to consent to having a more active role in the collection of their samples. It is the Sites' responsibility to assess a patient's understanding and ensure that they have capability to carry out this task. The patient may consent to this method but the Site could decide that the patient is not suitable. Further information on what is involved is discussed in section 8.4.1.

6.2.2 Consent for feedback of clinically-relevant genetic information

NGS performed in the translational research may identify molecular information of clinical significance. At consent patients will be specifically asked whether they accept clinically relevant information to be fed back to them. Only results which are of established clinical relevance and for which testing would be available under standard NHS genetic testing guidelines will be fed back e.g. germline pathogenic *BRCA1/2* mutations. Any genetic analysis undertaken does not replace clinically indicated investigations as it cannot be guaranteed that results will be fed back in a timely fashion and tests may not be clinically accredited.

The TMG will decide whether referral to a clinical geneticist is recommended. This is to facilitate access to genetic counselling and the required confirmatory testing, and also necessary in order to offer appropriate advice to biological relatives in the event of detection of a germline (inherited) genetic abnormality.

The TMG will review all detected germline variants detected and make the final decision of which are feedback to patients.

6.3 Consent to Whole Body Magnetic Resonance Imaging (WBMRI) (at selected centres only)

Patients who are eligible to receive a WBMRI can will be given the opportunity to consent to this at selected centres. The patient can be provided with the WBMRI PIS and consent form at the same time as the Main study PIS or at a later timepoint. For more information please see section 11.5.

7 REGISTRATION PROCEDURES

Patients can be registered to the study prior to starting ADT treatment or within 12 weeks of starting ADT. Patients cannot be registered after they have started treatment on docetaxel or abiraterone.

7.1 Registration to Pre-study (ADT Sample)

Sites will be provided with a log containing pre-study numbers for patients who have consented to the Pre-study consent form. Once consented, patients will be added to the log sequentially and a pre-study number provided. UCL CTC should then be emailed to confirm the pre-study patient ID and the date sample was taken.

7.2 Registration to Main Study

Patient registration to the Main study will be performed via a remote electronic data capture system hosted by UCL CTC. Please refer to the PARADIGM registration instructions prior to registering a patient. Patients must be confirmed to be eligible and have given consent prior to registration. Site staff responsible for patient registration must request access to the electronic case report forms (eCRF) database by completing their contact details on the site contacts form and delegation log. Access to the database and instructions are provided by UCL CTC. Note that patient initials are required to register a patient. Patients will be registered to either PARADIGM-A or PARADIGM- D depending on which treatment they will receive. Upon registration a study number will be assigned for the patient and these details appear on the registration confirmation screen. The study number must be recorded in the patient notes. Confirmation of successful registration will be sent to the person registering the patient.

Sites should contact UCL CTC if there are any difficulties in accessing the registration database. If the patient have consented to the patient directed sample collection UCL CTC will contact the site following registration to collect the patients name and telephone number. This will be stored securely on a restricted access password protected spreadsheet.

CONTACT DETAILS	
**PARADIGM Trial Coordinator:**	**020 7679 9351**

Once a patient has been registered onto the study they must be provided with the following:

- A copy of their signed consent form and PIS
- Patient samples diary
- Samples kit to include patient instruction, blood tubes, labels, and worksheets (For selected patients see section 8.4.1)

8 STUDY INTERVENTION

8.1 Treatment Summary

All patients will receive SOC treatment and there will be no randomisation. Treatment selection will be based on patient and physician choice, funding availability etc.
Toxicity, overdose, allergic reactions and concomitant medicine interactions will be managed according to local guidelines and do not need to be specifically reported to UCL CTC.

8.2 Standard of care ADT

If LHRH antagonists are used (with no prior anti-androgen), docetaxel or abiraterone should be started within 5 to 10 weeks. If LHRH agonists are used docetaxel or abiraterone should be started within 7 to 12 weeks or 5 to 10 weeks if anti-androgen has been used for a minimum of 2 weeks (+/- 3 days) previously. Single-agent anti-androgen is allowed for up to 3 weeks prior to LHRH agonists. Anti-androgen monotherapy is not permitted as a form of long-term ADT.

8.2.1 Standard of care docetaxel

Docetaxel will be given according to local protocols as a standard non-trial treatment.

8.2.2 Standard of care abiraterone

Abiraterone in combination with prednisolone will be given according to local protocols as a standard non-trial treatment. Recruitment to PARADIGM-A will be dependent on approval of NHS funding.

8.3 Prednisolone switch to dexamethasone

Some physicians may choose to continue abiraterone after failure but change prednisolone to dexamethasone. For the purposes of primary endpoint, the progression event on abiraterone will be defined when the definition is met, either on first-line or second-line glucocorticoid (prednisolone). (see section 3.2.1 for definition of primary endpoint).

8.4 Research blood sample collection

Blood samples for Research will be collected at the following timepoints for the following analyses. Blood samples can be taken up to 72 hours prior to pre-specified time points.

Timepoint	Samples to be taken
Pre-ADT[1]	Plasma (ptDNA), Whole blood (CTCs) and Whole blood (immunoprofiling), PAXGene RNA
Prior to Cycle 1 Day 1[2]	Plasma (ptDNA), Whole blood (CTCs)[3] and Whole blood (immunoprofiling) [3]
Prior to Cycle 2 Day 1	Plasma only (ptDNA)
Prior to Cycle 3 day 1	Plasma (ptDNA), Whole blood (CTCs)[3] and Whole blood (immunoprofiling) [3]
Prior to Cycle 5 day 1	Plasma only (ptDNA) and Whole blood (immunoprofiling) [3]
Every 3 months	Plasma (ptDNA)
At progression	Plasma (ptDNA), and Whole blood (immunoprofiling)[3]

[1]Selected patients who consent on the pre-study consent form or consent to the main study prior to starting ADT.
[2]Abiraterone or docetaxel
[3]Only patients who had pre-ADT sample taken

Pre-ADT research blood samples will be collected from approximately 50 patients (the TMG will review this target on a regular basis and may reduce the number if it is thought that it was affecting accrual). A ptDNA sample taken at cycle 3 day 1 is required for the primary endpoint and any patients who do not have the cycle 3 day 1 taken will not be included in the target 130 patients. A pre-ADT samples is not required for primary endpoint analysis.

There are two methods of collecting blood samples for the study. The method chosen will be determined by each site and will take into consideration logistics such as facilities at site as well as the patient's profile (performance status, capability and social support). Eligible patients must be confirmed by a suitable investigator prior deciding the method of collecting samples.

All Patients included in the study will be encouraged to bring a Patient Sample Diary to clinic with a record of samples taken. Research teams will ensure the correct record of samples in clinic. There will be a dedicated PARADIGM Clinical Trials Practitioner (CTP) employed by UCL CTC to support sites and patients in the collection of research blood samples for the study.

(See section 8.6 for further information on research blood sample processing).

8.4.1 Patient-directed sample collection

For patients who are capable for an "active role" regarding sample collection and have been consented to this will receive a Patient Samples Box at the time of registration, which will include:

- Patient Samples Manual
- Samples Kit with blood tubes, labels, worksheets
- Sample Collection Diary

Once registered, the PARADIGM CTP will telephone the patient for a welcome call. Prior to each clinic visit, patients will receive a call from the CTP to remind them to bring blood tubes to Phlebotomy. Following collection, tubes will be stored at Phlebotomy. The research team will coordinate the samples for collection and will be sent on the same day via post or courier. To maximise efficiency, CTP will be in regular contact with each site to ensure the proper collection of samples.

8.4.2 Site directed sample collection

If the patient does not consent to the patient directed sample collection the Research Team will coordinate the collection of the Research Blood Samples. Samples Kit will be kept at site, and study samples will be taken directly by the research team or via Phlebotomy as agreed at site initiation. All samples will be sent on the same day via post or courier. All patients will receive a copy of a Sample Collection Diary.

8.5 Archival diagnostic block collection

All patients are asked to consent for the use of remaining tissue samples e.g. those obtained at prostate biopsy or following surgery, for use in translational research. These samples are usually stored as Formaldehyde Fixed-Paraffin Embedded (FFPE) tissue blocks at the hospital where the procedure was performed. Recruiting sites will be asked to retrieve tissue samples stored in pathology stores or referring hospitals and send to the UCL Cancer Institute.

Up to 15 sections will be cut and the tumour blocks will be returns to sites if required or when feasible.

8.6 Research blood sample processing

For detailed information on the preparation, storage and shipping of blood samples please refer to the PARADIGM Lab Manual.

8.6.1 Collection of ptDNA sample

Whole blood will be collected into 4 x 10mL plasma collection tubes per time point and handled according to the PARADIGM lab manual/patient samples manual. It is important that plasma collection tubes are repeatedly inverted 8-10 times after collection to ensure adequate mixing of additives. Once extracted, plasma will be stored at -80°C and thawed at the time of analysis. DNA will be extracted using validated protocols. Samples are to be shipped to:

Shipping address:
LAB 205, UCL Cancer Institute
Paul O'Gorman Building
72 Huntley Street
London WC1E 6DD

PtDNA samples will be analysed in batches by team members blinded to clinical outcome. For the primary and secondary endpoint analysis, analytically validated and "fixed" custom targeted next-generation assays performed in a GCLP environment will be used. Patients will be classified into ptDNA positive or negative based on a pre-defined threshold. For pre-ADT samples, the plasma tumour fraction DNA will also be reported.

8.6.2 Collection of CTC samples

Whole blood will be collected in 1 X10mL CTC tubes and gently inverted 8 to 10 times . Samples should be kept at room temperature and shipped on the same day of collection to Epic Sciences, Inc by courier.

Shipping address:
Covance Central Laboratory services SARL
KIT receipt- CENTERLINX Rue Moises- Marcinhes 7
Meyrin1217
Switzerland

8.6.3 Collection of Immunoprofiling samples

Whole blood will be collected in 10ml immunoprofiling tubes and 10ml PAXGene RNA tubes and shipped to:
Shipping address:
LAB 205, UCL Cancer Institute
Paul O'Gorman Building
72 Huntley Street
London WC1E 6DD

9 WITHDRAWAL OF PATIENTS

In consenting to the study, patients are consenting to assessments, collection of biological samples, follow-up and data collection.

9.1 Future Data Collection

If a patient explicitly states they do not wish to contribute further data to the study their decision must be respected, with the exception of essential safety data (see section 14.1.3), and recorded on the relevant CRF. In this event data due up to the date of withdrawal must be submitted but no further data, other than essential safety data, sent to UCL CTC.

9.2 Losses to Follow-Up

If a patient moves from the area, every effort should be made for the patient to be followed up at another participating trial site and for this new site to take over the responsibility for the patient, or for follow-up via GP. Details of participating trial sites can be obtained from the UCL CTC trial team, who must be informed of the transfer of care and follow up arrangements. If it is not possible to transfer to a participating site, the registering site remains responsible for submission of CRFs.

If a patient is lost to follow-up at a site every effort should be made to contact the patient's GP to obtain information on the patient's status.

9.3 Loss of Capacity

Patients who lose capacity during the study would continue in the study for the purposes of data collection only. The data would be sourced from the medical notes and no further contact would be made with the patient. If the patient regained capacity, an Investigator would discuss with the patient their continued participation in the study and together, the patient and Investigator would decide what action, if any, to take.

10 ASSESSMENTS & DATA COLLECTION

All assessments are considered SOC (except of WBMRI) and local guidelines should be followed. However, there are a minimum number of assessments required for patients to be confirmed as eligible for the study and in order to appropriately monitor for response and disease progression.

Please also see Schedule of Events table in Appendix 2.

10.1 Pre-registration

The following is required to evaluate the suitability of patients for the study and should be carried out as part of the patient's SOC:

- Histological confirmation of prostate adenocarcinoma
- Age
- ECOG Performance status (within 1 month of registration)
- Relevant medical history
- Review of prior treatment for prostate cancer
- Review and documentation of ongoing medication taken within 30 days of registration
- Start date of LHRH agonist or antagonist and of anti-androgens if used as cover
- Planned start date of docetaxel or abiraterone
- A whole-body technetium labelled bone scan
- CT scans of the chest, abdomen and pelvis are required to assess visceral disease performed prior to registration. Use of other imaging to assess visceral disease volume status is permitted after discussion with the UCL CTC
- Serum PSA prior to ADT

10.2 Assessments prior to starting ADT

Patients must consent on either the pre-study consent form or the Main study consent form before any samples can be taken

- 4x 10ml blood for ptDNA analysis
- 1 x 10 ml blood for immunoprofiling
- 1 x 2.5ml PAXGene mRNA
- 1 x10 ml blood for CTC analysis
- WB-MRI pre-ADT (only patients who have consented to the imaging translational sub-study and to be performed ideally within 4 weeks of starting ADT and before docetaxel/abiraterone). See section 11.5 for more details.

10.3 Assessments prior to starting abiraterone/docetaxel

The following assessments should be carried out before the patient starts treatment (~within 4 weeks) on either abiraterone or docetaxel and results will be recorded on the trial CRFs.

Cycle 1 day 1

- 4x 10ml blood for ptDNA analysis

- 1 x 10 ml blood for immunoprofiling (only patients who had pre-ADT sample taken)
- 1 x10 ml blood for CTC analysis (only patients who had pre-ADT sample taken)
- Serum PSA
- Serum testosterone confirming castration and as close to collection of the Pre-abiraterone /docetaxel research blood sample as possible
- Serum Lactate Dehydrogenase (LDH) if physicians considered relevant
- Serum Alkaline phosphatase (ALP) if physicians considered relevant
- Full Blood Count including differential of components
- Serum creatinine
- Height and weight

10.4 Assessments during first six cycles of treatment

During treatment with abiraterone/docetaxel the patient should be seen every cycle as per standard practice and the following assessments performed:

Cycle 2 day 1

- Serum PSA
- Serum LDH if physicians considered relevant
- Serum ALP if physicians considered relevant
- 4x 10ml blood for ptDNA analysis

Cycle 3 day 1

- Serum PSA
- Serum LDH if physicians considered relevant
- Serum ALP if physicians considered relevant
- 4x 10ml blood for ptDNA analysis
- 1 x 10 ml blood for immunoprofiling (only patients who had pre-ADT sample taken)
- 1 x10 ml blood for CTC analysis ((only patients who had pre-ADT sample taken)
- WBMRI (+/- 2 weeks from cycle 3 day 1 and only patients participating in imaging translational sub-study only)

Cycle 4 day 1

- Serum PSA
- Whole body technetium labelled bone scan or equivalent (see section 12.3.1)
- CT, chest, abdomen and pelvis or equivalent

Cycle 5 day 1

- Serum PSA
- Serum LDH if physicians considered relevant
- Serum ALP if physicians considered relevant

- 4x 10ml blood for ptDNA analysis
- 1 x 10 ml blood for immunoprofiling (only patients who had pre-ADT sample taken)

Cycle 6 day 1

- Serum PSA
- Serum LDH if physicians considered relevant
- Serum ALP if physicians considered relevant
- 4x 10ml blood for ptDNA analysis

10.5 Assessments after first six cycles of treatment and follow-up

After completion of six cycles of docetaxel or abiraterone, it is not proposed to routinely assess patients for response. However, in order that objective progression can be assessed, it is necessary to have imaging taken at time of best response as judged by the treating clinician and in accordance with SOC.

All patients should have baseline radiological examinations as detailed in Section 10.1. The same imaging should be used throughout for staging assessments. In addition, it is recommended all patients should have scans repeated at 24 weeks (and whenever clinically appropriate) if they were abnormal at baseline, particularly if they have a low PSA value on entry in to the study making biochemical assessment of treatment failure difficult.

After the completion Of 6 cycles

- Serum PSA
- Serum testosterone
- Serum LDH if physicians considered relevant
- Serum ALP if physicians considered relevant
- CT chest, abdomen and pelvis or equivalent
- Whole body technetium labelled bone scan or equivalent
- 4x 10ml blood for ptDNA analysis

Follow up (Sequentially 3-6 monthly)

- Serum PSA
- CT chest, abdomen and pelvis or equivalent
- Whole body technetium labelled bone scan or equivalent
- 4x 10ml blood for ptDNA analysis

10.6 Recording disease progression

Progression is defined as at least an increase of 20% in the sum of the longest diameter of target lesions, including a maximum of five target lesions, or the appearance of new lesions in keeping with Response Criteria for Solid Tumour assessment.

The following assessments should be carried out as part of SOC , where indicated to confirm progression

- Serum PSA

- A whole-body technetium labelled bone scan or equivalent
- CT scans of the chest, abdomen and pelvis or equivalent
-

In addition the following research samples should be taken:

- 4x 10ml blood for ptDNA analysis
- 1 x 10 ml blood for immunoprofiling (only patients who had pre-ADT sample taken)

The following outcomes should be reported on the Progression CRF:

- PSA nadir and PSA progression values.
- Local (primary tumour or pelvic lymph nodes) progression (symptomatic and confirmed radiologically).
- Unequivocal progression or development of new distant metastases.

10.7 Assessments after Disease Progression

Every 6 months (+/- 2 weeks) the following data will be collected:

- Review of start and end date of subsequent treatment for mCRPC.
- Survival data.

11 TRANSLATIONAL RESEARCH

11.1 Predictors of response to systemic treatment

Objective 1:

Molecular analyses of archival diagnostic tissue (mostly formalin fixed paraffin embedded) will be performed, including but not exclusive to low-pass whole genome NGS, targeted high-coverage NGS, whole genome expression array analysis and targeted methylation studies. Areas of tumour will be micro-dissected from 10-micron thick sections to enrich for cellularity. It is anticipated that tumour blocks will be retrieved and successfully sequenced from 70-80% of patients.

Genomic analyses on ptDNA will be performed and the gene-specific or molecular sub-type specific correlation with patient-matched tumour tissue will be evaluated. Based on this finding, a decision will be made (for every molecular question separately) whether to define patients by tissue alone or plasma alone or a combination of tissue and plasma. Pre-ADT plasma is targeted for collection from 30-40% of patients.

Correlations with outcome will be performed for molecular sub-groups defined in ongoing studies in the STRATOSPHERE collaboration.

Objective 2:

Plasma *AR* status at progression will be defined as in Section 11.3, objective 3. Molecular sub-classes will be described as in Objective 1 and whether patients harbouring a specific molecular signature are more likely to progress with *AR* gained versus *AR* normal disease will be assessed. These analyses will be performed only on patients from whom a progression samples. *AR* gain is expected to occur at a higher prevalence (~50%) in patients who progress within the follow-up timelines of the study.

11.2 Tracking of plasma DNA dynamics

Blood for plasma analysis will be collected sequentially on follow-up every 3 months or as clinically indicated. Sequential plasma samples will be subjected to targeted custom next-generation sequencing.

Objective 1:

To define progression by ptDNA criteria and ascertain whether this precedes progression as defined by the criteria utilised in the primary endpoint . The target is to collect ~60% of 3-monthly plasma samples from ~80% of patients. PtDNA progression is defined as:

1. For patients who remain positive on treatment, the date of progression is recorded as the C3D1.
2. For patients who become negative, a second ptDNA positive reading is required. The date of first detection of ptDNA is considered the day of progression.

Objective 2:

Plasma will be subjected to custom NGS that will define both tumour content and clonal and sub-clonal aberrations. Technical validation of NGS calls could be performed using an orthogonal approach such as droplet digital Polymerase chain reaction (PCR).

Objective 3:

AR status will be ascertained from NGS data and defined as the ratio of *AR* copies to control regions on chromosome X (including *ZXDB*). *AR* copy number normal is defined as a ratio <1.93 and gain is defined as 1.93 and greater. The patient will be categorised by the highest *AR* copy number value observed in progression samples.

11.3 CTC dynamics at initiation of ADT

Objective:

1. To evaluate whether CTC count pre-ADT and after starting ADT associates with shorter PFS and OS.
2. To identify CTC molecular features prior to and after starting ADT that associate with PFS or OS with abiraterone or docetaxel.

CTC from 10mls fresh blood (within 72 hours of blood draw) will be collected prior to ADT, prior to start of abiraterone or docetaxel and at C3D1 on abiraterone or docetaxel. Blood will be shipped to a central laboratory and captured onto slides by industry collaborators Epic Sciences using a proprietary technology and slides will be frozen. Slides will be analysed in batches prior to correlations with clinical outcome. CTC count will be reported as the number of cells per ml of blood and CTC will be characterised by feature classes, including but not exclusive to the Epic CTC Heterogeneity or Genome Instability signatures, identified in ongoing prostate cancer studies.
The primary analysis of correlations of CTC features with outcome with be performed by the UCL CTC and Epic Sciences will be blinded to clinical data. Clinical outcome data could be shared with Epic Science for secondary analyses.

11.4 Interrogation of peripheral immune changes secondary to initiation of ADT

Objective:

1. To determine changes in myeloid-derived suppressor cells (PMN-MDSC) (CD11b+CD33+CD15+ cells) and M-MDSCs and immune cells (NK cells, T-lymphocytes, CD4+ T-lymphocytes, CD8+ T-lymphocytes and B-lymphocytes) from peripheral blood leukocyte samples after exposure to ADT and subsequently ADT with abiraterone or docetaxel.
2. To determine dynamic changes in IL-23 and other cytokines in plasma after exposure to ADT and subsequently ADT with abiraterone or docetaxel.
3. To evaluate whether patients with rising PMN-MDSCs, M-MDSCs or cytokines post ADT and during ADT with abiraterone or docetaxel have a shorter PFS and rPFS and OS.
4. To correlate archival tumour tissue immune profile with peripheral blood.
5. To correlate peripheral immune changes pre and post ADT with genomic changes in circulation.
6. To determine TCR repertoire changes pre and post ADT and correlate this to PFS and OS.

Whole blood from will be collected in Ethylenediaminetetraacetic acid (EDTA) tubes prior to ADT, prior to start of docetaxel or abiraterone, at C3D1 and C5D1 of docetaxel or abiraterone and at progression. A sample of whole blood prior to ADT will also be collected in PAXgene mRNA tubes. Samples will be shipped centrally and processed for mRNA extraction, peripheral immune cell profiles and cytokine levels. Approximately 30-40% of patients are expected to donate pre-ADT and subsequent samples for these studies.

Peripheral blood will be immunophenotyped to define B-cells, T-cell populations NK, PMN-MDSC and M-MDSC. Phenotypic analyses of immune cell populations will be performed with fluorescently labelled antibodies to HLA-DR, CD45, CD15, CD33, CD14 and CD11b, CD3, CD4, CD8, CD19, CD20 and CD56. PMN-MDSCs will be defined as CD15+ HLA-DR low CD14-/+ and M-MDSCs as HLA-DR low CD14+ CD15- CD11b+ CD33+ . Plasma cytokines, including IL-23, will be tested using validated enzyme-linked immunosorbent assay (ELISA) assays. Next generation TCR beta sequencing will be performed on peripheral blood mononuclear cells. Whole-blood samples collected in PAXgene RNA tubes will be subjected to a 6 giene prostate cancer specific gene expression model that will consist of *ABL2, SEMA4D, ITGAL, C1QA, TIMP1, CDKN1A.*

Archival tumour tissue will be immunophenotyped and may include RNA sequencing for IL-23 and other cytokines. Additionally, multiplex immunofluorescence will be performed on FFPE sections. Correlations with peripheral blood analyses will be performed.

Plasma genomic changes will be identified using custom next-generation sequencing as planned in Section 11.3 on plasma collected at the same time as blood for immunoprofiling.

11.5 Whole body MRI derived imaging biomarkers as a surrogate of response (selected sites only)

Due to funding and logistical limitations, this translational sub-study will be conducted solely in UCLH and selected other sites. Patients who are eligible for WBMRI and who have consented to this will undergo two WBMRI examinations - baseline defined as prior to initiation of abiraterone or docetaxel (i.e. both PARADIGM-D and -A) and ideally no later than 4 weeks after commencing ADT and the second scan to coincide with primary end point ptDNA assessment sample (e.g. with two weeks of C3D1). All WBMRIs will be performed on a 3T MR scanner and will be limited to maximum of around 60 minutes duration and acquisition will include for example (but not limited to) the following sequences:

- T2-weighted axial free-breathing turbo spin echo images from vertex to mid-thigh using multiple stacks
- Multi-echo Dixon axial breath-hold proton density fat fraction (PDFF) with multiple stacks covering vertex to mid-thigh
- Axial fat-suppressed free breathing echo planar imaging diffusion weighted MRI (with at least 2 b-values)

This will afford the evaluation of at least three QIB (apparent diffusion co-efficient, proton density fat fraction and T2* mapping) and allow the measurement of these at baseline, on treatment and the change between these at each identified disease site (measured by regions or volumes of interest on their respective parametric maps).
Response will be defined based on conventional morphological imaging assessment (e.g. Response Evaluation Criteria in Solid Tumours(RECIST) definitions and/or working group modifications thereof as appropriate) and informed by clinical, histopathological and SOC radiological follow-up as per usual pathways at the end of the study follow-up by consensus including, but not limited to, appropriate radiologist, oncologist and/or pathologist.

12 STATISTICS

The primary aim of this study is to determine whether the detection of ptDNA after two cycles of abiraterone (with prednisone) or docetaxel (with or without prednisone) added after start of ADT is associated with a worse clinical outcome in newly diagnosed polymetastatic prostate cancer. The clinical outcome measures used will be PFS and PCSS.

12.1 Calculation of target number of patients

We require 130 patients for our primary analysis but as we expect that around 20% of patients will not be evaluable for the primary endpoint (see section 12.2), we plan to recruit 170 patients. We intend to have approximately 65 patients recruited to PARADIGM-D and 65 patients to PARADIGM-A. The TMG will regularly review the proportion of recruited patients who will be eligible for primary analysis and may review the accrual target accordingly. The TMG will also review the allocation of patients to PARADIGM-D and PARADIGM-A and can change the target numbers based on drug funding access, local guidelines, changing landscape etc.

For PARADIGM-D, we assume a 50% PFS rate at 12 months in ptDNA positive patients and 80% PFS rate at 12 months in ptDNA negative patients; this represents a HR of 0.322 (comparing ptDNA negative PFS to ptDNA positive PFS). We also assume that 20% of patients recruited are expected to be ptDNA positive. To have at least 95% power to detect a HR of 0.322, we require 40 events (approximately 12 in ptDNA positive patients, 28 in ptDNA negative patients) to be observed in a total of 65 patients (with the aim of recruiting approximately 13 ptDNA positive and 52 ptDNA negative). This assumes a two-sided log-rank test with a type I error control of 10%, and accounts for an expected dropout rate of 5% per year for ptDNA positive patients and 5% per year for ptDNA negative patients.

For PARADIGM-A, we assume a 60% PFS rate at 12 months in ptDNA positive patients and 85% PFS rate at 12 months in ptDNA negative patients; this represents a HR of 0.322. Again, we assume that 20% of patients are expected to be ptDNA positive. To have at least 90% power to detect a HR of 0.322, we require 33 events (11 in ptDNA positive patients, 22 in ptDNA negative patients) to be observed in 65 patients (with the aim of recruiting 13 ptDNA positive and 52 ptDNA negative). This assumes a two-sided log-rank test with a type I error control of 10%, and accounts for an expected dropout rate of 5% per year for ptDNA positive patients and 5% per year for ptDNA negative patients. All sample size calculations were performed using nQuery Advanced (version 8.1.2.0). We assume recruitment of all 65 patients is conducted over 18 months and that the maximum possible follow-up period (time from first patient recruited to end of study) is 54 months.

PARADIGM-D and PARADIGM-A could be reported separately as they have been powered as stand-alone cohorts. The target number of events may be achieved earlier in PARADIGM-D due to i) the shorter time to PFS given the inclusion of PSA progression and previous studies reporting this is shorter than for AAP REF ii) delay to accrual to PARADIGM-A due to funding restrictions. This could lead to PARADIGM-D being reported first.

12.2 Definition of criteria required for a patient to be included in the primary endpoint analysis

As the study primary endpoint is defined by the association of ptDNA at C3D1 with outcome, only patients who meet the following criteria will be evaluable for the primary endpoint analysis:

- Received ADT followed by docetaxel or abiraterone as defined in Section 8.1.1 “SOC ADT”. Up to one-week delay of abiraterone or docetaxel is permitted.
- Received a minimum of two cycles of docetaxel or abiraterone within 8 weeks from start of treatment.
- Plasma sample collected at Cycle 3 Day 1.

- No radiotherapy, surgery or prostate intervention after consent until C3D1. Patients who have an intervention after C3D1 but prior to C5D1 will be included in the primary analysis but dropped from comparisons between C5D1 and C3D1 values.

Any patients who do not fulfil the above criteria can be withdrawn from study follow-up and sequential blood collection. Their samples will be retained and can be used for translational research.

12.3 Primary endpoint: analysis and detailed definition

The main endpoint is PFS as defined in Section 3.2.1.

Analysis will be performed on an Intention To Treat (ITT) basis. An estimate of the HR for PFS will be computed using Cox Regression, providing the assumption of proportional hazards is satisfied. As a sensitivity analysis, we will also perform an adjusted Cox regression where site will be included as a covariate in the model to assess if including study site materially affects the HR estimate. Comparison of PFS between ptDNA positive and ptDNA negative patients will be performed using a log-rank test (separate analyses per treatment arm). Kaplan-Meier (KM) curves will be plotted for each treatment arm to show PFS for ptDNA positive and ptDNA negative patients.

Detailed criteria for the primary endpoint definition are explained below:

12.3.1 Symptomatic or asymptomatic new or unequivocal progression of prior distant metastases confirmed by imaging

To assess objective progression, imaging is required in addition to clinical progression. Metastases are of two types:

Measurable lesions. These can be accurately measured in at least one dimension and the longest diameter is used to calculate progression compared to the smallest lesion detected on scans at best response. The imaging used is at the discretion of the investigator but the same technique should be used throughout. The investigator should be certain that the lesions are prostate cancer metastases. Progression is defined as at least an increase of 20% in the sum of the longest diameter of target lesions, including a maximum of five target lesions, or the appearance of new lesions in keeping with Response Criteria for Solid Tumour assessment.

Non-measurable lesions. All other lesions are included as non-measurable. Progression is defined as the appearance of one or more new soft tissue lesions or two or more lesions on bone scan and/or the unequivocal progression of existing non-target lesions. In PARADIGM-A, when two or more new bone lesions are detected on scans performed at or before C5D1, a second scan is required after at least 6 weeks to exclude this is secondary to a flare phenomenon.

For the purpose of PARADIGM, progression in fewer than 3 metastases and amenable to and treated by radiotherapy does not constitute a progression event.

12.3.2 Symptomatic progression of cancer in the prostate confirmed by imaging

Local disease progression in the pelvis accompanied by symptoms and confirmed radiologically will constitute a progression event. The date of scans should be used to denote the date of progression. Pelvic progression in the absence of symptoms does not constitute progression.

12.3.3 Serum PSA progression

In keeping with clinical practice, serum PSA progression does not constitute a progression event in PARADIGM-A but does in PARADIGM-D. Serum PSA progression is defined as an increase in PSA to more than 50% of nadir taking as reference the lowest recorded PSA level since starting ADT.
The PSA progression value is calculated in one of three ways:

A. If the lowest recorded PSA value in the 24 weeks following randomisation is more than 4ng/ml and more than 50% of the pre-treatment PSA level then the patient fulfils the criteria for immediate treatment failure with progression defined as the date of nadir.

B. For patients whose PSA nadir in the 24 weeks following randomisation is less than or equal to 50% of the pre-treatment PSA level but remains above 4ng/ml, biochemical failure will be defined as a rise of 50% above the nadir level.

C. For patient whose PSA nadir is less than or equal to 4ng/ml, biochemical failure is defined as at least a 50% rise above the nadir value that is also above 4ng/ml.

For B and C, a second, confirmatory PSA value should be obtained between one week and 3 months later. The timing of assessments needs to be considered because spurious rises in PSA can occur e.g. following procedures involving the urinary tract. PSA failure is confirmed if the second value is around the same level or higher i.e. the trend is confirmed. The date of PSA progression should be provided as the date of the **first** raised PSA that fulfilled the study definition of progression. A confirmatory PSA is not required if there is associated radiological progression as defined in section 12.7.1.
Second-line treatment commenced specifically for a PSA rise should not start until the study definition for serum PSA progression has been met. However, if second line treatment does start before the study definition is met then report the closest PSA value prior to the treatment start date as the progression value. This is not required if second line treatment is being started for radiological signs of progression.

12.3.4 Prostate cancer specific death

Death attributed by investigators as probably related to prostate cancer progression and not related to toxic death or death from any other cause.

12.4 Secondary endpoints: detailed definition and analyses

The secondary endpoints of interest are:

- PCSS defined as time from start of abiraterone or docetaxel with ADT to death from prostate cancer.
- OS defined as time from start of abiraterone or docetaxel with ADT to death from any cause.

Patients recruited to both treatment arms could be combined and an estimate of the HR for PCSS and OS by ptDNA status will be calculated.
With reference to the secondary objectives, secondary analyses will be conducted on each survival outcome (PFS, PCSS and OS) as follows:

1. ptDNA classification at C2D1 and C5D1 with C3D1: the number of patients in each treatment arm who are deemed ptDNA positive at C2D1, C3D1 and C5D1 will be presented in tables. Comparisons to the number at C3D1 will be performed using Fisher's exact tests.
2. To determine whether the detection of ptDNA after four to twelve weeks of ADT and prior to starting abiraterone or docetaxel (timepoint "pre-") associates with a worse survival outcome: Number of patients who are ptDNA positive and negative at this timepoint will be presented. KM estimates for each ptDNA status at this timepoint will be computed. Comparison between groups will be performed using a log-rank test.
3. To assess the association between each survival outcome and PSA level (<0.2, 0.2-4, >4ng/dl) at C2D1, C3D1 and C5D1 and at 7 months after start of ADT, KM estimates of PFS/PCSS/OS rates at the specified timepoints will be calculated. PFS/PCSS/OS rates per PSA level will be compared using Cox regression up to and including the timepoint of interest (assessing HR of PSA levels).
4. To assess whether ptDNA detection is better for predicting each survival outcome at PSA level after four to twelve weeks of starting ADT and prior to starting abiraterone or docetaxel and at C2D1, C3D1 and C5D1. Differences in time between PSA rise and ptDNA detection in progressors and non-progressors will be assessed (distribution and summary statistics).

5. To compare associations with each survival outcome for the change in ptDNA detection prior to start of abiraterone or docetaxel (timepoint "pre-") with ptDNA detection at C3D1, the number of patients who belong to each of the following four groups will be presented; (+ve pre-, +ve on treatment), (+ve pre-, -ve on treatment), (-ve pre-, +ve on treatment), (-ve pre-, -ve on treatment). PFS/PCSS/OS status will be evaluated at C3D1 compared across the four groups (use Cox regression to obtain hazard ratios against a reference group of interest).
6. To evaluate whether ptDNA fraction prior to ADT associates with worse survival outcomes, continuous measurement of ptDNA taken prior to ADT will be assessed (histogram and summary statistics). The association with each survival outcome will be done using time-to-event analyses (accounts for censoring of observations).

Analyses with PFS will be performed separately for each treatment cohort and we could consider pooling the cohorts for secondary analyses if the magnitude of effect in both cohorts appears consistent. Analyses with PCSS and OS will be performed with both arms pooled together and also separately for each treatment arm. In pooled analyses, we may adjust for treatment assignment by including a treatment covariate in Cox regression models.

12.5 Exploratory endpoint

To develop and test a composite model incorporating different biomarkers for assessing response: this exploratory analysis will use regression methods, with response category/survival status as outcome variables and biomarkers as covariates.

12.6 Statistical plan for Translational Research

Translational research will be performed on sub-sets of patients from whom the required evaluations are made. It is expected that not all patients will be able to participate in all the translational studies but as a fundamental aspect of PARADIGM, as many patients as possible should be included in these assessments.

12.6.1 Predictors of response to systemic treatment

Objective 1

To identify molecular signatures in plasma and tumour that associate with PFS or OS with abiraterone or docetaxel: KM estimates of survival outcomes across different molecular signature subgroups will be calculated and plotted for comparison. Pairwise comparisons to a reference subgroup category via log-rank tests may be performed to estimate the effect of different subgroups on survival outcomes.

Objective 2

To identify a molecular signature in pre-ADT plasma or tumour that associates with plasma *AR* aberrant status at progression to castration-resistant disease: distribution of *AR* gain and *AR* normal statuses across molecular subgroups will be presented to assess which subgroups account for at least 90% of the *AR* gain study population.

12.6.2 Tracking of plasma DNA dynamics

Objective 1

To determine whether detection of ptDNA precedes clinical, biochemical or radiological progression: amongst men who have confirmed clinical, biochemical or radiological progression, time between ptDNA detection and confirmation of progression will be calculated. Data may be left-censored (i.e. date of recorded ptDNA detection is an upper-bound for when it reached a detectable level, due to timing of visits/scans). Similarly, we may not detect ptDNA at the time of progression. Using appropriate survival analysis methods to account for this potential missingness, we will estimate the expected time between ptDNA detection and progression confirmation and test to see if this is significantly different from 0.

Objective 2

Genomic changes in sequential plasma samples will be reported for each patient separately although changes recurrent across patients could be grouped. A progressive rise in the abundance of an aberration supports its association with progression. The probability of falsely detecting a change that associates with resistance is reduced when detected in >1 sequential samples, improving the power for testing of multiple genes to identify ones that temporally associate with resistance.

Objective 3

To evaluate whether patients who progress with *AR* gain at the development of mCRPC have a shorter time to PFS (on ADT) and OS: Log-rank test effect of *AR* status on survival outcomes will be performed.

12.6.3 CTC dynamics at initiation of ADT

Objective 1

To evaluate whether CTC count pre-ADT and after starting ADT associates with shorter PFS and OS: Cox regression with survival outcome and CTC count as a time-varying covariate will be performed to assess possible associations.

Objective 2

To identify CTC molecular features prior to and after starting ADT that associate with PFS or OS with abiraterone or docetaxel. KM estimates of survival outcomes across subgroups defined by CTC molecular features will be calculated and plotted for descriptive comparison.

12.6.4 Interrogation of peripheral immune changes secondary to initiation of ADT

To determine changes in myeloid-derived suppressor cells, immune cells and cytokines from peripheral blood leukocyte samples after exposure to ADT and subsequently ADT with abiraterone or docetaxel. Mean differences in cell counts between ADT exposure and ADT with abiraterone or docetaxel will be calculated and summarised, complete with 95% confidence intervals. T-test/sign tests of differences from pre-study therapy (after ADT but before abiraterone or docetaxel) will be conducted.

12.6.5 Novel imaging as a surrogate of response

Differences in baseline and on treatment QIBs and proportional changes between the two may be compared between response groups e.g. with Mann Whitney U test and pairwise QIB changes between these could be compared with Wilcoxon signed rank test. As part of an 'all lesion' QIB analysis- accounting for multiple samples per patient will be required e.g. using linear mixed method modelling using response as the fixed factor and patient as the random factor.

13 DATA MANAGEMENT AND DATA HANDLING GUIDELINES

13.1 Entering data into the eCRF

The eCRF must be completed by staff who are listed on the site staff delegation log and authorized by the PI to perform this duty. Each authorized staff member will have their own unique login details for the eCRF. They must never be shared among staff as the eCRF audit trail will record all entries/changes made by each user. The PI is responsible for the accuracy of all data reported in the eCRF.

The use of abbreviations and acronyms must be avoided.

13.2 Corrections to eCRF Forms

Corrections can be made to data on the eCRF where necessary, the eCRF audit trail will record the original data, the change made, the user making the change and the date and time.

13.3 Missing Data

To avoid the need for unnecessary data queries, fields should not be left blank on the eCRF. If data is unavailable, please refer to the eCRF user guide for information on how to indicate that data is "Not Done", Not Applicable", "Not Available" or "Not Known" (only use if every effort has been made to obtain the data).

13.4 Timelines for Data Entry

The relevant eCRF forms must be completed as soon as possible after a patient's visit.

Sites who persistently do not enter data within the required timelines may be suspended from recruiting further patients into the study by UCL CTC and subjected to a 'for cause' monitoring visit. See section 16.2 for details.

13.5 Data Queries

Data entered onto the eCRF will be subject to some basic checks at the time of entry, and any discrepancies will be flagged to the user in the form of a warning. The data can be corrected immediately, or where this is not possible, the warning can be saved and the data amended at a later stage.

Further data review will be carried out at UCL CTC and queries raised where necessary. Further guidance on the process for handling data queries can be found in the eCRF user guide.

14 SAFETY REPORTING

14.1 Definitions

The following definitions have been adapted from Directive 2001/20/EC, ICH E2A "Clinical Safety Data Management: Definitions and Standards for Expedited Reporting" and International Conference of Harmonisation-Good Clinical Practice (ICH GCP E6).

This is a low-risk prospective observational cohort study.

14.1.1 Adverse Reactions (AR)

All untoward and unintended events related to a 'Study Procedure'; where a causal relationship between a 'Study Procedure' and an event is at least a reasonable possibility, i.e. the relationship cannot be ruled out.

14.1.2 Related & Unexpected SARs

A serious adverse reaction, the nature or severity of which is not consistent with the applicable Study Procedure.

Study Procedure means the blood sampling procedure for the purposes of obtaining research samples within the study.

14.1.3 Serious Adverse Reactions (SAR)

SARs are not anticipated in this study however should they occur they will be reported immediately to the UCL CTC and documented on the study database.

SARs are adverse reactions that meet any of the following criteria:

- Results in death.
- Is life-threatening (the term "life-threatening" refers to an event in which the patient was at risk of death at the time of the event. It does not refer to an event that hypothetically might have caused death if it were more severe).
- Requires inpatient hospitalisation or prolongs existing hospitalisation.
- Results in persistent or significant disability/incapacity.
- Is otherwise medically significant (e.g. important medical events that may not be immediately life-threatening or result in death or hospitalisation but may jeopardise the patient or may require intervention to prevent one of the other outcomes listed above).

14.2 Reporting of Serious Adverse Reactions (SARs)

As this is an observational cohort study where patients follow their normal clinical pathways, and the study introduces a procedural intervention (blood sampling) the PI, or other delegated site investigator should monitor each participant at each visit and only report to UCL CTC events that are serious and related (i.e. a SAR) to the study procedure.

All SARs that occur between the between the start of the first study procedure and 7 days post the last study procedure must be submitted electronically within **24 hours** of observing or notification/occurrence of the event, using the study specific SAR Report.

All sections on the SAR Report must be completed. If the SAR report is **not sent within 24 hours to** UCL CTC, the circumstances that led to this must be detailed in the SAR Report to avoid unnecessary queries.

Causality

The PI, or other delegated site investigator, must perform an evaluation of causality for each event.

- Related (reasonable possibility) to a study procedure.
- Not related (no reasonable possibility) to a study procedure.

Severity

Severity of each event must be determined by using the Common Terminology Criteria for Adverse Events (CTCAE) v5 as a guideline, wherever possible. The criteria are available online at:

https://ctep.cancer.gov/protocoldevelopment/electronic_applications/docs/CTCAE_v5_Quick_Reference_5x7.pdf

Completed SAR Reports must be entered on to the electronic database within 24 hours of becoming aware of the event

[Back-up option: Fax number 020 7679 9871]

SAR Follow-Up Reports

All SARs must be followed-up until resolution and until there are no further queries.

Sites must ensure any new and relevant information is provided promptly. If the reaction term changes or a new reaction is added, the causality must be re-assessed by an Investigator.

SAR Processing at UCL CTC

On receipt of the SAR Report, UCL CTC will check for legibility, completeness, accuracy and consistency. There are no expected serious adverse reactions for the study, therefore all SARs will be considered Related and Unexpected Serious Adverse Reactions.

The CI, or their delegate (e.g. a clinical member of the SMG), may be contacted to review the SAR and to perform an evaluation of causality on behalf of UCL CTC.

Related and Unexpected Serious Adverse Reaction

If the event is evaluated as a related and unexpected Serious Adverse Reaction, UCL CTC will submit a report to the REC within the required timeline.

Wherever possible, evaluations of causal relationship by both the site and the Sponsor’s clinical reviewer will be reported.

15 INCIDENT REPORTING AND SERIOUS BREACHES

15.1 Incident Reporting

Organisations must notify UCL CTC of all deviations from the protocol or GCP immediately. An incident report may be requested and will be provided, but an equivalent document (e.g. Trust Incident form) is acceptable where already completed.

If site staff are unsure whether a certain occurrence constitutes a deviation from the protocol or GCP, the UCL CTC trial team can be contacted immediately to discuss.

UCL CTC will use an organisation's history of non-compliance to make decisions on future collaborations.

UCL CTC will assess all incidents to see if they meet the definition of a serious breach.

15.2 Serious Breaches

A "serious breach" is defined as a breach of the protocol or of the conditions or principles of Good Clinical Practice (or equivalent standards for conduct of non-CTIMPs) which is likely to affect to a significant degree the safety or physical or mental integrity of the trial subjects, or the scientific value of the research.

Systematic or persistent non-compliance by a site with the principles of GCP and/or the protocol, occurring on study within the specified timeframe, may be deemed a serious breach.

In cases where a serious breach has been identified, UCL CTC will inform the REC within 7 calendar days of becoming aware of the breach.

16 STUDY MONITORING AND OVERSIGHT

Participating sites and PIs must agree to allow study-related on-site monitoring, Sponsor audits and regulatory inspections by providing direct access to source data/documents as required. Patients are informed of this in the PIS and are asked to consent to their medical notes being reviewed by appropriate individuals on the consent form.

UCL CTC will determine the appropriate level and nature of monitoring required for the study. Risk will be assessed on an ongoing basis and adjustments made accordingly.

16.1 Central Monitoring

Sites will be requested to submit Study specific logs and staff delegation logs to UCL CTC at the frequency detailed in the study monitoring plan, or on request, and these will be checked for consistency and completeness. Also refer to section 4.4.2(Required documentation).

Data received at UCL CTC will be subject to review in accordance with the data queries section, 13.5.

Sites will be requested to conduct quality control checks of documentation held within the Investigator Site File at the frequency detailed in the study monitoring plan. Checklists detailing the current version/date of version-controlled documents will be provided for this purpose.

Where central monitoring of data and/or documentation submitted by sites indicates that a patient may have been placed at risk the matter will be raised urgently with site staff and escalated as appropriate (refer to section 16 (Incident Reporting and Serious Breaches

16.2 'For Cause' On-Site Monitoring

On-site monitoring visits may be scheduled where there is evidence or suspicion of non-compliance at a site with important aspect(s) of the study protocol/GCP requirements. Sites will be sent a letter in advance outlining the reason(s) for the visit and confirming when it will take place. The letter will include a list of the documents that are to be reviewed, interviews that will be conducted, planned inspections of the facilities and who will be performing the visit.

Following a monitoring visit, the Trial Monitor/Trial Coordinator will provide a follow up email to the site, which will summarise the documents reviewed and a statement of findings, incidents, deficiencies, conclusions, actions taken and/or actions required. The PI at each site will be responsible for ensuring that monitoring findings are addressed in a timely manner, and by the deadline specified.

UCL CTC will assess whether it is appropriate for the site to continue participation in the study and whether the incident(s) constitute a serious breach. Refer to section 15 (Incident Reporting and Serious Breaches

16.3 Oversight Committees

16.3.1 Trial Management Group (TMG)

The TMG will include the Chief Investigator, clinicians and experts from relevant specialties and PARADIGM trial staff from UCL CTC (see page 2). The TMG will be responsible for overseeing the study. The group will meet regularly (approximately 2-3 times a year) and will send updates to PIs (via newsletters or at Investigator meetings) and to the National Cancer Research Institute (NCRI) Prostate Cancer name Clinical Studies Group.

The TMG will review substantial amendments to the protocol prior to submission to the REC . All PIs will be kept informed of substantial amendments through their nominated responsible individual and are responsible for their prompt implementation.

All TMG members will be required to sign the PARADIGM TMG charter and to declare all potential conflicts of interest.

Full responsibilities of the TMG will be detailed in the TMG charter and will include:

- Oversee and take responsibility for the conduct of the study according to the study protocol.
- Review number of patients with pre-ADT samples.
- Review proportion of recruited patients who are be eligible for primary analysis.
- Review of number of patients allocated to PARADIGM-D versus PARADIGM-A.
- Feedback of clinically relevant germline and other molecular variants.

16.3.2 Translational Research Committee

The role of the TRC will be to oversee the analysis of plasma DNA for the primary endpoint and the translational sub-studies. The committee will include members with specific expertise related to this work and will report to the TMG with results that are of interest.

All members will be required to sign a charter and declare all potential conflicts of interest.

16.3.3 Trial Steering Committee (TSC)

The role of the TSC is to provide overall supervision of the study. The TSC will review the recommendations of the Independent Data Monitoring Committee and, on consideration of this information, recommend any appropriate amendments/actions for the study as necessary. The TSC acts on behalf of the funders and the Sponsor.

All TSC will be required to sign the TSC charter and to declare all potential conflicts of interest.

16.3.4 Role of UCL CTC

UCL CTC will be responsible for the day to day coordination and management of the study and will act as custodian of the data generated in the study (on behalf of UCL). UCL CTC is responsible for all duties relating to safety reporting (which are conducted in accordance with section 14 (Safety Reporting).

17 STUDY CLOSURE

17.1 End of Trial

For regulatory purposes the end of the trial will be 5 years after the first patient has been registered, or once all patients have died, whichever is sooner. At this point the 'declaration of end of trial' form will be submitted to and Ethics Committee, as required.

Following this, UCL CTC will advise sites on the procedure for closing the study at the site.

Once the end of trial has been declared, no more prospective patient data will be collected but sites must co-operate with any data queries regarding existing data to allow for analysis and publication of results.

17.2 Archiving of Trial Documentation

At the end of the trial, UCL CTC will archive securely all centrally held study related documentation for a minimum of 5 years. Arrangements for confidential destruction will then be made. It is the responsibility of PIs to ensure data and all essential documents relating to the study held at site are retained securely for a minimum of 5 years after the end of the trial, and in accordance with national legislation.

Essential documents are those which enable both the conduct of the study and the quality of the data produced to be evaluated and show whether the site complied with the principles of GCP and all applicable regulatory requirements.

UCL CTC will notify sites when study documentation held at sites may be archived. All archived documents must continue to be available for inspection by appropriate authorities upon request.

17.3 Early Discontinuation of Trial

The trial may be stopped before completion on the recommendation of the TSC (see section 16.3.3 Trial Steering Committee (TSC). Sites will be informed in writing by UCL CTC of reasons for early closure and the actions to be taken with regards the treatment and follow up of patients.

17.4 Withdrawal from Trial Participation by a Site

Should a site choose to close to recruitment the PI must inform UCL CTC in writing. Follow up as per protocol must continue for any patients recruited into the trial at that site and other responsibilities continue as per the CTSA.

18 ETHICAL CONSIDERATIONS

In conducting the study, the Sponsor, UCL CTC and sites shall comply with all relevant guidance, laws and statutes, as amended from time to time, applicable to the performance of clinical trials including, but not limited to:

- The principles of Good Clinical Practice
- Human Rights Act 1998
- Data Protection Act 2018 , and General Data Protection Regulation (EU)2016/679 (GDPR)
- Freedom of Information Act 2000
- Mental Capacity Act 2005
- UK Policy Framework for Health and Social Care Research, issued by the Health Research Authority

18.1 Ethical Approval

The trial will be conducted in accordance with the World Medical Association Declaration of Helsinki entitled ‘Ethical Principles for Medical Research Involving Human Subjects’ (1996 version) and in accordance with the terms and conditions of the ethical approval given to the trial.

The trial has received a favorable opinion from the London-Brighton and Sussex Research Ethics Committee (REC) and Health Research Authority (HRA) approval for conduct in the UK.

UCL CTC will submit Annual Progress Reports to the REC, commencing one year from the date of ethical approval for the trial.

18.2 Site Approvals

Evidence of assessment of capability and capacity by the Trust/Health Board R&D for a trial site must be provided to UCL CTC. Sites will only be activated when all necessary local approvals for the trial have been obtained.

18.3 Protocol Amendments

UCL CTC will be responsible for gaining ethical for amendments made to the protocol and other study-related documents. Once approved, UCL CTC will ensure that all amended documents are distributed to sites as appropriate.

Site staff will be responsible for acknowledging receipt of documents and for implementing all amendments promptly.

18.4 Patient Confidentiality & Data Protection

Patient identifiable data, including full name/initials, , and telephone number will be collected by UCL CTC. UCL CTC will preserve patient confidentiality and will not disclose or reproduce any information by which patients could be identified.

Data will be stored in a secure manner and UCL CTC trials are registered in accordance with the Data Protection Act 2018 and GDPR, with the Data Protection Officer at UCL

Patient identifiable data, including initials will be provided to the UCL Cancer Institute and EPIC Sciences, Inc. in order to process the samples. Both Laboratories will preserve patient confidentiality and will not disclose or reproduce any information by which patients could be identified.

19 SPONSORSHIP AND INDEMNITY

19.1 Sponsor Details

Sponsor Name:	UCL
Address:	Joint Research Office
	Gower Street
	London
	WC1E 6BT
Contact:	Director of Research Support
Tel:	020 3447 9995/2178 (unit admin)
Fax:	020 3447 9937

19.2 Indemnity

UCL holds insurance against claims from participants for injury caused by their participation in the clinical trial/study. Participants may be able to claim compensation if they can prove that UCL has been negligent. However, as this clinical trial/study is being carried out in a hospital, the hospital continues to have a duty of care to the participant of the clinical trial/study. UCL does not accept liability for any breach in the hospital's duty of care, or any negligence on the part of hospital employees. This applies whether the hospital is an NHS Trust or otherwise.

Participants may also be able to claim compensation for injury caused by participation in this clinical trial/study without the need to prove negligence on the part of UCL or another party. Participants who sustain injury and wish to make claim for compensation should be advised to do so in writing in the first instance to the Chief Investigator, who will pass the claim to the Sponsor's Insurers, via the Sponsor's office."

Hospitals selected to participate in this clinical trial/study shall provide clinical negligence insurance cover for harm caused by their employees and a copy of the relevant insurance policy or summary shall be provided to University College London, upon request."

20 PUBLICATION POLICY

All publications and presentations relating to the study should be authorised by the TMG. The TMG will form the basis of the writing committee and advise on the nature of the publications. All collaborators who have actively contributed to the study will be named authors on all main study papers and anyone else who has had a significant input into the conduct, analysis and interpretation of the study.

Specialist papers focusing on a particular aspect of translational research may not require all collaborators to be authors. Data from all sites will be analysed together and published as soon as possible after the primary endpoint has been reached. Participating sites may not publish study results prior to the first publication by the TMG or without prior written consent from the TMG.

The Chief Investigator will make the final decision on authorship. The study data is owned by the Sponsor. The ClinicalTrials.gov number should be quoted in any publications resulting from this study.

21 REFERENCES

1. Uk CRUK, Cancer R. Prostate cancer mortality statistics. 2014.
2. James ND, Spears MR, Clarke NW, et al. Survival with Newly Diagnosed Metastatic Prostate Cancer in the "Docetaxel Era": Data from 917 Patients in the Control Arm of the STAMPEDE Trial (MRC PR08, CRUK/06/019). *European urology.* 2015;67(6):1028-1038.
3. Fizazi K, Faivre L, Lesaunier F, et al. Androgen deprivation therapy plus docetaxel and estramustine versus androgen deprivation therapy alone for high-risk localised prostate cancer (GETUG 12): a phase 3 randomised controlled trial. *The Lancet Oncology.* 2015;16(7):787-794.
4. Fizazi K, Lesaunier F, Delva R, et al. A phase III trial of docetaxel-estramustine in high-risk localised prostate cancer: a planned analysis of response, toxicity and quality of life in the GETUG 12 trial. *European journal of cancer.* 2012;48(2):209-217.
5. James ND, Sydes MR, Clarke NW, et al. Addition of docetaxel, zoledronic acid, or both to first-line long-term hormone therapy in prostate cancer (STAMPEDE): survival results from an adaptive, multiarm, multistage, platform randomised controlled trial. *The Lancet.* 2015.
6. Vale CL, Burdett S, Rydzewska LHM, et al. Addition of docetaxel or bisphosphonates to standard of care in men with localised or metastatic, hormone-sensitive prostate cancer: a systematic review and meta-analyses of aggregate data. *The Lancet Oncology.* 2016;17(2):243-256.
7. Sweeney CJ, Chen YH, Carducci M, et al. Chemohormonal Therapy in Metastatic Hormone-Sensitive Prostate Cancer. *The New England journal of medicine.* 2015;373(8):737-746.
8. Fizazi K, Tran N, Fein L, et al. Abiraterone plus Prednisone in Metastatic, Castration-Sensitive Prostate Cancer. *The New England journal of medicine.* 2017;377(4):352-360.
9. James ND, de Bono JS, Spears MR, et al. Abiraterone for Prostate Cancer Not Previously Treated with Hormone Therapy. *The New England journal of medicine.* 2017;377(4):338-351.
10. Kyriakopoulos CE, Chen Y-H, Carducci MA, et al. Chemohormonal Therapy in Metastatic Hormone-Sensitive Prostate Cancer: Long-Term Survival Analysis of the Randomized Phase III E3805 CHAARTED Trial. *Journal of Clinical Oncology.*0(0):JCO.2017.2075.3657.
11. OncologyPro. Effects of Abiraterone Acetate plus Prednisone/Prednisolone in High and Low Risk Metastatic Hormone Sensitive Prostate Cancer | OncologyPRO. 2018; https://oncologypro.esmo.org/Meeting-Resources/ESMO-2018-Congress/Effects-of-Abiraterone-Acetate-plus-Prednisone-Prednisolone-in-High-and-Low-Risk-Metastatic-Hormone-Sensitive-Prostate-Cancer.
12. Dawson SJ, Tsui DW, Murtaza M, et al. Analysis of circulating tumor DNA to monitor metastatic breast cancer. *The New England journal of medicine.* 2013;368(13):1199-1209.
13. Bettegowda C, Sausen M, Leary RJ, et al. Detection of circulating tumor DNA in early- and late-stage human malignancies. *Science translational medicine.* 2014;6(224):224ra224.
14. Siravegna G, Mussolin B, Buscarino M, et al. Clonal evolution and resistance to EGFR blockade in the blood of colorectal cancer patients. *Nature medicine.* 2015;21(7):827.
15. Diehl F, Li M, Dressman D, et al. Detection and quantification of mutations in the plasma of patients with colorectal tumors. *Proceedings of the National Academy of Sciences of the United States of America.* 2005;102(45):16368-16373.
16. Diehl F, Schmidt K, Choti MA, et al. Circulating mutant DNA to assess tumor dynamics. *Nature medicine.* 2008;14(9):985-990.
17. Lo YMD, Chan KCA, Sun H, et al. Maternal Plasma DNA Sequencing Reveals the Genome-Wide Genetic and Mutational Profile of the Fetus. *Science translational medicine.* 2010;2(61):61ra91-61ra91.
18. Forshew T, Murtaza M, Parkinson C, et al. Noninvasive Identification and Monitoring of Cancer Mutations by Targeted Deep Sequencing of Plasma DNA. *Science translational medicine.* 2012;4(136):136ra168-136ra168.
19. Diehl F, Schmidt K, Choti MA, et al. Circulating mutant DNA to assess tumor dynamics. *Nature medicine.* 2008;14(9):985-990.

20. Abbosh C, Birkbak NJ, Wilson GA, et al. Phylogenetic ctDNA analysis depicts early-stage lung cancer evolution. *Nature.* 2017;545(7655):446-451.
21. Tie J, Wang Y, Tomasetti C, et al. Circulating tumor DNA analysis detects minimal residual disease and predicts recurrence in patients with stage II colon cancer. *Science translational medicine.* 2016;8(346):346ra392-346ra392.
22. Gevensleben H, Garcia-Murillas I Fau - Graeser MK, Graeser Mk Fau - Schiavon G, et al. Noninvasive detection of HER2 amplification with plasma DNA digital PCR. (1078-0432 (Print)).
23. Annala M, Struss WJ, Warner EW, et al. Treatment Outcomes and Tumor Loss of Heterozygosity in Germline DNA Repair-deficient Prostate Cancer. *European urology.* 2017;72(1):34-42.
24. Annala M, Vandekerkhove G, Khalaf D, et al. Circulating tumor DNA genomics correlate with resistance to abiraterone and enzalutamide in prostate cancer. *Cancer discovery.* 2018.
25. Wyatt AW, Azad AA, Volik SV, et al. Genomic Alterations in Cell-Free DNA and Enzalutamide Resistance in Castration-Resistant Prostate Cancer. (2374-2445 (Electronic)).
26. Romanel A, Tandefelt DG, Conteduca V, et al. Plasma AR and abiraterone-resistant prostate cancer. *Science translational medicine.* 2015;7(312):312re310-312re310.
27. Chi KN, Kheoh T, Ryan CJ, et al. A prognostic index model for predicting overall survival in patients with metastatic castration-resistant prostate cancer treated with abiraterone acetate after docetaxel. *Annals of Oncology.* 2015.
28. <Ann Oncol-2015-Chi-annonc_mdv594.pdf>.
29. Struss WJ, Vandekerkhove G, Annala M, Chi KN, Gleave ME, Wyatt A. 796PDDetection of circulating tumor DNA in de novo metastatic castrate sensitive prostate cancer. *Annals of Oncology.* 2018;29(suppl_8):mdy284.005-mdy284.005.
30. Goodall J, Mateo J, Yuan W, et al. Circulating Cell-Free DNA to Guide Prostate Cancer Treatment with PARP Inhibition. *Cancer discovery.* 2017;7(9):1006-1017.
31. Carreira S, Romanel A, Goodall J, et al. Tumor clone dynamics in lethal prostate cancer. *Science translational medicine.* 2014;6(254):254ra125.
32. Quigley D, Alumkal JJ, Wyatt AW, et al. Analysis of Circulating Cell-Free DNA Identifies Multiclonal Heterogeneity of BRCA2 Reversion Mutations Associated with Resistance to PARP Inhibitors. *Cancer discovery.* 2017;7(9):999-1005.
33. Conteduca V, Wetterskog D, Sharabiani MTA, et al. Androgen receptor gene status in plasma DNA associates with worse outcome on enzalutamide or abiraterone for castration-resistant prostate cancer: a multi-institution correlative biomarker study. *Annals of oncology : official journal of the European Society for Medical Oncology / ESMO.* 2017;28(7):1508-1516.
34. Jayaram A, Wingate A, Sharabiani MTA, et al. Clinical qualification of plasma androgen receptor (pAR) status and outcome on abiraterone acetate (AA) plus prednisone or dexamethasone (+P/D) in a phase II multi-institutional study in metastatic castration resistant prostate cancer (mCRPC). 2018;36(15_suppl):5067-5067.
35. Newman AM, Bratman SV, To J, et al. An ultrasensitive method for quantitating circulating tumor DNA with broad patient coverage. *Nature Medicine.* 2014;20(5):552-558.
36. Lanman RB, Mortimer SA, Zill OA, et al. Analytical and Clinical Validation of a Digital Sequencing Panel for Quantitative, Highly Accurate Evaluation of Cell-Free Circulating Tumor DNA. *PLoS One.* 2015;10(10):e0140712.
37. Phallen J, Sausen M, Adleff V, et al. Direct detection of early-stage cancers using circulating tumor DNA. *Sci Transl Med.* 2017;9(403).
38. Mansukhani S, Barber LJ, Kleftogiannis D, et al. Ultra-Sensitive Mutation Detection and Genome-Wide DNA Copy Number Reconstruction by Error-Corrected Circulating Tumor DNA Sequencing. *Clin Chem.* 2018.
39. Sun K, Jiang P, Chan KC, et al. Plasma DNA tissue mapping by genome-wide methylation sequencing for noninvasive prenatal, cancer, and transplantation assessments. *Proc Natl Acad Sci U S A.* 2015;112(40):E5503-5512.

40. Boeve LMS, Hulshof M, Vis AN, et al. Effect on Survival of Androgen Deprivation Therapy Alone Compared to Androgen Deprivation Therapy Combined with Concurrent Radiation Therapy to the Prostate in Patients with Primary Bone Metastatic Prostate Cancer in a Prospective Randomised Clinical Trial: Data from the HORRAD Trial. *European urology.* 2018.
41. Parker CC, James ND, Brawley CD, et al. Radiotherapy to the primary tumour for newly diagnosed, metastatic prostate cancer (STAMPEDE): a randomised controlled phase 3 trial. *The Lancet.* 2018;392(10162):2353-2366.
42. Sydes MR, Spears MR, Mason MD, et al. Adding abiraterone or docetaxel to long-term hormone therapy for prostate cancer: directly randomised data from the STAMPEDE multi-arm, multi-stage platform protocol. *Annals of Oncology.* 2018:mdy072-mdy072.
43. Harshman LC, Chen Y-H, Liu G, et al. Seven-Month Prostate-Specific Antigen Is Prognostic in Metastatic Hormone-Sensitive Prostate Cancer Treated With Androgen Deprivation With or Without Docetaxel. *Journal of Clinical Oncology.* 2018;36(4):376-382.
44. Heller G, McCormack R, Kheoh T, et al. Circulating Tumor Cell Number as a Response Measure of Prolonged Survival for Metastatic Castration-Resistant Prostate Cancer: A Comparison With Prostate-Specific Antigen Across Five Randomized Phase III Clinical Trials. *Journal of Clinical Oncology.* 2017;36(6):572-580.
45. Mehra N, Seed G, Lambros M, et al. Myeloid-derived suppressor cells (MDSCs) in metastatic castration-resistant prostate cancer (CRPC) patients (PTS). *Annals of Oncology.* 2016;27(suppl_6):757P-757P.
46. Di Mitri D, Toso A, Chen JJ, et al. Tumour-infiltrating Gr-1+ myeloid cells antagonize senescence in cancer. *Nature.* 2014;515(7525):134-137.
47. Calcinotto A, Spataro C, Zagato E, et al. IL-23 secreted by myeloid cells drives castration-resistant prostate cancer. *Nature.* 2018;559(7714):363-369.
48. Morse MD, McNeel DG. Prostate cancer patients on androgen deprivation therapy develop persistent changes in adaptive immune responses. *Human immunology.* 2010;71(5):496-504.
49. Ross RW, Galsky MD, Scher HI, et al. A whole-blood RNA transcript-based prognostic model in men with castration-resistant prostate cancer: a prospective study. *The Lancet Oncology.* 2012;13(11):1105-1113.
50. Olmos D, Brewer D, Clark J, et al. Prognostic value of blood mRNA expression signatures in castration-resistant prostate cancer: a prospective, two-stage study. *The Lancet Oncology.* 2012;13(11):1114-1124.
51. Dehm SM, Schmidt Lj Fau - Heemers HV, Heemers Hv Fau - Vessella RL, Vessella Rl Fau - Tindall DJ, Tindall DJ. Splicing of a novel androgen receptor exon generates a constitutively active androgen receptor that mediates prostate cancer therapy resistance. (1538-7445 (Electronic)).
52. Antonarakis ES, Lu C, Wang H, et al. AR-V7 and resistance to enzalutamide and abiraterone in prostate cancer. *The New England journal of medicine.* 2014;371(11):1028-1038.
53. Antonarakis ES, Lu C, Luber B, et al. Clinical Significance of Androgen Receptor Splice Variant-7 mRNA Detection in Circulating Tumor Cells of Men With Metastatic Castration-Resistant Prostate Cancer Treated With First- and Second-Line Abiraterone and Enzalutamide. *Journal of Clinical Oncology.* 2017;35(19):2149-2156.
54. Antonarakis ES, Lu C, Luber B, et al. Androgen Receptor Splice Variant 7 and Efficacy of Taxane Chemotherapy in Patients With Metastatic Castration-Resistant Prostate Cancer. *JAMA Oncol.* 2015;1(5):582-591.
55. Scher HI, Lu D, Schreiber NA, et al. Association of AR-V7 on Circulating Tumor Cells as a Treatment-Specific Biomarker With Outcomes and Survival in Castration-Resistant Prostate Cancer. *JAMA Oncol.* 2016;2(11):1441-1449.
56. McDaniel AS, Ferraldeschi R, Krupa R, et al. Phenotypic diversity of circulating tumour cells in patients with metastatic castration-resistant prostate cancer. *BJU Int.* 2017;120(5B):E30-E44.
57. Scher HI, Graf RP, Schreiber NA, et al. Phenotypic Heterogeneity of Circulating Tumor Cells Informs Clinical Decisions between AR Signaling Inhibitors and Taxanes in Metastatic Prostate Cancer. *Cancer research.* 2017;77(20):5687.
58. Punnoose EA, Ferraldeschi R, Szafer-Glusman E, et al. PTEN loss in circulating tumour cells correlates with PTEN loss in fresh tumour tissue from castration-resistant prostate cancer patients. *British journal of cancer.* 2015;113(8):1225-1233.

59. Scher HI, Graf RP, Schreiber NA, et al. Nuclear-specific AR-V7 Protein Localization is Necessary to Guide Treatment Selection in Metastatic Castration-resistant Prostate Cancer. *European urology.* 2017;71(6):874-882.
60. Miyamoto DT, Lee RJ, Stott SL, et al. Androgen receptor signaling in circulating tumor cells as a marker of hormonally responsive prostate cancer. *Cancer discovery.* 2012;2(11):995-1003.
61. Padhani AR, Lecouvet FE, Tunariu N, et al. Rationale for Modernising Imaging in Advanced Prostate Cancer. *European Urology Focus.* 2017;3(2):223-239.
62. Sartor O, de Bono JS. Metastatic Prostate Cancer. *New England Journal of Medicine.* 2018;378(7):645-657.
63. Briganti A, Abdollah F, Nini A, et al. Performance Characteristics of Computed Tomography in Detecting Lymph Node Metastases in Contemporary Patients with Prostate Cancer Treated with Extended Pelvic Lymph Node Dissection. *European urology.* 2012;61(6):1132-1138.
64. Talab SS, Preston Ma Fau - Elmi A, Elmi A Fau - Tabatabaei S, Tabatabaei S. Prostate cancer imaging: what the urologist wants to know. (1557-8275 (Electronic)).
65. Messiou C, Cook G Fau - deSouza NM, deSouza NM. Imaging metastatic bone disease from carcinoma of the prostate. (1532-1827 (Electronic)).
66. Gundem G, Van Loo P, Kremeyer B, et al. The evolutionary history of lethal metastatic prostate cancer. *Nature.* 2015;520(7547):353-357.
67. Matsumoto M, Koike S, Kashima S, Awai K. Geographic Distribution of CT, MRI and PET Devices in Japan: A Longitudinal Analysis Based on National Census Data. *PloS one.* 2015;10(5):e0126036.
68. Schmidt GP, Schoenberg So Fau - Schmid R, Schmid R Fau - Stahl R, et al. Screening for bone metastases: whole-body MRI using a 32-channel system versus dual-modality PET-CT. (0938-7994 (Print)).
69. Schmidt GP, Baur-Melnyk A, Haug A, et al. Comprehensive imaging of tumor recurrence in breast cancer patients using whole-body MRI at 1.5 and 3 T compared to FDG-PET-CT. *Clinical Imaging.* 2008;32(4):330.
70. Lecouvet FE, El Mouedden J Fau - Collette L, Collette L Fau - Coche E, et al. Can whole-body magnetic resonance imaging with diffusion-weighted imaging replace Tc 99m bone scanning and computed tomography for single-step detection of metastases in patients with high-risk prostate cancer? (1873-7560 (Electronic)).
71. Latifoltojar A, Hall-Craggs M, Bainbridge A, et al. Whole-body MRI quantitative biomarkers are associated significantly with treatment response in patients with newly diagnosed symptomatic multiple myeloma following bortezomib induction. (1432-1084 (Electronic)).
72. UK CR. Prognostic/predictive Biomarker Roadmap https://www.cancerresearchuk.org/sites/default/files/prognostic_and_predictive.pdf.

APPENDIX 1: ABBREVIATIONS

**AAP**	Abiraterone acetate with prednisolone/prednisone
**ALP**	Alkaline Phosphatase
**AR**	Androgen receptor
**ADT**	Androgen Deprivation Therapy
**BP**	Base pairs
**CRF**	Case Report Form
**CEA**	Carcinoembryonic antigen
**CRPC**	Castrate resistant prostate cancer
**ctDNA**	Circulating tumour DNA
**CTC**	Circulating Tumour Cells
**CTP**	Clinical Trial Practioner
**CT**	Computerised Tomography
**CTCAE**	Common Terminology Criteria for Adverse Events
**CN**	Copy Number
**CXCR2**	CXC chemokine receptor 2
**Ctla-4**	Cytotoxic T lymphocyte antigen-4
**Doc**	Docetaxel with or without prednisolone/prednisone
**DFS**	Disease Free Survival
**DPA**	Data Protection Act
**ECOG**	Eastern Cooperative Oncology Group
**EDTA**	Ethylenediaminetetraacetic acid
**eCRF**	Electronic case report forms
**ELISA**	Enzyme-linked immunosorbent assay
**EPCAM**	Epithelial Cell Adhesion Molecule
**ESMO**	European Society of Medical oncology
**FFPE**	Formaldehyde Fixed-Paraffin Embedded
**GCP**	Good clinical practice

**GDPR**	General Data Protection Regulation
**GNRH**	Gonadotropin-releasing hormone
**HR**	Hazard ratio
**HRA**	Health Research Authority
**ICH GCP**	International Conference of Harmonisation-Good Clinical Practice
**IQR**	Interquartile range
**ISF**	Investigator site file
**LDH**	Lactate Dehydrogenase
**LH**	Luteinizing hormone
**LHRHa**	Luteinizing hormone-releasing hormone agonist/ antagonist
**mCRPC**	Metastatic castration-resistant prostate cancer
**MDSCs**	Myeloid-Derived Suppressor Cells
**M-MDSC**	Monocytic myeloid-derived suppressor cells
**mNCA**	Model clinical trial agreement
**MRD**	Minimal residual disease
**NK**	Natural Killer
**NGS**	Next generation Sequencing
**OS**	Overall Survival
**PET**	Positron emission tomography
**PFS**	Progression Free Survival
**PI**	Principal Investigator
**PIS**	Patient information sheet
**REC**	Research Ethics Committee
**RECIST**	Response Evaluation Criteria in Solid Tumours
**rPFS**	Radiographic Progression free survival
**SAR**	Serious Adverse Reaction
**SOC**	Standard of care
**SNVS**	Single nucleotide variants

**TAMs**	Tumour associated macrophages
**TCR**	T cell receptor
**TMF**	Trial Master File
**UCL CTC**	CR UK and UCL Cancer Trials Centre
**WB-DW-MRI**	Whole body diffusion weighted magnetic resonance imaging
**WBMRI**	Whole body Magnetic Resonance Image

APPENDIX 2: SCHEDULE OF ASSESSMENTS

Assessment	Registration to main study	Pre- ADT	C1D1	C2 D1	C3D1	C4D1	C5D1	C6D1	At completion of 6 cycles	Every 3-6 months	At disease progression	After Disease Progression
**Standard of Care Assessments**												
**Informed consent**	X											
**CT chest, abdomen and pelvis**[1]	X					X[11]			X	X[12]	X	
**Whole body technetium labelled bone scan** [1]	X					X[11]			X	X[12]	X	
**Histological confirmation of prostate carcinoma**	X											
**Relevant medical history**	X											
**Review ongoing medication** [2]	X											
**ECOG Performance Score** [2]	X											
**Serum PSA**	X[3]		X	X	X	X	X	X	X	X	X	
**Serum testosterone**			X[10]						X			
**Serum LDH** (if physicians considers relevant)			X		X		X	X	X			
**Serum ALP** (if physicians considers relevant)			X		X		X	X	X			
**Full blood count, including differential**			X									
**Serum creatinine**			X									
**Height and weight**			X									
**Survival follow-up**												X
**PARADIGM-Specific Assessments**												
**Plasma (ptDNA) 4 x 10 ml**[4]		X[6]	X	X	X		X	X	X	X	X[13]	
**Whole blood (immunoprofiling) 1 x 10 ml immunoprofiling tubes**[4]		X[6]	X[9]		X[9]		X[9]				X[13]	
**Whole Blood (CTCs) 1 x 10 ml**[4]		X[6]	X[9]		X[9]							
**PAXGene RNA 1 x 2.5 ml**[4]		X[6]										
**WB-MRI**[8]		X[7,8]			X[7, 11]							
**Retrieve archival tumour blocks**	X[5]											

Notes

1. Alternative imaging is permitted after discussion with UCL CTC
2. Within 30 days of registration
3. Prior to ADT
4. All samples can be collected up to 72 hours prior to pre-specified time points
5. Archival tumour blocks to be shipped as soon as possible after entering patient onto trial
6. For a subset of patients (~50) and can be collected after consent to the pre-study consent form
7. Subset of patients who have consented on MRI consent form at UCLH and selected centres
8. Ideally within 4 weeks ADT and before starting Abi/Doc
9. Only patients who had the pre-ADT blood sample
10. Confirming castration and as close as possible to collection of pre Abi/Doc research blood sample
11. +/- 2 weeks
12. Recommended to be repeated at 24 weeks and whenever clinically appropriate
13. Progression samples missed at the time of progression can be collected when new treatment is commenced

APPENDIX 3: PROTOCOL VERSION HISTORY

**Protocol**		**Amendments:**		
Version no.	Date	Amendment no.	Protocol Section (no./title)	Summary of main changes from previous version.
1	14.01.2019	N/A	N/A	N/A

APPENDIX 4: PROTOCOL VERSION HISTORY

Protocol		Amendments:		
Version no.	Date	Amendment no.	Protocol Section (no./title)	Summary of main changes from previous version.
1	14.01.2019	N/A	N/A	N/A
2	20.12.2019	4.0	General	Administrative changes, including correction of typographical and grammatical changes.
2	20.12.2019	4.0	General	Update to TMG membership.
2	20.12.2019	4.0	General	Change of wording from eCRF to EDC.
2	20.12.2019	4.0	General	Translational research wording changed to tracking of plasma DNA dynamics and extracellular vesicles.
2	20.12.2019	4.0	General	Secondary objectives changed in line with inclusion criteria.
2	20.12.2019	4.0	1.1	Addition of Clinical trials.gov number.
2	20.12.2019	4.0	1.1 & 6.2	Modifications to inclusion and exclusion criteria- Changes made to the definition of polymetastatic disease,<br>extending the time limits of starting LHRA agonist or antagonist, extending the time limits to start docetaxel and abiraterone after stating LHRA antagonist or agonist, patients must be 18 and over, removal of medically unsuitable for either abiraterone, prednisolone or docetaxel as this is already mentioned in the inclusion criteria.
2	20.12.2019	4.0	2.3	Addition of background to nomenclature and categorisation related to disease metastatic status.
2	20.12.2019	4.0	2.7	Clarification of wording to WB DW MRI for hormone sensitive metastatic prostate cancer.
2	20.12.2019	4.0	4.1	Addition of definition of serious breach.
2	20.12.2019	4.0	4.1	Update to wording- addition of ‘Framework for Health and Social Care’.
2	20.12.2019	4.0	4.2.3	Site activation can be via letter or email
2	20.12.2019	4.0	5.0	Updated number of patient information sheets.
2	20.12.2019	4.0	7.1	Addition of data collected about pre-study consent.
2	20.12.2019	4.0	7.2	Clarification regarding registration to main study procedure.
2	20.12.2019	4.0	8.6	Timepoint and Samples to be taken updated
2	20.12.2019	4.0	8.6.1	Clarification of patient directed sample collection.
2	20.12.2019	4.0	8.7.1	Removal of lab processing instructions.
2	20.12.2019	4.0	8.7.1	Name of central laboratory changed.
2	20.12.2019	4.0	8.9	Addition of central review of imaging.
2	20.12.2019	4.0	9.0	Addition of information about WB-MRI timelines, site qualification process, transferring of WB-MRI data and WB-MRI results.

2	20.12.2019	4.0	10.4	Addition of pre-study patient withdrawal of consent.
2	20.12.2019	4.0	11.0	Clarification that data and assessments collected must be standard of care unless these are research blood samples or research tissue samples.
2	20.12.2019	4.0	11.0	Addition of immunoprofiling tube
2	20.12.2019	4.0	11.1	Collection of data for patients who have registered onto pre-study.
2	20.12.2019	4.0	11.2	Collection of self-reported race.
2	20.12.2019	4.0	12.5	Addition of 1.5T scanner used for WB-MRI imaging
2	20.12.2019	4.0	13.2	Modification of secondary endpoints in line with changes to inclusion criteria.
2	20.12.2019	4.0	15.1	Changes in definition of SAR.
2	20.12.2019	4.0	15.2	Addition of SARS submitted via email and fax. Removal of SARS submitted electronically.
2	20.12.2019	4.0	17.1	Details of study specific logs added- Biological sample logs and PI GCP and CV.
2	20.12.2019	4.0	17.2.2	Explanation of role or TRC.
2	20.12.2019	4.0	19.4	Collection of patient identifiable data for central review of imaging and how data for central imaging will be transferred and stored.
2	20.12.2019	4.0	Appendix 2.0	Gleason score and self-reported race data collected.
2	20.12.2019	4.0	Appendix 2.0	Testosterone and PSA data can be collected if performed as per SOC.
2	20.12.2019	4.0	Appendix 2.0	Follow up research samples can be collected before 72 hours.
3	26.05.2020	7.0	General	Addition of PCUK movember logo.
3	26.05.2020	7.0	General	Update to TMG membership.
3	26.05.2020	7.0	General	Throughout-Abiraterone has been replaced by Androgen receptor signalling inhibitors (ARSI) as applicable.
3	26.05.2020	7.0	General	Throughout - C3D1 collection for primary analysis modified to C3D1 or C4D1 as applicable.
3	26.05.2020	7.0	General	Sample collection at C2D1, C3D1 and C5D1 have been revised to every cycle.
3	26.05.2020	7.0	1.1 ,6.2.1 & 6.2.2	Clarification of wording in inclusion criterion 7, to include patients planning to start LHRH or having LHRH treatment.<br><br>Pre-ADT and LHRHa treatment timelines defined.<br><br>Previous inclusion criterion 10 is now criterion 2.<br><br>Rewording of exclusion criterion 1 ‘Concurrent or planned for (within the first 5 cycles of docetaxel or abiraterone…’ to ‘Concurrent or

				planned for (i.e. prior to development of castration resistance)'.<br><br>Exclusion criterion 4 revised to 'Any surgery planned prior to Cycle 4 Day 1'
3	26.05.2020	7.0	1.2	Study summary diagram amended & updated to include reference to ARSI and addition of C4D1.
3	26.05.2020	7.0	2.1	Additional background information provided for Enzalutamide and Apalutamide in PARADIGM-A.
3	26.05.2020	7.0	2.2	Typographical errors corrected and references updated.
3	26.05.2020	7.0	2.3	Removal of ''Approval of abiraterone and funding access in the UK will be restricted to this population''.
3	26.05.2020	7.0	2.4	PARADIGM justification - reference to ARSI instead of Abiraterone - overall justification unchanged.
3	26.05.2020	7.0	3.1.1 & 3.1.2	Primary objective amended to include detection of ptDNA after two, or three cycles.<br><br>Secondary objective amended to include C4D1 sample.
3	26.05.2020	7.0	4.1	Removal of serious breach details (cross reference formatting error).
3	26.05.2020	7.0	5.1	Clarification added that each specific ARSI treatment will have a separate PIS.
3	26.05.2020	7.0	8.2	Addition of anti-androgen use and LHRAa timelines defined.
3	26.05.2020	7.0	8.3	Addition of 'with or without prednisolone' and docetaxel cycles defined.
3	26.05.2020	7.0	8.4	Standard of care abiraterone has been amended to Standard of care ARSI - *footnote added to include NICE funding approval for Enzalutamide treatment option.*
3	26.05.2020	7.0	8.6	Table updated to include collection of samples at C4D1.
3	26.05.2020	7.0	11.1	Removal of Gleason Score for pre-registration of pre-study patients
3	26.05.2020	7.0	11.2	Addition of Gleason Score for pre-registration for Main Study.<br><br>Addition of type of ARSI to be used for patient receiving ARSI treatment.
3	26.05.2020	7.0	11.5	Sample collection at C2D1, C3D1, C5D1 and C6D1 revised to also include sample collection at C4D1.<br><br>Addition of CTC bloods at C4D1 are taken if missed at C3D1.
3	26.05.2020	7.0	12.3	Clarification and justification of CTC sample collection at C4D1 if not taken at C3D1.

3	26.05.2020	7.0	13.1	Statement to clarifying statistics analysis in PARADIGM-A cohort will include patients receiving all ARSI treatments.
3	26.05.2020	7.0	13.2	Definition of patients to be included in primary end-point analysis revised:<br>Point 1 -Delays in treatment extended from 1 to 2 weeks.<br><br>Point 2 wording revised from Received a minimum of two cycles of docetaxel or abiraterone within 9weeks from start of treatment' to 'Received a minimum of two cycles within 8 weeks or three cycles within 11 weeks of docetaxel from start of treatment.<br><br>Addition of point 3 Received at least 7 days continuous and a minimum of 42 days of ARSI prior to C3D1 sample or 63 days prior to C4D1.<br><br>Addition of point 4- Received a minimum of 4 cycles of docetaxel or ARSI until progression.<br><br>Addition of point 7 - Plasma testosterone in the castrate range measured in the central lab on the sample used for ptDNA assessment.<br><br>Amended follow-up procedures for patients who do not meet the criteria of primary end-point analysis.
3	26.05.2020	7.0	13.3	Clarification of how positive ptDNA primary endpoint samples at C3D1 & C4D1 will be analysed.<br><br>Serum PSA progression for PARADIGM-D patients defined.
3	26.05.2020	7.0	13.3.1	Paradigm A and D included in title
3	26.05.2020	7.0	13.3.3	Addition of PARADIGM -D to title.<br><br>Statement has been removed in section 13.3.3 and added to 13.3 'In keeping with clinical practice, serum PSA progression does not constitute a progression event in PARADIGM-A but does in PARADIGM-D.'<br><br>Removal of Methods for PSA calculation.<br><br>Additional wording in text to clarify radiological progression and when C3D1 or C4D1 can be used to define progression.
3	26.05.2020	7.0	15.1	Removal of definition *'Definitions and Standards for Expedited Reporting" and ICH GCP E6'* – text here in error.

3	26.05.2020	7.0	17.1	Removal of text relating to site initiation visits (cross reference formatting error).
3	26.05.2020	7.0	22	References updated.
3	26.05.2020	7.0	Appendix 1.0	Table updated with addition of ARSI and LHRAa
3	26.05.2020	7.0	Appendix 2.0	Corrections made in wording of carcinoma to adenocarcinoma.
3	26.05.2020	7.0	Appendix 2.0	Samples collected at C4D1 added in assessment table.
3	26.05.2020	7.0	Appendix 2.0	Footnote 16 - added to confirm when samples should be collected if treatment is delayed.
3	26.05.2020	7.0	Appendix 2.0	Footnote 17 - conditions of when C4D1 CTC sample collection should be taken is defined.
4	17.07.2020	8.0	1.1 & 6.2	Inclusion criteria point 7 & 8 amended to patients have to have started within 18 weeks of starting antiandrogens with a minimum of 5 weeks of LHRH agonist, instead of 12 weeks of LHRH agonist.
5	22.09.2020	9.0	1.1 & 6.2	Removal of inclusion criterion point 3. Exclusion criteria point 1 has been revised to clarify that patients may be eligible for PARADIGM if randomised to receive an experimental drug or to the placebo arm of an open-label clinical trial after discussion and approval of the CI/delegate.
5	22.09.2020	9.0	1.1 & 6.2.1	Inclusion criterion 3 revised from '≥1 measurable visceral metastasis' to '≥1 unequivocal visceral metastasis.
5	22.09.2020	9.0	1.1,6.2 and 8.2	Removal of minimum time required for LHRH agonist and antagonists.
5	22.09.2020	9.0	4.2.1	Site initiation statement revised to include SIV via teleconference.
5	22.09.2020	9.0	7 & 15.2	Trial team contact details updated.
5	22.09.2020	9.0	8.6	Sample collection window has been increased from 72 hours prior to each cycle to 120 hours (5 days).
5	22.09.2020	9.0	8.8	Statement revised to confirm that consent can be requested if biopsy tissue is available.
5	22.09.2020	9.0	11.2	Recording histological information on the EDC made optional for patient registration.
5	22.09.2020	9.0	11.6	Clarification added that after completion of 6 cycles of treatment is the end of treatment for docetaxel and is C7D1 for ARSI.
5	22.09.2020	9.0	17.1	Removal of duplicated information refer to section 3.3. Refer corrected to 4.2.
5	22.09.2020	9.0	Appendix 2	Removal of histological confirmation of prostate adenocarcinoma from the assessment table.
5	22.09.2020	9.0	Appendix 3	Correction of protocol version 3 dates amended from 15.05.2020 to 26.05.2020.
6	11/02/2022	14.0	General	Throughout - CTC protocol template changes included in sections applicable.
6	11/02/2022	14.0	General Appendix 2	Throughout – ARSI – abiraterone, apalutamide or enzalutamide (PARADIGM-A), or

				enzalutamide (PARADIGM-E) or docetaxel (PARADIGM-D).
6	11/02/2022	14.0	General	Throughout -primary end-point (PFS) changed to major endpoint(s) which now included OS.
6	11/02/2022	14.0	Cover Page	Astellas added as a funder of separate PARADIGM-E cohort.
6	11/02/2022	14.0	Cover Page	Removal of sponsor authorisation signature from the Director.
6	11/02/2022	14.0	Cover Page	Trial statistician details removed.
6	11/02/2022	14.0	Cover Page	Update to TMG membership.
6	11/02/2022	14.0	1.1,1.3	Astellas details added to the funders and reference section. Funders for CTC sample analysis and WB-MRI sub-study included.
6	11/02/2022	14.0	1.1	Target accrual for cohort stipulated as 85 men to have 65 men evaluable for the primary endpoint.
6	11/02/2022	14.0	1.1	Inclusion of target of 6 cycles for docetaxel and until progression for ARSI
6	11/02/2022	14.0	1.1,2.4. & 3.1	Primary objective for cohort E outlined which will be used for validation of cohort A.
6	11/02/2022	14.0	1.1 and 18.1	Cohort E recruitment set for 18 months<br>All cohort patients will be followed up for up to 120 months (from the first patient registered) for PFS and OS and subsequent treatments will be recorded.
6	11/02/2022	14.0	1.1& 3.2	Study major endpoints defined as PFS and OS. PFS amended to include death from any cause instead of prostate cancer specific death.<br>OS included as a major end-point for docetaxel and ARSI cohorts.
6	11/02/2022	14.0	1.1,3.2.2 and 13.4	Replacement of OS with PCSS as the secondary end-point.
6	11/02/2022	14.0	1.1	Treatment summary for the enzalutamide cohort included in study summary.
6	11/02/2022	14.0	1.1	Number of sites participating increased to 20 and confirmation of pre-ADT participating sites included.
6	11/02/2022	14.0	1.2	Study summary updated to include sample collection for cohort E patients.
6	11/02/2022	14.0	1.3	Astellas funding information added in section.
6	11/02/2022	14.0	3.1	Translational objective point 2 primary end-point has been substituted by OS.
6	11/02/2022	14.0	4.1	Removal of monitoring requirements statement
6	11/02/2022	14.0	4.2	Astellas funding information included as well other funders for sub-studies.
6	11/02/2022	14.0	5.0	Number of PIS removed. Main-study and sub-study PIS listed.
6	11/02/2022	14.0	5.1	Cohort E PIS included
6	11/02/2022	14.0	5.1<br>Appendix 3	Addition of guidance outlining remote consent processes for patients recruited onto the main-study and sub-studies remotely.
6	11/02/2022	14.0	7.2	Contact details updated fax number removed.

6	11/02/2022	14.0	8.4	Removal of statement that enzalutamide interim treatment change will be reviewed after 3 months.
6	11/02/2022	14.0	8.5	Standard of care procedure for PARADIGM -E outlined.
6	11/02/2022	14.0	8.2.2	Epic science central lab address updated to reflect their new name 'Labcorp Central Laboratory Services Sàrl'.
6	11/02/2022	14.0	8.7	Pre-ADT recruitment target increased from 50 to 100 patients.<br>Follow-up visits frequency set to be as per SoC. Follow- up samples should not be taken more than once every 3months.
6	11/02/2022	14.0	9,12.5,11.5,12.5 & Appendix 2	WB-MRI scanning window extended from 2 weeks of cycle 3 day 1 to + 2 weeks of cycle 4- day 1.
6	11/02/2022	14.0	11.2	Type of ARSI specified for PARADIGM-A
6	11/02/2022	14.0	11	Throughout section 11 statement added that procedures and assessments will be conducted in in line with standard of care.
6	11/02/2022	14.0	11.4	Statement added to confirm that delayed treatment samples at any timepoint can be collected at later time and a suitable timepoint/visit assigned.
6	11/02/2022	14.0	13	Cohort E recruitment starting point added and clarification on use as validation cohort for A. Calculation sample size section revised to tailor analyses for PARADIGM-A, PARADIGM-D and include PARADIGM-E
6	11/02/2022	14.0	13.1	Outlining of PARADIGM, A, D & E statistical analyses for PFS and OS using hazard ratios across different samples sizes.
6	11/02/2022	14.0	13.2	Major end-points for each cohort specified.
6	11/02/2022	14.0	13.3	Inclusion of OS as major endpoint in analysis. Analyses reporting section for primary ptDNA positive cohorts revised.<br>Statement revised to state that confirmation for a progression date of the first scan should be used is second scan is positive.
6	11/02/2022	14.0	13.4	Statement removed on analyses of PFS.
6	11/02/2022	14.0	14	Added request for baseline forms 2 weeks after registration.
6	11/02/2022	14.0	15.2	Removal of fax number.
6	11/02/2022	14.0	21	Removal of statement on data being published after primary end-point is reached.
7	09/03/2022	14.0	Throughout	Clarification added in sections applicable that PARADIGM E will be activated after recruitment has been completed for PARADIGM A.
8	28/10/2022	18.0	Throughout	Protocol updated to include gut microbiome analysis and stool sample collection in metastatic prostate cancer patient.

8	28/10/2022	18.0	Throughout	Typographical errors corrected throughout the protocol.
8	28/10/2022	18.0	Throughout	Reference to the PARADIGM CTP has been replaced by PARADIGM team.
8	28/10/2022	18.0	Throughout	Clarification added throughout the protocol that mHSPC stands for metastatic hormone sensitive prostate cancer.
8	28/10/2022	18.0	Throughout	CTC Template changes from version 13 dated 08/07/2022 have been incorporated into protocol v8.<br>In Section 4.12 to Clarify that GCP training is not required for all staff responsible for study activities.<br>Section 5: Patient should be given a copy of the PIS & signed ICF.<br>Section 7.2: Instruction for sites to send GP letter added.<br>Section 17:4: updated to clarify that the CTC Director is the custodian of the data.<br>Section 20: Updated with new address of UCL JRO.
8	28/10/2022	18.0	Cover Page	Trial Management Group members updated to removal PARADIGM Clinical Trial Practitioner, Salahah Ahmed Laskar and include Memuna Rashid as the Trial Statistician.
8	28/10/2022	18.0	Cover page	Fax number removed and Memuna Rashid added as the study statistician.
8	28/10/2022	18.0	Section 1.1 & 1.3:	Protocol Summary and Summary of Trial Design updated to include gut microbiome sub-study funder, John Black Foundation.
8	28/10/2022	18.0	Section 1.1, 3.2.4 12:5 & 13.6.6	Translation research updated to include objectives for gut microbiome sub-study.
8	28/10/2022	18.0	Section 2.8	Justification added for conduct of the microbiome analysis in metastatic prostate cancer patients.
8	28/10/2022	18.0	Section 5:3	Title for Next-Generation Sequencing (NGS) Results added.
8	28/10/2022	18.0	Section 5:6	Consent for the gut microbiome sub-study include explaining timepoints where this will occur.
8	28/10/2022	18.0	Section 8.8	Stool sample collection and processing updates included.
8	28/10/2022	18.0	Section 8:9	Stool sample timepoints outlined and explanation provided for when questionnaire should be completed.
8	28/10/2022	18.0	Section11:1	Assessment prior to ADT have been added into section. Clarification included to explain that WB-MRI consent on the MRI PIS before MRI can be performed.
8	28/10/2022	18.0	Section 11:3 & 11:4:	Stool sample and questionnaire completion added to applicable timepoints.

8	28/10/2022	18.0	Section:13	Corrections made to Hazard ratio tables for PARADIGM A & D in statistical section 13.
8	28/10/2022	18.0	Section:13.6.6	Analysis tests which will be used to evaluate changes in gut microbiome defined.
8	28/10/2022	18.0	Section:15.2	Reporting SAR procedures updated to highlight inclusion of WB-MRI.
8.1	15/11/2022	18.0	Section 5.6 Throughout section 11	Removal of terminology referring to patient completing questionnaire. Clarification added to state that questions will be asked by the research team.
8.1	15/11/2022	18.0	Section 8.9 Appendix 2	Header added to differentiate the timeline of stool sample collection from ADT to Progression.<br><br>C1D1 added to main-study registration statement.<br><br>Appendix 2 updated to clarify that there are only 4 collection timepoints for stool sample collection form ADT to Progression. Removal of main-study registration timepoint as sample is being taken at C1D1.
8.2	25/04/2024	28.0	Throughout	Typographical errors corrected throughout the protocol.
8.2	25/04/2024	28.0	Section 1	In protocol version 8.1, SPM was updated from Marian Duggan to Ka Man Mak. SPM has been changed from Ka Man Mak to Rubina Begum in protocol v8.2.<br><br>Laura White has been replaced by Temi Adedoyin the Clinical Programme Lead Manager.<br><br>TMG members list updated to reflect members current on the committee.<br><br>Translational research sub-committee removed in protocol v8.2.<br><br>Pre-ADT recruitment target increased from 50 to 100 patients.<br><br>Recruitment duration extended to 24months from 18months.
8.2	25/04/2024	28.0	Section 5	Microbiome sub-study added
8.2	25/04/2024	28.0	Section 8	Statements added under cohort A and D to confirm recruitment closure dates.<br><br>Pre-ADT recruitment target increased from 50 to 100 patients.

				Note added to confirm that CTC sample collection is no longer required for patients recruited in the pre-ADT sub-study as of April 2023.
8.2	25/04/2024	28.0	Section 9	WB-MRI sub-study closure date confirmed
8.2	25/04/2024	28.0	Section 11	Removal of statement requesting completion of antibiotic use questions at cycle 2-day 1
8.2	25/04/2024	28.0	Appendix 2	Added death column to table in Appendix Transfer of antibiotic use and diet questions from the Standard of Care assessment section to PARADIGM-specific assessments.<br><br>Appendix has been updated to incorporate minor changes reflecting the accurate sample collection timelines.

DocuSign

Certificate Of Completion

Envelope Id: 7975D371924547798B1AB26934F4B098		Status: Completed
Subject: Complete with DocuSign: 20240425_PARADIGM_Protocol_v8.2.pdf		
Source Envelope:		
Document Pages: 92	Signatures: 2	Envelope Originator:
Certificate Pages: 5	Initials: 0	Millenn Chiwewe
AutoNav: Enabled		5th Floor, 90 Tottenham Court Road,
EnvelopeId Stamping: Enabled		London, London W1T4TJ
Time Zone: (UTC) Dublin, Edinburgh, Lisbon, London		m.olu-akinyemi@ucl.ac.uk
		IP Address: 128.40.163.112

Record Tracking

Status: Original<br>30 April 2024 | 16:12	Holder: Millenn Chiwewe<br>m.olu-akinyemi@ucl.ac.uk	Location: DocuSign

Signer Events	Signature	Timestamp
Gerhardt Attard<br>g.attard@ucl.ac.uk<br>PI<br>Security Level: Email, Account Authentication (None)<br><br>**Electronic Record and Signature Disclosure:**<br>Accepted: 01 December 2023 | 16:27<br>ID: befa2d16-e3bc-4aa0-a866-5c81650241df	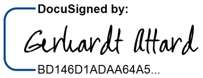<br><br>Signature Adoption: Pre-selected Style<br>Using IP Address: 176.26.108.121<br>Signed using mobile	Sent: 30 April 2024 | 16:15<br>Viewed: 11 May 2024 | 06:32<br>Signed: 11 May 2024 | 06:32
Rubina Begum<br>rubina.begum@ucl.ac.uk<br>Trial Manager<br>CRUK and UCL Cancer Trials Centre<br>Security Level: Email, Account Authentication (None)<br><br>**Electronic Record and Signature Disclosure:**<br>Not Offered via DocuSign	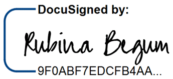<br><br>Signature Adoption: Pre-selected Style<br>Using IP Address: 128.40.163.112	Sent: 30 April 2024 | 16:15<br>Viewed: 30 April 2024 | 16:32<br>Signed: 30 April 2024 | 16:32

In Person Signer Events	Signature	Timestamp

Editor Delivery Events	Status	Timestamp

Agent Delivery Events	Status	Timestamp

Intermediary Delivery Events	Status	Timestamp

Certified Delivery Events	Status	Timestamp

Carbon Copy Events	Status	Timestamp

Witness Events	Signature	Timestamp

Notary Events	Signature	Timestamp

Envelope Summary Events	Status	Timestamps
Envelope Sent	Hashed/Encrypted	30 April 2024 | 16:15
Certified Delivered	Security Checked	30 April 2024 | 16:32
Signing Complete	Security Checked	30 April 2024 | 16:32

Envelope Summary Events	Status	Timestamps
Completed	Security Checked	11 May 2024 | 06:32

Payment Events	Status	Timestamps

Electronic Record and Signature Disclosure

DocuSign Envelope ID: 487536AC-FFA9-4A62-BAA9-C8C921CC9811

CANCER RESEARCH UK & UCL CANCER TRIALS CENTRE
UCL CANCER INSTITUTE

PARADIGM

Plasma Analysis for Response Assessment and to direct the management of Metastatic prostate cancer (PARADIGM)

PARADIGM A & D

STATISTICAL ANALYSIS PLAN

**Version number:**	**1.0**
**Date finalised:**	**16th April 2024**
**Prepared by Trial Statistician:**	Memuna Rashid
Signature:	DocuSigned by: Memuna Rashid 494A955136CF49C...
**Approved by Senior Statistician:**	Andre Lopes
Signature:	DocuSigned by: Andre Lopes FD53A2B5DD3D400...
Date approved:	16-Apr-2024
**Approved by Chief Investigator:**	Professor Gerhardt Attard
Signature:	DocuSigned by: [illegible] BD146D1ADAA64A5...
Date approved:	16-Apr-2024

Contents

Summary description of the PARADIGM study 5

Analysis considerations 6

Study objectives.. 6

Sample size.. 7

Recruitment, follow-up, and baseline characteristics 8

Patient disposition 9

Baseline characteristics 10

COMBINED COHORT PARADIGM-A AND -D.. 12

Analyses of Associations with Overall survival.. 12

Analysis 1 – Descriptive statistics..12

Analysis 2 – Comparison of OS between ptDNA positive versus ptDNA negative at C3D1/C4D1(Co-Primary Analysis)..12

Analysis 3 – Association between OS and ptDNA at C1D1..14

Analysis 4 - Association between OS and PSA level (<0.2, 0.2-4, >4ng/dl) at every cycle and at 7 months after start of ADT..14

Analysis 5 – Predictive value of ptDNA detection and PSA level at C1D1 and at every cycle.15

Analysis 6 – The association with OS and the change in ptDNA detection at C1D1 versus ptDNA detection at C3D1 or C4D1 15

Analysis 7 – The association with OS of the change in PSA at C1D1 versus PSA at C3D1 or C4D1 16

Analysis 8 – To compare ptDNA classification at C2D1 and C5D1 with C3D1 or C4D1...........17

Analysis 9 – The association of OS and the effect of ptDNA fraction prior to ADT.................18

Analysis 10 – Exploratory univariate and multivariate analysis cox regression taking into account baseline characteristics 19

Prostate Cancer Specific Survival (PCSS) 19

Analysis 1 – Descriptive statistics..20

Analysis 2 – Comparison of PCSS between ptDNA positive versus ptDNA negative at C3D1/C4D1(Co-Primary Analysis)..20

Analysis 3 – Association between PCSS and ptDNA at C1D1 21

Analysis 4 - Association between PCSS and PSA level (<0.2, 0.2-4, >4ng/dl) at every cycle and at 7 months after start of ADT 21

Analysis 5– Predictive value of ptDNA detection and PSA level at C1D1 and at every cycle...23

Analysis 6 – The association with PCSS and the change in ptDNA detection at C1D1 versus ptDNA detection at C3D1 or C4D1 23

Analysis 7 – The association with PCSS with the change in PSA at C1D1 versus PSA at C3D1 or C4D1 24

Analysis 8 – To compare ptDNA classification at C2D1 and C5D1 with C3D1 or C4D1...........24

Analysis 9 – The association of PCSS and the effect of ptDNA fraction prior to ADT 25

Analysis 10 – Exploratory univariate analysis cox regression taking into account baseline characteristics 26

DocuSign Envelope ID: 487536AC-FFA9-4A62-BAA9-C8C921CC9811

COHORT PARADIGM-A 27

Analyses of Associations with Progression-free survival........ 27

Analysis 1 – Descriptive statistics........ 27

Analysis 2 – Comparison of PFS between ptDNA positive versus ptDNA negative at C3D1/C4D1(CO-PRIMARY ANALYSIS) 28

Analysis 3 – Association between PFS and ptDNA at C1D1 29

Analysis 4 - Association between PFS and PSA level (<0.2, 0.2-4, >4ng/dl) at every cycle and at 7 months after start of ADT........ 29

Analysis 5– Predictive value of ptDNA detection and PSA level at C1D1 and at every cycle...30

Analysis 6 – The association with PFS and the change in ptDNA detection at C1D1 versus ptDNA detection at C3D1 or C4D1 30

Analysis 7 – The association with PFS andthe change in PSA at C1D1 versus PSA at C3D1 or C4D1 31

Analysis 8 – To compare ptDNA classification at C2D1 and C5D1 with C3D1 or C4D1........ 32

Analysis 9 – The association of PFS and the effect of ptDNA fraction prior to ADT 33

Analysis 10 – Exploratory univariate and multivariate analysis cox regression taking into account baseline characteristics 33

Analyses of Associations with Overall survival........ 33

Analysis 1 – Descriptive statistics........ 34

Analysis 2 – Comparison of OS between ptDNA positive versus ptDNA negative at C3D1/C4D1(Co-Primary Analysis)........ 34

Analysis 3 – Association between OS and ptDNA at C1D1........ 35

Analysis 4 - Association between OS and PSA level (<0.2, 0.2-4, >4ng/dl) at every cycle and at 7 months after start of ADT........ 35

Analysis 5 – Predictive value of ptDNA detection and PSA level at C1D1 and at every cycle.37

Analysis 6 – The association with OS and the change in ptDNA detection at C1D1 versus ptDNA detection at C3D1 or C4D1 37

Analysis 7 – The association with OS of the change in PSA at C1D1 versus PSA at C3D1 or C4D1 38

Analysis 8 – To compare ptDNA classification at C2D1 and C5D1 with C3D1 or C4D1........ 39

Analysis 9 – The association of OS and the effect of ptDNA fraction prior to ADT........ 39

Analysis 10 – Exploratory univariate and multivariate analysis cox regression taking into account baseline characteristics 40

Prostate Cancer Specific Survival (PCSS) 40

Analysis 1 – Descriptive statistics........ 41

Analysis 2 – Comparison of PCSS between ptDNA positive versus ptDNA negative at C3D1/C4D1(Co-Primary Analysis)........ 41

Analysis 3 – Association between PCSS and ptDNA at C1D1 42

Analysis 4 - Association between PCSS and PSA level (<0.2, 0.2-4, >4ng/dl) at every cycle and at 7 months after start of ADT 43

Analysis 5– Predictive value of ptDNA detection and PSA level at C1D1 and at every cycle...44

DocuSign Envelope ID: 487536AC-FFA9-4A62-BAA9-C8C921CC9811

Analysis 6 – The association with PCSS and the change in ptDNA detection at C1D1 versus ptDNA detection at C3D1 or C4D1 44

Analysis 7 – The association with PCSS withthe change in PSA at C1D1 versus PSA at C3D1 or C4D1 45

Analysis 8 – To compare ptDNA classification at C2D1 and C5D1 with C3D1 or C4D1....46

Analysis 9 – The association of PCSS and the effect of ptDNA fraction prior to ADT 47

Analysis 10 – Exploratory univariate analysis cox regression taking into account baseline characteristics 47

The concordance between ptDNA classification at C2D1/C5D1and at C3/C4.... 47

The Analysis of Changes in ptDNA and PSA Levels at Different Time Points.... 49

COHORT PARADIGM-D 49

Recruitment, follow-up, and baseline characteristics 50

Progression-free survival 51

Summary description of the PARADIGM study

Trial design

PARADIGM is a prospective, observational, biomarker-focused, translational platform, cohort study in newly diagnosed poly-metastatic prostate cancer patients starting long-term systemic therapy.

There are 3 cohorts:

- PARADIGM-D: Docetaxel
- PARADIGM-A: Androgen receptor signalling inhibitor (ARSI) – abiraterone, apalutamide or enzalutamide
- PARADIGM-E: Enzalutamide

PARADIGM-A and PARADIGM-D opened to accrual concurrently (funded by a Clinical Trials Award from Prostate Cancer UK). PARADIGM-E was added to the protocol as an amendment following award of a collaborative grant from Astellas. PARADIGM-E is regarded as a validation cohort and will be reported at a later date. This SAP is only applicable to cohorts A and D.

Cohorts A and D are not randomised and designed to reflect local practice and changing clinical guidelines. The protocol *a priori* stated that reporting will depend on patient accrual to the two cohorts. It was anticipated that data emerging over the accrual period could influence docetaxel use, which in effect occurred with a notable reduction in single-agent docetaxel use, accelerated by the 2020 Covid pandemic. Enzalutamide received NICE funding approval on 23/04/2020 as an interim treatment change option for the treatment of prostate cancer in this patient population during the COVID-19 pandemic, as endorsed by NHS England. Enzalutamide is likely to remain an option as a standard of care treatment if local funding arrangements are in place Cohort-D was therefore expected to under-recruit whilst Cohort-A was allowed to over-recruit. The Trial Management Group agreed to report all end-points for Cohort-A and combine Cohorts-A and -D for reporting for survival-based end-points.

Patients have been classified as plasma tumour DNA positive or negative based on the PCF-SELECT framework as pubished in Orlando et al, Nucleic Acids Research Cancer (PMID: 35664542, DOI: 10.1093/narcan/zcac016) and based on the following decision tree:

1. detection of tumour ≥0.03 based on allelic imbalance at sites of hemi-deletion,

Or

2. tumor estimation at 0.01-0.02 based on allelic imbalance with secondary evidence of tumor copy number alterations.

Analysis considerations

Aspects	Description
Primary aim	The primary aim of this study is to determine whether the detection of plasma tumour DNA (ptDNA) after two or three cycles of ARSI or docetaxel added after start of ADT is associated with a worse clinical outcome in newly diagnosed poly-metastatic prostate cancer.
Study outcomes	Progression-Free Survival (PFS) (major endpoint), Overall Survival (OS) (major endpoint) and Prostate Cancer Specific Survival (PCSS) (secondary endpoint).
Interim analysis	PFS will be reported when 30% of patients have had a PFS event or when an expected 50% of PFS events have occurred in the ptDNA positive, whichever occurs first. As stated in the protocol, OS will also be reported at the primary analysis but a second later analysis for OS could be considered if insufficient events had occurred and the trend for association was consistent with a significant association for PFS.
Analysis population	All eligible patients, except those without plasma samples collected at either Cycle 3 Day 1 or Cycle 4 Day 1, will be included in the analysis
Sensitivity analysis	Subsequent sensitivity analysis will be conducted on patients who do not fulfil the criteria stated in Section 13.2 of the protocol.

Study objectives

Aspects	Description
**Major endpoints**	• Progression-free survival<br>• Overall survival
**Major objective**	• To determine whether the detection of plasma tumour DNA (ptDNA) after two or three cycles of ARSI added after start of ADT is associated with a worse clinical outcome in newly diagnosed metastatic prostate cancer.
**Secondary outcomes**	• Prostate Cancer-specific survival
**Secondary objectives**	• To compare ptDNA classification at C2D1 and C5D1 with C3D1 or C4D1.<br>• To determine whether the detection of ptDNA after five to sixteen weeks of starting ADT and prior to starting ARSI is associated with a worse clinical outcome.<br>• To determine the association between clinical outcome and prostate specific antigen (PSA) level (<0.2, 0.2-4, >4ng/dl) after five to sixteen weeks of starting ADT and prior to starting ARSI, prior to treatment at every cycle and at 7 months after start of ADT.

Aspects	Description
	• To assess whether ptDNA detection is a better predictor of clinical outcome than PSA level (as assessed in objective 3) after five to sixteen weeks of starting ADT and prior to treatment at every cycle.<br>• To compare associations with clinical outcome for the change in ptDNA detection and PSA level (as assessed in objective 3) prior to start of ARSI and at C3D1 or C4D1.<br>• To evaluate whether ptDNA fraction prior to LHRHa (stratified by no anti-androgen versus 2-3 weeks of anti-androgen) is associated with Progression Free Survival (PFS) and Overall Survival (OS) – as part of pre-ADT translational sub-study.

Sample size

Parameters	Assumptions
Outcome to power the study	• The sample size was powered individually for PARADIGM-A and -D on PFS outcome and the PFS comparison between ptDNA-negative and ptDNA-positive patients.
Accrual period of this study	• The anticipated accrual was 18 months with a minimum follow-up period of 36 months. This had to be extended due to reduced accrual during the Covid pandemic.
Prevalence ratio ptDNA-positive and negative	• Approximately 20% of patients were anticipated to be ptDNA-positive, whereas 80% should be ptDNA-negative.
Sample size justification	• It was anticipated that the 12-month PFS rate for ptDNA-positive patients is 60%. With a power of 73% and a 2-sided significance level of 10%, approximately 65 patients should be sufficient to observe 41 PFS events. This should be sufficient to detect a 20% greater 12-month PFS rate between ptDNA-negative and ptDNA-positive patients, corresponding to a PFS HR=0.437 (PASS sample size software, v2020.0.5).<br>• With a sample size of 65 patients, this study will also have >90% power to detect a larger-than-anticipated difference in PFS rates between ptDNA groups, such as a 12-month PFS rate of 85% for negative ptDNA and 60% for positive ptDNA (equating to a PFS HR of 0.317). In this instance, a total of 34 PFS occurrences are required for analyses.
Recruitment considerations	• The samples size is also large enough to detect similar hazard ratios for OS with longer follow-up and sufficient

Parameters	Assumptions
	events. If accrual rates are higher than expected during the recruitment period, we may continue to enrol beyond 65 evaluable patients to increase power for potentially less extreme hazard ratios (e.g. 0.4 to 0.5).

Recruitment, follow-up, and baseline characteristics

Aspects	Description
Recruitment information to be reported	• The day, month and year between which patients were recruited.<br>• The accrual rate per month of accrual<br>• The number of centres that recruited patients and the total number of patients recruited at each centre<br>• The number of patients who were recruited to the trial but then withdrew, and the reasons (if available), divided by whether patients withdraw from sample collection alone or sample collection and follow-up reporting.<br>• The number of patients who were ineligible for primary end-point analysis and the reasons why they were ineligible
Follow-up information to be reported	• The reverse K- M curve will be used to calculate the median follow up. The median follow-up time will be presented with its 95% confidence interval.
Baseline characteristics information to be reported	• A table of baseline characteristics. This should contain age, and any other relevant factor such as disease stage, performance status, and other key biological and physiological measurements.<br>• For categorical variables, each column will contain N (%)<br>• For continuous variables, each column will contain the median value, and in brackets, the range (minimum and maximum) or IQR if more appropriate.

DocuSign Envelope ID: 487536AC-FFA9-4A62-BAA9-C8C921CC9811

Patient disposition

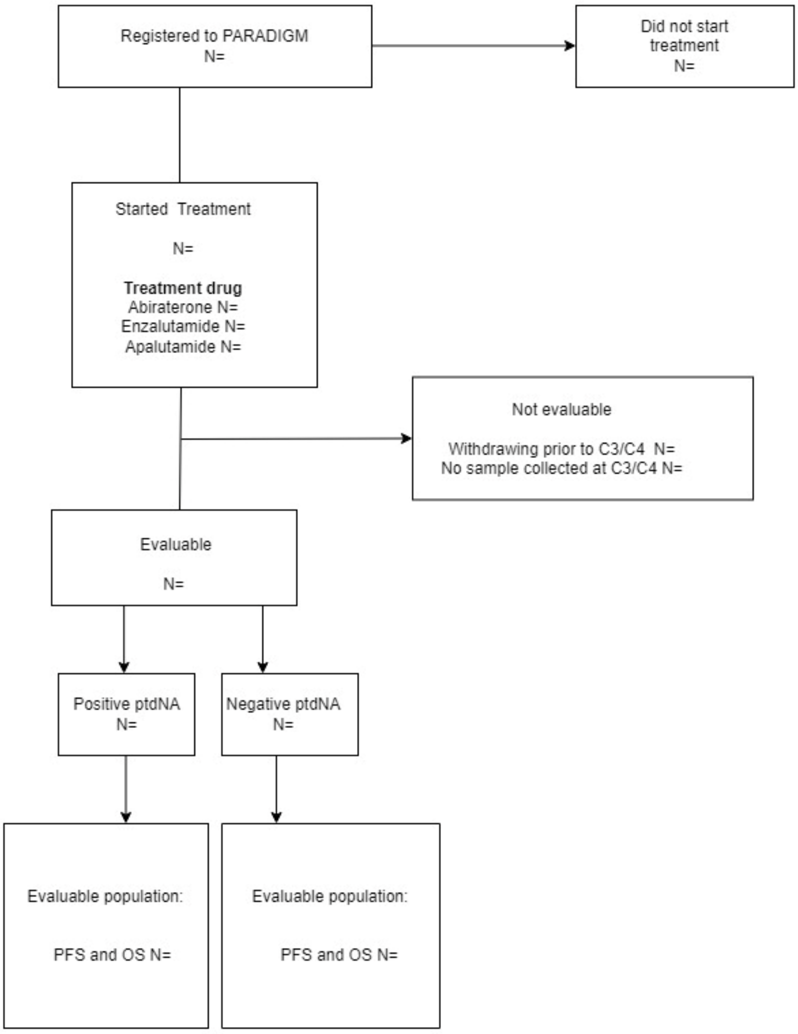

The patient disposition for PARADIGM-D and for PARADIGM-A and -D will also be presented using the same format.

Baseline characteristics

Baseline characteristics will be presented for PARADIGM-A and -D separately and combined.

**Baseline characteristics**	**Overall cohort N (%) N=**	**ptDNA positive N (%) N=**	**ptDNA negative N (%) N=**
**Age at registration (years)**			
Median (range)			
< 65 years			
65 - 74 years			
≥ 75 years			
**ECOG**			
0-1			
≥2			
**Gleason score**			
≤ 7			
≥8			
No biopsy taken*			
**Metastatic site**			
Presence of >= 5 bone metastases			
Presence of >=1 unequivocal visceral metastases			
Both bone and visceral metastases			
**Ethnicity**			
White			
Black			
Asian			
Mixed			
Other			
**ADT (at C1D1)**			
LHRH analogue/agonist			
LHRH antagonist			
**Time on ADT before C1D1 (days)**			
Median (range)			
**Has the patients had anti-androgens?**			
Yes			
No			
**Previous Prostatectomy**			
Yes			
No			

Baseline characteristics	Overall cohort N (%) N=	ptDNA positive N (%) N=	ptDNA negative N (%) N=
**Previous Radiotherapy**			
Yes			
No			
**Pre-ADT PSA**			
Median (range)			

COMBINED COHORT PARADIGM-A AND -D

- The two cohorts will be combined and an estimate of the HR for OS and PCSS by ptDNA status will be calculated
- Whether there is an interaction between ptDNA and cohorts can be tested

Analyses of Associations with Overall survival

Definition of Overall Survival

Outcome	Type of endpoint	Definition
**Overall survival (OS)**	Major/Primary	Overall survival (OS) is defined as the interval from C1D1 (start of ARSI) to death from any cause.<br><br>Patients without documented death at the time of the final analysis will be censored at the date of the last follow-up.

Analysis 1 – Descriptive statistics

- The OS rate at 12, 24 and 36 months will be reported using the Kaplan Meier method with the 95% CI.
- The median time to event will also be reported with 95% CI.
- OS curve will be depicted using Kaplan Meier plot.
- This will be done for the overall cohort and then separately for the ptDNA subgroups

Overall survival	Overall cohort
N	
N events	
Median time in months (95% CI)	
12 months event-free rate (95% CI)	
24 months event-free rate (95% CI)	
36 months event-free rate (95% CI)	

Analysis 2 – Comparison of OS between ptDNA positive versus ptDNA negative at C3D1/C4D1(Co-Primary Analysis)

- Patients will be defined as ptDNA positive if tumour is detected at either C3D1 or C4D1. Some patients may have only one time-point. For patients with measurements at both C3D1 and C4D1, detection at either time-point will classify the patient as ptDNA positive.
- A comparison of OS between ptDNA positive and ptDNA negative patients will be performed using a log-rank test and cox regression.
- It is possible that the ptDNA test may be associated with a smaller difference in OS at, for example, 18-24 months, than the difference in the first 12-18 months. The Wilcoxon test for time-to-event analyses which gives a higher weight to events that occur earlier will also be performed.
- The proportionality of hazards (PH) assumption will be tested on the basis of Schoenfeld residuals after fitting the cox model.
    - If there is no evidence that the PH assumption is not fulfilled at the 10% significance level, then the HR, 95% CI and p-values for comparison of ptDNA groups derived from the cox model will be reported.
    - If there is evidence that the PH assumption is not fulfilled at the 10% significance level, then the difference in the Restricted Mean Survival Time (RMST) at 18 and 24 months between the ptDNA groups will be computed along with bootstrap based 95%CI and p-value.
- As a sensitivity analysis, we will also perform an adjusted Cox regression where patient tumour factors will be included as covariates in the model. Subsequent analysis will be done adjusting for sites; sites with fewer than 4 patients will be combined for this analysis.

**Overall survival**	**ptDNA status**	
	**Negative**	**Positive**
N		
N events		
Median time in months (95% CI)		
12 months event-free rate (95% CI)		
24 months event-free rate (95% CI)		
36 months event-free rate (95% CI)		
Cox regression		
HR (95%CI)	1.00 (reference)	
p value	-	
24-months RMST		
Estimates		
Difference in RMST (95%CI)	0 (reference)	
p-value		

Analysis 3 – Association between OS and ptDNA at C1D1

- The frequency and percentage of patients who are ptDNA positive and negative ptDNA at C1D1 will be reported. Frequencies will also be reported for groups split by 5-10 and >10-16 weeks of ADT.
  The association between ptDNA at C1D1 and OS will be performed by presenting KM estimates by ptDNA status; OS comparison between ptDNA status will be performed using a log-rank test and cox regression; OS will be measured from date of blood draw (ptDNA measurement taken (landmark analysis).

Analysis 4 - Association between OS and PSA level (<0.2, 0.2-4, >4ng/dl) at every cycle and at 7 months after start of ADT

- OS will be compared across the 3 PSA groups using cox regression
- HRs, 95%CI and p-value from cox regression will be reported
- A landmark analysis will be performed for PSA subgroups defined during treatment.

**Overall survival**	**PSA levels in ng/ml**		
	**≤ 0.2**	**> 0.2 to 4**	**>4**
**PSA C1D1**			
N			
N events			
Median time in months (95% CI)			
12 months event-free rate (95% CI)			
24 months event-free rate (95% CI)			
36 months event-free rate (95% CI)			
HR (95%CI)	1.00(reference)		
p value	-		
**PSA C2D1**			
N			
N events			
Median time in months (95% CI)			
12 months event-free rate (95% CI)			
24 months event-free rate (95% CI)			
36 months event-free rate (95% CI)			
HR (95%CI)	1.00 reference)		
p value	-		
**PSA C3D1/C4D1**			
N			
N events			
Median time in months (95% CI)			
12 months event-free rate (95% CI)			
24 months event-free rate (95% CI)			

DocuSign Envelope ID: 487536AC-FFA9-4A62-BAA9-C8C921CC9811

**Overall survival**	**PSA levels in ng/ml**		
	**≤ 0.2**	**> 0.2 to 4**	**>4**
36 months event-free rate (95% CI)			
HR (95%CI)	1.00 reference)		
p value	-		
**PSA C5D1**			
N			
N events			
Median time in months (95% CI)			
12 months event-free rate (95% CI)			
24 months event-free rate (95% CI)			
36 months event-free rate (95% CI)			
HR (95%CI)	1.00 reference)		
p value	-		
**PSA 7 months after start of ADT**			
N			
N events			
Median time in months (95% CI)			
12 months event-free rate (95% CI)			
24 months event-free rate (95% CI)			
36 months event-free rate (95% CI)			
HR (95%CI)	1.00 reference)		
p value	-		

Analysis 5 – Predictive value of ptDNA detection and PSA level at C1D1 and at every cycle

- ptDNA and PSA level will be compared in terms of detection of patients who experienced an OS event at 12,18,24 and 36 months or not in a qualitative manner using frequencies, percentages, sensitivity rate and false positive rate.

Analysis 6 – The association with OS and the change in ptDNA detection at C1D1 versus ptDNA detection at C3D1 or C4D1

- The number of patients who belong to each of the following four groups will be presented; (+ve pre-, +ve on treatment), (+ve pre-, -ve on treatment), (-ve pre-, +ve on treatment), (-ve pre-, -ve on treatment).
- OS will be compared across the four groups using cox regression
- HRs, 95%CI and p-value from cox regression will be reported
- A landmark analysis will be performed for subgroups defined at C3D1 or C4D1

**Overall survival**	**Change in ptDNA detection at C1D1 with ptDNA detection at C3D1 or C4D1**			
	No change in ptDNA-	No change in ptDNa+	ptDNA+ to ptDNA-	ptDNA- to ptDNA+
N<br>N events<br>Median time in months (95% CI)<br>12 months event-free rate (95% CI)<br>24 months event-free rate (95% CI)<br>36 months event-free rate (95% CI)				
Cox regression<br>HR (95%CI)<br>p value	 1.00 (reference)<br>-			
24-months RMST<br>Estimates<br>Difference in RMST (95%CI)<br>p-value	 <br>0 (reference)			

Analysis 7 – The association with OS of the change in PSA at C1D1 versus PSA at C3D1 or C4D1

- The number of patients who belong to each of the following three groups will be presented; (PSA<0.01 at C1D1 and C3D1 and/or C4D1), (≥0.01 C3D1/C4D1 with any PSA at C1D1), (≥0.01 at C1D1 but <0.01 at C3D1 and/or C4D1).
- OS will be compared across the three groups using cox regression

- HRs, 95%CI and p-value from cox regression will be reported
- A landmark analysis will be performed for subgroups defined at C3D1 or C4D1

**Overall survival**	**Change in PSA at C1D1 with PSA at C3D1 or C4D1**		
	PSA<0.01 at C1D1 and C3D1 and/or C4D1)	(≥0.01 C3D1/C4D1 with any PSA at C1D1	0.01 at C1D1 but <0.01 at C3D1 and/or C4D1
N<br>N events<br>Median time in months (95% CI)<br>12 months event-free rate (95% CI)<br>24 months event-free rate (95% CI)<br>36 months event-free rate (95% CI)<br><br>Cox regression<br>HR (95%CI)<br>p value<br><br>24-months RMST<br>Estimates<br>Difference in RMST (95%CI)<br>p-value	 <br><br><br><br><br><br><br>1.00 (reference)<br>-<br><br><br><br>0 (reference)		

Analysis 8 – To compare ptDNA classification at C2D1 and C5D1 with C3D1 or C4D1

**Overall Survival**	**N**	**N events**	**Median time in months (95% CI)**	**36 months event-free rate (95% CI)**	**HR (95%CI)**	**p value**
**Overall cohort**					-	-
**ptDNA C2D1**						

Negative					1.00 (reference)	-
Positive						
**ptDNA C5D1**						
Negative					1.00 (reference)	-
Positive						
**ptDNA C3D1/C4D1**						
Negative					1.00 (reference)	-
Positive						

Analysis 9 – The association of OS and the effect of ptDNA fraction prior to ADT

- This analyses will be performed on the sub-set of patients who have consented to the pre-ADT sub-study. This may be reported separately when additional samples are obtained.
- This analysis will be performed on the sub-set of patients who have pre-ADT samples. This may be reported separately when additional samples are obtained.
- The continuous measurement of ptDNA fraction taken prior to ADT will be assessed in a descriptive manner using histograms.
- The frequency, mean, standard deviation, median and range of ptDNA fraction prior to ADT will be reported
- An unadjusted and adjusted cox regression will be used to evaluate the association between ptDNA fraction taken prior to ADT and OS.
- If appropriate, ptDNA fraction prior to ADT will be categorised in tertitles and OS curves will be plotted using the Kaplan Meier curve
- ROC analysis will be used to evaluate the best cut-off value of ptDNA fraction prior to ADT for detection of OS event at 24 months, which will be selected based on the highest ratio

DocuSign Envelope ID: 487536AC-FFA9-4A62-BAA9-C8C921CC9811

sensitivity rate over false positive rate. The ROC analysis will account for age and ethnicity too. The ROC analysis of ptDNA will also be compared to that of PSA.

Analysis 10 – Exploratory univariate and multivariate analysis cox regression taking into account baseline characteristics

An exploratory univariate and multivariate cox model for OS will also be performed taking into account baseline characteristics:

- Age at registration (continuous)
- Pre-ADT PSA (continuous)
- ECOG (categorical)
- Gleason score(≤7 vs ≥8)
- Visceral disease: yes vs no
- C1D1 PSA (continuous)
- Other relevant covariates

Backward selection will be used to select the multivariate Cox model with a 5% significance level for terms to be removed from the model. Given the strong possibility that pre-ADT PSA and C1D1 PSA may be collinear, C1D1 PSA will be the variable included in the multivariate analysis and pre-ADT PSA will be excluded from the model if they are found to be correlated.

Prostate Cancer Specific Survival (PCSS)

Definition of Prostate Cancer Specific Survival

Outcome	Type of endpoint	Definition
**Prostate Cancer Specific Survival (PCSS)**	Secondary	Prostate Cancer Specific Survival (PCSS) defined as interval from C1D1 to death from prostate cancer.<br><br>Patient without documented death from Prostate cancer at the time of the final analysis will be censored at the date of the last follow-up.

The results will be presented as follows:

Analysis 1 – Descriptive statistics

- The PCSS rate at 12 months, 24 and 36 months will be reported using the Kaplan Meier method along with the 95%CI.
- The median time to event will also be reported with 95% CI.
- PCSS curve will be depicted using Kaplan Meier plot.
- This will be done for the overall cohort and then separately for the ptDNA subgroups

Prostate Cancer Specific Survival	Overal Cohort
N<br>N events<br>Median time in months (95% CI)<br>12 months event-free rate (95% CI)<br>24 months event-free rate (95% CI)<br>36 months event-free rate (95% CI)	

Analysis 2 – Comparison of PCSS between ptDNA positive versus ptDNA negative at C3D1/C4D1(Co-Primary Analysis)

- Patients will be defined as ptDNA positive if tumour is detected at either C3D1 or C4D1. Some patients may have only one time-point. For patients with measurements at both C3D1 and C4D1, detection at either time-point will classify the patient as ptDNA positive.
- A comparison of PCSS between ptDNA positive and ptDNA negative patients will be performed using a log-rank test and cox regression.
- It is possible that the ptDNA test may be associated with a smaller difference in PCSS at, for example, 18-24 months, than the difference in the first 12-18 months. The Wilcoxon test for time-to-event analyses which gives a higher weight to events that occur earlier will also be performed.
- The proportionality of hazards (PH) assumption will be tested on the basis of Schoenfeld residuals after fitting the cox model.
    - If there is no evidence that the PH assumption is not fulfilled at the 10% significance level, then the HR, 95% CI and p-values for comparison of ptDNA groups derived from the cox model will be reported.
    - If there is evidence that the PH assumption is not fulfilled at the 10% significance level, then the difference in the Restricted Mean Survival Time (RMST) at 18 and 24 months between the ptDNA groups will be computed along with bootstrap based 95%CI and p-value.

- As a sensitivity analysis, we will also perform an adjusted Cox regression where patient tumour factors will be included as covariates in the model. Subsequent analysis will be done adjusting for sites; sites with fewer than 4 patients will be combined for this analysis.

**Prostate Cancer Specific Survival**	**ptDNA status**	
	**Negative**	**Positive**
N		
N events		
Median time in months (95% CI)		
12 months event-free rate (95% CI)		
24 months event-free rate (95% CI)		
36 months event-free rate (95% CI)		
Cox regression		
HR (95%CI)	1.00 (reference)	
p value	-	
24-months RMST		
Estimates		
Difference in RMST (95%CI)	0 (reference)	
p-value		

Analysis 3 – Association between PCSS and ptDNA at C1D1

- The frequency and percentage of patients who are ptDNA positive and negative ptDNA at C1D1 will be reported. Frequencies will also be reported for groups split by 5-10 and >10-16 weeks of ADT.
  The association between ptDNA at C1D1 and PCSS will be performed by presenting KM estimates by ptDNA status; PCSS comparison between ptDNA status will be performed using a log-rank test and cox regression; PCSS will be measured from date of blood draw (ptDNA measurement taken (landmark analysis).

Analysis 4 - Association between PCSS and PSA level (<0.2, 0.2-4, >4ng/dl) at every cycle and at 7 months after start of ADT

- PCSS will be compared across the 3 PSA groups using cox regression
- HRs, 95%CI and p-value from cox regression will be reported
- A landmark analysis will be performed for PSA subgroups defined during treatment.

**Prostate Cancer Specific Survival**	**PSA levels in ng/ml**		
	**≤ 0.2**	**> 0.2 to 4**	**>4**
**PSA C1D1**			
N			
N events			

**Prostate Cancer Specific Survival**	**PSA levels in ng/ml**		
	**≤ 0.2**	**> 0.2 to 4**	**>4**
Median time in months (95% CI)			
12 months event-free rate (95% CI)			
24 months event-free rate (95% CI)			
36 months event-free rate (95% CI)			
HR (95%CI)	1.00(reference)		
p value	-		
**PSA C2D1**			
N			
N events			
Median time in months (95% CI)			
12 months event-free rate (95% CI)			
24 months event-free rate (95% CI)			
36 months event-free rate (95% CI)			
HR (95%CI)	1.00 reference)		
p value	-		
**PSA C3D1/C4D1**			
N			
N events			
Median time in months (95% CI)			
12 months event-free rate (95% CI)			
24 months event-free rate (95% CI)			
36 months event-free rate (95% CI)			
HR (95%CI)	1.00 reference)		
p value	-		
**PSA C5D1**			
N			
N events			
Median time in months (95% CI)			
12 months event-free rate (95% CI)			
24 months event-free rate (95% CI)			
36 months event-free rate (95% CI)			
HR (95%CI)	1.00 reference)		
p value	-		
**PSA 7 months after start of ADT**			
N			
N events			
Median time in months (95% CI)			
12 months event-free rate (95% CI)			
24 months event-free rate (95% CI)			
36 months event-free rate (95% CI)			
HR (95%CI)	1.00 reference)		
p value	-		

Analysis 5– Predictive value of ptDNA detection and PSA level at C1D1 and at every cycle

- ptDNA and PSA level will be compared in terms of detection of patients who experienced an PCSS event at 12,18,24 and 36 months or not in a qualitative manner using frequencies, percentages, sensitivity rate and false positive rate.

Analysis 6 – The association with PCSS and the change in ptDNA detection at C1D1 versus ptDNA detection at C3D1 or C4D1

- The number of patients who belong to each of the following four groups will be presented; (+ve pre-, +ve on treatment), (+ve pre-, -ve on treatment), (-ve pre-, +ve on treatment), (-ve pre-, -ve on treatment).
- PCSS will be compared across the four groups using cox regression
- HRs, 95%CI and p-value from cox regression will be reported
- A landmark analysis will be performed for subgroups defined at C3D1 or C4D1

**Prostate Cancer Specific Survival**	**Change in ptDNA detection at C1D1 with ptDNA detection at C3D1 or C4D1**			
	No change in ptDNA-	No change in ptDNa+	ptDNA+ to ptDNA-	ptDNA- to ptDNA+
N<br>N events<br>Median time in months (95% CI)<br>12 months event-free rate (95% CI)<br>24 months event-free rate (95% CI)<br>36 months event-free rate (95% CI)				
Cox regression<br>HR (95%CI)<br>p value	 1.00 (reference)<br>-			
24-months RMST<br>Estimates<br>Difference in RMST (95%CI)<br>p-value	 <br>0 (reference) 			

Analysis 7 – The association with PCSS with the change in PSA at C1D1 versus PSA at C3D1 or C4D1

- The number of patients who belong to each of the following three groups will be presented; (PSA<0.01 at C1D1 and C3D1 and/or C4D1), (≥0.01 C3D1/C4D1 with any PSA at C1D1), (≥0.01 at C1D1 but <0.01 at C3D1 and/or C4D1).
- PCSS will be compared across the three groups using cox regression
- HRs, 95%CI and p-value from cox regression will be reported
- A landmark analysis will be performed for subgroups defined at C3D1 or C4D1

**Prostate Cancer Specific Survival**	**Change in PSA at C1D1 with PSA at C3D1 or C4D1**		
	Increased PSA	Decreased PSA	No change in PSA
N<br>N events<br>Median time in months (95% CI)<br>12 months event-free rate (95% CI)<br>24 months event-free rate (95% CI)<br>36 months event-free rate (95% CI)<br><br>Cox regression<br>HR (95%CI)<br>p value<br><br>24-months RMST<br>Estimates<br>Difference in RMST (95%CI)<br>p-value	 <br><br><br><br><br><br><br>1.00 (reference)<br>-<br><br><br><br>0 (reference)		

Analysis 8 – To compare ptDNA classification at C2D1 and C5D1 with C3D1 or C4D1

**Prostate Cancer Specific Survival**	**N**	**N events**	**Median time in months (95% CI)**	**36 months event-free rate (95% CI)**	**HR (95%CI)**	**p value**
**Overall cohort**					-	-

**ptDNA C2D1**						
Negative					1.00 (reference)	-
Positive						
**ptDNA C5D1**						
Negative					1.00 (reference)	-
Positive						
**ptDNA C3D1/C4D1**						
Negative					1.00 (reference)	-
Positive						

Analysis 9 – The association of PCSS and the effect of ptDNA fraction prior to ADT

- This analyses will be performed on the sub-set of patients who have consented to the pre-ADT sub-study. This may be reported separately when additional samples are obtained.
- This analysis will be performed on the sub-set of patients who have pre-ADT samples. This may be reported separately when additional samples are obtained.
- The continuous measurement of ptDNA fraction taken prior to ADT will be assessed in a descriptive manner using histograms.
- The frequency, mean, standard deviation, median and range of ptDNA fraction prior to ADT will be reported
- An unadjusted and adjusted cox regression will be used to evaluate the association between ptDNA fraction taken prior to ADT and PCSS.
- If appropriate, ptDNA fraction prior to ADT will be categorised in tertiles and PCSS curves will be plotted using the Kaplan Meier curve
- ROC analysis will be used to evaluate the best cut-off value of ptDNA fraction prior to ADT for detection of PCSS event at 24 months, which will be selected based on the highest ratio sensitivity rate over false positive rate. The ROC analysis will account for age and ethnicity too. The ROC analysis of ptDNA will also be compared to that of PSA.

DocuSign Envelope ID: 487536AC-FFA9-4A62-BAA9-C8C921CC9811

Analysis 10 – Exploratory univariate analysis cox regression taking into account baseline characteristics

An exploratory univariate and multivariate cox model for PCSS will also be performed taking into account baseline characteristics:

- Age at registration (continuous)
- Pre-ADT PSA (continuous)
- ECOG (categorical)
- Gleason score(≤7 vs ≥8)
- Visceral disease: yes vs no
- C1D1 PSA (continuous)

Backward selection will be used to select the multivariate Cox model with a 5% significance level for terms to be removed from the model. Given the strong possibility that pre-ADT PSA and C1D1 PSA are collinear, C1D1 PSA will be the variable included in the multivariate analysis and pre-ADT PSA will be excluded from the model if they are found to be correlated.

COHORT PARADIGM-A

Analyses of Associations with Progression-free survival

Definition of Progression-Free Survival

Outcome	Type of endpoint	Definition
**Progression-free survival (PFS)**	Major/Primary	Progression-free survival (PFS) is defined as the interval from C1D1 (start of ARSI), to disease progression as determined by any of:<br>• Symptomatic or asymptomatic new or unequivocal progression of prior distant metastases confirmed by imaging<br>• Symptomatic progression of cancer in the prostate confirmed by imaging<br>• Death from any cause<br><br>Patients without documented death or documented disease progression at the time of the final analysis will be censored at the date of the last follow-up.

Analysis 1 – Descriptive statistics

- Break-down of components of PFS endpoint, showing proportion (or %): radiographic progression of distant metastases, local progression, death
- The PFS rate at 12, 24 and 36 months will be reported using the Kaplan Meier method with 95%CI.
- The median time to event will also be reported with 95% CI.
- PFS curve will be depicted using Kaplan Meier plot.
- This will be done for the overall cohort and then separately for the ptDNA subgroups

Progression-free survival	PARADIGM A
N	
N events	
Median time in months (95% CI)	
12 months event-free rate (95% CI)	
24 months event-free rate (95% CI)	
36 months event-free rate (95% CI)	

Analysis 2 – Comparison of PFS between ptDNA positive versus ptDNA negative at C3D1/C4D1(CO-PRIMARY ANALYSIS)

- Patients will be defined as ptDNA positive if tumour is detected at either C3D1 or C4D1. Some patients may have only one time-point. For patients with measurements at both C3D1 and C4D1, detection at either time-point will classify the patient as ptDNA positive.
- A comparison of PFS between ptDNA positive and ptDNA negative patients will be performed using a log-rank test and cox regression.
- It is possible that the ptDNA test may be associated with a smaller difference in PFS at, for example, 18-24 months, than the difference in the first 12-18 months. The Wilcoxon test for time-to-event analyses which gives a higher weight to events that occur earlier will also be performed.
- The proportionality of hazards (PH) assumption will be tested on the basis of Schoenfeld residuals after fitting the cox model.
    - If there is no evidence that the PH assumption is not fulfilled at the 10% significance level, then the HR, 95% CI and p-values for comparison of ptDNA groups derived from the cox model will be reported.
    - If there is evidence that the PH assumption is not fulfilled at the 10% significance level, then the difference in the Restricted Mean Survival Time (RMST) at 18 and 24 months between the ptDNA groups will be computed along with bootstrap based 95%CI and p-value.
- As a sensitivity analysis, we will also perform an adjusted Cox regression where patient tumour factors will be included as covariates in the model. Subsequent analysis will be done adjusting for sites; sites with fewer than 4 patients will be combined for this analysis.

**Progression-free survival**	**pDNA status**	
	**Negative**	**Positive**
N		
N events		
Median time in months (95% CI)		
12 months event-free rate (95% CI)		
24 months event-free rate (95% CI)		
36 months event-free rate (95% CI)		
Cox regression		
HR (95%CI)	1.00 (reference)	
p value	-	
24-months RMST		
Estimates		
Difference in RMST (95%CI)	0 (reference)	
p-value		

Analysis 3 – Association between PFS and ptDNA at C1D1

- The frequency and percentage of patients who are ptDNA positive and negative ptDNA at C1D1 will be reported. Frequencies will also be reported for groups split by 5-10 and >10-16 weeks of ADT.

The association between ptDNA at C1D1 and PFS will be performed by presenting KM estimates by ptDNA status; PFS comparison between ptDNA status will be performed using a log-rank test and cox regression; PFS will be measured from date of blood draw (ptDNA measurement taken (landmark analysis).

Analysis 4 - Association between PFS and PSA level (<0.2, 0.2-4, >4ng/dl) at every cycle and at 7 months after start of ADT

- PFS will be compared across the 3 PSA groups using cox regression
- HRs, 95%CI and p-value from cox regression will be reported
- A landmark analysis will be performed for PSA subgroups defined during treatment.

**Progression-free survival**	**PSA levels in ng/ml**		
	**≤ 0.2**	**> 0.2 to 4**	**>4**
**PSA C1D1**			
N			
N events			
Median time in months (95% CI)			
12 months event-free rate (95% CI)			
24 months event-free rate (95% CI)			
36 months event-free rate (95% CI)			
HR (95%CI)	1.00(reference)		
p value	-		
**PSA C2D1**			
N			
N events			
Median time in months (95% CI)			
12 months event-free rate (95% CI)			
24 months event-free rate (95% CI)			
36 months event-free rate (95% CI)			
HR (95%CI)	1.00 reference)		
p value	-		
**PSA C3D1/C4D1**			
N			
N events			
Median time in months (95% CI)			
12 months event-free rate (95% CI)			
24 months event-free rate (95% CI)			
36 months event-free rate (95% CI)			

Progression-free survival	PSA levels in ng/ml		
	≤ 0.2	> 0.2 to 4	>4
HR (95%CI)	1.00 reference)		
p value	-		
**PSA C5D1**			
N			
N events			
Median time in months (95% CI)			
12 months event-free rate (95% CI)			
24 months event-free rate (95% CI)			
36 months event-free rate (95% CI)			
HR (95%CI)	1.00 reference)		
p value	-		
**PSA 7 months after start of ADT**			
N			
N events			
Median time in months (95% CI)			
12 months event-free rate (95% CI)			
24 months event-free rate (95% CI)			
36 months event-free rate (95% CI)			
HR (95%CI)	1.00 reference)		
p value	-		

Analysis 5– Predictive value of ptDNA detection and PSA level at C1D1 and at every cycle

- ptDNA and PSA level will be compared in terms of detection of patients who experienced progressive disease at 12,18,24 and 36 months or not in a qualitative manner using frequencies, percentages, sensitivity rate and false positive rate.

Analysis 6 – The association with PFS and the change in ptDNA detection at C1D1 versus ptDNA detection at C3D1 or C4D1

- The number of patients who belong to each of the following four groups will be presented; (positive (+ve) pre-, +ve on treatment), (+ve pre-, negative (-ve) on treatment), (-ve pre-, +ve on treatment), (-ve pre-, -ve on treatment).
- PFS will be compared across the four groups using cox regression
- HRs, 95%CI and p-value from cox regression will be reported
- A landmark analysis will be performed for subgroups defined at C3D1 or C4D1

**Progression-free survival**	**Change in ptDNA detection at C1D1 with ptDNA detection at C3D1 or C4D1**			
	No change in ptDNA-	No change in ptDNA+	ptDNA+ to ptDNA-	ptDNA- to ptDNA+
N<br>N events<br>Median time in months (95% CI)<br>12 months event-free rate (95% CI)<br>24 months event-free rate (95% CI)<br>36 months event-free rate (95% CI)				
Cox regression				
HR (95%CI)	1.00 (reference)			
p value	-			
24-months RMST				
Estimates				
Difference in RMST (95%CI)	0 (reference)			
p-value				

Analysis 7 – The association with PFS andthe change in PSA at C1D1 versus PSA at C3D1 or C4D1

- The number of patients who belong to each of the following three groups will be presented; (PSA<0.01 at C1D1 and C3D1 and C4D1), (≥0.01 C3D1/C4D1 with any PSA at C1D1), (≥0.01 at C1D1 but <0.01 at C3D1 and C4D1).
- PFS will be compared across the three groups using cox regression
- HRs, 95%CI and p-value from cox regression will be reported
- A landmark analysis will be performed for subgroups defined at C3D1 or C4D1

**Progression-free survival**	**Change in PSA at C1D1 with PSA at C3D1 or C4D1**		
	<0.01 at C1D1 and C3D1 and C4D1	≥0.01 C3D1/C4D1 with any PSA at C1D1	≥0.01 at C1D1 but <0.01 at C3D1 and C4D1
N<br>N events<br>Median time in months (95% CI)<br>12 months event-free rate (95% CI)<br>24 months event-free rate (95% CI)<br>36 months event-free rate (95% CI)			

Cox regression<br>HR (95%CI)<br>p value	 1.00 (reference)<br>-		
24-months RMST<br>Estimates<br>Difference in RMST (95%CI)<br>p-value	 <br>0 (reference) 		

Analysis 8 – To compare ptDNA classification at C2D1 and C5D1 with C3D1 or C4D1

**Progression-Free-Survival**	**N**	**N events**	**Median time in months**<br><br>**(95% CI)**	**36 months event-free rate**<br><br>**(95% CI)**	**HR (95%CI)**	**p value**
**PARADIGM-A**					-	-
**ptDNA C2D1**						
Negative					1.00 (reference)	-
Positive						
**ptDNA C5D1**						
Negative					1.00 (reference)	-
Positive						
**ptDNA C3D1/C4D1**						
Negative					1.00 (reference)	-
Positive						

DocuSign Envelope ID: 487536AC-FFA9-4A62-BAA9-C8C921CC9811

Analysis 9 – The association of PFS and the effect of ptDNA fraction prior to ADT

- This analyses will be performed ion the sub-set of patients who have consented to the pre-ADT sub-study. This may be reported separately when additional samples are obtained.
- The continuous measurement of ptDNA fraction taken prior to ADT will be assessed in a descriptive manner using histograms.
- The frequency, mean, standard deviation, median and range of ptDNA fraction prior to ADT will be reported
- An unadjusted and adjusted cox regression will be used to evaluate the association between ptDNA fraction taken prior to ADT and PFS.
- If appropriate, ptDNA fraction prior to ADT will be categorised in tertitles and PFS curves will be plotted using the Kaplan Meier curve
- ROC analysis will be used to evaluate the best cut-off value of ptDNA fraction prior to ADT for detection of PFS event at 24 months, which will be selected based on the highest ratio sensitivity rate over false positive rate. The ROC analysis will account for age and ethnicity too. The ROC analysis of ptDNA will also be compared to that of PSA.

Analysis 10 – Exploratory univariate and multivariate analysis cox regression taking into account baseline characteristics

An exploratory univariate and multivariate cox model for PFS will also be performed taking into account baseline characteristics:

- Age at registration (continuous)
- Pre-ADT PSA (continuous)
- ECOG (categorical)
- Gleason score(≤7 vs ≥8)
- Visceral disease: yes vs no
- Pre-adt PSA (continuous)

Backward selection will be used to select the multivariate Cox model with a 5% significance level for terms to be removed from the model. Given the strong possibility that pre-ADT PSA and C1D1 PSA may be collinear, C1D1 PSA will be the variable included in the multivariate analysis and pre-ADT PSA will be excluded from the model if they are found to be correlated.

Analyses of Associations with Overall survival

Definition of Overall Survival

Outcome	Type of endpoint	Definition
**Overall survival (OS)**	Major/Primary	Overall survival (OS) is defined as the interval from C1D1 (start of ARSI) to death from any cause.<br><br>Patients without documented death at the time of the final analysis will be censored at the date of the last follow-up.

Analysis 1 – Descriptive statistics

- The OS rate at 12, 24 and 36 months will be reported using the Kaplan Meier method with the 95% CI.
- The median time to event will also be reported with 95% CI.
- OS curve will be depicted using Kaplan Meier plot.
- This will be done for the overall cohort and then separately for the ptDNA subgroups

Overall survival	Overall cohort
N<br>N events<br>Median time in months (95% CI)<br>12 months event-free rate (95% CI)<br>24 months event-free rate (95% CI)<br>36 months event-free rate (95% CI)	

Analysis 2 – Comparison of OS between ptDNA positive versus ptDNA negative at C3D1/C4D1(Co-Primary Analysis)

- Patients will be defined as ptDNA positive if tumour is detected at either C3D1 or C4D1. Some patients may have only one time-point. For patients with measurements at both C3D1 and C4D1, detection at either time-point will classify the patient as ptDNA positive.
- A comparison of OS between ptDNA positive and ptDNA negative patients will be performed using a log-rank test and cox regression.
- It is possible that the ptDNA test may be associated with a smaller difference in OS at, for example, 18-24 months, than the difference in the first 12-18 months. The Wilcoxon test for time-to-event analyses which gives a higher weight to events that occur earlier will also be performed.
- The proportionality of hazards (PH) assumption will be tested on the basis of Schoenfeld residuals after fitting the cox model.

  - If there is no evidence that the PH assumption is not fulfilled at the 10% significance level, then the HR, 95% CI and p-values for comparison of ptDNA groups derived from the cox model will be reported.
  - If there is evidence that the PH assumption is not fulfilled at the 10% significance level, then the difference in the Restricted Mean Survival Time (RMST) at 18 and 24 months between the ptDNA groups will be computed along with bootstrap based 95%CI and p-value.
- As a sensitivity analysis, we will also perform an adjusted Cox regression where patient tumour factors will be included as covariates in the model. Subsequent analysis will be done adjusting for sites; sites with fewer than 4 patients will be combined for this analysis.

**Overall survival**	**ptDNA status**	
	**Negative**	**Positive**
N		
N events		
Median time in months (95% CI)		
12 months event-free rate (95% CI)		
24 months event-free rate (95% CI)		
36 months event-free rate (95% CI)		
Cox regression		
HR (95%CI)	1.00 (reference)	
p value	-	
24-months RMST		
Estimates		
Difference in RMST (95%CI)	0 (reference)	
p-value		

Analysis 3 – Association between OS and ptDNA at C1D1

- The frequency and percentage of patients who are ptDNA positive and negative ptDNA at C1D1 will be reported. Frequencies will also be reported for groups split by 5-10 and >10-16 weeks of ADT.
  The association between ptDNA at C1D1 and OS will be performed by presenting KM estimates by ptDNA status; OS comparison between ptDNA status will be performed using a log-rank test and cox regression; OS will be measured from date of blood draw (ptDNA measurement taken (landmark analysis).

Analysis 4 - Association between OS and PSA level (<0.2, 0.2-4, >4ng/dl) at every cycle and at 7 months after start of ADT

- OS will be compared across the 3 PSA groups using cox regression
- HRs, 95%CI and p-value from cox regression will be reported
- A landmark analysis will be performed for PSA subgroups defined during treatment.

**Overall survival**	**PSA levels in ng/ml**		
	**≤ 0.2**	**> 0.2 to 4**	**>4**
**PSA C1D1**			
N			
N events			
Median time in months (95% CI)			
12 months event-free rate (95% CI)			
24 months event-free rate (95% CI)			
36 months event-free rate (95% CI)			
HR (95%CI)	1.00(reference)		
p value	-		
**PSA C2D1**			
N			
N events			
Median time in months (95% CI)			
12 months event-free rate (95% CI)			
24 months event-free rate (95% CI)			
36 months event-free rate (95% CI)			
HR (95%CI)	1.00 reference)		
p value	-		
**PSA C3D1/C4D1**			
N			
N events			
Median time in months (95% CI)			
12 months event-free rate (95% CI)			
24 months event-free rate (95% CI)			
36 months event-free rate (95% CI)			
HR (95%CI)	1.00 reference)		
p value	-		
**PSA C5D1**			
N			
N events			
Median time in months (95% CI)			
12 months event-free rate (95% CI)			
24 months event-free rate (95% CI)			
36 months event-free rate (95% CI)			
HR (95%CI)	1.00 reference)		
p value	-		
**PSA 7 months after start of ADT**			
N			
N events			
Median time in months (95% CI)			
12 months event-free rate (95% CI)			
24 months event-free rate (95% CI)			

Overall survival	PSA levels in ng/ml		
	≤ 0.2	> 0.2 to 4	>4
36 months event-free rate (95% CI)<br>HR (95%CI)<br>p value	 1.00 reference)<br>-		

Analysis 5 – Predictive value of ptDNA detection and PSA level at C1D1 and at every cycle

- ptDNA and PSA level will be compared in terms of detection of patients who experienced an OS event at 12,18,24 and 36 months or not in a qualitative manner using frequencies, percentages, sensitivity rate and false positive rate.

Analysis 6 – The association with OS and the change in ptDNA detection at C1D1 versus ptDNA detection at C3D1 or C4D1

- The number of patients who belong to each of the following four groups will be presented; (+ve pre-, +ve on treatment), (+ve pre-, -ve on treatment), (-ve pre-, +ve on treatment), (-ve pre-, -ve on treatment).
- OS will be compared across the four groups using cox regression
- HRs, 95%CI and p-value from cox regression will be reported
- A landmark analysis will be performed for subgroups defined at C3D1 or C4D1

Overall survival	Change in ptDNA detection at C1D1 with ptDNA detection at C3D1 or C4D1			
	No change in ptDNA-	No change in ptDNa+	ptDNA+ to ptDNA-	ptDNA- to ptDNA+
N<br>N events<br>Median time in months (95% CI)<br>12 months event-free rate (95% CI)<br>24 months event-free rate (95% CI)<br>36 months event-free rate (95% CI)				
Cox regression<br>HR (95%CI)<br>p value	 1.00 (reference)<br>-			
24-months RMST<br>Estimates<br>Difference in RMST (95%CI)<br>p-value	 <br>0 (reference) 			

**Overall survival**	**Change in ptDNA detection at C1D1 with ptDNA detection at C3D1 or C4D1**			
	No change in ptDNA-	No change in ptDNa+	ptDNA+ to ptDNA-	ptDNA- to ptDNA+

Analysis 7 – The association with OS of the change in PSA at C1D1 versus PSA at C3D1 or C4D1

- The number of patients who belong to each of the following three groups will be presented; (PSA<0.01 at C1D1 and C3D1 and/or C4D1), (≥0.01 C3D1/C4D1 with any PSA at C1D1), (≥0.01 at C1D1 but <0.01 at C3D1 and/or C4D1).
- OS will be compared across the three groups using cox regression
- HRs, 95%CI and p-value from cox regression will be reported
- A landmark analysis will be performed for subgroups defined at C3D1 or C4D1

**Overall survival**	**Change in PSA at C1D1 with PSA at C3D1 or C4D1**		
	PSA<0.01 at C1D1 and C3D1 and/or C4D1)	(≥0.01 C3D1/C4D1 with any PSA at C1D1	0.01 at C1D1 but <0.01 at C3D1 and/or C4D1
N<br>N events<br>Median time in months (95% CI)<br>12 months event-free rate (95% CI)<br>24 months event-free rate (95% CI)<br>36 months event-free rate (95% CI)			
Cox regression<br>HR (95%CI)<br>p value	 1.00 (reference)<br>-		
24-months RMST<br>Estimates<br>Difference in RMST (95%CI)<br>p-value	 <br>0 (reference)		

DocuSign Envelope ID: 487536AC-FFA9-4A62-BAA9-C8C921CC9811

Analysis 8 – To compare ptDNA classification at C2D1 and C5D1 with C3D1 or C4D1

**Overall Survival**	**N**	**N events**	**Median time months (95% CI)**	**36 months in event-free rate (95% CI)**	**HR (95%CI)**	**p value**
**PARADIGM-A**					-	-
**ptDNA C2D1**						
Negative					1.00 (reference)	-
Positive						
**ptDNA C5D1**						
Negative					1.00 (reference)	-
Positive						
**ptDNA C3D1/C4D1**						
Negative					1.00 (reference)	-
Positive						

Analysis 9 – The association of OS and the effect of ptDNA fraction prior to ADT

- This analyses will be performed on the sub-set of patients who have consented to the pre-ADT sub-study. This may be reported separately when additional samples are obtained.
- This analysis will be performed on the sub-set of patients who have pre-ADT samples. This may be reported separately when additional samples are obtained.
- The continuous measurement of ptDNA fraction taken prior to ADT will be assessed in a descriptive manner using histograms.
- The frequency, mean, standard deviation, median and range of ptDNA fraction prior to ADT will be reported

DocuSign Envelope ID: 487536AC-FFA9-4A62-BAA9-C8C921CC9811

- An unadjusted and adjusted cox regression will be used to evaluate the association between ptDNA fraction taken prior to ADT and OS.
- If appropriate, ptDNA fraction prior to ADT will be categorised in tertitles and OS curves will be plotted using the Kaplan Meier curve
- ROC analysis will be used to evaluate the best cut-off value of ptDNA fraction prior to ADT for detection of OS event at 24 months, which will be selected based on the highest ratio sensitivity rate over false positive rate. The ROC analysis will account for age and ethnicity too. The ROC analysis of ptDNA will also be compared to that of PSA.

Analysis 10 – Exploratory univariate and multivariate analysis cox regression taking into account baseline characteristics

An exploratory univariate and multivariate cox model for OS will also be performed taking into account baseline characteristics:

- Age at registration (continuous)
- Pre-ADT PSA (continuous)
- ECOG (categorical)
- Gleason score(≤7 vs ≥8)
- Visceral disease: yes vs no
- C1D1 PSA (continuous)
- Other relevant covariates

Backward selection will be used to select the multivariate Cox model with a 5% significance level for terms to be removed from the model. Given the strong possibility that pre-ADT PSA and C1D1 PSA may be collinear, C1D1 PSA will be the variable included in the multivariate analysis and pre-ADT PSA will be excluded from the model if they are found to be correlated.

Prostate Cancer Specific Survival (PCSS)

Definition of Prostate Cancer Specific Survival

Outcome	Type of endpoint	Definition
**Prostate Cancer Specific Survival (PCSS)**	Secondary	Prostate Cancer Specific Survival (PCSS) defined as interval from C1D1 to death from prostate cancer.<br><br>Patient without documented death from Prostate cancer at the time of the final analysis will be censored at the date of the last follow-up.

The results will be presented as follows:

Analysis 1 – Descriptive statistics

- The PCSS rate at 12 months, 24 and 36 months will be reported using the Kaplan Meier method along with the 95%CI.
- The median time to event will also be reported with 95% CI.
- PCSS curve will be depicted using Kaplan Meier plot.
- This will be done for the overall cohort and then separately for the ptDNA subgroups

**Prostate Cancer Specific Survival**	**PARADIGM A**
N	
N events	
Median time in months (95% CI)	
12 months event-free rate (95% CI)	
24 months event-free rate (95% CI)	
36 months event-free rate (95% CI)	

Analysis 2 – Comparison of PCSS between ptDNA positive versus ptDNA negative at C3D1/C4D1(Co-Primary Analysis)

- Patients will be defined as ptDNA positive if tumour is detected at either C3D1 or C4D1. Some patients may have only one time-point. For patients with measurements at both C3D1 and C4D1, detection at either time-point will classify the patient as ptDNA positive.

- A comparison of PCSS between ptDNA positive and ptDNA negative patients will be performed using a log-rank test and cox regression.
- It is possible that the ptDNA test may be associated with a smaller difference in PCSS at, for example, 18-24 months, than the difference in the first 12-18 months. The Wilcoxon test for time-to-event analyses which gives a higher weight to events that occur earlier will also be performed.
- The proportionality of hazards (PH) assumption will be tested on the basis of Schoenfeld residuals after fitting the cox model.
    - If there is no evidence that the PH assumption is not fulfilled at the 10% significance level, then the HR, 95% CI and p-values for comparison of ptDNA groups derived from the cox model will be reported.
    - If there is evidence that the PH assumption is not fulfilled at the 10% significance level, then the difference in the Restricted Mean Survival Time (RMST) at 18 and 24 months between the ptDNA groups will be computed along with bootstrap based 95%CI and p-value.
- As a sensitivity analysis, we will also perform an adjusted Cox regression where patient tumour factors will be included as covariates in the model. Subsequent analysis will be done adjusting for sites; sites with fewer than 4 patients will be combined for this analysis.

**Prostate Cancer Specific Survival**	**ptDNA status**	
	**Negative**	**Positive**
N		
N events		
Median time in months (95% CI)		
12 months event-free rate (95% CI)		
24 months event-free rate (95% CI)		
36 months event-free rate (95% CI)		
Cox regression		
HR (95%CI)	1.00 (reference)	
p value	-	
24-months RMST		
Estimates		
Difference in RMST (95%CI)	0 (reference)	
p-value		

Analysis 3 – Association between PCSS and ptDNA at C1D1

- The frequency and percentage of patients who are ptDNA positive and negative ptDNA at C1D1 will be reported. Frequencies will also be reported for groups split by 5-10 and >10-16 weeks of ADT.

  The association between ptDNA at C1D1 and PCSS will be performed by presenting KM estimates by ptDNA status; PCSS comparison between ptDNA status will be performed using

a log-rank test and cox regression; PCSS will be measured from date of blood draw (ptDNA measurement taken (landmark analysis).

Analysis 4 - Association between PCSS and PSA level (<0.2, 0.2-4, >4ng/dl) at every cycle and at 7 months after start of ADT

- PCSS will be compared across the 3 PSA groups using cox regression
- HRs, 95%CI and p-value from cox regression will be reported
- A landmark analysis will be performed for PSA subgroups defined during treatment.

**Prostate Cancer Specific Survival**	**PSA levels in ng/ml**		
	**≤ 0.2**	**> 0.2 to 4**	**>4**
**PSA C1D1**			
N			
N events			
Median time in months (95% CI)			
12 months event-free rate (95% CI)			
24 months event-free rate (95% CI)			
36 months event-free rate (95% CI)			
HR (95%CI)	1.00(reference)		
p value	-		
**PSA C2D1**			
N			
N events			
Median time in months (95% CI)			
12 months event-free rate (95% CI)			
24 months event-free rate (95% CI)			
36 months event-free rate (95% CI)			
HR (95%CI)	1.00 reference)		
p value	-		
**PSA C3D1/C4D1**			
N			
N events			
Median time in months (95% CI)			
12 months event-free rate (95% CI)			
24 months event-free rate (95% CI)			
36 months event-free rate (95% CI)			
HR (95%CI)	1.00 reference)		
p value	-		
**PSA C5D1**			
N			

**Prostate Cancer Specific Survival**	**PSA levels in ng/ml**		
	**≤ 0.2**	**> 0.2 to 4**	**>4**
N events			
Median time in months (95% CI)			
12 months event-free rate (95% CI)			
24 months event-free rate (95% CI)			
36 months event-free rate (95% CI)			
HR (95%CI)	1.00 reference)		
p value	-		
**PSA 7 months after start of ADT**			
N			
N events			
Median time in months (95% CI)			
12 months event-free rate (95% CI)			
24 months event-free rate (95% CI)			
36 months event-free rate (95% CI)			
HR (95%CI)	1.00 reference)		
p value	-		

Analysis 5– Predictive value of ptDNA detection and PSA level at C1D1 and at every cycle

- ptDNA and PSA level will be compared in terms of detection of patients who experienced an PCSS event at 12,18,24 and 36 months or not in a qualitative manner using frequencies, percentages, sensitivity rate and false positive rate.

Analysis 6 – The association with PCSS and the change in ptDNA detection at C1D1 versus ptDNA detection at C3D1 or C4D1

- The number of patients who belong to each of the following four groups will be presented; (+ve pre-, +ve on treatment), (+ve pre-, -ve on treatment), (-ve pre-, +ve on treatment), (-ve pre-, -ve on treatment).
- PCSS will be compared across the four groups using cox regression
- HRs, 95%CI and p-value from cox regression will be reported
- A landmark analysis will be performed for subgroups defined at C3D1 or C4D1

DocuSign Envelope ID: 487536AC-FFA9-4A62-BAA9-C8C921CC9811

**Prostate Cancer Specific Survival**	**Change in ptDNA detection at C1D1 with ptDNA detection at C3D1 or C4D1**			
	No change in ptDNA-	No change in ptDNa+	ptDNA+ to ptDNA-	ptDNA- to ptDNA+
N<br>N events<br>Median time in months (95% CI)<br>12 months event-free rate (95% CI)<br>24 months event-free rate (95% CI)<br>36 months event-free rate (95% CI)				
Cox regression<br>HR (95%CI)<br>p value	 1.00 (reference)<br>-			
24-months RMST<br>Estimates<br>Difference in RMST (95%CI)<br>p-value	 <br>0 (reference) 			

Analysis 7 – The association with PCSS withthe change in PSA at C1D1 versus PSA at C3D1 or C4D1

- The number of patients who belong to each of the following three groups will be presented; (PSA<0.01 at C1D1 and C3D1 and/or C4D1), (≥0.01 C3D1/C4D1 with any PSA at C1D1), (≥0.01 at C1D1 but <0.01 at C3D1 and/or C4D1).
- PCSS will be compared across the three groups using cox regression
- HRs, 95%CI and p-value from cox regression will be reported
- A landmark analysis will be performed for subgroups defined at C3D1 or C4D1

**Prostate Cancer Specific Survival**	**Change in PSA at C1D1 with PSA at C3D1 or C4D1**		
	Increased PSA	Decreased PSA	No change in PSA
N<br>N events<br>Median time in months (95% CI)<br>12 months event-free rate (95% CI)<br>24 months event-free rate (95% CI)<br>36 months event-free rate (95% CI)			

Cox regression<br>HR (95%CI)<br>p value<br><br>24-months RMST<br>Estimates<br>Difference in RMST (95%CI)<br>p-value	1.00 (reference)<br>-<br><br><br><br>0 (reference)		

Analysis 8 – To compare ptDNA classification at C2D1 and C5D1 with C3D1 or C4D1

**Prostate Cancer Specific Survival**	**N**	**N events**	**Median time in months (95% CI)**	**36 months event-free rate (95% CI)**	**HR (95%CI)**	**p value**
**PARADIGM-A**					-	-
**ptDNA C2D1**						
Negative					1.00 (reference)	-
Positive						
**ptDNA C5D1**						
Negative					1.00 (reference)	-
Positive						
**ptDNA C3D1/C4D1**						
Negative					1.00 (reference)	-
Positive						

DocuSign Envelope ID: 487536AC-FFA9-4A62-BAA9-C8C921CC9811

Analysis 9 – The association of PCSS and the effect of ptDNA fraction prior to ADT

- his analyses will be performed on the sub-set of patients who have consented to the pre-ADT sub-study. This may be reported separately when additional samples are obtained.
- This analysis will be performed on the sub-set of patients who have pre-ADT samples. This may be reported separately when additional samples are obtained.
- The continuous measurement of ptDNA fraction taken prior to ADT will be assessed in a descriptive manner using histograms.
- The frequency, mean, standard deviation, median and range of ptDNA fraction prior to ADT will be reported
- An unadjusted and adjusted cox regression will be used to evaluate the association between ptDNA fraction taken prior to ADT and PCSS.
- If appropriate, ptDNA fraction prior to ADT will be categorised in tertiles and PCSS curves will be plotted using the Kaplan Meier curve
- ROC analysis will be used to evaluate the best cut-off value of ptDNA fraction prior to ADT for detection of PCSS event at 24 months, which will be selected based on the highest ratio sensitivity rate over false positive rate. The ROC analysis will account for age and ethnicity too. The ROC analysis of ptDNA will also be compared to that of PSA.

Analysis 10 – Exploratory univariate analysis cox regression taking into account baseline characteristics

An exploratory univariate and multivariate cox model for PCSS will also be performed taking into account baseline characteristics:

- Age at registration (continuous)
- Pre-ADT PSA (continuous)
- ECOG (categorical)
- Gleason score(≤7 vs ≥8)
- Visceral disease: yes vs no
- C1D1 PSA (continuous)

Backward selection will be used to select the multivariate Cox model with a 5% significance level for terms to be removed from the model. Given the strong possibility that pre-ADT PSA and C1D1 PSA are collinear, C1D1 PSA will be the variable included in the multivariate analysis and pre-ADT PSA will be excluded from the model if they are found to be correlated.

The concordance between ptDNA classification at C2D1/C5D1and at C3/C4

DocuSign Envelope ID: 487536AC-FFA9-4A62-BAA9-C8C921CC9811

- The number of patients who are deemed ptDNA positive at C2D1, C3D1, C4D1 and C5D1 will be presented in tables.
- ptDNA classification concordance at C2D1 and C5D1 with C3D1 or C4D1 will be assessed in a descriptive manner by presenting the percentage of concordant and discordant cases.
- Comparisons to the number at C3D1 or C4D1 will be performed using Fisher's exact tests.

**ptDNA classification**		**Classification**	**N (%)**
C2D1	C3/C4		
Positive	Positive	Concordant	
Negative	Negative	Concordant	
Positive	Negative	Discordant	
Negative	Positive	Discordant	
C5D1	C3/C4		
Positive	Positive	Concordant	
Negative	Negative	Concordant	
Positive	Negative	Discordant	
Negative	Positive	Discordant	

The Analysis of Changes in ptDNA and PSA Levels at Different Time Points

The analysis of discordant and concordant pairs of ptDNA (positive and negative) and PSA levels (PSA ≤ 0.2 ng/ml, PSA > 0.2 to 4 ng/ml and PSA>4 ng/ml) will be conducted for every time-point with data available.

The median and range of the percentage change in ptDNA detection using continuous ptDNA and PSA will be presented qualitatively for cycles 2 through 6, compared to cycle 1. This information will be depicted in waterfall plot

COHORT PARADIGM-D

DocuSign Envelope ID: 487536AC-FFA9-4A62-BAA9-C8C921CC9811

Major outcomes:

- Progression-free survival
- Overall survival

Major objective:

- To determine whether the detection of plasma tumour DNA (ptDNA) after two or three cycles of Docetaxel added after start of ADT is associated with a worse clinical outcome in newly diagnosed metastatic prostate cancer.

Secondary outcomes

- Prostate Cancer-specific survival

Sample size

As of 2nd February 2023, PARADIGM D was closed early due to a change in practice so the 65 patients was not reached. Given that the number of required events will not be reached, the focus of the analysis will be on the estimated effect size (which is expected to be similar to the one for PARADIGM A) instead of significance levels. Analyses will be performed as for PARADIGM-A but note the difference in definition for PFS

Recruitment, follow-up, and baseline characteristics

The following should be obtained:

- The month and year between which patients were recruited (eg between January 2000 and June 2006)

DocuSign Envelope ID: 487536AC-FFA9-4A62-BAA9-C8C921CC9811

- The age range of patients recruited (eg 25 to 87 years)
- The number of centres that recruited patients
- The reverse K- M curve will be used to calculate the median follow up. The median follow-up time will be presented along with its 95% confidence interval.
- A table of baseline characteristics. This should contain age, and any other relevant factor such as disease stage, performance status, and other key biological and physiological measurements.
- For categorical variables, each column will contain N (%)
- For continuous variables, each column will contain the median value, and in brackets, the range (minimum and maximum) or IQR if more appropriate.
- The number of patients who were ineligible for primary end-point analysis and the reasons why they were ineligible
- The number of patients who were recruited to the trial but withdrew later on, and the reasons (if available), divided by whether patients withdraw from sample collection alone or sample collection and follow-up reporting.

Progression-free survival

Definition of Progression-Free Survival

Outcome	Type of endpoint	Definition
**Progression-free survival (PFS)**	Primary	Progression-free survival (PFS) is defined as the interval from the start of Docetaxel, to disease failure as determined by:

DocuSign Envelope ID: 487536AC-FFA9-4A62-BAA9-C8C921CC9811

Outcome	Type of endpoint	Definition
		• Symptomatic or asymptomatic new or unequivocal progression of prior distant metastases confirmed by imaging<br>• Symptomatic progression of cancer in the prostate confirmed by imaging<br>• Serum PSA progression<br>• Death from any cause<br><br>Patients without documented death or documented disease progression at the time of the final analysis will be censored at the date of the last follow-up<br><br>A further definition of PFS including only disease failure as confirmed by imaging or death will be considered (rPFS).

Certificate Of Completion

Envelope Id: 487536ACFFA94A62BAA9C8C921CC9811 | Status: Completed

Subject: Complete with DocuSign: PARADIGM_SAP_v1_20240416.pdf

Source Envelope:

Document Pages: 52	Signatures: 3	Envelope Originator:
Certificate Pages: 5	Initials: 0	Rubina Begum
AutoNav: Enabled		5th Floor, 90 Tottenham Court Road,
EnvelopeId Stamping: Enabled		London, London W1T4TJ
Time Zone: (UTC) Dublin, Edinburgh, Lisbon, London		rubina.begum@ucl.ac.uk
		IP Address: 128.40.163.112

Record Tracking

Status: Original<br>16 April 2024 | 15:52	Holder: Rubina Begum<br>rubina.begum@ucl.ac.uk	Location: DocuSign

Signer Events | Signature | Timestamp

Signer Events	Signature	Timestamp
Andre Lopes<br>andre.lopes@ucl.ac.uk<br>Security Level: Email, Account Authentication (None)	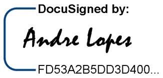 Signature Adoption: Pre-selected Style<br>Using IP Address: 128.40.163.112	Sent: 16 April 2024 | 15:59<br>Viewed: 16 April 2024 | 16:05<br>Signed: 16 April 2024 | 16:05

Electronic Record and Signature Disclosure:
Accepted: 09 November 2023 | 11:41
ID: ce2a055a-d65b-4f59-b425-e8a9fadb11cb

Signer Events	Signature	Timestamp
Memuna Rashid<br>memuna.rashid@ucl.ac.uk<br>Security Level: Email, Account Authentication (None)	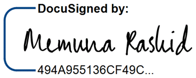 Signature Adoption: Pre-selected Style<br>Using IP Address: 128.40.163.112	Sent: 16 April 2024 | 15:59<br>Viewed: 16 April 2024 | 16:06<br>Signed: 16 April 2024 | 16:06

Electronic Record and Signature Disclosure:
Accepted: 16 April 2024 | 16:06
ID: d644dff1-a26f-4384-9ac0-06171f794170

Signer Events	Signature	Timestamp
Professor Gerhardt Attard<br>g.attard@ucl.ac.uk<br>PI<br>Security Level: Email, Account Authentication (None)	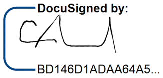 Signature Adoption: Drawn on Device<br>Using IP Address: 82.132.222.134	Sent: 16 April 2024 | 15:59<br>Viewed: 16 April 2024 | 18:16<br>Signed: 16 April 2024 | 18:16

Electronic Record and Signature Disclosure:
Accepted: 01 December 2023 | 16:27
ID: befa2d16-e3bc-4aa0-a866-5c81650241df

In Person Signer Events	Signature	Timestamp

Editor Delivery Events	Status	Timestamp

Agent Delivery Events	Status	Timestamp

Intermediary Delivery Events	Status	Timestamp

Certified Delivery Events	Status	Timestamp

Carbon Copy Events	Status	Timestamp

Witness Events	Signature	Timestamp

Notary Events	Signature	Timestamp

Envelope Summary Events	Status	Timestamps
Envelope Sent	Hashed/Encrypted	16 April 2024 | 15:59
Certified Delivered	Security Checked	16 April 2024 | 18:16
Signing Complete	Security Checked	16 April 2024 | 18:16
Completed	Security Checked	16 April 2024 | 18:16

Payment Events	Status	Timestamps

Electronic Record and Signature Disclosure

ELECTRONIC RECORD AND SIGNATURE DISCLOSURE

From time to time, CR UK and UCL Cancer Trials Centre (UCL CTC) (we, us or Company) may be required by law to provide to you certain written notices or disclosures. Described below are the terms and conditions for providing to you such notices and disclosures electronically through the DocuSign system. Please read the information below carefully and thoroughly, and if you can access this information electronically to your satisfaction and agree to this Electronic Record and Signature Disclosure (ERSD), please confirm your agreement by selecting the check-box next to 'I agree to use electronic records and signatures' before clicking 'CONTINUE' within the DocuSign system.

Getting paper copies

At any time, you may request from us a paper copy of any record provided or made available electronically to you by us. You will have the ability to download and print documents we send to you through the DocuSign system during and immediately after the signing session and, if you elect to create a DocuSign account, you may access the documents for a limited period of time (usually 30 days) after such documents are first sent to you. After such time, if you wish for us to send you paper copies of any such documents from our office to you, you will be charged a $0.00 per-page fee. You may request delivery of such paper copies from us by following the procedure described below.

Withdrawing your consent

If you decide to receive notices and disclosures from us electronically, you may at any time change your mind and tell us that thereafter you want to receive required notices and disclosures only in paper format. How you must inform us of your decision to receive future notices and disclosure in paper format and withdraw your consent to receive notices and disclosures electronically is described below.

Consequences of changing your mind

If you elect to receive required notices and disclosures only in paper format, it will slow the speed at which we can complete certain steps in transactions with you and delivering services to you because we will need first to send the required notices or disclosures to you in paper format, and then wait until we receive back from you your acknowledgment of your receipt of such paper notices or disclosures. Further, you will no longer be able to use the DocuSign system to receive required notices and consents electronically from us or to sign electronically documents from us.

All notices and disclosures will be sent to you electronically

Unless you tell us otherwise in accordance with the procedures described herein, we will provide electronically to you through the DocuSign system all required notices, disclosures, authorizations, acknowledgements, and other documents that are required to be provided or made available to you during the course of our relationship with you. To reduce the chance of you inadvertently not receiving any notice or disclosure, we prefer to provide all of the required notices and disclosures to you by the same method and to the same address that you have given us. Thus, you can receive all the disclosures and notices electronically or in paper format through the paper mail delivery system. If you do not agree with this process, please let us know as described below. Please also see the paragraph immediately above that describes the consequences of your electing not to receive delivery of the notices and disclosures electronically from us.

How to contact CR UK and UCL Cancer Trials Centre (UCL CTC):

You may contact us to let us know of your changes as to how we may contact you electronically, to request paper copies of certain information from us, and to withdraw your prior consent to receive notices and disclosures electronically as follows:
To contact us by email send messages to: ctc.regulatory@ucl.ac.uk

To advise CR UK and UCL Cancer Trials Centre (UCL CTC) of your new email address

To let us know of a change in your email address where we should send notices and disclosures electronically to you, you must send an email message to us at ctc.regulatory@ucl.ac.uk and in the body of such request you must state: your previous email address, your new email address. We do not require any other information from you to change your email address.

If you created a DocuSign account, you may update it with your new email address through your account preferences.

To request paper copies from CR UK and UCL Cancer Trials Centre (UCL CTC)

To request delivery from us of paper copies of the notices and disclosures previously provided by us to you electronically, you must send us an email to ctc.regulatory@ucl.ac.uk and in the body of such request you must state your email address, full name, mailing address, and telephone number. We will bill you for any fees at that time, if any.

To withdraw your consent with CR UK and UCL Cancer Trials Centre (UCL CTC)

To inform us that you no longer wish to receive future notices and disclosures in electronic format you may:

i. decline to sign a document from within your signing session, and on the subsequent page, select the check-box indicating you wish to withdraw your consent, or you may;

ii. send us an email to ctc.regulatory@ucl.ac.uk and in the body of such request you must state your email, full name, mailing address, and telephone number. We do not need any other information from you to withdraw consent.. The consequences of your withdrawing consent for online documents will be that transactions may take a longer time to process..

Required hardware and software

The minimum system requirements for using the DocuSign system may change over time. The current system requirements are found here: https://support.docusign.com/guides/signer-guide-signing-system-requirements.

Acknowledging your access and consent to receive and sign documents electronically

To confirm to us that you can access this information electronically, which will be similar to other electronic notices and disclosures that we will provide to you, please confirm that you have read this ERSD, and (i) that you are able to print on paper or electronically save this ERSD for your future reference and access; or (ii) that you are able to email this ERSD to an email address where you will be able to print on paper or save it for your future reference and access. Further, if you consent to receiving notices and disclosures exclusively in electronic format as described herein, then select the check-box next to 'I agree to use electronic records and signatures' before clicking 'CONTINUE' within the DocuSign system.

By selecting the check-box next to 'I agree to use electronic records and signatures', you confirm that:

- You can access and read this Electronic Record and Signature Disclosure; and
- You can print on paper this Electronic Record and Signature Disclosure, or save or send this Electronic Record and Disclosure to a location where you can print it, for future reference and access; and
- Until or unless you notify CR UK and UCL Cancer Trials Centre (UCL CTC) as described above, you consent to receive exclusively through electronic means all notices, disclosures, authorizations, acknowledgements, and other documents that are required to be provided or made available to you by CR UK and UCL Cancer Trials Centre (UCL CTC) during the course of your relationship with CR UK and UCL Cancer Trials Centre (UCL CTC).
