# Supplementary material for: Combined ctDNA and serum PSA for dynamic monitoring of metastatic prostate cancer starting first-line treatment: a prospective national cohort study
Source: Nat Cancer. 2026 May 15;7(6):915–27. doi: 10.1038/s43018-026-01172-9 (PMC13309274; doi:10.1038/s43018-026-01172-9)
Supplement: Supplementary file 2 — Reporting Summary [file 43018_2026_1172_MOESM2_ESM.pdf]

nature portfolio

Corresponding author(s): Gerhardt Attard

Last updated by author(s): 9 Nov 2025

Reporting Summary

Nature Portfolio wishes to improve the reproducibility of the work that we publish. This form provides structure for consistency and transparency in reporting. For further information on Nature Portfolio policies, see our Editorial Policies and the Editorial Policy Checklist.

Statistics

For all statistical analyses, confirm that the following items are present in the figure legend, table legend, main text, or Methods section.

n/a	Confirmed	
☐	☒	The exact sample size (*n*) for each experimental group/condition, given as a discrete number and unit of measurement
☐	☒	A statement on whether measurements were taken from distinct samples or whether the same sample was measured repeatedly
☐	☒	The statistical test(s) used AND whether they are one- or two-sided<br>*Only common tests should be described solely by name; describe more complex techniques in the Methods section.*
☐	☒	A description of all covariates tested
☐	☒	A description of any assumptions or corrections, such as tests of normality and adjustment for multiple comparisons
☐	☒	A full description of the statistical parameters including central tendency (e.g. means) or other basic estimates (e.g. regression coefficient) AND variation (e.g. standard deviation) or associated estimates of uncertainty (e.g. confidence intervals)
☐	☒	For null hypothesis testing, the test statistic (e.g. *F*, *t*, *r*) with confidence intervals, effect sizes, degrees of freedom and *P* value noted<br>*Give P values as exact values whenever suitable.*
☒	☐	For Bayesian analysis, information on the choice of priors and Markov chain Monte Carlo settings
☒	☐	For hierarchical and complex designs, identification of the appropriate level for tests and full reporting of outcomes
☒	☐	Estimates of effect sizes (e.g. Cohen's *d*, Pearson's *r*), indicating how they were calculated

Our web collection on statistics for biologists contains articles on many of the points above.

Software and code

Policy information about availability of computer code

Data collection	All the data within the PARADIGM study was collected prospectively and managed centrally by the University College London Cancer Clinical Trials Centre using Case Report Tracker (CRF) Tracker version 4.0.0.0, Macro EDC version 4.9.1.8852 and Macro Paradigm Database version: PARADIGM-20240520-0170. Blood sample and patient meta data were collected as described in the trial protocol. Sequencing data was generated by the Genomics Facility at the Institute of Cancer Research, Sutton, UK and transferred to UCL for data storage and analysis.
Data analysis	Sequencing data analysis and generation of tumour content estimates were performed using the CLONET pipeline (as described in Orlando et.al Nucleic Acid Research 2022). Pre-processing of data as per the manuscript description was conducted using a Singularity /Apptainer computational environment (Pre-processing.sif). Similarly, a separate Singularity containing all the CLONET computational tools including the PCF-SELECTv3 capture region BED file was used to generate the tumour content estimates (PCF-SELECTv3.sif). The Singularity files are available on the PCF_SELECT GitHub repository (https://github.com/demichelislab/PCF_SELECT).<br><br>Statistical tests, outcome analysis, and visualizations were generated using Stata Statistical Software: Release StataNow 18.5 SE (StataCorp LLC, College Station, TX).

For manuscripts utilizing custom algorithms or software that are central to the research but not yet described in published literature, software must be made available to editors and reviewers. We strongly encourage code deposition in a community repository (e.g. GitHub). See the Nature Portfolio guidelines for submitting code & software for further information.

Data

Policy information about availability of data

All manuscripts must include a data availability statement. This statement should provide the following information, where applicable:

- Accession codes, unique identifiers, or web links for publicly available datasets
- A description of any restrictions on data availability
- For clinical datasets or third party data, please ensure that the statement adheres to our policy

UCL and the UCL Clinical Trials Centre are obligated to protect the rights and privacy of trial participants, thereby necessitating restricted access to patient-level clinical and genomic sequencing data. De-identified participant data will be made available to researchers who are registered with an appropriate institution following publication. Methodologically sound proposals for any purpose will be considered by the trial executive committee who will have the right to review and comment on any draft manuscripts before publication. The original files and raw next-generation sequencing data generated in this study have been deposited in the European Genome-Phenome Archive (EGA) and can be downloaded from the EGA web portal (https://ega-archive.org) study number EGAS50000001357, under controlled access. Proposals should be directed to g.attard@ucl.ac.uk.

Research involving human participants, their data, or biological material

Policy information about studies with human participants or human data. See also policy information about sex, gender (identity/presentation), and sexual orientation and race, ethnicity and racism.

Reporting on sex and gender	Prostate cancer only affects people with prostates (i.e. biological males). This cohort includes people with prostate cancer irrespective of gender identity. All samples are de-identified at time of collection and all researchers are blind to gender identity and gender representation.
Reporting on race, ethnicity, or other socially relevant groupings	Patient self-reported race was collected at registration to the PARADIGM main study. This data was part of the analysis assessing difference in patients' characteristics between circulating tumour DNA positive and negative patients (Table 1 of this manuscript) as well as between treatments (Supplementary table 1)
Population characteristics	Patient characteristics of the PARADIGM clinical trial have been described in Table 1 and Supplementary tables 1 and 2 of this manuscript.
Recruitment	Recruitment details have been comprehensively described in the publication, the clinicaltrials.gov entry (https://clinicaltrials.gov/study/NCT04067713) and the trial protocol attached to the manuscript. We analysed samples from patients who were enrolled to PARADIGM and voluntarily consented to provide samples for research purposes.
Ethics oversight	The study protocol was approved by the Health Research Authority (HRA) for conduct in the United Kingdom. All participants provided signed, written, and informed consent for their samples to be used for research purposes. The trial was done in accordance with the principles of good clinical practice guidelines and the Declaration of Helsinki.

Note that full information on the approval of the study protocol must also be provided in the manuscript.

Field-specific reporting

Please select the one below that is the best fit for your research. If you are not sure, read the appropriate sections before making your selection.

☒ Life sciences ☐ Behavioural & social sciences ☐ Ecological, evolutionary & environmental sciences

For a reference copy of the document with all sections, see nature.com/documents/nr-reporting-summary-flat.pdf

Life sciences study design

All studies must disclose on these points even when the disclosure is negative.

Sample size	Sample size was predetermined for outcome analysis. All details can be found in the Study protocol and Statistical Analytical Plan included with the manuscript.
Data exclusions	No data was excluded. The inclusion and exclusion criteria for patients into the study is listed in the publication, the clinicaltrials.gov entry (https://clinicaltrials.gov/study/NCT04067713) and the trial protocol attached to the manuscript.
Replication	No experiments requiring technical or biological replicates were performed (as is convention for panel-based DNA sequencing of clinical trial samples). Two plasma samples were re-sequenced to reach the mandated minimum coverage.
Randomization	There was no randomization.
Blinding	The study was open label.

Reporting for specific materials, systems and methods

We require information from authors about some types of materials, experimental systems and methods used in many studies. Here, indicate whether each material, system or method listed is relevant to your study. If you are not sure if a list item applies to your research, read the appropriate section before selecting a response.

Materials & experimental systems

n/a	Involved in the study
☒	☐ Antibodies
☒	☐ Eukaryotic cell lines
☒	☐ Palaeontology and archaeology
☒	☐ Animals and other organisms
☐	☒ Clinical data
☒	☐ Dual use research of concern
☒	☐ Plants

Methods

n/a	Involved in the study
☒	☐ ChIP-seq
☒	☐ Flow cytometry
☒	☐ MRI-based neuroimaging

Clinical data

Policy information about clinical studies

All manuscripts should comply with the ICMJE guidelines for publication of clinical research and a completed CONSORT checklist must be included with all submissions.

Clinical trial registration	Clinicaltrails.gov: NCT04067713
Study protocol	The full trial protocol is attached with the manuscript.
Data collection	All the data within the PARADIGM study was collected prospectively and managed centrally by the University College London Cancer Clinical Trials. Blood sample and patient meta data were collected as described in the study protocol.
Outcomes	Outcome measures have been comprehensively described in the statistical analytical plan attached to the manuscript as well as detailed in the publication, the clinicaltrials.gov entry (https://clinicaltrials.gov/study/NCT04067713) and the trial protocol attached to the manuscript.

Plants

Seed stocks	N/A
Novel plant genotypes	N/A
Authentication	N/A
