# Supplementary material for: Combined ctDNA and serum PSA for dynamic monitoring of metastatic prostate cancer starting first-line treatment: a prospective national cohort study
Source: Nat Cancer. 2026 May 15;7(6):915–27. doi: 10.1038/s43018-026-01172-9 (PMC13309274; doi:10.1038/s43018-026-01172-9)
Supplement: Supplementary file 4 — Readme file for the computational pipeline for allele-informed copy number analysis of plasma DNA samples. [file 43018_2026_1172_MOESM4_ESM.pdf]

README for Nature Cancer Code Submission

Software name: PCF_SELECT

Repository: https://github.com/demichelislab/PCF_SELECT

---

1. Overview

PCF_SELECT is a computational pipeline for allele-informed copy number analysis of plasma DNA samples from metastatic prostate cancer patients, as described in:

> Orlando et al., *Allele-informed copy number evaluation of plasma DNA samples from metastatic prostate cancer patients: the PCF_SELECT consortium assay*, NAR Cancer (2022).

All the required info as per Nature Cancer submission guidelines can be found in the original repository. This file only reports the necessary infos to run a demo of the PCF_SELECT pipeline on toy bams.

2. Demo and example data

Detailed information on the PCF_SELECT pipeline (i.e. installation and dependencies) and command examples are provided in the main repository `README.md`.
The user can test the pipeline on the toy bams provided at the following link.

Once downloaded the toy bams, the user can follow the detailed instructions available at the original repository to set up and running the pipeline. In brief, the user can follow these steps:

1. Clone the GitHub repository (`git clone https://github.com/demichelislab/PCF_SELECT`)
2. Prepare the sample info file (i.e. three columns tab-separated file with absolute paths to bam files)
3. Run the PCF_SELECT pipeline (`singularity run --app pcfs ${PWD}/PCF_SELECT/singularity/versions/v3/2025_06_05/pcfselect.sif -s ${PWD}/sif.tsv -t ${PWD}/output/tmp/ -o ${PWD}/output/ -n 90`)

3. Runtime and outputs

The expected run time on a linux machine with 90 threads is: ~50 minutes. The output of the pipeline are:

```
.
├── abemus
│   ├── SNVs
```

```
│    │    ├── filtering_criteria.txt
│    │    └── toy_cfDNA
│    │         ├── 1
│    │         │    ├── chrpm_f1.tsv
│    │         │    ├── chrpm_f2.tsv
│    │         │    └── chrpm_f3.tsv
│    │         ├── 10
│    │         │    ├── chrpm_f1.tsv
│    │         │    ├── chrpm_f2.tsv
│    │         │    └── chrpm_f3.tsv
│    │         ├── 11
│    │         │    ├── chrpm_f1.tsv
│    │         │    ├── chrpm_f2.tsv
│    │         │    └── chrpm_f3.tsv
│    │         ├── 12
│    │         │    ├── chrpm_f1.tsv
│    │         │    ├── chrpm_f2.tsv
│    │         │    └── chrpm_f3.tsv
│    │         ├── 13
│    │         │    ├── chrpm_f1.tsv
│    │         │    ├── chrpm_f2.tsv
│    │         │    └── chrpm_f3.tsv
│    │         ├── 14
│    │         │    ├── chrpm_f1.tsv
│    │         │    ├── chrpm_f2.tsv
│    │         │    └── chrpm_f3.tsv
│    │         ├── 15
│    │         │    ├── chrpm_f1.tsv
│    │         │    ├── chrpm_f2.tsv
│    │         │    └── chrpm_f3.tsv
│    │         ├── 16
│    │         │    ├── chrpm_f1.tsv
│    │         │    ├── chrpm_f2.tsv
│    │         │    └── chrpm_f3.tsv
│    │         ├── 17
│    │         │    ├── chrpm_f1.tsv
│    │         │    ├── chrpm_f2.tsv
│    │         │    └── chrpm_f3.tsv
│    │         ├── 18
│    │         │    ├── chrpm_f1.tsv
│    │         │    ├── chrpm_f2.tsv
│    │         │    └── chrpm_f3.tsv
│    │         ├── 19
│    │         │    ├── chrpm_f1.tsv
│    │         │    ├── chrpm_f2.tsv
│    │         │    └── chrpm_f3.tsv
│    │         ├── 2
│    │         │    ├── chrpm_f1.tsv
│    │         │    ├── chrpm_f2.tsv
│    │         │    └── chrpm_f3.tsv
```

```
│       │           ├── 20
│       │           │   ├── chrpm_f1.tsv
│       │           │   ├── chrpm_f2.tsv
│       │           │   └── chrpm_f3.tsv
│       │           ├── 21
│       │           │   ├── chrpm_f1.tsv
│       │           │   ├── chrpm_f2.tsv
│       │           │   └── chrpm_f3.tsv
│       │           ├── 22
│       │           │   ├── chrpm_f1.tsv
│       │           │   ├── chrpm_f2.tsv
│       │           │   └── chrpm_f3.tsv
│       │           ├── 3
│       │           │   ├── chrpm_f1.tsv
│       │           │   ├── chrpm_f2.tsv
│       │           │   └── chrpm_f3.tsv
│       │           ├── 4
│       │           │   ├── chrpm_f1.tsv
│       │           │   ├── chrpm_f2.tsv
│       │           │   └── chrpm_f3.tsv
│       │           ├── 5
│       │           │   ├── chrpm_f1.tsv
│       │           │   ├── chrpm_f2.tsv
│       │           │   └── chrpm_f3.tsv
│       │           ├── 6
│       │           │   ├── chrpm_f1.tsv
│       │           │   ├── chrpm_f2.tsv
│       │           │   └── chrpm_f3.tsv
│       │           ├── 7
│       │           │   ├── chrpm_f1.tsv
│       │           │   ├── chrpm_f2.tsv
│       │           │   └── chrpm_f3.tsv
│       │           ├── 8
│       │           │   ├── chrpm_f1.tsv
│       │           │   ├── chrpm_f2.tsv
│       │           │   └── chrpm_f3.tsv
│       │           ├── 9
│       │           │   ├── chrpm_f1.tsv
│       │           │   ├── chrpm_f2.tsv
│       │           │   └── chrpm_f3.tsv
│       │           ├── X
│       │           │   ├── chrpm_f1.tsv
│       │           │   ├── chrpm_f2.tsv
│       │           │   └── chrpm_f3.tsv
│       │           ├── pmtab_F1_toy_cfDNA.tsv
│       │           ├── pmtab_F2_toy_cfDNA.tsv
│       │           ├── pmtab_F3_optimalR_toy_cfDNA.tsv
│       │           └── pmtab_F3_toy_cfDNA.tsv
│       ├── samples_info_file_rpa.tsv
│       ├── tabindex_optimalR.tsv
```

```
│   ├── table_mutations.tsv
│   └── table_mutations_nocommonSNPs.tsv
├── allelicImbalance
│   ├── ai_log2_table.chrX.csv
│   └── ai_log2_table.csv
├── annovar
│   ├── SNPs_annotation
│   │   ├── annovar_functional_annotation.tsv
│   │   ├── toy_cfDNA.exonic_variant_function
│   │   ├── toy_cfDNA.log
│   │   └── toy_cfDNA.variant_function
│   └── SNPs_no_annotation
│       └── toy_cfDNA.avinput
├── beta_computation
│   ├── betaTable.RData
│   ├── segmentation.seg
│   ├── segmentation_amplicons_log2r.tsv
│   └── toy_cfDNA.amp_log2r.RData
├── cn_snv_calls
│   ├── CN_SNVs_calls.csv
│   ├── SNVs_calls.csv
│   └── SNVs_calls_corrected.csv
├── exec.log
├── focalTables
│   ├── bed_with_rc.Rdata
│   ├── segmentation_focal.seg
│   ├── toy_cfDNA_control.RData
│   └── toy_cfDNA_target.RData
├── pacbam
│   ├── toy_cfDNA.pabs
│   ├── toy_cfDNA.pileup
│   ├── toy_cfDNA.rc
│   ├── toy_cfDNA.snps
│   ├── toy_gDNA.pabs
│   ├── toy_gDNA.pileup
│   ├── toy_gDNA.rc
│   └── toy_gDNA.snps
├── pacbamByChrom
│   ├── toy_cfDNA
│   │   ├── pileup
│   │   │   ├── toy_cfDNA_chr1.pileup
│   │   │   ├── toy_cfDNA_chr10.pileup
│   │   │   ├── toy_cfDNA_chr11.pileup
│   │   │   ├── toy_cfDNA_chr12.pileup
│   │   │   ├── toy_cfDNA_chr13.pileup
│   │   │   ├── toy_cfDNA_chr14.pileup
│   │   │   ├── toy_cfDNA_chr15.pileup
│   │   │   ├── toy_cfDNA_chr16.pileup
│   │   │   ├── toy_cfDNA_chr17.pileup
│   │   │   ├── toy_cfDNA_chr18.pileup
```

```
│       │       │       ├── toy_cfDNA_chr19.pileup
│       │       │       ├── toy_cfDNA_chr2.pileup
│       │       │       ├── toy_cfDNA_chr20.pileup
│       │       │       ├── toy_cfDNA_chr21.pileup
│       │       │       ├── toy_cfDNA_chr22.pileup
│       │       │       ├── toy_cfDNA_chr3.pileup
│       │       │       ├── toy_cfDNA_chr4.pileup
│       │       │       ├── toy_cfDNA_chr5.pileup
│       │       │       ├── toy_cfDNA_chr6.pileup
│       │       │       ├── toy_cfDNA_chr7.pileup
│       │       │       ├── toy_cfDNA_chr8.pileup
│       │       │       ├── toy_cfDNA_chr9.pileup
│       │       │       └── toy_cfDNA_chrX.pileup
│       │       └── snvs
│       │               ├── toy_cfDNA_chr1.pabs
│       │               ├── toy_cfDNA_chr10.pabs
│       │               ├── toy_cfDNA_chr11.pabs
│       │               ├── toy_cfDNA_chr12.pabs
│       │               ├── toy_cfDNA_chr13.pabs
│       │               ├── toy_cfDNA_chr14.pabs
│       │               ├── toy_cfDNA_chr15.pabs
│       │               ├── toy_cfDNA_chr16.pabs
│       │               ├── toy_cfDNA_chr17.pabs
│       │               ├── toy_cfDNA_chr18.pabs
│       │               ├── toy_cfDNA_chr19.pabs
│       │               ├── toy_cfDNA_chr2.pabs
│       │               ├── toy_cfDNA_chr20.pabs
│       │               ├── toy_cfDNA_chr21.pabs
│       │               ├── toy_cfDNA_chr22.pabs
│       │               ├── toy_cfDNA_chr3.pabs
│       │               ├── toy_cfDNA_chr4.pabs
│       │               ├── toy_cfDNA_chr5.pabs
│       │               ├── toy_cfDNA_chr6.pabs
│       │               ├── toy_cfDNA_chr7.pabs
│       │               ├── toy_cfDNA_chr8.pabs
│       │               ├── toy_cfDNA_chr9.pabs
│       │               └── toy_cfDNA_chrX.pabs
│       └── toy_gDNA
│               ├── pileup
│               │       ├── toy_gDNA_chr1.pileup
│               │       ├── toy_gDNA_chr10.pileup
│               │       ├── toy_gDNA_chr11.pileup
│               │       ├── toy_gDNA_chr12.pileup
│               │       ├── toy_gDNA_chr13.pileup
│               │       ├── toy_gDNA_chr14.pileup
│               │       ├── toy_gDNA_chr15.pileup
│               │       ├── toy_gDNA_chr16.pileup
│               │       ├── toy_gDNA_chr17.pileup
│               │       ├── toy_gDNA_chr18.pileup
│               │       ├── toy_gDNA_chr19.pileup
```

```
│       │   ├── toy_gDNA_chr2.pileup
│       │   ├── toy_gDNA_chr20.pileup
│       │   ├── toy_gDNA_chr21.pileup
│       │   ├── toy_gDNA_chr22.pileup
│       │   ├── toy_gDNA_chr3.pileup
│       │   ├── toy_gDNA_chr4.pileup
│       │   ├── toy_gDNA_chr5.pileup
│       │   ├── toy_gDNA_chr6.pileup
│       │   ├── toy_gDNA_chr7.pileup
│       │   ├── toy_gDNA_chr8.pileup
│       │   ├── toy_gDNA_chr9.pileup
│       │   └── toy_gDNA_chrX.pileup
│       └── snvs
│           ├── toy_gDNA_chr1.pabs
│           ├── toy_gDNA_chr10.pabs
│           ├── toy_gDNA_chr11.pabs
│           ├── toy_gDNA_chr12.pabs
│           ├── toy_gDNA_chr13.pabs
│           ├── toy_gDNA_chr14.pabs
│           ├── toy_gDNA_chr15.pabs
│           ├── toy_gDNA_chr16.pabs
│           ├── toy_gDNA_chr17.pabs
│           ├── toy_gDNA_chr18.pabs
│           ├── toy_gDNA_chr19.pabs
│           ├── toy_gDNA_chr2.pabs
│           ├── toy_gDNA_chr20.pabs
│           ├── toy_gDNA_chr21.pabs
│           ├── toy_gDNA_chr22.pabs
│           ├── toy_gDNA_chr3.pabs
│           ├── toy_gDNA_chr4.pabs
│           ├── toy_gDNA_chr5.pabs
│           ├── toy_gDNA_chr6.pabs
│           ├── toy_gDNA_chr7.pabs
│           ├── toy_gDNA_chr8.pabs
│           ├── toy_gDNA_chr9.pabs
│           └── toy_gDNA_chrX.pabs
├── peak_correction
│   ├── germline_distribution_shifts.Rdata
│   └── peak_shifts.tsv
├── tcEstimation
│   └── tc_estimations_CLONETv2.tsv
└── tmp
```
