# Supplementary material for: Combined ctDNA and serum PSA for dynamic monitoring of metastatic prostate cancer starting first-line treatment: a prospective national cohort study
Source: Nat Cancer. 2026 May 15;7(6):915–27. doi: 10.1038/s43018-026-01172-9 (PMC13309274; doi:10.1038/s43018-026-01172-9)
Supplement: Supplementary file 3 — Peer Review File [file 43018_2026_1172_MOESM3_ESM.pdf]

nature portfolio

# Peer Review File

# Combined circulating tumor DNA and serum prostate specific antigen for dynamic monitoring of metastatic prostate cancer starting first-line treatment: a prospective national cohort study

Corresponding Author: Professor Gerhardt Attard

**This file contains all reviewer reports in order by version, followed by all author rebuttals in order by version.**

Version 0:

Reviewer comments:

Reviewer #1

(Remarks to the Author)

Assessment of manuscript NATCANCER-A19483

In their study, Jayaram et al. applied the PCF-SELECT assay, which was previously developed as a cfDNA-based biomarker, to evaluate the association of ctDNA detection with patient survival in a cohort of patients with metastatic, hormone-sensitive prostate cancer initiating first-line treatment. As serum PSA is an established prognostic marker in this clinical setting, the secondary objective was to evaluate combinations of serum PSA categories with ctDNA positivity. This is a carefully designed multicenter study that includes a substantial number of longitudinal samples collected in a highly relevant clinical context. The findings highlight the value of ctDNA detection at baseline and during treatment, demonstrating its role as an independent prognostic factor for survival.

Major Comments
(1) What is the explanation for the surprisingly low detection rate in patients with visceral metastases (Table 1, bottom)? ctDNA-neg. patients have a higher rate of these types of metastases than ctDNA-pos. Patients?
(2) The authors imply at the end of their abstract and in the Discussion that their results could be used for treatment guidance. However, it must be clear in the Abstract and throughout the text that this a only a prognostic study; there is no indication that ctDNA is a predictive marker and there war no intervention based on the ctDNA results. Thus, it remains to be determined whether the ctDNA test can be used for guidance of treatment.
(3) The groups of Johann de Bono and Howard Scher have published large-scale studies on the prognostic value of CTCs in metastatic prostate cancer with remarable results. How do these previous results compare with the ctDNA-based results presented in this manuscript? Are CTC, ctDNA and PSA independent biomarkers in prostate cancer? The biomarker field suffers from an endless number of prognostic markers and it remains unclear whether a new marker is redundant or complimentary to an „old“ one.
(4) The authors state on page 11 that „these analyses were not under-powered. However, looking at the Kaplan Meier plots of Figure the most interesting subgroup (i.e., ctDNA-pos. patients with low PSA) constitute only a small cohort of 7 (Fig. 4a) or 8 (Fig. 4b) patients depending on the PSA cut-off selected.
(5) Fig. 4 shows that the largest subgroup (45 or 41 pats.) are patients with an increased PSA level but a negative ctDNA test. Considering the reliability of PSA as treatment monitoring marker, it is surprising that so many patients are ctDNA-neg. in this group.
(6) An important message of this study is that ctDNA detects progression earlier than PSA. While some studies support this conclusion, others are non-significant (Figure 3), and the HRs are not so impressive. This might be at least discussed.
(7) The authors could discuss the fact that ctDNA measures are economically inferior to PSA, which could hinder their clinical implementation.

Minor Comments:

Line 47: The authors should use clearer language and provide a more detailed explanation of what they mean by "more dynamic" in relation to their findings and PSA measures.
Line 126: The authors should consider finding a descriptive title for the results section that includes the available longitudinal measurements. Overall, the author should aim to ensure that the titles in the results section are consistent in their approach to specific time points, analytical endpoints and/or analysis conducted.
Figure 1C: In Figure 1C the authors need to improve the representation of the results of longitudinal measurements of individual patients. The current representation as 'Sankey plots' is confusing because it indicates a change in patient identity rather than a change in ctDNA positivity. The labelling of the plots with only sample numbers, as well as the corresponding caption, is incomplete. The authors shall either improve this or consider using 'swimmer plots' as an alternative way to illustrate their results.

(Remarks on code availability)

Reviewer #2

(Remarks to the Author)
A. Summary of the key results
This article deals with the development of blood ctDNA alone or in combination with PSA as a more dynamic marker of disease progression in men with metastatic prostate cancer. The idea is that a predictive non-invasive test will enable targeted treatment modifications to improve outcome whilst minimizing over-treatment in those who stand not to benefit. The side effects from these drugs can be onerous so this is a valid objective.
The study provides strong evidence that detection of ctDNA on diagnosis/start of treatment for metastatic prostate cancer is a significant indicator of overall and progression-free survival 12, 24 and 36 months later. Detectable ctDNA was found to be as strong a prognostic indicator as PSA, and that both markers acted as independent risk-factors for progression.

B. Originality and significance: if not novel, please include reference
The concept of the prognostic value of ctDNA in mCRPC is not in itself novel. However, the significance of the study is underlined by the fact that the authors claim that this is the first prospective study to demonstrate that detection of ctDNA at the outset of treatment is a strong predictor of higher-risk patients, within a metastatic context. It was also shown to be an earlier and more dynamic marker than PSA, which is valuable because this is clinically actionable information.

C. Data & methodology: validity of approach, quality of data, quality of presentation
It is a multicentre, prospective study. Data will be made publicly available upon request. The quality of the data is high and approach valid.

D. Appropriate use of statistics and treatment of uncertainties
Seems appropriate. However, I am not a statistician and would therefore recommend that the manuscript be reviewed by someone experienced in this specialism.

E. Conclusions: robustness, validity, reliability
The conclusions are valid.

F. Suggested improvements: experiments, data for possible revision
- Patients are classified as either ctDNA-positive or -negative rather analysing quantitative ctDNA levels and assessing the significance of higher detectable yields. This approach should be justified. For example, in Figure 1c, a threshold of 20 is used to define high ctDNA, yet it is unclear why this specific cut-off was selected. Similarly, patients are divided into three PSA thresholds in the same figure, all of which are very low relative to the cohort median PSA of over 100 ng/ml. Clarification is needed for the rationale behind these thresholds.

- Although multivariable analyses incorporating both PSA and ctDNA have been conducted, the figures (3 and 4) do not clearly convey the magnitude of benefit gained by combining the two markers compared with PSA alone. A head-to-head comparison highlighting the performance improvement of the combined approach would strengthen interpretation and provide clearer clinical relevance.

- It is also worth noting that ctDNA and PSA tests differ substantially in terms of chemistry, cost, and complexity. The discussion should address how the authors envisage implementing this combination test in clinical practice, given that it would be considerably more expensive than PSA alone. Additionally, strategies to ensure equitable access for all metastatic prostate cancer patients should be considered, to avoid disparities in who can benefit from these more advanced diagnostics

G. References: appropriate credit to previous work?
Yes

H. Clarity and context: lucidity of abstract/summary, appropriateness of abstract, introduction and conclusions
Overall the text is clear and appropriate.

(Remarks on code availability)
NA

Reviewer #3

(Remarks to the Author)
Most importantly, OS/PFS start from the time of duplet therapy, while PSA/ctDNA are measured after this start. This makes PSA and ctDNA time dependent covariates since they are not known at baseline. Since I don't see that they were analyzed as time dependent covariates, or landmark analysis was used, all of the numbers and curves stratifying OS/PFS on variables that will become known in the future are wrong. That can be easily fixed by landmark analysis or modeling it as time dependent covariate.

The definition of allelic imbalance, ctDNA fraction and ctDNA positivity are subject to arbitrary decisions and thresholds, which could be better justified and supported by data. Please confirm if these thresholds and definitions were chosen prior to data analysis or are based on these data.

The protocol required 65 evaluable patients in Paradigm A and B. Please clarify why there are only 31 in D.
OS/PFS were adjusted by numerous baseline covariates listed in line 339. Please provide univariate results for these covariates. The total number of events does not support so many covariates, especially for OS.
Primary endpoint per the protocol is PFS, for which this study was powered, while all main figures only show OS curves, and line 165 states OS is primary. Please present PFS analysis as primary.

Two treatment cohorts are quite different in patient characteristics, and potentially in PFS and OS – which has not been shown. Also the effect of ctDNA seems smaller in PARADIGM A. Pooled analysis as a primary analysis might be misleading. In addition, two cohorts have different cycle lengths, adding to potential risk of confounding. Please reconcile for a pooled analysis.

It's interesting that in Fig 3 ctDNA at cycle 5, granted it's wrong since analyzed not adjusting for time dependency, has smaller effect than cycle 2 or 5. Please comment.

Figure 4b shows correct numbers since it's cycle 1 measurement, and PSA doesn't seem to be contributing much to prediction especially among ctDNA+ patients. Interaction between PSA and ctDNA should be formally tested for all time points. Also change between cycle 1 and cycles ¾ or later should be analyzed as a potential biomarker.

Since this is potentially clinically relevant biomarker, authors should explore sensitivity, specificity, calibration and other predictive model characteristics say in predicting 1 or 5 year PFS or OS. For decision making can the author suggest a single risk score?

Later time point analyses should investigate the biases in a sample of patients with missing data.

(Remarks on code availability)

Version 1:

Reviewer comments:

Reviewer #1

(Remarks to the Author)
The modifications performed by the authors improved the quality of the manuscript and answered the questions raised during the peer-review.

(Remarks on code availability)

Reviewer #2

(Remarks to the Author)
The authors have addressed the comments and recommendations made by all three reviewers in a very thorough manner, and in doing so have clarified any areas of confusion or ambiguity. The manuscript is improved as a result and is of a very high standard adding great value to the field.

(Remarks on code availability)

Reviewer #3

(Remarks to the Author)
The authors provided detailed responses to many comments. There is still concern that adjustment for time dependent nature of ctdna is not appropriate
1) landmark analysis seems to be implemented with various start date - 3 or 4 cycles. It might introduce bias if there are systematic difference in who had blood draw at 3 vs 4 cycles. Picking a fixed time point of 4 cycles would be better. Please confirm what you mean by landmark analysis – it should be explicitly defined in the methods, e.g. later time point as baseline and excluded patients that had event or were censored prior to new baseline. in the methods it says "landmark analysis was adjusted for time from treatment start to blood draw" – that in itself is not a landmark analysis.
2) in response to critique authors state that primary time point is at 1.8 months and no individual progressed or died before that - it would be ok to use ctdna as a baseline covariate. However, this would not be appropriate for later time points and only time dependent covariate results should be reported, not previous models. For example in figure 3 it's wrong to report panel d. Panel e seems to present the right analysis but please confirm in more details how the time dependent covariate is set up. For example, once ctDNA is positive do you assume it's positive for the duration of the followup or can its value change at subsequent measurement.
3) paradigm A has 39 PFS events,28 OS events. Paradigm D has 25 PFS events, 15 OS events, based from Figure 3ab. These numbers are inconsistent with Figure 3 e number of events in a model with time dependent covariate.
It is widely recommended that multivariate model will have at least 10 events per modeled variable, including variable of interest. Thus, pooled OS analysis would have 40 events, so allowing ctDNA + 3 more, while extended table 3 still used 5 more variables ("Adjusted for age at registration, time on ADT prior to cycle 1, PSA prior to ADT, Eastern Cooperative Oncology Group performance status and study cohort 570 (ARPI or docetaxel"). This will likely result in overfit. In addition, adding variables that are not significantly associated with the outcome as shown in supplementary tables 5,6 and don't have interaction effects is just adding noise to the models.

4) please describe how 12 and 24 months discrimination was performed. Were patients who are censored before this time point discarded? Time-dependent ROC should be used unless censoring before time point is minimal
5) contrary to answer to the last question, time dependent covariates are not designed to handle bias resulting from measurements missing not at random, but I would agree that the sample size is fairly small to explore this bias.

(Remarks on code availability)

Version 2:

Reviewer comments:

Reviewer #3

(Remarks to the Author)
My comments were adequately addressed

(Remarks on code availability)

**Open Access** This Peer Review File is licensed under a Creative Commons Attribution 4.0 International License, which permits use, sharing, adaptation, distribution and reproduction in any medium or format, as long as you give appropriate credit to the original author(s) and the source, provide a link to the Creative Commons license, and indicate if changes were made.
In cases where reviewers are anonymous, credit should be given to 'Anonymous Referee' and the source.
The images or other third party material in this Peer Review File are included in the article's Creative Commons license, unless indicated otherwise in a credit line to the material. If material is not included in the article's Creative Commons license and your intended use is not permitted by statutory regulation or exceeds the permitted use, you will need to obtain permission directly from the copyright holder.
To view a copy of this license, visit https://creativecommons.org/licenses/by/4.0/

**Point-by-point replies to reviewer comments.**

Reviewers' Comments:
Reviewer #1 (Remarks to the Author):
Assessment of manuscript NATCANCER-A19483
In their study, Jayaram et al. applied the PCF-SELECT assay, which was previously developed as a cfDNA-based biomarker, to evaluate the association of ctDNA detection with patient survival in a cohort of patients with metastatic, hormone-sensitive prostate cancer initiating first-line treatment. As serum PSA is an established prognostic marker in this clinical setting, the secondary objective was to evaluate combinations of serum PSA categories with ctDNA positivity. This is a carefully designed multicenter study that includes a substantial number of longitudinal samples collected in a highly relevant clinical context. The findings highlight the value of ctDNA detection at baseline and during treatment, demonstrating its role as an independent prognostic factor for survival.

Major Comments

(1) What is the explanation for the surprisingly low detection rate in patients with visceral metastases (Table 1, bottom)? ctDNA-neg patients have a higher rate of these types of metastases than ctDNA-pos. patients?

We believe it will be too speculative to provide explanations for the observation that patients with visceral disease are more likely to be ctDNA negative after treatment has started. This was a disease characteristic that was pre-defined for inclusion in our patient characteristics table. We agree with the reviewer that this is an intriguing and unexpected observation and have added the following to **Discussion** (line 250-257):

"Given at the time of study design, there was no information on evaluation of ctDNA on treatment in this disease setting…. a higher proportion of patients with visceral disease being ctDNA negative at the primary time-point whilst interesting, will require confirmation in additional studies.

(2) The authors imply at the end of their abstract and in the Discussion that their results could be used for treatment guidance. However, it must be clear in the Abstract and throughout the text that this a only a prognostic study; there is no indication that ctDNA is a predictive marker and there war no intervention based on the ctDNA results. Thus, it remains to be determined whether the ctDNA test can be used for guidance of treatment.

We agree with the reviewer. The study was set up to assess the presence of ctDNA on treatment and its association with outcome and testing prediction is the next but as-yet unproved step in the biomarker roadmap we established. We have made the following changes:

Our abstract had concluded: "Combining ctDNA and PSA could provide an effective dynamic biomarker…" and we have changed this to "Additionally, combining ctDNA with serum PSA and clinical characteristics can improve accuracy of survival prediction and should be evaluated for ctDNA-informed treatment modification."

We have added the following to the **Discussion** Line 209:

"…the PARADIGM study results have the potential, if implemented in the next generation of clinical trials,…"

lines 269-275:

"In conclusion, this is the first prospective evaluation to report strong associations with survival for residual ctDNA. This is important as it could enable initiation of life-prolonging treatment for poor prognosis patients. Whilst more expensive than PSA, testing, ctDNA is an independent risk factor after start of doublet therapy for metastatic prostate cancer and improves the prediction of survival when combined with PSA to dynamically predict survival. These data support the conduct of clinical trials that formally assess whether modifying treatment with ADT and an ARPI based on ctDNA detection improves patient outcomes."

We have changed our concluding sentence to:

"These data support the conduct of clinical trials that formally assess whether modifying treatment with ADT and an ARPI based on ctDNA detection improves patient outcomes."

(3) The groups of Johann de Bono and Howard Scher have published large-scale studies on the prognostic value of CTCs in metastatic prostate cancer with remarkable results. How do these previous results compare with the ctDNA-based results presented in this manuscript? Are CTC, ctDNA and PSA independent biomarkers in prostate cancer? The biomarker field suffers from an endless number of prognostic markers and it remains unclear whether a new marker is redundant or complimentary to an „old" one.

The reviewer is probably referring to the study that we were involved in: "Scher HI, Heller G, Molina A, Attard G, Danila DC, Jia X, Peng W, Sandhu SK, Olmos D, Riisnaes R, McCormack R, Burzykowski T, Kheoh T, Fleisher M, Buyse M, de Bono JS. Circulating tumor cell biomarker panel as an individual-level surrogate for survival in metastatic castration-resistant prostate cancer. J Clin Oncol. 2015 Apr 20;33(12):1348-55."

This study was conducted in mCRPC previously treated with docetaxel, i.e. a cohort of patients at the opposite end of the treatment spectrum with a much higher tumor burden. In mCRPC, high baseline circulating tumor cell (CTC) count is a strong predictor of poor OS and disease progression, whereas a decrease in CTC counts after treatment associated with improved outcomes. However, in metastatic castration sensitive prostate cancer (mCSPC), it is harder to capture CTCs in comparison to ctDNA which is significantly more abundant (from ~7.5mls blood, on average there is capture of 1 to 10 CTCs versus >5000 genomes from cell free DNA). A recent study in mCSPC (Goldkorn et al, JAMA, 2024;7;(10):e2437871) demonstrated that only 11.9% of patients had ≥5 CTCs and 21.3% of patients had 1 to 4 CTCs per 7.5 mLs of blood within 30 days of ADT. We report ctDNA detection decreases on treatment and the decrease in tumour load is likely to limit the utility of CTCs after 3 or 4 cycles of treatment with an ARPI or docetaxel. We agree with the reviewer that discussing CTC work at first-line treatment is

important to allow readers to contextualise the value of ctDNA and have included this in the **Discussion** (line 218-223):

"Circulating tumor cells (CTC) associate with OS and PFS in metastatic prostate cancer but as current technologies capture a relatively small number of CTC at start of ADT or at progression after several treatment lines, the decrease in tumor load we report with sequential sampling, suggests possibly lower utility at the time-points we report in this PARADIGM study." New references 32 and 33 were added to support this statement.

At this year's large and important annual meetings of the European Society of Medical Oncology and the American Society of Clinical Oncology, the presidential sessions included three presentations of trials using ctDNA detection to inform treatment in bladder cancer, breast cancer and colorectal cancer. The reviewer is correct that the biomarker field suffers from an excess of prognostic markers that have not been implemented into clinical practice. However, ctDNA dynamics has been successfully used across several cancers and as the first, prospective evaluation in mCSPC, the PARADIGM study will be a landmark for the implementation of this biomarker for prostate cancer management.

(4) The authors state on page 11 that „these analyses were not under-powered. However, looking at the Kaplan Meier plots of Figure the most interesting subgroup (i.e., ctDNA-pos. patients with low PSA) constitute only a small cohort of 7 (Fig. 4a) or 8 (Fig. 4b) patients depending on the PSA cut-off selected.(5) Fig. 4 shows that the largest subgroup (45 or 41 pats.) are patients with an increased PSA level but a negative ctDNA test. Considering the reliability of PSA as treatment monitoring marker, it is surprising that so many patients are ctDNA-neg. in this group.

The statement "these analyses were not under-powered" on page 11 refers to testing of the primary outcome measures (PFS and OS) in PARADIGM-D. The hazard ratios in PARADIGM-D were large enough for statistically-significant results despite the smaller number of patients (**Discussion**, lines 242-245). In contrast, **Figure 4** includes secondary and exploratory analyses – see next comment.

We agree that the results from combined analysis of ctDNA and PSA may not have been expected (are surprising) – indeed, we believe this is an exciting and important result and will be of major interest to the journal's readers. Our results highlight the limitation of PSA alone at this time-point (the HR comparing patients by PSA alone is 1.59 (95% CI, 0.69 to 3.68)). We show that ctDNA identifies poor prognosis patients *independently* of PSA and this explains why so many increased PSA patients are ctDNA-negative, i.e. if all high PSA patients were ctDNA-positive, then ctDNA testing would not be an independent (or useful) risk factor.

(5) An important message of this study is that ctDNA detects progression earlier than PSA. While some studies support this conclusion, others are non-significant (Figure 3), and the HRs are not so impressive. This might be at least discussed.

We thank the reviewer for this comment and have added the following to the **Discussion** (line 238-240 and 250-257):

"Notably we observe numerically stronger associations between ctDNA and the endpoint of OS rather than PFS, which may be partly explained by the difficulty of recording radiographic progression for this group of patients."

"Given at the time of study design, there was no information on evaluation of ctDNA on treatment in this disease setting, this first study was not large enough to have sufficient power to formally test every time-point from cycle 1 to cycle 6. The protocol therefore pre-specified a time-point to prove the association with worse outcome. Additional time-points were included in exploratory analyses but tests for statistical significance provide nominal p values that could be inaccurate and some unexpected observations, such as a weaker association between OS and ctDNA at cycle 5 … whilst interesting, will require confirmation in additional studies."

(7) The authors could discuss the fact that ctDNA measures are economically inferior to PSA, which could hinder their clinical implementation.

We agree with the reviewer that the clinical utility of ctDNA will need to be great enough to justify the cost. This has been in-part addressed in the changes listed in reply to comment 2 and specifically in line 278 of the **Discussion**:

"Whilst more expensive than PSA, testing, ctDNA is an independent risk factor after start of doublet therapy for metastatic prostate cancer and improves the prediction of survival when combined with PSA …"

Minor Comments:

Line 47: The authors should use clearer language and provide a more detailed explanation of what they mean by "more dynamic" in relation to their findings and PSA measures.

We interpret the meaning for "Dynamic" as continuous or rapid change, implying greater responsive.
We have changed "more dynamic" to "more responsive" in line 200 in the **Discussion** and we have removed "dynamic" from the abstract.

There are two other changes we could make. We suggest these can be considered with editorial input.

We have retained "dynamic" in line 181 ("…a slower dynamic for PSA,…") but this could be changed to "…a slower decline of PSA,…": its use here reinforces the meaning implied by the title and addresses the reviewer's request to clarify the meaning where-ever else "dynamic" is used.

The title can also be changed as below:
"**Combined circulating tumor DNA and serum prostate specific antigen for ~~dynamic~~ monitoring of metastatic prostate cancer starting first-line treatment: a prospective national cohort study**"

This has the advantage of being a shorter title but may not convey the more responsive changes observed with ctDNA.

Line 126: The authors should consider finding a descriptive title for the results section that includes the available longitudinal measurements. Overall, the author should aim to ensure that the titles in the results section are consistent in their approach to specific time points, analytical endpoints and/or analysis conducted.

We have changed **Results** sections titles as follows:

"**Pre-ADT Sub-study**"
Changed to
"**Circulating tumor DNA detection in sequential sampling from initiation of ADT**" (line 82)

"**Secondary Time-Points and Overall Survival**"
Changed to
"**Overall survival according to circulating tumor DNA detection at secondary time-points**" (line 137)

"**Combined Circulating Tumor DNA and PSA for Predicting Survival**"
Changed to:
"**Secondary analyses of combined circulating tumor DNA and PSA for predicting overall survival**" (line 158)

Figure 1C: In Figure 1C the authors need to improve the representation of the results of longitudinal measurements of individual patients. The current representation as 'Sankey plots' is confusing because it indicates a change in patient identity rather than a change in ctDNA positivity. The labelling of the plots with only sample numbers, as well as the corresponding caption, is incomplete. The authors shall either improve this or consider using 'swimmer plots' as an alternative way to illustrate their results.

We have taken the reviewer's advice and changed **Figures 1C** and **1D** to an illustration where the patient row remains the same across sequential timepoints and cases are ordered by ctDNA positivity in the pre-ADT and sequential samples.

Reviewer #2 (Remarks to the Author):

1. Summary of the key results
This article deals with the development of blood ctDNA alone or in combination with PSA as a more dynamic marker of disease progression in men with metastatic prostate cancer. The idea is that a predictive non-invasive test will enable targeted treatment modifications to improve outcome whilst minimizing over-treatment in those who stand not to benefit. The side effects from these drugs can be onerous so this is a valid objective.

The study provides strong evidence that detection of ctDNA on diagnosis/start of treatment for metastatic prostate cancer is a significant indicator of overall and progression-free survival 12, 24 and 36 months

later. Detectable ctDNA was found to be as strong a prognostic indicator as PSA, and that both markers acted as independent risk-factors for progression.

2. Originality and significance: if not novel, please include reference
The concept of the prognostic value of ctDNA in mCRPC is not in itself novel. However, the significance of the study is underlined by the fact that the authors claim that this is the first prospective study to demonstrate that detection of ctDNA at the outset of treatment is a strong predictor of higher-risk patients, within a metastatic context. It was also shown to be an earlier and more dynamic marker than PSA, which is valuable because this is clinically actionable information.

We agree that many (including our groups) have published extensively on ctDNA for mCRPC (i.e. when disease volume is greater, consequently ctDNA levels are much higher and the disease behaves differently) but the relationship at prostate cancer presentation (metastatic castrate sensitive prostate cancer) was not known. The PARADIGM study is the first to address this knowledge gap and used an innovative and prospective, multi-center study design. This is arguably the disease state when dynamic biomarkers will have the greatest impact.

3. Data & methodology: validity of approach, quality of data, quality of presentation
It is a multicentre, prospective study. Data will be made publicly available upon request. The quality of the data is high and approach valid.

Thank you

4. Appropriate use of statistics and treatment of uncertainties
Seems appropriate. However, I am not a statistician and would therefore recommend that the manuscript be reviewed by someone experienced in this specialism.

5. Conclusions: robustness, validity, reliability
The conclusions are valid.

6. Suggested improvements: experiments, data for possible revision
- Patients are classified as either ctDNA-positive or -negative rather analysing quantitative ctDNA levels and assessing the significance of higher detectable yields. This approach should be justified.

Given <10% of patients had ctDNA ≥20% on treatment and levels are relatively low in the remainder of ctDNA positive patients, it is not feasible to test associations with outcome for a range of tumour fractions in ctDNA-positive patients. A classifier splitting patients into ctDNA-negative or -positive is consistent with MRD studies where ctDNA fraction is also low in other cancer types for example most recently as used in Powles et al, N Engl J Med. 2025 Oct 20. doi: 10.1056/NEJMoa2511885. Importantly this approach will allow clinical implementation for testing of ctDNA-informed treatment decisions as highlighted by Reviewer 1. Further information on the pre-specified classifier used in this study is included in reply to Reviewer 3.

For example, in Figure 1c, a threshold of 20 is used to define high ctDNA, yet it is unclear why this specific cut-off was selected. Similarly, patients are divided into three PSA thresholds in the same figure, all of which are very low relative to the cohort median PSA of over 100 ng/ml. Clarification is needed for the rationale behind these thresholds.

Samples ≥0.20 ctDNA fraction are highlighted as this is the lower threshold in synthetic dilutions for detection of 100% of allelic imbalance calls at every genomic site in an individual sample (Orlando et al NAR Cancer, Volume 4, Issue 2, June 2022, Figure 2C). As there are many sites of allelic imbalance across the genome, ctDNA is detected at significantly lower fractions but tumour content ≥0.20 could allow pan-genome detailed molecular analyses. The relevance of this analysis has been explained in the **Methods** (line 372) and highlighted in the **Discussion** (lines 211-215):

"By defining changes in ctDNA sequentially from prior to ADT, we show that whilst ctDNA is sufficiently abundant for molecular analyses in some patients before treatment (≥20%), a notable decrease in fraction will restrict the type of molecular studies possible after start of ADT and an ARPI. We also show in the whole cohort that persistence of ctDNA on treatment is associated with a very poor prognosis and identifies a group of urgent unmet need."

Regarding the three PSA thresholds, this is a misunderstanding that we have clarified. Median PSA of >100ng/ml refers to PSA prior to androgen deprivation therapy. We have added the following explanation of PSA values on treatment (line 71-73):

"At cycle 3 or 4, 27% (28/104) of patients had a serum PSA >4ng/mL, 52% (54/104) had a PSA of 0.2 to 4ng/mL and PSA was ≤0.2ng/mL in the remainder (21%, 22/104, **Supplementary Table 3**)."

- Although multivariable analyses incorporating both PSA and ctDNA have been conducted, the figures (3 and 4) do not clearly convey the magnitude of benefit gained by combining the two markers compared with PSA alone. A head-to-head comparison highlighting the performance improvement of the combined approach would strengthen interpretation and provide clearer clinical relevance.

We thank the reviewer for this suggestion and have added analyses to test the accuracy of prediction of survival at 12 months (**Fig. 4C** for primary endpoint and **Fig. 4D** for cycle 1 day 1) and 24 months, as also suggested by Reviewer 3. We also applied likelihood ratio tests to assess whether inclusion of ctDNA in the overall survival models with baseline clinical variables and serum PSA significantly improved model fit relative to survival models without ctDNA. These exploratory analyses have been added as a new result section (line 185-193):

"**Dynamic improvement in survival prediction when circulating tumour DNA is combined with PSA**

Overall survival models including baseline clinical variables had a better fit when including ctDNA in addition to serum PSA (likelihood $p<0.001$ for cycle 3 or 4 measurements and likelihood $p=0.012$ for cycle

1 testing). We then tested accuracy of survival prediction at 12 or 24 months after start of treatment. Firstly, adding ctDNA to serum PSA collected at cycle 3 or 4 led to an increase in AUC for predicting OS at 12 months from 0.82 (95% CI, 0.68 to 0.95) to 0.91 (95% CI, 0.79 to 1.00) (Figure 4C) and 24 months from 0.69 (95% CI, 0.57 to 0.82) to 0.73 (95% CI, 0.60 to 0.86). At cycle 1, the AUC for predicting OS at 12 months increased from 0.74 (95% CI, 0.58 to 0.91) to 0.95 (95% CI, 0.89 to 1.00) (Figure 4D) and at 24 months from 0.70 (95% CI, 0.58 to 0.82) to 0.74 (95% CI, 0.62 to 0.87)."

- It is also worth noting that ctDNA and PSA tests differ substantially in terms of chemistry, cost, and complexity. The discussion should address how the authors envisage implementing this combination test in clinical practice, given that it would be considerably more expensive than PSA alone. Additionally, strategies to ensure equitable access for all metastatic prostate cancer patients should be considered, to avoid disparities in who can benefit from these more advanced diagnostics.

We agree with the reviewer that the clinical utility of ctDNA will need to be great enough to justify the cost. This has been in-part addressed in the changes listed in reply to comment 2 and specifically in line 278 of the **Discussion**:

"Whilst more expensive than PSA, testing, ctDNA is an independent risk factor after start of doublet therapy for metastatic prostate cancer and improves the prediction of survival when combined with PSA …"

The new analyses added in reply to the previous comment on improvements in prediction when ctDNA is combined with PSA are encouraging and could justify the added cost and complexity of ctDNA testing. The assay we present has been developed for prostate cancer but can be delivered off-the shelf and does not require tumour sequencing and development of patient-specific probes. This will reduce cost and facilitate wide-spread implementation.

G. References: appropriate credit to previous work? Yes
H. Clarity and context: lucidity of abstract/summary, appropriateness of abstract, introduction and conclusions. Overall the text is clear and appropriate.

Thank you

Reviewer #2 (Remarks on code availability): NA

Note the code for CLONETv2 has been published previously and is available for download as a singularity package at the PCF_SELECT Git Hub repository (https://github.com/demichelislab/PCF_SELECT).

Reviewer #3 (Remarks to the Author):

Most importantly, OS/PFS start from the time of duplet therapy, while PSA/ctDNA are measured after this start. This makes PSA and ctDNA time dependent covariates since they are not known at baseline. Since I don't see that they were analyzed as time dependent covariates, or landmark analysis was used, all of

the numbers and curves stratifying OS/PFS on variables that will become known in the future are wrong. That can be easily fixed by landmark analysis or modeling it as time dependent covariate.

We opted to report OS/PFS from start of treatment to improve readability, given we evaluated the associations of PFS and OS with biomarkers measured at both baseline and several other time-points. In our study the time interval from cycle 1 to the primary time-point (cycles 3 or 4) is 1.8 months (only ~4% of the total follow-up time) and no individual progressed or died during this interval. It therefore should be acceptable to consider levels at time of blood draw in the modelling. Nevertheless, we agree with the reviewer and have added results from time-dependent regression analyses (**Figure 3E**) and landmark analyses (**Extended Data Table 2** for PFS and **Extended Data Table 3** OS). These analyses produced consistent findings with our primary analysis.

Lines 112-115:

Landmark analyses from time of blood draw identified a similarly strong association for ctDNA with PFS in PARADIGM-D (HR, 6.34; 95% CI, 2.02 to 19.88) but no association in PARADIGM-A (HR, 1.22; 95% CI, 0.58 to 2.57; **Extended data Table 2**).

Lines 131-133:

Landmark analysis at time of blood draw was consistent with these estimates, with median overall survival of 22.31 months (95% CI, 11.70 to not reached) for ctDNA-positive patients and not reached (95% CI, 41.5 to not reached) for ctDNA-negative patients (HR, 3.02; 95% CI, 1.52 to 5.98, **Extended Data Table 3**).

We have also added a statement in the **Discussion** (line 202-205) to explain these considerations to readers.

"Our primary analysis reported from the start of treatment to allow comparisons of median times to event for samples collected at different time-points. The time from start of treatment to primary end-point blood draw was only 4% of the total follow-up time and in landmark analysis there was a consistent effect."

The definition of allelic imbalance, ctDNA fraction and ctDNA positivity are subject to arbitrary decisions and thresholds, which could be better justified and supported by data. Please confirm if these thresholds and definitions were chosen prior to data analysis or are based on these data.

Yes, classification of samples as ctDNA positive or negative was determined utilising a pre-defined decision tree that was developed prior to inspection of any of plasma DNA sequencing data from the PARADIGM study. The decision tree has been added as **Supplementary Figure 3** and we have specified this in the **Introduction** (line 52) to ensure this is clear to readers:

"…ctDNA, from targeted next-generation sequencing classified using a pre-specified test (PCF_SELECT[31]),…"

Notably, the PCF_SELECT assay which defines allelic imbalance includes a bespoke targeted panel coupled with an ad-hoc computation pipeline (CLONET2) which was previously described (Orlando et al NAR Cancer, Volume 4, Issue 2, June 2022). The ad-hoc computational pipeline available as a Singularity package for reproducibility provides two ctDNA estimates: The first is based on overall copy number (i.e., the CLONETv2 framework described in Prand and Demichelis 2019) and the second was designed to specifically exploit the high-MAF SNPs-enriched panel design and more sensitive to low ctDNA quantities, solely based on the depth of allelic imbalance on putative hemizygous deletions. The approach for allelic imbalance detection and depth estimation was formalized in the methods of the original PCF_SELECT manuscript (Orlando et al NAR Cancer, Volume 4, Issue 2, June 2022). In three prior publications, (Orlando et al NAR Cancer, Volume 4, Issue 2, June 2022, Ruiz-Vico, European Urology Oncology, Volume 8, Issue 1, 135 – 144 and Brighi et al Clinical Cancer Research, 2025) ctDNA estimates were manually reviewed to check for the reliability of these signals. Based on analysis of 968 samples, cut-offs were identified to provide robust estimations, where manual inspection was not required. This formed the basis of the decision tree in this manuscript. We implemented this to be operator independent and enable reproducibility in future clinical studies utilising the PCF_SELECT pipeline. We have more clearly stated that the classification was pre-specified and fixed prior to analysis in the **Introduction** (line 52), **Discussion** (line 218) and **Methods** (line 371).

<u>The protocol required 65 evaluable patients in Paradigm A and B. Please clarify why there are only 31 in D.</u>

This was stated in the limitations section of our **Discussion** (line 240-242):

“Secondly, the PARADIGM-D cohort did not recruit the target number of patients due to changes in national prescribing practices. Nonetheless due to the large magnitude of the main associations with OS and PFS, these were statistically significant and therefore by definition, these analyses were not under-powered.”

<u>OS/PFS were adjusted by numerous baseline covariates listed in line 339. Please provide univariate results for these covariates. The total number of events does not support so many covariates, especially for OS.</u>

To avoid redundancy, we initially omitted the univariable results because the estimates were consistent with those from the multivariable analysis. However, for completeness, as requested by the reviewer, we have included in **Supplementary Table 5** and **6**. We have also added to the **Results** (line 110-112):

“Consistent with this in the PARADIGM-A, cohort age was the only variable associated with PFS (**Supplementary Table 5**; multivariable adjustments were not performed for PARADIGM-D due to the smaller number of events).”

and (line 130-132):

“In multivariable models including ctDNA and all clinical variables used for adjustment, ctDNA at the primary time-point was the only factor with a significant association with OS (**Supplementary Table 6**).”

In our first submission, we had included the following in the legend of **Figure 2**:

“The adjusted HR for PFS in PARADIGM-D was 5.01 (95% CI, 1.65 to 15.19). For completeness, the OS HR for ctDNA detection at cycles 3 or 4 after adjustment including timing of presentation of metastases relative to diagnosis and presence of visceral metastases was 3.92 (95% CI, 1.90 to 8.09).”

This was included to show consistency for adjusted HR in PARADIGM-D and when including an additional two potential clinical variables. However, as these two variables were imbalanced between ctDNA-negative and ctDNA-positive, and given the fewer events in PARADIGM-D, we have removed this information.

We agree that adjusting for baseline characteristics must be done with caution. We only performed MVA and report adjusted HR for the total cohort and for PARADIGM-A, to ensure sufficient events. UVA (now also provided) show consistency with adjusted analyses.

<u>Primary endpoint per the protocol is PFS, for which this study was powered, while all main figures only show OS curves, and line 165 states OS is primary. Please present PFS analysis as primary.</u>

Both PFS and OS were analysed as equal primary endpoints. PFS was used for sample size calculation but from protocol version 6.0 (dated May 2022) and in the statistical analysis plan, both PFS and OS were joint major endpoints. OS is a more definitive outcome and has greater weight clinically than PFS in advanced prostate cancer. We have discussed this in the **Discussion** (line 223-234):

“Although PFS was used to estimate study size initially, both PFS and OS were pre-specified to be joint major endpoints in later versions of the protocol, and the study was powered for both PFS and OS with sufficient follow-up. Data emerging over the course of the PARADIGM study identified a relatively moderate association between PFS and OS in mCSPC so we therefore reported associations with both PFS and OS in our primary analyses but prioritized testing of OS in secondary and exploratory analysis. Current use of PSA for initiating treatment change or new imaging can also influence time to recording of progression, potentially confounding the testing of PSA and ctDNA for predicting outcomes. Also, increasing clinical use of diffusion weighted magnetic resonance imaging and positron emission tomography for patients with a rising PSA on ADT and ARPI is further affecting the PFS endpoint. Notably we observe numerically stronger associations between ctDNA and the endpoint of OS rather than PFS, which may be partly explained by the difficulty of recording radiographic progression for this group of patients.”

The PFS data were presented alongside OS data in the main Results section “Major Outcomes according to detection of Circulating Tumor DNA at Cycle 3 or 4”. However, the comments raised suggest that this needs to be clarified. We have modified the Results section (“Major Outcomes according to detection of Circulating Tumor DNA at Cycle 3 or 4”) to first present PFS and then OS. The forest plot with the PFS

HR and 95% CI (**Fig. 2C**) has been moved to **Fig. 2A**. We have added KM plots of PFS for PARADIGM-A and PARADIGM-D in supplemental data (**Extended Data Figure 1** for primary end-points and **Extended Data Figure 2** for cycle 1).

And we have added the following to the **Methods** (line 373 – 377):

"At the outset (protocol version 1.0), PFS was the primary-endpoint with a plan to report OS after sufficient deaths at a later analysis. Due to additional funding for longer follow-up, the protocol was amended in May 2022 (changes listed on page 49, protocol version 6.0) to include both OS and PFS as major end-points, while retaining the same sample size, and report both OS and PFS concurrently when ~30% deaths had occurred."

In the sentence at line 165, we did not intend to imply that OS was the sole primary end-point. We have changed as follows:

"Having met the primary objective~~, and confirmed an association between OS and ctDNA at cycles 3 or 4,~~ we proceeded to evaluate…."

Two treatment cohorts are quite different in patient characteristics, and potentially in PFS and OS – which has not been shown. Also the effect of ctDNA seems smaller in PARADIGM A.   analysis as a primary analysis might be misleading. In addition, two cohorts have different cycle lengths, adding to potential risk of confounding. Please reconcile for a pooled analysis.

We agree that the two treatment cohorts may differ in baseline characteristics and cycle length. The pooled analysis was pre-specified for OS with adjustment for treatment cohort, based on prior evidence that overall survival does not differ between ARPI and docetaxel doublets (Sydes, et al. Annals of Oncology 2018). We have added PFS and OS data calculated for each cohort separately in **Supplementary Table 4**. Regarding OS, our study is consistent with prior studies, and we have now stated this at line 118:

"Unlike PFS, median overall survival was similar in PARADIGM-D (42.87, 95% CI 23.95 to not reached) and PARADIGM-A (49.02; 95% CI, 35.55 to not reached, **Supplementary Table 4**)."

All pooled OS analyses also included sub-group analysis of each cohort separately, reported as forest plots (**Figure 2b** and **Figure 3b**). We have added results both by cohort and in the pooled analysis adjusted for relevant confounding factors, which include treatment type, age, ECOG performance status, PSA prior to ADT, and duration of ADT prior to treatment initiation. The direction of association between ctDNA detection and outcomes was consistent across cohorts.

It's interesting that in Fig 3 ctDNA at cycle 5, granted it's wrong since analyzed not adjusting for time dependency, has smaller effect than cycle 2 or 5. Please comment.

We have added the following to the **Discussion** (line 250-257):

“Given at the time of study design, there was no information on evaluation of ctDNA on treatment in this disease setting, this first study was not large enough to have sufficient power to formally test every time-point from cycle 1 to cycle 6. The protocol therefore pre-specified a time-point to prove the association with worse outcome. Additional time-points were included in exploratory analyses but tests for statistical significance provide nominal p values that could be inaccurate and some unexpected observations, such as a weaker association between OS and ctDNA at cycle 5 … whilst interesting, will require confirmation in additional studies.”

As recommended by the reviewer, we have now included time-dependent analysis at lines 154-156:

“Time-dependent analysis (adjusted for baseline variables) demonstrated, at any given time during follow-up, patients who were ctDNA-positive had a higher hazard of death compared to patients who were ctDNA-negative (HR, 2.74; 95% CI, 1.35 to 5.59; p=0.005) (**Figure 3E**).”

Figure 4b shows correct numbers since it’s cycle 1 measurement, and PSA doesn’t seem to be contributing much to prediction especially among ctDNA+ patients. Interaction between PSA and ctDNA should be formally tested for all time points.

Whilst ctDNA is prognostic at cycle 1, PSA is not – this is both novel and surprising as highlighted by Reviewer 2 and an important result, supporting our hypotheses that ctDNA is more responsive (dynamic). We had not formally tested interaction as the absence of statistically-significant interactions in an exploratory analysis on 104 patients is unlikely to be informative. We have calculated interaction p values between ctDNA and PSA across cycles 1 to 6:

| **Cycle** | **1** | **2** | **3** | **4** | **5** | **6** |
|---|---|---|---|---|---|---|
| Interaction p value | 0.254 | 0.619 | 0.768 | 0.640 | <0.001 | 0.282 |

However, we do not believe this merits inclusion in the manuscript. We have added the following sentence to **Discussion** (line 264):

“The size of this first study also limits the interpretation of tests of interaction between biomarkers (for eg ctDNA and PSA at different time-points) and....”

Also change between cycle 1 and cycles ¾ or later should be analyzed as a potential biomarker.

We have added new analysis of ctDNA at cycle 1 and cycle 3/4 to the **Results** (lines 156-160):

“Patients who were ctDNA-positive both at cycle 1 and cycle 3 or 4 had significantly worse OS (median, 8.5 months, 95% CI, 3.7 to 13.1; HR, 17.00, 95% CI, 6.64 to 43.47, p<0.0001 when compared to patients ctDNA-negative at both time-points; **Supplementary Table 7**).”

And based on this result, added the following to the **Discussion** (lines 213-214):

"We also show in the whole cohort that persistence of ctDNA on treatment is associated with a very poor prognosis and identifies a group of urgent unmet need."

Since this is potentially clinically relevant biomarker, authors should explore sensitivity, specificity, calibration and other predictive model characteristics say in predicting 1 or 5 year PFS or OS. For decision making can the author suggest a single risk score?

We thank the reviewer for this suggestion and have added analyses to test the accuracy of prediction of survival at 12 months (**Fig. 4C** for primary endpoint and **Fig. 4D** for cycle 1 day 1) and 24 months. We have provided data for 1- and 2-year OS as there is insufficient follow-up to calculate 5-year PFS and OS (will be ~2028). We also applied likelihood ratio tests to assess whether inclusion of ctDNA in the overall survival models with baseline clinical variables and serum PSA significantly improved model fit relative to survival models without ctDNA. These exploratory analyses were also recommended by Reviewer 2 and we have now included in a new **Results** section (line 185-193):

"**Dynamic improvement in survival prediction when circulating tumour DNA is combined with PSA**

Overall survival models including baseline clinical variables had a better fit when including ctDNA in addition to serum PSA (likelihood $p<0.001$ for cycle 3 or 4 measurements and likelihood $p=0.012$ for cycle 1 testing). We then tested accuracy of survival prediction at 12 or 24 months after start of treatment. Firstly, adding ctDNA to serum PSA collected at cycle 3 or 4 led to an increase in AUC for predicting OS at 12 months from 0.82 (95% CI, 0.68 to 0.95) to 0.91 (95% CI, 0.79 to 1.00) (Figure 4C) and 24 months from 0.69 (95% CI, 0.57 to 0.82) to 0.73 (95% CI, 0.60 to 0.86). At cycle 1, the AUC for predicting OS at 12 months increased from 0.74 (95% CI, 0.58 to 0.91) to 0.95 (95% CI, 0.89 to 1.00) (Figure 4D) and at 24 months from 0.70 (95% CI, 0.58 to 0.82) to 0.74 (95% CI, 0.62 to 0.87)."

We agree with the reviewer on the importance of a clinically-implementable risk score. Reviewer 2 also commented on the use of a classifier instead of a continuous variable so we have added the following to the **Discussion** (line 280-281):

"The use of discrete categorical classifiers for ctDNA and PSA allows a path to clinical implementation, allowing assignment of patients to pre-defined prognostic subgroups."

Later time point analyses should investigate the biases in a sample of patients with missing data.

We thank the reviewer for raising this point. We agree that later time point analyses may be influenced by missing data and that further investigation of potential biases in larger datasets, will be important to validate our findings. We have added time-dependent analysis which could compensate for these biases and we have acknowledged this as a limitation in the revised **Discussion**:

"The size of this first study also limits … the ability to conduct a reliable survival bias analysis on subsets of patients with missing data."

**Re: NATCANCER-A19483B**

**Combined circulating tumor DNA and serum prostate specific antigen for dynamic monitoring of metastatic prostate cancer starting first-line treatment: a prospective national cohort study**

**Point-by-point reply to referee 3 comments**

**1) landmark analysis seems to be implemented with various start date - 3 or 4 cycles. It might introduce bias if there are systematic difference in who had blood draw at 3 vs 4 cycles. Picking a fixed time point of 4 cycles would be better. Please confirm what you mean by landmark analysis – it should be explicitly defined in the methods, e.g. later time point as baseline and excluded patients that had event or were censored prior to new baseline. in the methods it says "landmark analysis was adjusted for time from treatment start to blood draw" – that in itself is not a landmark analysis.**

We amended the landmark analysis to be fixed at day 1 cycle 4: updated results in Extended data tables 2 and 3. We have clarified this in the Methods section:

"A landmark analysis was performed, including only patients who were event-free and not censored at Cycle 4 and had ctDNA measured at Cycle 3 or Cycle 4. Survival time was measured from Day 1, Cycle 4, and models were adjusted for the time from the start of treatment to Cycle 4, Day 1 to reduce potential confounding".

**2) in response to critique authors state that primary time point is at 1.8 months and no individual progressed or died before that - it would be ok to use ctdna as a baseline covariate. However, this would not be appropriate for later time points and only time dependent covariate results should be reported, not previous models. For example in figure 3 it's wrong to report panel d. Panel e seems to present the right analysis but please confirm in more details how the time dependent covariate is set up. For example, once ctDNA is positive do you assume it's positive for the duration of the followup or can its value change at subsequent measurement**.

We agree that whilst visually informative, Figure 3 panel d could be misinterpreted – in fact we had warned against this in our discussion. We have therefore removed Figure 3 panel d and the associated text from the Results.
We have updated figure 3 panel e (now d) and revised the Methods to provide a more detailed description of the time dependent covariate analysis:

"We fitted a Cox proportional hazards model with ctDNA modelled as a time-dependent covariate to account for changes in ctDNA status over time. The follow-up of each patient was divided into intervals between consecutive assessments. For each interval, ctDNA status was assumed to remain constant until the next assessment."

**3) paradigm A has 39 PFS events,28 OS events. Paradigm D has 25 PFS events, 15 OS events, based from Figure 3ab. These numbers are inconsistent with Figure 3 e number of events in a model with time dependent covariate.**

We thank the reviewer for this careful assessment. We have clarified in the Results and Figure 3 legend that Figures 3a and 3b included only patients evaluable for the primary endpoint with ctDNA measured at cycle 1 (N=102), whilst the time-dependent analysis in Figure 3d (previously Figure 3e) includes all evaluable patients (N=104).

**4) It is widely recommended that multivariate model will have at least 10 events per modelled variable, including variable of interest. Thus, pooled OS analysis would have 40 events, so allowing ctDNA + 3 more, while**

**extended table 3 still used 5 more variables ("Adjusted for age at registration, time on ADT prior to cycle 1, PSA prior to ADT, Eastern Cooperative Oncology Group performance status and study cohort 570 (ARPI or docetaxel"). This will likely result in overfit. In addition, adding variables that are not significantly associated with the outcome as shown in supplementary tables 5,6 and don't have interaction effects is just adding noise to the models.**

We thank the reviewer for highlighting the issue of events per variable in the multivariable Cox model. Although the commonly cited "ten events per variable" guidance originates from early simulation work (Peduzzi et al. 1995), more recent studies have shown that acceptable performance can be achieved with lower ratios in many settings (Vittinghoff and McCulloch 2007). The covariates were selected a priori for their established clinical relevance rather than statistical significance within this dataset. We were reassured that despite the relatively modest number of events, the unadjusted and adjusted models yielded very similar hazard ratio estimates with overlapping confidence intervals, suggesting that the model outputs are not dependent on the number of covariates. We agree with the reviewer that future studies will have the opportunity to further evaluate these observations, and we have expanded the limitations section in the Discussion:

"Another limitation of the analysis is that it does not strictly follow the commonly cited guidelines of ten events per variable for multivariable Cox models[37,38]. This may introduce risk of over-fitting but the similarity between adjusted and unadjusted hazard ratio estimates provides reassurance regarding the model's stability despite the limited number of events; covariates were selected a priori based on established clinical relevance rather than statistical significance within this dataset."

**5) please describe how 12 and 24 months discrimination was performed. Were patients who are censored before this time point discarded? Time-dependent ROC should be used unless censoring before time point is minimal**

Yes, we had selected 12 and 24 months as no patients were censored before 24 months and all had sufficient follow-up at these timepoints. We have clarified this in the Results and revised Methods:

"…chosen as all evaluable patients had follow-up of at least 24 months."

"Time-points were chosen to have no censoring."

**5) contrary to answer to the last question, time dependent covariates are not designed to handle bias resulting from measurements missing not at random, but I would agree that the sample size is fairly small to explore this bias.**

We appreciate this clarification. The time-dependent covariate approach in our analysis was used solely to model the changes in ctDNA status over time, not to correct for bias from missing data mechanisms. We agree that missing data are not at random and this is a limitation that we had acknowledged in the Discussion:

"The size of the study … prevents us from assessing missing not-at-random mechanisms."

## Editorial requests

- We have provided a Word file of the main text in which revisions are marked by tracked changes and a text version with all changes accepted.
- We have converted Supplemental Figure 1 to Supplemental Table 1 and moved the two other supplemental figures to extended data figures.

- We have moved the tables that were in Extended data to Supplemental tables.
- We have uploaded original pdf versions of all main and extended data figures.
- We have no blots or gels.
